# GBS Mapping and Analysis of Genes Conserved between *Gossypium tomentosum* and *Gossypium hirsutum* Cotton Cultivars that Respond to Drought Stress at the Seedling Stage of the BC_2_F_2_ Generation

**DOI:** 10.3390/ijms19061614

**Published:** 2018-05-30

**Authors:** Richard Odongo Magwanga, Pu Lu, Joy Nyangasi Kirungu, Latyr Diouf, Qi Dong, Yangguang Hu, Xiaoyan Cai, Yanchao Xu, Yuqing Hou, Zhongli Zhou, Xingxing Wang, Kunbo Wang, Fang Liu

**Affiliations:** 1 State Key Laboratory of Cotton Biology/Institute of Cotton Research, Chinese Academy of Agricultural Sciences, Anyang 455000, China; magwangarichard@yahoo.com (R.O.M.); lupu1992@cricaas.com.cn (P.L.); joynk@cricaas.com.cn (J.N.K.); latyr@cricaas.com.cn (L.D.); dongqi@cricaas.com.cn (Q.D.); huygsh@163.com (Y.H.); caixy@cricaas.com.cn (X.C.); xuyanchao2016@163.com (Y.X.); houyp@cricaas.com.cn (Y.H.); zhouzl@cricaas.com.cn (Z.Z.); wangxx@cricaas.com.cn (X.W.); 2 School of Biological and Physical Sciences (SBPS), Main Campus, Jaramogi Oginga Odinga University of Science and Technology (JOOUST), Main Campus, P.O. Box 210-40601 Bondo, Kenya

**Keywords:** cotton, drought, genetic diversity, genotyping by sequencing, backcross

## Abstract

Cotton production is on the decline due to ever-changing environmental conditions. Drought and salinity stress contribute to over 30% of total loss in cotton production, the situation has worsened more due to the narrow genetic base of the cultivated upland cotton. The genetic diversity of upland cotton has been eroded over the years due to intense selection and inbreeding. To break the bottleneck, the wild cotton progenitors offer unique traits which can be introgressed into the cultivated cotton, thereby improving their performance. In this research, we developed a BC_2_F_2_ population between wild male parent, *G. tomentosum* as the donor, known for its high tolerance to drought and the elite female parent, *G. hirsutum* as the recurrent parent, which is high yielding but sensitive to drought stress. The population was genotyped through the genotyping by sequencing (GBS) method, in which 10,888 single-nucleotide polymorphism (SNP) s were generated and used to construct a genetic map. The map spanned 4191.3 cM, with average marker distance of 0.3849 cM. The map size of the two sub genomes had a narrow range, 2149 cM and 2042.3 cM for At and Dt_sub genomes respectively. A total of 66,434 genes were mined, with 32,032 (48.2%) and 34,402 (51.8%) genes being obtained within the At and Dt_sub genomes respectively. Pkinase (PF00069) was found to be the dominant domain, with 1069 genes. Analysis of the main sub family, serine threonine protein kinases through gene ontology (GO), cis element and miRNA targets analysis revealed that most of the genes were involved in various functions aimed at enhancing abiotic stress tolerance. Further analysis of the RNA sequence data and qRT-PCR validation revealed 16 putative genes, which were highly up regulated under drought stress condition, and were found to be targeted by ghr-miR169a and ghr-miR164, previously associated with *NAC*(NAM, ATAF1/2 and CUC2) and myeloblastosis (*MYB*), the top rank drought stress tolerance genes. These genes can be exploited further to aid in development of more drought tolerant cotton genotypes.

## 1. Introduction

Cotton has been the number one source of natural fibre, which over the centuries been the indispensable raw material for textile industries globally [1]. Since cotton production has become a major economic activity globally, the desire to have improved production and superior fibre qualities has been the main focus for cotton breeders [2]. For a long period of time, breeding and intensive selection for desirable agronomic traits has negatively affected the diversity, and therefore, reduced the genetic diversity of the modern cotton genotypes [3]. Cotton being a mesophytic plant, its production and fibre quality has been greatly affected by various abiotic and biotic stresses globally [4]. The development of more abiotic stress resilient cotton varieties has become a difficult due to the narrow genetic diversity of the available elite cultivars [5]. Although cotton has been described as a relatively tolerant crop, the rate of salinization of the arable land and diminishing level of fresh water for agriculture compound the desire to have super performing cotton genotypes, with higher productivity in saline soil, and with higher water use efficiency [6].

Several studies have been conducted in various agronomic crops to enhance their genetic diversity and in turn improve their performance under various stress factors [7]. A number of physiological, morphological and molecular aspects of plants to abiotic stress tolerance has been proposed, for instance, leaf morphological and anatomical features of wheat have been found to enhance cell membrane stability and water balance under drought stress [8]. In cotton, root development, closing of the stomata, cellular adaptations, photosynthesis regulation, plant hormonal synthesis such as abscisic acid (ABA) and jasmonic acid (JA) production, and reactive oxygen species (ROS) scavenging enzymes such as catalase (CAT), peroxidase (POD) among others have been strongly suggested as critical for enhancing abiotic stress tolerance [9]. The uses of wild progenitors have been of great significance in boosting the genetic diversity of various crops [10]. In rice, various new and improved genotypes have been developed by crossing the upland rice to its wild relatives [11]. Similar approaches have also been used in wheat [12]. For tetraploid upland cotton, having emerged due to polyploidization of the A and D genomes, direct crossing has been hampered due to chromosomal breakdown and or pollen grain mortality [5]. Globally, upland cotton is known for its superior fibre, high yielding ability, and versatility in a range of climatic conditions, accounting for more than 90% world fibre production [13]. Among the tetraploid cotton, two are prominent strains, *G. barbadense* known for its superior fibre quality with low lint quantity, though limited by low yield capacity even though widely grown in arid and semi-arid regions globally [14]; and *G. hirsutum*, on the other hand, which has high yielding capacity but is limited in terms of adaptability to various stress factors and relatively inferior fibre quality compared to *G. barbadense* [14]. The two upland cotton genotypes would be important varieties to improve cotton in terms of wider adaptability, with increased tolerance to various abiotic stress factors and high yield with improved qualities. However, this has not been possible due to reproductive barrier, indicated by hybrid breakdown in interspecific F_2_ and advanced generations, even though both are cultivated tetraploid species that originated from the same ancestor 1–2 million years ago [15].

The closest relative to *G. hirsutum* of the wild type is *G. tomentosum* [16]. It is endemic to the dry, saline, and rocky environment of Hawaiian island. Several studies have shown that *G. tomentosum* has an inherent ability to tolerate high levels of salinity and drought stress. Hybridization between *G. hirsutum* and *G. tomentosum* has been found to be viable, resulting in a viable offspring [17]. And thus, the introgression of favourable traits from *G. tomentosum* into the genome of upland cotton has been achieved through the development of an F_2:3_ population [18]. The F_2:3_ population has been widely used in the development of genetic maps, a microsatellite-based linkage map was developed spanning a map distance of 3320.8 cM [19], a high density genetic map with map distance of 4365.3 cM [20], and a simple sequence repeat (SSR) based linkage map of 3328.24 cM [18]. In the F_2:3_ population, the parental contribution is significantly higher, basically 50:50, and thus polymorphism between the offspring’s is relatively higher because of higher probability of gene introgression [21]. Even though several studies have been conducted in the F_2:3_ population derived from the two tetraploid cotton genotypes, little research work has been done on the backcross inbred lines (BILs). The backcross technique allows for maintenance of higher percentage of the recurrent parent genomic content and only allows for significantly low flow of genetic materials from the donor parents [22]. Backcross method had been valuable for incorporating a blast resistance gene into a rice cultivar [23]; enhancing genetic diversity in soybeans [24]; lowering grain amylose content in rice [25]; improving yield components, agronomic traits, and disease resistance in barley [26]; improving resistance to common bacterial blight in pinto bean (*Phaseolus vulgaris* L.) [27]. Furthermore, the backcross technique has also been applied in broadening the genetic base of cucumber (*Cucumis sativus* L.) [28]. Based on the previous research, signifying the importance of backcross method in enhancing the performance of crops, we developed a backcross population between *G. hirsutum* as the recurrent parent and *G. tomentosum* as the donor parent. The BC_2_F_2_ generations were then genotyped through genotyping by sequence (GBS) in order to develop a fine genetic map with broader application. The map developed was then used to determine the various genes which could have been possibly introgressed from the wild, tolerant donor parent to the recurrent parent.

## 2. Results

### 2.1. Sequencing Base Content Distribution, and Error Rate Distribution Statistics

**Figure 1:**
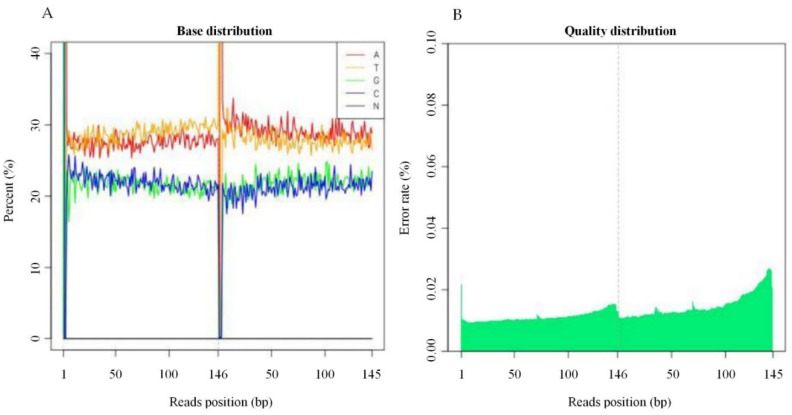
Sample Base Composition and the Error Rate Distribution. (**A**) The *x*-axis is the base of the reads base, the *y*-axis represent the order of bases from 5′ to 3′ on reads; the ordinate is all reads on the test. The percentage of bases A, C, G, T, and N, respectively, and different bases are represented by different colors. (**B**) The *x*-axis is the reads base sitting position, which represents the order of bases from 5′ to 3′ on reads; the *y*-axis are all the reads with average error rate in (%).

The base content distribution test is generally used to detect the separation of Adenine (A)-Thymine (T) and Guanine (G)-Cytosine (C), “AT” and “GC”, given the randomness of the sequence and base complementarity. The principle of pairing is that, theoretically, GC and AT contents are equal on each sequencing cycle, and they are the same throughout the sequencing process. The “N” is the base type that the sequencer cannot detect (Figure 1A). The sequencing error rate increases with the length of the sequence, caused by the consumption of chemical reagents during the sequencing process. In addition, due to the technical characteristics of Hiseq sequencing, the error rate at the end of the sequencing segment and at the end of several cycles were higher as illustrated in (Figure 1B).

### 2.2. Raw Sequencing Data Filtering Mechanism, SNP Detection, and Annotation

Illumina’s library-based sequencing platform was used to construct a sequencing library with an insertion size of approximately 400 bp. The library contraction required sequencing agreement between overlaps of paired-end reads in Massively Parallel Sequencing (MPS), also referred to as called Next Generation Sequencing (NGS), which is important in quality filtering to increase sequence accuracy. The MPS genotyping methods do not require fluorescently-labeled oligonucleotides to distinguish amplification products of similar size. Furthermore, it is not necessary to design primers within a color channel to generate amplicons of different sizes to avoid allele overlap. Consequently, all the amplicons can be of a similar, small size (typically < 275 base pairs) [29]. For marker gene analyses, the selection of primer sites within well-conserved regions aids to control the library insert size and specify the extent of overlap between paired-end reads. Alternatively, the insert size of shotgun genomic or metagenomic libraries can be constrained to a narrow range by size selection, which also controls the extent of overlap for the forward and reverse reads. Since Illumina’s raw sequencing data is likely to have some low-quality data. The data was filtered to remove the low-quality reads, five steps were followed; firstly, the adapter sequence removed in reads, the 5′ end containing non-AGCT bases before cutting removed, the reads with lower sequencing quality (sequencing quality values less than Q20) were removed, the reads containing 10% of N were removed, and finally the small fragments of less than 25 bp after the adapter and quality filtering were discarded. The data before and after mass shear were sequenced respectively: reads number, total numbers of bases, average library insert length, and Q30% were obtained (Supplementary Table S1).

Single nucleotide polymorphism (SNP), mainly refers to a single level at the genome. DNA sequence polymorphism caused by nucleotide variation is one of the highest polymorphisms in the genome. The variations of SNP type are of two kinds, the conversion and transversion type. The mutation between the same types is referred to as transition. For example, mutations between purines and purines and or between pyrimidines and pyrimidines, on the other hand, mutation between different types of bases is known as transversions, such as the variation between purines and pyrimidines. In normal condition, conversion is highly favored compared to transversion. The GATK’s Best Practices (Available online: https://software.broadinstitute.org/), was used to process the result (BAM file), using GATK’s Haplotype method of SNP detection [30].

### 2.3. Indel Detection, Annotation, and Location Distribution

**Figure 2:**
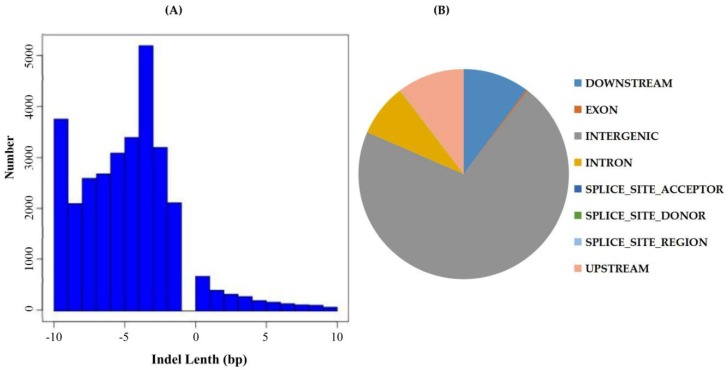
Indel Detection and Annotation. (**A**) Indel size distribution, the *y*-axis is the length distribution of the Indel (a positive value indicates the length of the insertion; a negative value indicates the length of the missing segment), and the *x*-axis is the number of Indels corresponding to the length; (**B**) Indel location information statistics.

Advancement in population and evolutionary genetic research has been a consequence of continuous improvement in the way genetic similarity or dissimilarity between genomes is assessed [31]. Currently, there is an increasing focus on polymorphisms of the type short insertions and deletions (indels) in genomic research both in animals more human and the plants in general [32]. Indels have been recognized as an abundant source of genetic markers that are widely spread across the genome, although not as common as SNPs. Genetic differences between individuals are encoded as local changes consisting of substitutions of small indels, which alter a few base pairs, and large-scale changes that consist of larger indels rearrangements and copy number variations [33]. Indels are the most common structural variant that contributes to the pathogenesis of disease in animals, and more so to human gene expression and functionality [34]. Current approaches to identify indels include de-novo assembly of unaligned reads [35], read splitting [36], depth of coverage analysis [37], and analysis of insert size inconsistencies. GATK’s was applied to compare the results of the BAM files, using GATK’s Haplotyper method to perform SNP detection and filter conditions according to GATK recommendation parameters (Available online: https://software.broadinstitute.org/). The wild donor parent, *G. tomentosum* exhibited a higher level of insertion number of 371,852, a deletion number of 352,463, a heterozygosity level of 522,862 and a homozygosity of 201,453. The recurrent parent, known to be highly affected by various environmental stresses due to massive erosion of genetic diversity, had relatively lower values of insertion (86,467), deletion number (76,206), heterozygosity (50,170), and a relatively higher level of homozygosity of 112,503. We further compared the two parental lines, with the BC_2_F_1_, the result showed that the BC_2_F_1_ generation had enhanced heterozygosity (20,895) compared to homozygosity level of 13,097. The results showed that the BC_2_F_1_ were more diverse compared to the recurrent parent; the results were also replicated among the entire BC_2_F_2_ generation, in which all were found to be more heterozygous (Supplementary Table S2). The length distributions (Figure 2A) and site locations of the indels in all the samples were determined (Figure 2B). The high number of indel markers within the intergenic regions, could possibly explain their significant roles, similar results have also been obtained in *Arabidopsis*, in which 6545 intergenic transcribed fragments (ITFs) were detected in *Arabidopsis* intergenic region and were found to be associated with known genes [38].

### 2.4. Genetic Map Construction

Backcross Inbred Lines (BILs) were developed using *G. hirsutum*, coded as CRI-12 (G09091801-2) as the recurrent parent and *G. tomentosum*, accession number AD3-00 (P0601211) as the donor parent. The parental lines were sequenced using genotyping by sequencing (GBS) method with efficient sequencing depths. An average mapped reads of ten individuals for each of the parent lines were mapped to the reference genome sequence downloaded from cotton genome (Available online: http://mascotton.njau.edu.cn). A total of 13,695,154 and 13,496,550 reads were obtained for *G. hirsutum* and *G. tomentosum* respectively. An average of 85,372 and 117,128 SNPs were identified for *G. hirsutum* and *G. tomentosum* respectively, where the efficiency of enzyme digestion was 99%. For the BC_2_F_2_ populations, the efficiency of enzyme digestion was relatively low compared to the efficiency levels of the two parental lines; the efficiency level for the BILs was 98.85%. 1,507,193,217 mapped reads were produced, with an average of 5,074,724.636 mapped reads per individual, which corresponded to nearly 186.98 Gb of clean bases. This was equivalent to approximately 83.13-fold haploid genome coverage, of raw paired-end Illumina reads by sequencing whole genome shotgun (WGS) libraries of homozygous cv. “TM-1” compared to the previous results [39]. In their study, which generated a total of 445.7 Gb of clean reads or 181-fold haploid genome coverage of raw paired-end, Illumina read by sequencing whole genome shotgun (WGS) libraries of homozygous cv. “TM-1” with fragment lengths that ranged from 250 to 40,000 bp. The average guanine-cytosine (GC) content of the sequences for the parental lines was 41.17% (*G. hirsutum*), 37.76% (*G. tomentosum*), and 43.1% (BC_2_F_1_), with a Q30 score range of 94.31% to 94.75%. The GC content range of the sequence among the mapping population (BC_2_F_2_) lines, ranged from 41.16 to 48.89% with Q30 score arrange of 93.59% to 96.02%, an indication that the sequencing process was minimal of errors. The parental lines, *G. hirsutum*-CRI-12 and *G. tomentosum*-AD3-00 were homozygous lines with “aa” and “bb” genotypes respectively. A total of 28,660 markers, after removing duplicated markers, were used for further analysis. All the SNPs generated were used because none felt below the threshold level, all had coverage of 75–100% of the entire BC_2_F_2_ population. 

**Figure 3:**
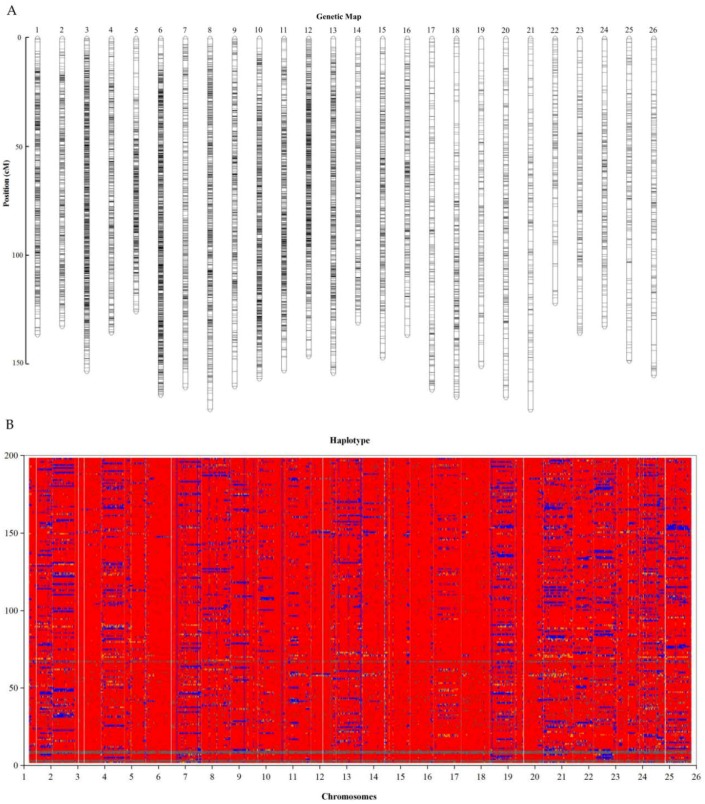
High Density Genetic Map Construction. (**A**) Genetic map. The *x*-axis: map size in centiMorgans (cM), *y*-axis: the linkage groups. (**B**) Recombination map of 200 BC_2_F_2_. Red: *G. hirsutum* blue: *G. tomentosum*.

The marker distributions on the 26 linkages ranged from 193 to 2368 in At_sub-genome (13 linkages) and 109 to 1918 in Dt_sub-genome (13 linkages). The markers were found to span 97.3–100% of the entire length of the reference genome (Supplementary Table S3). The highest marker density was observed in LG19_chrD06 (38 markers/Mb), while the lowest level of marker loci density was noted in LG18_chrD05 (2 markers/Mb). The marker distribution was asymmetric, the highest markers were found on LG19_chrD06 with 2419 markers, while the least number of markers were detected on LG18_chrD05 with only 109 translating to 0.38% of all the SNPs mapped as illustrated in genetic map (Figure 3A) and bin map (Figure 3B). The bin map showed that the markers were located within a narrow interval. Ultra-high density bin maps are significant for precise quantitative trait loci (QTL) mapping in various agricultural crops [40].

### 2.5. Fine Genetic Linkage Map Construction using the GBS-SNP Markers

In mapping the BC_2_F_2_ population, not all the 28,660 SNP markers generated were mapped, high number of markers were found to be duplicated in the same positions and with a high level of segregation distortion (SD). The highest level of markers duplication was detected in LG9_chrA09, with 824 markers found to be duplicated, which translated to 78.18% of the markers mapped in the linkage. The lowest level of markers duplication was observed in LG18_chrD05, with only 39 markers found to be duplicated (35.78%). The marker duplication is much more common in tetraploid organisms as opposed to diploid organisms, and it is believed that duplication occurs due to large and complex genome and presence of homoeologous loci from the individual sub genomes or paralogous loci from duplicated segments of the genome [41]. In order to improve the precision of the map, all the duplicated markers were removed and finally 10,888 markers were applied. All the markers were successfully linked to all the 26 linkage groups, being the mapping population of tetraploid cotton, within 2*n* = 52 chromosomes. The map size was 4191.3 cM, with 2149 cM, and 2042.3 cM in At and Dt_sub genomes respectively. The average marker distance in the map developed was 0.3849 cM, which make the map generated to the finest linkage map ever developed from the BIL population of semi-wild origin. The At_sub genome had the highest number of markers of 6318, while the Dt_sub genome contained only 4570 markers. The results obtained could be possibly explained by the variation in sizes of the two sub-genomes; At_sub genome was relatively larger than the Dt_sub genome.

The marker distributions were uneven among the linkage groups, LG6_D06 (chr25) had the highest number of marker loci of 947 with chromosome size of 158.72 cM, and an average marker distance of 0.168 cM while LG1_D01(chr15) was the lowest populated linkage, with only 45 markers with chromosomes size of 151.78 cM and average distance of 3.3728 cM. The chromosomes LG_chrA01, LG_chrA02, LG_chrA04, LG_chrA07, LG_chrA08, LG_chr11, LG_chr18 (D13), LG_chr20 (D10), LG_chr24 (D08), LG_chr25 (D06), and LG_chr26 (D12) had the highest number of markers, while LG_chr15 (D01) had the least number of markers of 45, but had the smallest gap of 0.1047 cM among all the 26 chromosomes (Table 1).

### 2.6. Evaluation of Reorganization Relationship and Collinearity of Genetic Maps and Genomes

**Figure 4:**
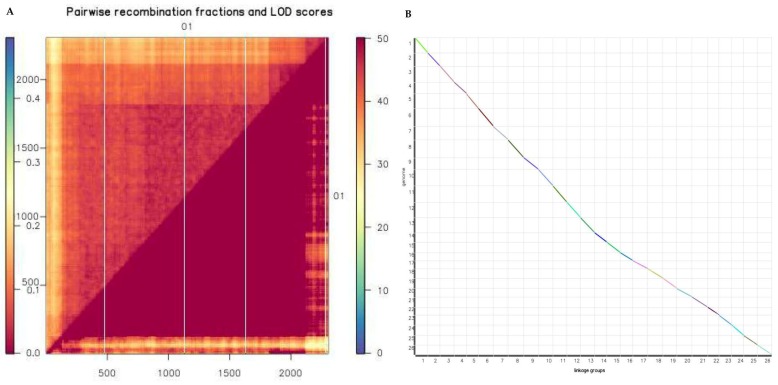
Reorganization and collinearity analysis. (**A**) Marker Chain Diagram of chr01, each row and column are markers arranged in map order. Each small square in the upper left part of the graph represents two Markers, left bar: the average marker distance, right bar: the LOD values. (**B**) Syntenic analysis. The *x*-axis is the physical distance of each chromosome, and the *y*-axis is the genetic length of each linkage group, where marker is represented in the form of a scatter. Genome and genetic map collinearity. The more diagonal the mark, the better the collinearity between the genetic map and the genome.

The genetic map is essentially a multipoint recombination analysis. The closer the marker distance, the smaller the recombination rate. The analysis of the marker reorganization in the entire genetic map was performed in order to determine defective markers in the sequencing work of the BC_2_F_2_ progenies. The map generated showed that the markers had a lower recombination frequency due to relatively narrow distance between markers. The narrower the distance, the higher the precision level of the genetic map for detection of reliable quantitative traits within the mapping population [42]. The map generated showed that the logarithm of odds (LOD) values were above the noise level. The closer the color is to red, the larger the LOD value is, while the closer to blue, the lower the LOD as evident in chr01, which had a total of 772 markers, with a map size of 185.4601 cM and average marker distance of 0.24 cM (Figure 4A). Colinearity analysis was performed; the results obtained showed effective synteny between the genetic map generated and the physical map in relation to the reference genome (Figure 4B). 

### 2.7. Gene Mining within the GBS Marker Regions of the Mapping Population

Based on the physical position of the GBS markers per linkage, we utilized the various physical positions in base pair to mine the genes. The genes mined were of different domain, in total 66,434 genes were mined, with 32,032 (48.2%) and 34,402 (51.8%) genes being obtained within the At and Dt_sub genomes respectively. The results obtained were in agreement to previous publications in which Dt_sub genome had been found to harbor more genes than the At_sub genome despite the genome size variation, where At_sub genome is relatively larger compared to Dt_sub genome [43]. The gene proportions varied across the linkage groups, the highest percentage of genes was noted for Dt_chr09 (96.3%) while the smallest proportion was detected for At_chr05 with only 72% of the genes mined. It is of interest to note that the proportions of the genes mined were not positively correlated to the number of markers per linkage, for instance, LG22_chrD09 of the Dt_sub genome had the highest number of genes even though the marker numbers were low, with only 852, compared to other linkages such as LG19_chrD06 (2419 markers), LG23_chrD10 (1854 markers), LG25_chrD12 (1593), and LG26_chrD13 (1230) in Dt_sub genome and even compared to those in At_sub genome as illustrated in Table 2. The high number of genes detected in LG22_chrD08 (chr23) indicated that the chromosome could be harboring some important traits, which are vital for aiding plants tolerance to various abiotic stress factors, such as drought stress. In a number of research findings on the analysis of various important drought tolerant traits in upland cotton, Chr23 (LG22_chrD08) has been found to harbor various significant traits known to be important for enhancing drought tolerance in plants, fresh root weight (FRW) [44], relative water content (RLWC), and excise leaf water loss (ELWL) [45]. Generally, more QTLs have been detected in the Dt_sub genome compared to the At_sub gnomes [46], an indication that the Dt_sub genome plays a major role in enhancing tolerance level of the tetraploid cotton to various abiotic stress factors.

We examined the mined genes in order to determine the various functional domains, 6141 gene domains were obtained, at 3007 and 3134 domains in the At and Dt_sub genomes respectively. The lowest number of gene domains was noted in At_chr04 with only 202 (3.3%), while the highest domain numbers was observed for Dt_chr05 with 1128 (18.4%) gene domains. It was technically impossible to analyse all the domains obtained in each linkage group. Therefore, we determined the frequency of each domain, the domains with the highest frequency was finally analysed, thus termed as the dominant domains (Supplementary Table S4). In the dominant domain, the genes were found to belong to the functional domain of Pkinase (PF00069), with a total of 1069 genes. The protein kinase (PF00069) domain contains proteins with highly conserved structure and mainly functions as catalytic protein kinases [47,48]. The protein kinases are mainly involved in the process of phosphorylation, which are of a regulatory role in metabolism, transcription, cell cycle progression, cytoskeletal rearrangement and cell movement, apoptosis, and differentiation. In animals, the protein kinases have been found to be integral in embryonic development, physiological responses, and in the nervous and immune systems. Abnormal phosphorylation causes many human diseases, including cancer, and drugs that affect phosphorylation can treat those diseases [49]. In the Pkinase domain (PF00069), we identified 44 different sub domains, 3-phosphoinositide-dependent protein kinase (four genes), Brassinosteroid insensitive 1-associated receptor kinase (three genes), Calcium and calcium/calmodulin-dependent serine/threonine-protein kinase (2 genes), Calcium-dependent protein kinase (79 genes), Calmodulin-binding receptor-like cytoplasmic kinase (five genes), Casein kinase (five genes), Casein kinase I homolog (five genes), Casein kinase I isoform α (six genes), Casein kinase I isoform β (five genes), Casein kinase I isoform δ-like (31 genes), Casein kinase II subunit α, chloroplastic (five genes), Casein kinase II subunit α-1 (seven genes), CBL-interacting protein kinase (81 genes), CDPK-related kinase 1 (16 genes), cell division control protein (seven genes), Cyclin-dependent kinase (24 genes), Cysteine-rich receptor-like protein kinase (two genes), Mitogen-activated protein kinase (111 genes), probable receptor-like protein kinase (208 genes), and Serine/threonine-protein kinase (271 genes) among others. In all the various sub families, Serine/threonine-protein kinase gene members were the most abundant, with 271 genes. 

Based on the number of the various sub families of the Pkinases, we analysed the members of Serine/threonine-protein kinase, as they were the most abundant. Forty four (44) sub classes of the Pkinases sub family were identified, Serine/threonine-protein kinase 16/38 (seven genes), *AFC1/2/3* (seven genes), *AGC1-7* (two genes), *At3g07070* (six genes), *ATG1a/c* (four genes), *ATG1t* (one gene), *AtPK2/AtPK19* (nine genes), *Aurora-1/3* (four genes), *BLUS1* (13 genes), *CBK1* (six genes), *CDL1* (ten genes), *D6PKL2* (16 genes), *dst1* (four genes), *fray2* (19 genes), *Seri GRIK2* (four genes), *HT1* (one gene), *KIPK* (six genes), *MHK* (four genes), *mph1* (three genes), *Nek2* (20 genes), *OSR1* (one gene), O*XI1* (three genes), and *PBS1* (26 genes) among other as illustrated in the (Supplementary Table S5). The high number of the serine/threonine-protein kinases sub group could mean that the genes transgressed from the tolerant genotype, in to the semi wild mapping population might be linked to various stress factors tolerance.

### 2.8. Chromosomes Mapping of the Genes Mined for the Dominant Domain, Pkinase

**Figure 5:**
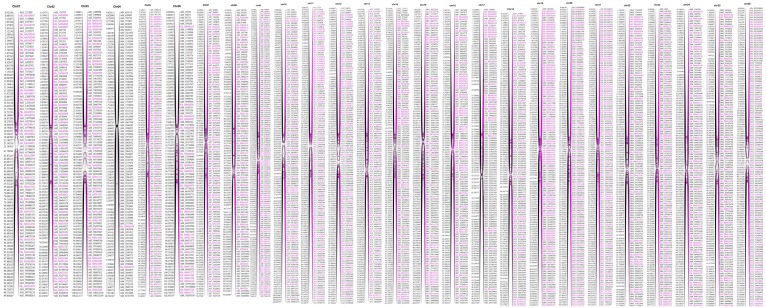
The 26 linkage groups of the tetraploid cotton in which all the genes were mapped. Pink: are the various genes, Black: the flanking GBS markers. The positions are in mega base pairs.

Chromosome mapping is critical in determining the distribution of the genes across the linkage groups. We sought to determine the distributions of the 1069 genes mined for the dominant domain, Pkinase (PF00069). The genes were skewed towards the Dt_sub genome, with 594 genes found to be mapped in Dt_sub genome, accounting for 56% of all the genes found to belong to the Pkinase, as mined within the GBS markers in the entire genome. The D genome is known to harbour very significant genes, and in various mapping for various quantitative trait loci (QTLs), controlling either phenotypic traits or fiber qualities, higher proportions are found to be located in Dt_sub genome as opposed to At_sub genome [50]. The highest number of genes, were found to be located in chr19 (D05), with 75 genes, while the least number of genes were mapped in chr04, with only two genes (Figure 5). We observed some element of gene loss between homologous chromosomal pairs, for instance chr04 and its homolog chr22, had two and 30 genes respectively, this phenomena was evident across the entire chromosomes. 

### 2.9. RNA Sequence Data of the Genes of the Pkinase Domain

RNA sequence is a critical tool which provides a summative role of the genes. It provides information in relation to the abundance of the genes in various plant tissues and expression levels when plants are exposed various abiotic and biotic stress elicitors. Among all the members of the Pkinase domain, we analysed the RNA sequence data for the dominant sub family, the Serine/threonine-protein kinases. The entire dominant sub family was 271 genes, their RNA sequence data were downloaded from cotton functional genome project, transformed into log10, in order to determine the abundance and expression levels under abiotic stress factors, such as drought and salt stress.

The genes were basically clustered in to four main groups, group 1 was highly up regulated, followed by group 3, while group 2 and 4 were mainly down regulated (Supplementary Figure S1). In group 1, had 21 genes, all were highly up regulated in both salt and drought stress conditions, among them were, *Gh_A12G1556*, *Gh_D11G0352*, *Gh_A13G0314*, *Gh_D12G1659*, *Gh_D06G2249*, *Gh_A02G0300*, *Gh_D06G1942*, *Gh_A12G0364*, *Gh_D06G2142*, *Gh_D13G1374*, *Gh_D07G0582*, *Gh_A09G0713*, *Gh_D12G0859*, *Gh_A12G0641*, *Gh_D11G1445*, *Gh_A11G1297*, *Gh_D01G1809*, *Gh_A01G1558*, *Gh_A06G1729*, and *Gh_D07G1063*. Majority of the highly up regulated genes, were mainly members of Serine/threonine-protein kinase *D6PKL2* (four genes), Shaggy-related protein kinase *eta* (three genes), while the other sub groups either had two or one genes each, such as Serine/threonine-protein kinase *STN7*, chloroplastic, Serine/threonine-protein kinase *MHK*, Serine/threonine-protein kinase *SRK2I*, and Shaggy-related protein kinase *kappa*, which had two genes each. The very lowest numbers of genes were observed for Serine/threonine-protein kinase *WNK1*, Serine/threonine-protein kinase *SRK2B*, Serine/threonine-protein kinase *AtPK2/AtPK19*, Formin-like protein 20, and Serine/threonine-protein kinase 16 with one gene each.

### 2.10. miRNA Target Analysis of the 271 Dominant Genes

**Figure 6:**
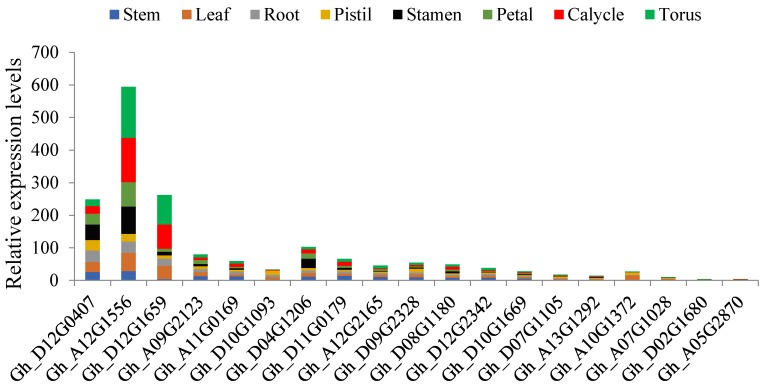
Relative expression levels of the transcriptome factors found to be targeted by ghr-miR390a. The axis *x*: the genes, *y*: the expression levels.

Gene functions are regulated by a number of biomolecules, in which miRNAs have been found to be integral in the regulation of gene activities [51]. The microRNAs are single-stranded noncoding RNAs with sizes ranging from 20 to 24 nucleotides and mainly have functions in controlling gene expression by translational inhibition and destabilization of miRNAs [52]. The miRNAs have been found to be involved in various biological processes in plants including development of organs such as stems, roots, leaves, and floral parts [53]. A growing body of evidence suggests that miRNAs play key roles in plant responses to biotic and abiotic stresses [54]. In the determination of the association of the mined genes to whether they were targeted to any of the known cotton ghr-miRNAs, a total of 69 ghr-miRNAs were found to target 183 genes out of the 271, translating to 68% of all the genes of the serine/threonine-protein kinase groups. The highest level of miRNA target was noted for ghr-miR749, found to target 21 genes each, ghr-miR390a (19 genes), ghr-miR7493 (13 genes), ghr-miR7511 (12 genes), ghr-miR2949b (seven genes), ghr-miR7484a (seven genes), and ghr-miR7512 (seven genes), while the rest targeted genes ranging from one to six (Supplementary Table S6). Deep sequencing has revealed the enormous roles played by plant miRNAs, for instance, miR169 is a large miRNA family that is widespread in the plant kingdom and has been found to have a functional role in plant development and responses to environmental cues [55]. In rice, being a hydrophytic plant, some members of the miR169 family were found to be induced by drought stress [56]. In the analysis of the miRNAs, cotton ghr-miR169a, which was found to target *Gh_A05G3152 (D6PKL2)*, *Gh_A10G2128* (ppk15), and *Gh_D04G0480 (D6PKL2),* could possibly be have a functional role in enhancing drought stress tolerance in plants. Other known miRNAs, whose functions have been described, such as miR482, with a functional role in the regulation of *NBS-LRR* defense genes during fungal pathogen infection [57]. Two types of ghr-miR482 were found to target five genes, ghr-miR482a targeted *Gh_A10G1428 (PBS1)*, *Gh_A11G1858 (SAPK1)*, *Gh_D05G0690 (UCNL)*, and *Gh_D11G2149 (SAPK2)* while ghr-miR482b targeted *Gh_D05G3179 (WAG1)*. The five sets of genes targeted could possibly be involved in defense mechanism to prevent fungal infection. Plant growth, such as primary root growth, is vital to improve plants water uptake under low soil water potential. The plant miR390, *TAS3*-derived trans-acting short-interfering RNAs (tasiRNAs), and Auxin response factors (ARFs) form an auxin-responsive regulatory network controlling lateral root growth [58]. The cotton, ghr-miR390a was found to target 19 genes with different relative abundance as per the RNA sequencing data profiling in various tissues under drought condition, the most abundant gene was *Gh_A12G1556* across all the tissues, but more importantly in the root, followed by *Gh_D12G0407* and *Gh_D04G1206* (Figure 6). The abundance levels of *Gh_A12G1556*, *Gh*_D12G0407 and *Gh_D04G1206* in the root tissues could perhaps be linked to the promotion of primary root growth, and therefore increasing the capacity of the plants to acquire more water due increased absorption area.

### 2.11. Cis Element Analysis

Gene regulatory networks refer to a collection of molecules, which regulate a set of gene expression in a specific growth stage or in response to external stimuli [59]. One of the important stages of gene expression regulation is at transcription level in which cis-acting elements and transcription factors (TFs) mediate it [60]. Cis-acting elements are strength (sequences) of DNA at the promoter region of a gene, which interact with transcription factors. TFs bound to cis-acting elements form the transcriptional initiation complex that activates RNA polymerase to start transcription process of specific genes. In the transcription process, TFs act as molecular switchers to start transcription of the extraordinary gene. TFs themselves are activated in response to external stimuli such as salinity, drought, temperature alterations, and internal stimuli such as hormones [61]. In the analysis of the cis elements, GATABOX (GATA), GT1CONSENSUS (GRWAAW), EBOXBNNAPA (CANNTG), WRKY71OS (TGAC), and MYCCONSENSUSAT (CANNTG) were the most abundant with the role of chlorophyll a/b binding protein/light regulations, GT-1 binding site in many light-regulated genes, light-responsive and tissue-specific activation, a transcriptional repressor of the gibberellin signaling pathway and dehydration-responsive respectively (Table 3). Photosynthesis is an important biochemical process to the plants, which enables the plants to synthesize their own food, any process that affects the photosynthetic process and the organelles, do effectively affect the performance of the plant and in turn reduces the quality and quantity of the yield [62]. Among the cis elements with the regulatory roles in plant stress regulation detected were MYCATERD1 (water-stress responsiveness), AGCBOXNPGLB (stress signal-response factors), ASF1MOTIFCAMV (response to abiotic and biotic stress), ACGTATERD1 (required for etiolation-induced/expression of erd1 (early responsive to dehydration), CCAATBOX1 (promoter of heat shock protein), and MYB2CONSENSUSAT (MYB recognition site/dehydration-responsive) among others; the detection of these cis elements, is a pointer to show that some important alleles from the tolerant cultivar, *G. tomentosum* used as the donor parent for the development of the BC_2_F_2_ mapping population could have been introgressed into the population, thus could help in solving the drought stress effect in cotton.

### 2.12. Gene Ontology (GO) Analysis of the Mined Genes

**Figure 7:**
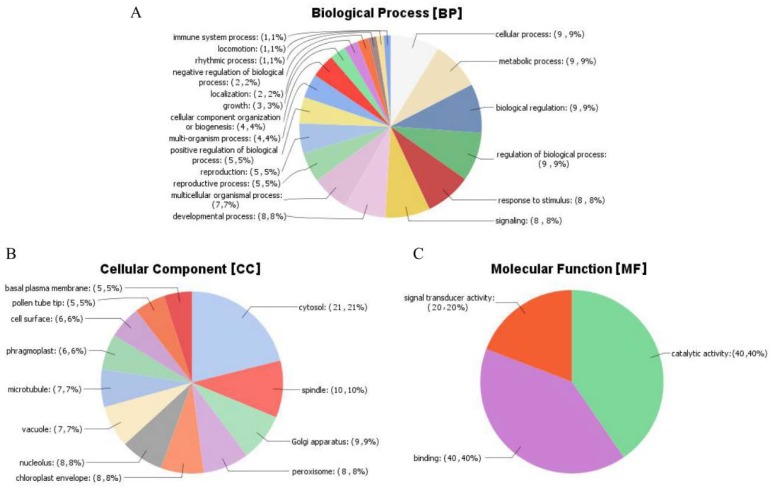
GO functional analysis of the major classes of genes of the family serine/threonine protein kinases. (**A**) Biological processes (BP), (**B**) cellular components (CC) and (**C**) molecular functions (MF).

To understand the functions of these differentially expressed probe sets, we performed gene ontology (GO) analysis using Blast2GO online tool (Available online: https://www.blast2go.com/). GO functional annotation describes the genes in to three groups, as genes with roles in the cellular components (CC), molecular functions (MF), and biological processes (BP). The analysis of the mined genes showed that all the genes were involved in all the three GO functional annotations. The highest GO functions were detected for the following genes: Gh_A11G1297 (41 GO functions), Gh_D11G1445 (39 GO functions), Gh_D11G2830 (35 GO functions), Gh_A09G0713 (34 GO functions), Gh_D06G2142, Gh_D01G1809, Gh_A09G0712, and Gh_A01G1558 with 33 GO functions each. Among all the 271 genes, Gh_D03G0962 and Gh_A03G0665 had the least number of GO functions annotation, of six each. Interestingly, in all the 6 GO functional annotations, all the three GO functional terms were detected. For the majority of the genes, their GO functional annotations ranged from 7 to 34 (Supplementary Table S7), an indication that the gene subfamily, serine/threonine protein kinases, are playing vital roles within the plant. Environmental stresses such as drought, salinity, cold among others requires the plant not only adopt morphological changes but molecular and biological processes are vital for plant survival under various abiotic stress factors. The highest GO functional annotation was detected for biological process (BP) with 236 functions, then cellular component (CC) with 60 functions, while molecular function (MF) had the least functions of 54 (Figure 7).

In -biological process (BP), various functions with direct correlation to stress factors such as GO:0009738 (abscisic acid-activated signaling pathway); GO:0009864 (induced systemic resistance, jasmonic acid mediated signaling pathway), GO:0035556 (intracellular signal transduction); GO:0032270 (positive regulation of cellular protein metabolic process), GO:1901002 (positive regulation of response to salt stress), GO:0080136 (priming of cellular response to stress), GO:2000037 (regulation of stomatal complex patterning), GO:0000226 (microtubule cytoskeleton organization), GO:0000278 (mitotic cell cycle), GO:0006468 (protein phosphorylation), GO:0007049 (cell cycle), GO:0006397 (miRNA processing), GO:0007093 (mitotic cell cycle checkpoint), GO:0006470 (protein dephosphorylation), GO:0051304 (chromosome separation), GO:0007059 (chromosome segregation), GO:0055085 (transmembrane transport), and GO:0032465 (regulation of cytokinesis). Extracellular and intracellular signals detections are vital for the plant in order to initiate precise cellular responses such as programmed cell death (PCD) in case of stress conditions. Signaling to programmed cell death result in major reorganization of cellular components. The plant cytoskeleton is known to play a major role in cellular organization [63,64]. Microtubule cytoskeleton organization function was detected for two genes, *Gh_A08G1143* and *Gh_D08G1426*. The two genes were located in the two homologous chromosomes, chrA08 of At_sub genome and chr23 (D08) of the Dt_sub genome. Given that cotton is an important crop for its natural fibers, the fibers are cells, and therefore their growth to broad spectrum of sizes and shape, is largely influenced by the microtubule cytoskeletons just like it does in the coordination of growth of other cells within the plant [65].

In relation to molecular functions (MF), various functions were detected, such as GO:0008641 (ubiquitin-like modifier activating enzyme activity), GO:0004871 (signal transducer activity), GO:0003700 (DNA binding transcription factor activity), GO:0004672 (protein kinase activity), GO:0004540 (ribonuclease activity), GO:0005524 (ATP binding), GO:0004712 (protein serine/threonine/tyrosine kinase activity), GO:0004725 (protein tyrosine phosphatase activity), GO:0005488 (binding), GO:0005515 (protein binding), GO:0004674 (protein serine/threonine kinase activity), GO:0016772 (transferase activity transferring phosphorus-containing groups), GO:0004713 (protein tyrosine kinase activity), and GO:0046873 (metal ion transmembrane transporter activity). The molecular functions detected point to various mechanisms adopted by the plants in order to increase their tolerance levels to various stress factors. The majority of the molecular functions play critical roles in the signal pathways, for instance protein tyrosine kinase activity has been found to be involved in abscisic acid signaling pathways [66]. Abscisic acid (ABA) is an important plant phytohormone, it regulates several aspects of plant development and adaptation to stress, more drought [67].

Finally, the following GO functional annotations were detected for cellular component (CC), such as GO:0005634 (nucleus), GO:0005654 (nucleoplasm), GO:0005730 (nucleolus), GO:0005737 (cytoplasm), GO:0005773 (vacuole), GO:0005776 (autophagosome), GO:0005777 (peroxisome), GO:0009507 (chloroplast), GO:0009534 (chloroplast thylakoid), GO:0012505 (endomembrane system), and GO: 0016020 (membrane), among others. The cellular membrane integrity is important for the normal functioning of the cell. Plants, being sessile, are exposed to many types of biotic and abiotic stresses. Plants sense these stimuli and transduce the signal into downstream biological responses often through the plasma membrane, which is generally the source for signaling lipids. These are usually generated by modifying enzymes like phospholipases, lipid kinases, or phosphatases. Lipid signaling molecules accumulate transiently and have a fast turnover [68], thus enhancing tolerance to various stress factors. Any damage to the membrane affects the delicate osmotic balance in the cell eventually leading to plant death. The detection of cellular component function provided fundamental information on some of the vital genes which could have been introgressed from the tolerant donor parent to the BC_2_F_2_ generations.

### 2.13. qRT-PCR Validation of the Candidate Genes

**Figure 8:**
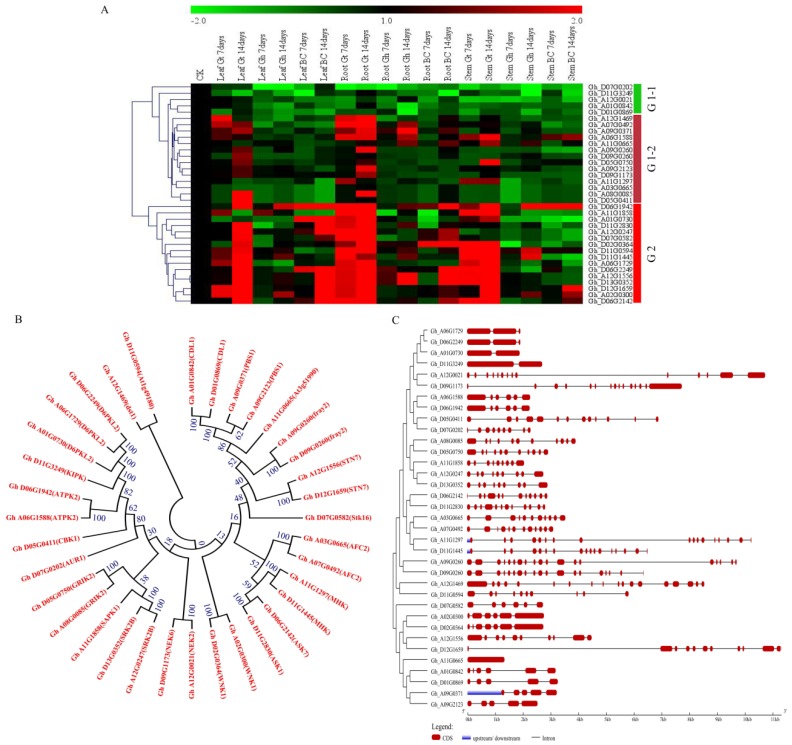
qRT-PCR validation, phylogenetic tree and structural analysis of the 35 highly up regulated genes as determined through RNA sequestration. (**A**) The heat map was visualized using Mev.exe program. (shown by log2 values) in control, and in treated samples seven and 14 days after drought treatment. BC–BC_2_F_1_ (offspring), Gt–*Gossypium tomentosum* and Gh–*Gossypium hirsutum*. Red—up regulated, Green—down regulated and black-no expression. (**B**) Phylogenetic relationship of the 35 genes, values represent percentage similarity. (**C**) Gene structure.

In response to various abiotic stresses, plants continuously need to adjust their transcriptome profile [69]. The expression levels of the various transcriptome enable us to understand the possible roles played by the various genes under stress condition. In this research work, the top ranked genes, as per the RNA sequence data, were analyzed by determining their expression levels in the root, stem, and leaves of the drought susceptible cultivar, *G. hirsutum*, drought tolerant *G. tomentosum,* and their first backcross filial generation, BC_2_F_1_. Based on the expression profile, the genes were clustered into two groups, Group 1 (19 genes) were mainly partially up regulated in the various tissues of *G. tomentosum*, but all were down regulated in both *G. hirsutum* and BC_2_F_1_ generation. The group 2 (16 genes) were highly up regulated in all the tissues of the drought tolerant genotype, *G. tomentosum*, but were partially up regulated in the tissues of drought susceptible parent, *G. hirsutum*, but relatively up regulated in BC_2_F_1_ compared to *G. hirsutum*. (Figure 8A). We further analyzed the structure and phylogenetic tree relationship of the 35 genes, in order to understand the expression behaviors of the paralogous gene pairs; generally genes of the same functional domain were cladded together, for instance *Gh_A01G0842* and its homolog pair in Dt_sub genome *Gh_D01G0869*, were cladded together with 100% similarity (Figure 8B). Though it is important to note that not all the paired genes were homologous to each other, for instance *Gh_A03G0665* and *Gh_A07G0492*, but were all members of Serine/threonine-protein kinase *AFC2*. All the genes were found to be interrupted by introns, except *Gh_A11G0665* (Figure 8C). Introns have been found to be playing an important role in gene expression. Significantly higher level of gene expression has been observed in intron-containing transgenes than from otherwise identical intronless constructs [70]. In general, more genes were found to up regulated in various tissues of the tolerant genotype, *G. tomentosum* as opposed to the expression levels in both *G. hirsutum* and BC_2_F_1_, a similar observation has been reported in which tolerant genotypes have shown the ability to induct more stress related genes compared to the less tolerant genotypes in various crops such as barley [71]. 

## 3. Discussion

The use of backcross population is vital as compared to the real inbred lines of the F_2_ populations. In this research work, we successfully developed a backcross population between an elite upland cotton, *G. hirsutum* and a wild type *G. tomentosum.* We developed the backcross in order to solve the problem of hybrid sterility and the difficulty of using wild genotypes for trait mapping and genotype improvements [72]. A backcross population was developed through a two tire-backcross of a wild donor parent, *G. tomentosum* crossed to an adapted recurrent parent, *G. hirsutum*. The backcross generation developed had a much smaller portion of the wild genome present in each line; the effects of agronomically-unadapted alleles were reduced, allowing estimates of the value of wild alleles in the context of cultivated germplasm. Similar approaches have been used successfully in various crops such as barley [73], wheat, and maize [72] among other plants.

In the past decade, several successive molecular maps of cotton have been constructed using diverse DNA molecular markers and mapping populations. The major mapping populations used were the inter- and intra-specific populations mainly of inbred lines of the F_2_ generations, such as F_2_ generations developed between *G. hirsutum* and *G. mustelinum* [74], *G. hirsutum* and the Hawaiian endemic, *G. tomentosum* [75], among many others. The significant distinguishing feature of the linkage map constructed in this work, compared with previous maps, is that it was developed from a more recent and precise mapping technique known as genotyping by sequence (GBS). The mapped developed had a map size of 4191.259 cM covering all the chromosomes with an average marker distance of 0.3849 cM. The percentage proportions of marker distance of less than 1cM compared to the total of markers generated was 82%, which translated to 8921 out of the 10,888 markers used. And therefore, the map developed qualifies as a fine map, which would meet the needs for marker assisted selection (MAS). In addition, the mapping coverage in relation to the reference genome, *G. hirsutum* across the 26 linkage groups ranged from 97.3 to 100%. The highest coverage per chromosome was observed in At_chr07, At_chr08, Dt_chr03, and Dt_chr07, while the lowest coverage was in Dt_chr05. The overall genome coverage was 99.6%, with 99.4% and 99.7% coverage in the At and Dt_sub genomes respectively. The map generated was not only suitable for MAS but an important tool in mining various genes with significant roles both in growth and development.

The ever-changing environmental conditions have posed a great challenge not only to cotton production but to other crops too. The loss caused by drought and salt stress combined currently stands at 60% of the total economic loss in the agricultural sector [76]. Cotton production has continued to decline as a result of abiotic stress factors, the problem is much more compounded due to narrow genetic base of the cultivated cotton due to intensive section and interbreeding over a period of time [77]. The application of cotton wild progenitors will help to solve the bottleneck of narrow genetic base and in turn change the response dynamics of cultivated cotton to various environmental stress factors. Due to excellent reference genome coverage of the map developed, we carried out gene mining per linkage group, and obtained a total of 32,892 genes, with the lowest number of genes mined in At_chr04 (1210 genes), while the highest number of genes mined were obtained in Dt_chr05 (3600 genes). All the genes mined were found to be distributed in 6141 domains. Due to the huge number of genes domains obtained in this research, we further analysed the domains in order to determine the frequency of each domain per the linkages, the domain with the highest frequency was termed as the dominant domain. The dominant domain was the Pkinase (PF00069) with a total of 1069 genes. In the Pkinase domain (PF00069), we identified 44 different sub domains. The sub domain with the highest number of genes was found to serine/threonine-protein kinase (271 genes), in which of interest were those involved in abiotic stress tolerance. The various serine/threonine-protein kinases types detected were; Serine/threonine-protein kinase 16/38 (seven genes), *AFC1* (seven genes), *AGC1-7* (two genes), *At3g07070* (six genes), *ATG1a* (five genes), *AtPK2/AtPK19* (eight genes), *Aurora-1/3* (four genes), *BLUS1* (ten genes), *CBK1* (six genes), *CDL1* (ten genes), *D6PKL2* (16 genes), *dst1* (four genes), *fray2* (17 genes), *GRIK2* (four genes), *KIPK* (11 genes), *mph1* (three genes), *Nek2/5/6/7* (20 genes), *OSR1* (one gene), *PBS1* (23 genes), *PEPKR2* (four genes), *ppk15* (four genes), *Prpf4b* (one gene), *RUK* (two genes), *SAPK1/2/3/7/8* (8 genes), *spk-1* (one gene), *SRK2B/E/G/H* (12 genes), *SRK2I* (three genes), *SRPK* (three genes), *STN7/8* (four genes), *svkA* (four genes), *TIO* (one gene), *Tnni3k* (one gene), *TOUSLED* (three genes), *trc* (six genes), *UCNL* (five genes), *WAG1* (four genes), *WNK1/8* (eight genes), *IRE1B* (two genes), *At1g28390/ At5g23170* (four genes), *CCR1/4* (four genes), and *ASK1/7/8/10* (14 genes). Plants respond to environmental stresses such as drought, salinity, and cold by activation of complex intracellular signaling cascades that regulate physiological and biochemical acclimation. In multicellular organisms, protein kinases are key elements involved in signal transduction responsive to metabolism, biotic, and abiotic stresses [78]. Results obtained were in agreement to previous publications, in which sucrose non-fermenting 1 (SNF1)-type serine-threonine protein kinase *SAPK4* has been found to regulate stress-responsive gene expression in rice [79]. In addition, the *PBS1* genes have been found to be involved in defense mechanisms against diseases in plants [80]. The use of the wild cotton progenitors is significant in solving various abiotic and biotic stress pandemics in plants; Kirungu et al. [81] applied simple sequence repeat (SSR) markers in mapping an F_2_ population developed from two wild cotton of the D genome, they were able to find vital genes such as the *NAC* genes, which have been previously shown to be involved in enhancing various stress factors in plants. And therefore, the detection of the myriad genes of the serine/threonine protein kinases, of being the most abundant domain, could offer reprieve in solving the problem of drought stress in cotton breeding strategies.

In carrying out deep analysis of the genes of the dominant sub domains of the Pkinases, we characterized the genes through miRNA target predictions, cis element analysis, GO functional annotations, and RNA sequence analysis. The genes were found to be targeted by various miRNAs, such as ghr-miR156c (five genes), ghr-miR164 (6 genes), ghr-miR390a (19 genes), ghr-miR7491 (21 genes), and ghr-miR7511 (12 genes) among others. The microRNAs, such as miR164, have been found to be playing an important role in drought stress conditions, and have been closely associated to the *LEA* genes, which do accumulate in high levels in seeds under dehydrated conditions [82]. Similarly, various cotton miRNAs such as miR164, miR172, miR396, miR1520, miR6158, ghr-n24, ghr-n56, and ghr-n59, among which some were detected in this research, have been found to be associated to some of the top ranked genes related to drought and salinity, such as *NAC*, *MYB*, and *MAPK* [83]. The cotton miRNA such as ghr-miR164 targeted the following genes, *Gh_A01G1666* (*NEK7*), *Gh_A09G1167* (*NEK6*), *Gh_D01G1916* (*NEK7*), *Gh_D08G0686* (*NEK2*), *Gh_D09G1173* (*NEK6*), and *Gh_D10G1380* (*BLUS1*), while ghr-miR396a targeted four genes which were *Gh_A07G1048* (*TOUSLED*), *Gh_D05G0400* (*Tnni3k*), *Gh_D05G0835* (*KIPK*) and *Gh_A11G2021* (*ppk15*). The NIMA-related kinases (NEKs) genes have been identified in eukaryotic organisms, including human beings, the *NEK* genes are classified into 11 groups, *NEK1* to *NEK11*. A number of the *NEK* gene family members have been found to play significant roles in cell cycle control. In particular, *NEK2*, *NEK6*, *NEK7*, and *NEK9* contribute to the establishment of the microtubule-based mitotic spindle, whereas *NEK1*, *NEK10*, and *NEK11* have been implicated in the DNA damage response [84]. Roles for *NEKs* in other aspects of mitotic processes, such as nuclear envelope breakdown, spindle assembly checkpoint signaling, chromatin condensation, and cytokinesis have also been pointed out. Environmental stresses such as drought and salt stress, leads to over production of oxidants such as H_2_O_2_, which has degrading effect on the DNA, the detection of *NEK* associated genes, could be involved in the DNA repair.

Gene ontology (GO), provides critical information on various functions, the genes could be involved in within the cell. It basically classifies the genes into three functional groups, biological process (BP), molecular function (MF), and cellular component (CC). Among the serine/threonine genes, various functions were detected for biological and molecular process, whereas only a single function was detected for the cellular component, which was the membrane. The various functions detected were highly correlated to various biotic and abiotic stress tolerances in plants. The results obtained for GO annotations were further supported by the results obtained for the cis elements analysis. The cis elements regulate the gene functions and there are known cis elements, such as the MYBs, LTRE, and ABRE, which play significant roles in enhancing drought tolerance in plants [85]. The detection of these cis elements among others clearly shows the important roles played by the mined genes, in enhancing tolerance to various biotic and abiotic stresses.

RNA sequence data revealed differential expression pattern among the 271 genes of the serine/threonine protein kinases. The majority of the genes were found to be up regulated under salt and drought stress conditions, more so group 1 and 3. The highly up regulated gene among the entire 271 genes was *Gh_A12G1556*, *STN7* (serine/threonine-protein kinases STN7, chloroplastic). The *STN7* kinase catalyzes the phosphorylation of the globally most common membrane proteins in plant chloroplasts [86]. Recent discoveries have shown that different biotic and abiotic stresses have detrimental effects on plant growth and development, affecting chloroplast functions. Photosynthesis is significantly impaired in response to abiotic stresses, such as drought, salinity, cold, high light stress, and heat. Photosynthesis also responds rapidly to pathogen infection [87]. Increased levels of hydrogen peroxide (H_2_O_2_) and reactive oxygen (ROS) due stress effects destroys the photosynthetic apparatus, therefore the primary response is the regulation of ROS resulting from photosynthesis [88]. Increased levels of reactive oxygen causes massive damage to the cell, affecting the cell membrane stability and in extreme conditions leads to cell death [89]. Cell membrane stability (CMS) under drought stress condition is critical for plants to maintain its various physiochemical activities and thus a physiological trait used for determining drought stress tolerance in plants [90]. Plants have internal repair mechanisms whenever the chloroplast is damaged. The chloroplasts have repair mechanism for the photosynthetic core proteins, most importantly the D1 protein of the photosystem II (PSII) [91]. The role of *STN7* in enhancing stress tolerance has been investigated in the model plant, *Arabidopsis thaliana*, in which STN7 was found to be regulated by oxidative and or salt stress [92]. Similarly, water deficit has been found to accelerate the phosphorylation process of the photosystem II (PSII) core and D1 protein synthesis in *Pisum sativum* [93]. Moreover, phosphorylations of the thylakoid proteins and related non-photochemical quenching (NPQ) have been proposed to be an adaptive mechanism of chloroplasts to abiotic stress [94].

## 4. Materials and Methods

### 4.1. Development of Plant Materials

Backcross Inbred Lines (BILs) was developed using *G. hirsutum*-CRI-12 (G09091801-2) and *G. tomentosum*-AD3-00 (P0601211) as the recurrent and donor parents respectively. The maternal parental line, accession number CRI-12 forms over 90% of cotton being currently cultivated in China, but its production is being limited due to its low tolerance to drought and salt stresses. It is mostly preferred due its high production and relatively superior fiber quality. *G. hirsutum* accession number CRI-12 was developed by our research institute, cotton research institute (CRI), Chinese Academy of Agricultural Sciences, thus the code CRI. The accession AD3-00 (P0601211) of *G. tomentosum* was used as the male donor parent, it was collected from its natural habitat from the dry and rocky coastal areas of the Hawaiian Islands [16]. *G. hirsutum* was crossed with *G. tomentosum*, and F_1_ was then crossed with the recurrent parent, *G. hirsutum* twice. BC_2_F_1_ was then selfed to obtain BC_2_F_2_; over 400 lines were eventually developed. Two hundred backcross progenies were used in this study; the 200 lines were selected due to insufficient amount of seeds. The lines were developed in Sanya Island, Hainan province, China. The region has a tropic monsoon climate with mean annual temperature range of 22–27 °C with annual precipitation between 1500 and 2600 mm. 

### 4.2. Sample Collection, Extraction, Quantification and Quality Determination of DNA

Tender leaves of the 200 backcross population (BC_2_F_2_) and the two parental lines were collected and frozen in liquid nitrogen, before being stored under –80 °C in preparation for DNA extraction. The DNA extraction kit, obtained from TaKaRa MiniBEST Plant Genomic (TAKARA BIO Inc., Beijing, China) was used for DNA extraction. The kit contained 70 µL 50× DTT Buffer, 500 µL, 28 mL buffer HS1, 1.8 mL Buffer KAC, 28 mL Buffer GB, and 28 mL Buffer WA, 24 mL buffer WB and elution buffer of 14 mL. We carried out pre-preparation by adding 56 mL of absolute ethanol (100%) to buffer WB, mixed before use, 500 µL of HS1 with 10 µL of 50× DTT buffer was prepared for each sample in 1.5 mL tubes. Each sample of approximately 100 mg of leaf tissues was vortexed in liquid nitrogen in to fine powder, then immediately added to the marked tubes containing a mixture of HS1 and 50× DTT Buffers, 10 µL RNaseA was immediately added, mixed and the tubes incubated in water bath at 56 °C for 10 min. In each sample tube, 62.5 µL of buffer KAC was mixed with 12.5% of Buffer HS1 by volume, the mixture was then vortexed for one minute, then put in ice for 5 min, after which, centrifuged at 12,000 rpm for 5 min. The supernatant was then transferred into a new tube, and an equal amount of buffer GB was added, and then mixed. The mixture was then transferred to spin column and centrifuged at 12,000 rpm for 1 min. The flow through was discarded. On to the spin column, 500 µL of Buffer WA was added, and centrifuged at 12,000 rpm for one minute. The flow through was then discarded. On the same spin column, 700 µL of buffer WB was added, and then centrifuged at 12,000 rpm for 1 min, the procedure was repeated twice. The spin column was then transferred to collecting tube, and centrifuged for 2 min. The spin column was then transferred into a 1.5 mL tube, 50 µL of preheated elution buffer was added, and then left for 5 min before centrifuging at 12,000 rpm 2 min, and this was to dissolve the DNA. We determined the degradation and contamination of DNA by running the DNA samples through 1% agarose gels. The purity of DNA was determined by using the Nano Photometer^®^ spectrophotometer (IMPLEN, Westlake Village, CA, USA). The ratio of absorbance at 260 nm and 280 nm was used to assess the purity of DNA. The DNA samples with the ratio of ~1.8 were then qualified as pure [95]. The concentration of DNA was done using Qubit^®^ DNA Assay Kit in Qubit^®^ 2.0 Fluorimeter (Life Technologies, Camarillo, CA, USA). The Qubit^®^ dsDNA HS (High Sensitivity) Assay Kits make DNA quantitation easy and accurate, the kit was used as per the procedure [96].

### 4.3. The GBS Library Preparation, Sequencing, and SNP Genotyping

The Illumina platform offers increased sequencing depth and a robust paired-end sequencing technology that recovers DNA sequence from the both ends of a single DNA template [97]. After the Illumina HiSeqTM 2500, California, United Sates of America, sequencing data (raw data) is down, quality control of the down data was performed, and the low-quality data was filtered to obtain high quality data (Clean Data). The quality filtering methods for Illumina reads generally rely upon machine-reported *Q*-scores and empirically defined thresholds to eliminate noise [98]. Varying the stringency of these thresholds changes the sensitivity and specificity of the outcome, without guaranteeing an accurate base call using sequencing data with burrows wheeler aligner (BWA) software [99]. Comparison of the sequence to the published reference genome of *G. hirsutum* was done in order to obtain the position of the sequences in bam file format by use of GATK software [100]. Correction of bam files, detection of SNP, and small Indel were carried out using the vcfutils tool of the Samtools software [99] to obtain high-quality mutation sites. The principle of genetics requires that the markers are coded according to the parental alleles, and the markers were filtered to obtain high-quality molecular markers.

The sequencing through GBS was carried out, as outlined by Elshire et al. [101], by incorporating three PCR plates of 96 wells across the 200 barcodes for library preparation and sequencing. For SNP determination, the raw sequence data for the mapping population of 200 BC_2_F_2_ and two parental lines were analyzed through GATK software GBS pipeline [102] by integrating *G. hirsutum* sequence as the reference genome [103]. The reference genome was downloaded from Cotton research institute website (Available online: http://mascotton.njau.edu.cn/info/1054/1118.htm). For alignment, the Burrows–Wheeler Aligner (BWA) mem [104] with default parameters was employed. The output consisted of variant call format (VCF) file version 4.1 [105] including SNPs present in at least 40% of the progeny and with a minor allele frequency (MAF) 0.1. Subsequently, the VCF was filtered using vcf tools version. 1.12 [105] and GATK software [100]. Ninety three thousand three hundred and eighty four (93,384) raw SNPs were identified in 200 BC_2_F_2_ population by using GATK software [100]; a custom filtering process was applied for alignment. The filtering was based on maintaining sites with read depth range of 6% and 75% completeness by site across progeny and by progeny across sites. Results were obtained in hapmap file format. Finally, through the use of a custom perl script marker heterozygous in the BC_2_F_1_ generations, with a co-dominant segregation ratio of 1:2:1 among the BC_2_F_2_ population, a chi-squared (χ2) goodness-of-fit test at α ≤ 0.01 was determined. The whole data obtained was then reformatted and transferred to JoinMap^®^ 4.1 for linkage group determination. Twenty six (26) linkage groups were generated, being the parental lines were tetraploid cotton, with 26 chromosomes.

### 4.4. Linkage Map Construction 

Markers were arranged based on their LOD scores, pairwise recombination fractions and linkage group length [106]. Linkage analysis was done with JoinMap 4.0 [107] with a recombination frequency set at 0.40 with LOD score of 2.5 for the BIL population, this was a somewhat more stringent threshold than that the value used for the relatively smaller genomes, though appropriate for the approximately 4500 cM genome of cotton. The Kosambi mapping function was used to convert the recombination frequencies to map distances [108]. Linkages at distances of >35 Kosambi cM, were considered non-significant. 

### 4.5. Gene Mining and Functional Characterization 

20 kb up and down stream regions were applied for mining of genes, similar method has been used in mining of genes in diploid cotton [81]. The mined genes were analysed and their various functional annotation determined. We carried out functional annotation through Blast2GO pro-software version 4.1.1 (Available online: https://www.blast2go.com), phylogentic tree and gene structures analysis of the key genes by the use of gene structure displayer server (Available online: http://gsds.cbi.pku.edu.cn/).

### 4.6. miRNA Target and Promoter Analysis 

MicroRNAs (miRNAs) are small RNA molecules, which function as regulators of gene expression in a range of developmental and signaling pathways in plants and animals. A number of studies have shown that abiotic stresses trigger aberrant expression of many miRNAs, thus indicating that miRNAs may be a new target for genetically improving plant tolerance to certain stresses [54]. 

In order to determine if the mined genes were targeted by any known miRNAs, we carried out the prediction of miRNAs which could be targeting the mined genes. The miRNA sequences were downloaded from miRBase (Available online: http://www.mirbase.org), the plant miRNA database (Available online: http://bioin formatics.cau.edu.cn/PMRD/), and EST database (Available online: http://www.ncbi.nlm.nih.gov/nucest). The genes targeted by miRNAs were predicted by searching 5′ and 3′ untranslated regions (UTRs) and the coding sequence (CDS) of all the mined genes for complementary sequences of the cotton miRNAs using the psRNATarget server with default parameters (Available online: http://plantgrn.noble.org/psRNATarget/?Function=3). 

In addition, we also carried out cis promoter analysis, the promoter sequences (2 kb upstream of the translation start site) of all the mined genes, which were obtained from the cotton genome project (Available online: http://cgp.genomics.org.cn/page/species/index.jsp). Transcriptional response elements of the mined genes promoters were predicted using an online tool the PLACE database (Available online: http://www.dna.affrc.go.jp/PLACE/signals can.html).

### 4.7. RNA Sequence Analysis of the Mined Genes and qRT-PCR Validation of Key Genes under Drought Stress Condition

The mined genes were further analyzed by extracting their RNA sequences from cotton genome database (Available online: http://mascotton.njau.edu.cn) in reference to salt and drought stresses expression profiles at varying time intervals. The reads per kilobase of exon per million reads mapped (FPKM) data was then transformed into log10 and a heatmap constructed. The top 35 highly expressed key genes were later used for qRT-PCR validation under drought stress condition. Primer premier 5 (Available online: http://www.premierbiosoft.com/primerdesign/) was used to design the genes specific primers with melting temperatures of 55–60 °C, primer lengths of 18–25 bp, and amplicon lengths of 101–221 bp (Supplementary Table S8). *Ghactin* gene forward sequence “ATCCTCCGTCTTGACCTTG” and reverse sequence “TGTCCGTCAGGCAACTCAT” were used as the reference gene for the qRT-PCR analysis. The tissues used were mainly obtained from the two parental lines, drought resistant male donor genotype, *G. tomentosum*, the drought sensitive recurrent female genotype, *G. hirsutum* and their first backcross filial generation BC_2_F_1_ progenies. The three cotton genotypes were grown in a greenhouse, and at three true leaves stage, water was totally withdrawn, the moisture level was monitored daily by use of soil moisture sensor, Em50 DECAGON, Pullman, WA, USA. When soil is used as opposed to hydroponic set up for carrying out drought stress tolerance screening in plants, longer stress exposure is always suitable for obtaining samples for carrying out gene expression analysis [82]. The condition in the greenhouse was set with temperature at 23 ± 1 °C and a 14 h light/10 h dark photoperiod. The soil water potential was maintained at −20 kPa under the controlled plants, while drought treated plants, water was totally withdrawn. Soil is known to be well watered when the soil water potential is above −30 kPa [109]. Roots, leaves, and stem tissues were collected at 0, 7 and 14 days of post drought stress treatments for RNA extraction. The RNA extraction kit, EASYspin plus plant RNA kit, by Aid Lab., Beijing, China (Available online: www.aidlab.cn), was employed in the extraction of RNA from the samples. The concentration and quality of each extracted RNA samples was determined by using a NanoDrop 2000 spectrophotometer (Thermo Fisher, Waltham, MA, USA) and gel electrophoresis. The RNA samples which met the criterion 260/280 ratio of 1.8–2.1, 260/230 ratio ≥ 2.0, were used for further analyses. The first strand cDNA synthesis was done with TranScriptAll-in-One First-Strand cDNA Synthesis SuperMix for qRT-PCR (Roche Diagnostics GmbH, Mannheim, Germany), it was used as per the manufacturer’s instructions. The Fast Start Universal SYBR green Master (Rox) (Roche, Mannheim, Germany) was used to carry out qRT-PCR analysis in accordance with the manufacturer’s instructions. The qRT-PCR reactions samples were prepared in a total volume of 20 μL, containing 10 μL of SYBR green master mix, 2 μL of cDNA template, 6 μL of ddH_2_O, and 2 μL of each primer.

## 5. Conclusions

The development and mapping of the backcross population developed from the two tetraploid cottons of diverse origin provided vital information on how the wild cotton progenitors can be exploited to solve the impasse of low and poor yield in cotton due to effects of abiotic and biotic stress factor. The broadening of genetic diversity of the elite cotton cultivars is paramount, if we have to meet the ever-growing demand for food and clothing. Advancement in the spinning industries too has worsened the situation, as very high-quality fibres are required. *Gossypium hirsutum*, an upland cotton, is the most preferred tetraploid cotton, due to its high yielding and comparatively good fibre qualities, but its production is threatened by its narrow genetic base, as a result of intensive selection and inbreeding. The development of the BC_2_F_2_ population between *G. hirsutum* and *G. tomentosum* provides an excellent germplasm, which can be utilised in solving the problem of narrow genetic base. The map developed in this study through GBS markers was the very first fine genetic map developed between the two germplasm, the map covered a total map size of 4,191.259 cM, with 10,888 SNPs being distributed across the 26 linkage groups, generating an average marker distance of 0.3849 cM. The total SNPs generated in this study had Q30 range of 93.75% to 95.84%, an indication that the sequencing process was of high threshold, with error minimised to zero level. The map generated enabled us to mine a total of 32,892 genes, being distributed across 6141 gene domains. In the analysis of the various gene domains, Pkinase (PF00069) was found to be the most dominant group with a total of 1069 genes. The dominant domain was further analysed, and the serine/threonine-protein kinases were found to the majority genes, with a total of 271 genes, belonging to different sub classes such as the *NEK* and *STN* genes among others. In the analysis of the RNA sequence data, the genes were found to exhibit differential expression under salt and drought stress conditions, in which 16 genes were found to be the candidate genes with greater role under drought stress condition as revealed by both RNA sequence and qRT-PCR validation results. The candidate genes are *Gh_A02G0364* (Enoyl-CoA delta isomerase 3), *Gh_A06G1729* (Serine/threonine-protein kinase *D6PKL2*), *Gh_D13G0352* (Serine/threonine-protein kinase *SRK2B*), *Gh_A12G0247* (Serine/threonine-protein kinase SRK2B), *Gh_D06G2142* (Shaggy-related protein kinase *eta*), *Gh_A02G0300* (Serine/threonine-protein kinase WNK1), *Gh_A12G1659* (Kelch domain-containing protein 3). *Gh_A12G1556* (Serine/threonine-protein kinase *STN7*, chloroplastic *Klhdc3*), *Gh_D06G2249* (Serine/threonine-protein kinase *D6PKL2*), *Gh_D11G1445* (Serine/threonine-protein kinase *MHK*), *Gh_D11G0594* (Serine/threonine-protein kinase ATG1t), *Gh_D07G0582* (Serine/threonine-protein kinase Stk16), *Gh_D11G2830* (Shaggy-related protein kinase alpha *ASK1*). *Gh_A01G0730* (Serine/threonine-protein kinase *D6PKL2*), *Gh_A11G1858* (Serine/threonine-protein kinase *SAPK1*), and *Gh_D06G1942* (Serine/threonine-protein kinase *AtPK2/AtPK19*) were found to be highly up regulated under drought stress condition, and thus can be exploited further in the development of robust cotton genotypes with improved performance under water deficit condition.

## Figures and Tables

**Table 1:** Genetic map characteristics.

Linkages	Markers (P. map)	Markers (G. map)	G. map Size (cM)	Largest Gap (cM)	Smallest Gap (cM)	<1 cM	1–5 cM	5–10 cM	10–20 cM	>20 cM	Ratio
LG1_chrA01	2306	772	185.4601	19.828	0.1706	767	2	0	1	0	0.99
LG2_chrA02	2368	707	133.6326	2.1472	0.1527	705	1	0	0	0	1
LG3_chrA03	221	70	183.6242	60.9522	0.7464	44	19	4	0	2	0.63
LG4_chrA04	2057	803	184.7308	1.3336	0.1704	800	2	0	0	0	1
LG5_chrA05	778	285	180.1844	10.5478	0.3584	247	35	0	2	0	0.87
LG6_chrA06	193	88	135.7902	15.3278	0.4373	48	36	2	1	0	0.55
LG7_chrA07	1572	474	146.5589	4.5868	0.2307	467	6	0	0	0	0.99
LG8_chrA08	1664	542	164.4535	13.5821	0.1979	536	4	0	1	0	0.99
LG9_chrA09	1054	230	176.1339	3.8988	0.6234	190	39	0	0	0	0.83
LG10_chrA10	1139	380	184.0143	2.227	0.3827	361	18	0	0	0	0.95
LG11_chrA11	1650	480	182.8387	20.4651	0.2612	474	4	0	0	1	0.99
LG12_chrA12	353	113	131.5913	8.1154	0.6518	71	40	1	0	0	0.63
LG13_chrA13	1285	354	159.996	9.8116	0.3271	350	1	2	0	0	0.99
**At_sub Total**	**16,640**	**6318**	**2149.009**	**60.9522**	**0.1527**	**5060**	**207**	**9**	**5**	**3**	**0.8**
LG14_chrD01	198	45	151.7771	145.8727	0.104742	43	0	0	0	1	0.96
LG15_chrD02	237	85	129.8661	32.0047	0.75454	60	21	2	0	1	0.71
LG16_chrD03	161	79	131.4133	17.51484	0.861499	44	31	2	1	0	0.56
LG17_chrD04	365	129	151.0376	8.6138	0.841359	98	27	3	0	0	0.76
LG18_chrD05	109	70	173.9945	78.08868	0.570595	33	34	0	1	0	0.47
LG19_chrD06	2419	947	158.7206	0.81108	0.12249	499	0	0	0	0	0.53
LG20_chrD07	393	164	159.8483	5.9477	0.617165	96	66	1	0	0	0.59
LG21_chrD08	1918	824	178.4512	0.85601	0.164057	823	0	0	0	1	1
LG22_chrD09	852	293	149.2598	101.1146	0.131356	291	0	0	0	1	0.99
LG23_chrD10	1854	705	137.6682	2.3434	0.136199	700	4	0	0	0	0.99
LG24_chrD11	691	218	186.8999	2.0093	0.714943	173	44	0	0	0	0.79
LG25_chrD12	1593	558	133.5851	1.5444	0.178899	555	2	0	0	0	0.99
LG26_chrD13	1230	455	199.7287	17.6884	0.300543	446	7	0	1	0	0.98
**Dt_sub total**	**12,020**	**4572**	**2042.25**	**145.8727**	**0.104742**	**3861**	**236**	**8**	**3**	**4**	**0.84**
**At/Dt Totals**	**28,660**	**10,888**	**4191.259**	**145.8727**	**0.104742**	**8921**	**443**	**17**	**8**	**7**	**0.82**

P. map: physical map; G. map: genetic map; Ratio: number of markers less than (<) 1 cM divided by total number of markers within the chromosome.

**Table 2:** Gene mining within the SNP markers for all the linkage groups based on their physical position in mega base pairs (Mb).

Chr.	Number of Markers	Cover Length (Mb)	Chr. Length (Mb)	Coverage (%)	Density (marker/Mb)	Genes in Reference Genome/chr	Mined Genes/Chro.	% of Mined Genes/Chro.	Number of Domains
LG1_chrA01	2306	99.85026	99.8847	100	23.1	2162	1946	90	800
LG2_chrA02	2368	83.27669	83.447906	99.8	28.4	1824	1692	92.8	673
LG3_chrA03	221	100.2122	100.26305	99.9	2.21	2187	1860	85	771
LG4_chrA04	2057	62.76193	62.913772	99.8	32.8	1491	1210	81.2	102
LG5_chrA05	778	91.9822	92.047023	99.9	8.46	4026	2900	72	997
LG6_chrA06	193	102.9532	103.17044	99.8	1.87	2119	1788	84.4	757
LG7_chrA07	1572	78.15538	78.251018	99.9	20.1	2369	2078	87.7	831
LG8_chrA08	1664	103.6089	103.62634	100	16.1	2571	2233	86.9	869
LG9_chrA09	1054	74.86152	74.999931	99.8	14.1	2532	2168	85.6	873
LG10_chrA10	1139	100.6926	100.8666	99.8	11.3	2357	2176	92.3	897
LG11_chrA11	1650	93.30573	93.316192	100	17.7	3305	2947	89.2	993
LG12_chrA12	353	87.39659	87.484866	99.9	4.04	2733	2498	91.4	938
LG13_chrA13	1285	78.02031	79.961121	97.6	16.5	2356	1829	77.6	777
**At-sub Total**	**16,640**	**1157.077**	**1160.23**	**99.7**	**14.4**	**32,032**	**17,106**	**85.9**	**3007**
LG14_chrD01	198	60.91516	61.456009	99.1	3.25	2383	2063	86.6	800
LG15_chrD02	237	67.22226	67.284553	99.9	3.53	2448	2356	96.2	845
LG16_chrD03	161	46.6713	46.690656	100	3.45	1860	1632	87.7	681
LG17_chrD04	365	51.27027	51.45413	99.6	7.12	2040	1765	86.5	121
LG18_chrD05	109	60.2436	61.933047	97.3	1.81	3942	3610	91.6	1128
LG19_chrD06	2419	64.09113	64.294643	99.7	37.7	2394	2231	93.2	844
LG20_chrD07	393	55.29315	55.312611	100	7.11	2503	2191	87.5	854
LG21_chrD08	1918	65.83381	65.894135	99.9	29.1	2765	2621	94.8	951
LG22_chrD09	852	50.90761	50.995436	99.8	16.7	2493	2401	96.3	921
LG23_chrD10	1854	62.78548	63.374666	99.1	29.5	2646	2378	89.9	888
LG24_chrD11	691	65.54361	66.087774	99.2	10.5	3539	3250	91.8	1038
LG25_chrD12	1593	58.94278	59.109837	99.7	27	2838	2581	90.9	931
LG26_chrD13	1230	59.8794	60.534298	98.9	20.5	2551	2367	92.8	922
**Dt-sub total**	**12,020**	**769.6**	**774.4**	**99.4**	**15.6**	**34,402**	**15,786**	**91.2**	**3134**
**At/Dt Totals**	**28,660**	**1926.7**	**1934.7**	**99.6**	**14.9**	**66,434**	**32,892**	**88.5**	**6141**

Chr: chromosome; Mb: mega base pair; Dt: Dt_sub genome; At: At_sub genome.

**Table 3:** Cis elements detected for the mined genes of the serine threonine domain.

Factor or Site Name	Signal Sequence	Function	Number of Genes
MYCATERD1	CATGTG	water-stress responsiveness	123
AGCBOXNPGLB	AGCCGCC	stress signal-response factors	32
ASF1MOTIFCAMV	TGACG	Response to abiotic and biotic stress	123
AUXREPSIAA4	KGTCCCAT	Response to abiotic and biotic stress	16
ATHB1ATCONSENSUS	CAATWATTG	Response to abiotic and biotic stress	10
ATHB5ATCORE	CAATNATTG	Response to abiotic and biotic stress	10
AUXRETGA1GMGH3	TGACGTAA	Response to abiotic and biotic stress	3
ACGTATERD1	ACGT	Early responsive to dehydration	214
CCAATBOX1	CCAAT	Promoter of heat shock protein	251
MYBST1	GGATA	Plant MYB binding site	233
MYBPZM	CCWACC	Plant MYB binding site	176
MYBPLANT	MACCWAMC	Plant MYB binding site	79
GCCCORE	GCCGCC	pathogen-responsive genes	65
CPBCSPOR	TATTAG	NADPH-protochlorophyllide reductase	127
CCA1ATLHCB1	AAMAATCT	myb-related transcription factor	24
MYB2CONSENSUSAT	YAACKG	MYB recognition site/dehydration-responsive	236
MYBCOREATCYCB1	AACGG	Myb core/M-phase-specific expression	207
MYBGAHV	TAACAAA	Myb binding site	79
LTRECOREATCOR15	CCGAC	Low-temperature-responsive element	171
LTRE1HVBLT49	CCGAAA	Low-temperature-responsive element	159
LTREATLTI78	ACCGACA	Low-temperature-responsive element	30
INRNTPSADB	YTCANTYY	Light-responsive transcription	229
EBOXBNNAPA	CANNTG	Light-responsive and tissue-specific activation	260
GT1CORE	GGTTAA	Light-dependent transcriptional activation	91
IBOXCORE	GATAA	Light regulation	248
IBOX	GATAAG	Light regulation	158
IBOXCORENT	GATAAGR	Light regulation	135
GLMHVCHORD	RTGASTCAT	Involved in the nitrogen response	10
GMHDLGMVSPB	CATTAATTAG	Involved in the nitrogen response	2
TAAAGSTKST1	TAAAG	Guard cell-specific gene expression	254
GT1CONSENSUS	GRWAAW	GT-1 binding site in many light-regulated genes	260
AGATCONSENSUS	TTWCCWWWWNNGGWW	Function in flower development	3
BOXIIPCCHS	ACGTGGC	Essential for light regulation	9
CRTDREHVCBF2	GTCGAC	Regulation of low-temperature responsive genes	33
CTRMCAMV35S	TCTCTCTCT	Regulation of low-temperature responsive genes	13
ABRELATERD1	ACGTG	Early responsive to dehydration	157
ABREOSRAB21	ACGTSSSC	Early responsive to dehydration	3
DRECRTCOREAT	RCCGAC	Drought/high-light/cold responsive	109
DRE1COREZMRAB17	ACCGAGA	Drought response	102
BIHD1OS	TGTCA	Disease resistance responses	242
MYB1AT	WAACCA	Dehydration-responsive gene	236
MYB2AT	TAACTG	Dehydration-responsive gene	120
MYB1LEPR	GTTAGTT	Dehydration-responsive gene	29
MYB26PS	GTTAGGTT	Dehydration-responsive gene	10
CBFHV	RYCGAC	Dehydration-responsive element (DRE)	179
MYCCONSENSUSAT	CANNTG	Dehydration-responsive	260
MYBCORE	CNGTTR	Dehydration/Water stress	258
MYCATRD22	CACATG	Dehydration/Water stress	123
MYBATRD22	CTAACCA	Dehydration/Water stress	19
AGMOTIFNTMYB2	AGATCCAA	Defense-related gene	49
CARGATCONSENSUS	CCWWWWWWGG	Component of the vernalization (low-temperature)	32
TBOXATGAPB	ACTTTG	Chloroplast glyceraldehyde-3-phosphate dehydrogenase(GADPH)	187
GATABOX	GATA	Chlorophyll a/b binding protein/light regulations	260
CMSRE1IBSPOA	TGGACGG	Carbohydrate Metabolite Signal Responsive Element 1	18
TCA1MOTIF	TCATCTTCTT	Salicylic acid/stress induced	26
ACGTABREMOTIFA2OSEM	ACGTGKC	ABA-responsive expression	27
ABREATCONSENSUS	YACGTGGC	ABA-responsive elements (ABREs)	5
ABRECE1HVA22	TGCCACCGG	ABA-responsive elements (ABREs)	3
ABREATRD22	RYACGTGGYR	ABA-responsive elements (ABREs)	2
ABREBZMRAB28	TCCACGTCTC	ABA-responsive elements (ABREs)	2
ABREDISTBBNNAPA	GCCACTTGTC	ABA-responsive elements (ABREs)	1
WRKY71OS	TGAC	A transcriptional repressor of the gibberellin signaling pathway	260
E2F1OSPCNA	GCGGGAAA	Involved in transcriptional activation in actively dividing cells and tissue	5
DPBFCOREDCDC3	ACACNNG	Abscisic acid response gene	202

R: G A (puRine); Y: T C (pYrimidine); K: G T (Keto); M: A C (aMino); S: G C (Strong); W: A T (Weak) and N: A G C T (aNy).

## Supplementary Materials

Supplementary materials can be found at http://www.mdpi.com/1422-0067/19/6/1614/s1.

## Abbreviations

GBS	Genotyping by sequencing
WGS	Whole genome shotgun
GO	Gene ontology
PCD	Programmed cell death
ABA	Abscisic acid
MAS	Marker assisted selection
MPS	Massively parallel sequencing
NGS	Next generation sequencing
