# Supplementary material for: GBS Mapping and Analysis of Genes Conserved between Gossypium tomentosum and Gossypium hirsutum Cotton Cultivars that Respond to Drought Stress at the Seedling Stage of the BC2F2 Generation
Source: Int J Mol Sci. 2018 May 30;19(6):1614. doi: 10.3390/ijms19061614 (PMC6032168; doi:10.3390/ijms19061614)
Supplement: Supplementary file 1 [file ijms-19-01614-s001.zip › Supplimentary/Supplementary Table 1.Sequencing Quality Statistics.docx]

Supplementary Table 1: Sequencing Quality Statistics

| Sample ID | Raw Data (Gbp) | Clean Data (Gbp) | Clean Data Percentage (%) | Q30 Percentage (%) | GC Percentage（%） | Sample ID | Raw Data (Gbp) | Clean Data (Gbp) | Clean Data Percentage (%) | Q30 Percentage (%) | GC Percentage（%） |
| --- | --- | --- | --- | --- | --- | --- | --- | --- | --- | --- | --- |
| 1-Gh | 5.26 | 4.68 | 88.95 | 94.31 | 41.17 | 39 | 2.62 | 2.28 | 87.02 | 94.28 | 43.99 |
| 2-Gt | 7.44 | 6.8 | 91.39 | 94.75 | 37.76 | 40 | 1.51 | 1.35 | 89.4 | 94.85 | 43.92 |
| 3_BC_2_F_1_ | 4.18 | 3.75 | 89.71 | 94.48 | 43.1 | 41 | 1.69 | 1.51 | 89.35 | 94.62 | 43.39 |
| 4 | 2.09 | 1.87 | 89.47 | 94.21 | 43.25 | 42 | 3.04 | 2.73 | 89.8 | 94.39 | 44.03 |
| 5 | 2.95 | 2.65 | 89.83 | 94.34 | 42.91 | 43 | 1.46 | 1.29 | 88.36 | 94.58 | 43.14 |
| 6 | 3.01 | 2.69 | 89.37 | 94.36 | 42.94 | 44 | 1.33 | 1.15 | 86.47 | 94.57 | 43.41 |
| 7 | 1.66 | 1.49 | 89.76 | 94.37 | 43.03 | 45 | 2.49 | 2.2 | 88.35 | 94.64 | 42.96 |
| 8 | 2.2 | 1.97 | 89.55 | 94.23 | 43.2 | 46 | 1.92 | 1.71 | 89.06 | 94.61 | 43.63 |
| 9 | 2.83 | 1.97 | 69.61 | 95.48 | 43.19 | 47 | 1.99 | 1.76 | 88.44 | 94.41 | 42.82 |
| 10 | 1.79 | 1.42 | 79.33 | 95.38 | 42.86 | 48 | 2.79 | 2.49 | 89.25 | 94.44 | 43.82 |
| 11 | 2.59 | 2.02 | 77.99 | 95.47 | 43.78 | 49 | 1.98 | 1.77 | 89.39 | 94.65 | 43.16 |
| 12 | 1.37 | 1.14 | 83.21 | 94.59 | 43.6 | 50 | 2.32 | 1.79 | 77.16 | 95.71 | 42.84 |
| 13 | 1.71 | 1.29 | 75.44 | 95.4 | 43.31 | 51 | 2.08 | 1.87 | 89.9 | 94.63 | 43.12 |
| 14 | 2.52 | 2.09 | 82.94 | 95.84 | 43.33 | 52 | 3.14 | 2.78 | 88.54 | 94.82 | 42.88 |
| 15 | 2.29 | 1.98 | 86.46 | 94.62 | 43.15 | 53 | 1.44 | 1.24 | 86.11 | 93.59 | 44.09 |
| 16 | 2.12 | 1.86 | 87.74 | 94.55 | 42.8 | 54 | 1.46 | 1.28 | 87.67 | 94.97 | 43.09 |
| 17 | 1.28 | 1.1 | 85.94 | 94.56 | 42.57 | 55 | 2.69 | 1.77 | 65.8 | 95.75 | 42.94 |
| 18 | 1.21 | 1.07 | 88.43 | 94.61 | 42.84 | 56 | 1.76 | 1.49 | 84.66 | 94.61 | 43.63 |
| 19 | 1.73 | 1.54 | 89.02 | 94.09 | 43.92 | 57 | 2.44 | 2.16 | 88.52 | 93.9 | 43.68 |
| 20 | 1.62 | 1.31 | 80.86 | 95.46 | 43.1 | 58 | 2.51 | 1.94 | 77.29 | 95.99 | 42.63 |
| 21 | 1.5 | 1.3 | 86.67 | 94.33 | 43.04 | 59 | 1.83 | 1.59 | 86.89 | 94.56 | 43.19 |
| 22 | 1.33 | 1.19 | 89.47 | 94.6 | 42.76 | 60 | 6.93 | 6.16 | 88.89 | 93.89 | 43.12 |
| 23 | 1.21 | 1.08 | 89.26 | 94.27 | 43.43 | 61 | 3.9 | 3.37 | 86.41 | 93.96 | 42.6 |
| 24 | 2.38 | 2.13 | 89.5 | 94.27 | 43.19 | 62 | 1.92 | 1.7 | 88.54 | 94.6 | 42.8 |
| 25 | 2.36 | 1.91 | 80.93 | 95.41 | 43.51 | 63 | 3.75 | 3.28 | 87.47 | 94.31 | 42.85 |
| 26 | 2.08 | 1.79 | 86.06 | 95.51 | 43.36 | 64 | 2.11 | 1.66 | 78.67 | 94.17 | 43.94 |
| 27 | 2.83 | 2.29 | 80.92 | 95.78 | 43.42 | 65 | 1.41 | 1.2 | 85.11 | 93.71 | 43.99 |
| 28 | 2.82 | 2.51 | 89.01 | 94.51 | 42.85 | 66 | 1.7 | 1.49 | 87.82 | 94.75 | 42.93 |
| 29 | 2.89 | 2.58 | 89.27 | 94.46 | 43.69 | 67 | 3.02 | 2.66 | 88.08 | 94.21 | 43.97 |
| 30 | 1.21 | 1.08 | 89.26 | 94.81 | 43.68 | 68 | 7.32 | 6.53 | 89.21 | 93.99 | 43.17 |
| 31 | 1.61 | 1.42 | 88.2 | 94.97 | 43.58 | 69 | 3.66 | 3.21 | 87.7 | 93.99 | 42.93 |
| 32 | 1.64 | 1.35 | 82.32 | 94.57 | 42.58 | 70 | 1.68 | 1.46 | 86.9 | 93.94 | 43.17 |
| 33 | 1.5 | 1.34 | 89.33 | 94.59 | 43.63 | 71 | 1.2 | 1.05 | 87.5 | 94.62 | 43.11 |
| 34 | 1.52 | 1.32 | 86.84 | 94.6 | 42.83 | 72 | 1.64 | 1.43 | 87.2 | 94.34 | 43.58 |
| 35 | 1.68 | 1.45 | 86.31 | 94.28 | 43.55 | 73 | 1.27 | 1.05 | 82.68 | 93.73 | 43.94 |
| 36 | 3.05 | 2.71 | 88.85 | 94.37 | 43.94 | 74 | 1.54 | 1.29 | 83.77 | 94.73 | 43.19 |
| 37 | 1.51 | 1.25 | 82.78 | 94.76 | 43.58 | 75 | 2.73 | 2.37 | 86.81 | 94.35 | 42.91 |
| 38 | 1.92 | 1.71 | 89.06 | 94.67 | 42.88 | 76 | 1.41 | 1.19 | 84.4 | 94.33 | 43 |
| Sample ID | Raw Data (Gbp) | Clean Data (Gbp) | Clean Data Percentage (%) | Q30 Percentage (%) | GC Percentage（%） | Sample ID | Raw Data (Gbp) | Clean Data (Gbp) | Clean Data Percentage (%) | Q30 Percentage (%) | GC Percentage（%） |
| 77 | 6.54 | 5.82 | 88.99 | 93.97 | 43.19 | 117 | 2.39 | 2.19 | 91.63 | 95.1 | 43.15 |
| 78 | 1.9 | 1.7 | 89.59 | 94.68 | 43.05 | 118 | 2.03 | 1.73 | 85.22 | 95.15 | 42.77 |
| 79 | 1.3 | 1.14 | 87.69 | 93.75 | 43.63 | 119 | 2.04 | 1.86 | 91.18 | 95.14 | 42.98 |
| 80 | 2.09 | 1.96 | 93.58 | 95.5 | 41.88 | 120 | 2.64 | 2.43 | 92.05 | 94.9 | 42.68 |
| 81 | 2.5 | 2.19 | 87.6 | 94.04 | 43.09 | 121 | 1.52 | 1.26 | 82.89 | 95.27 | 43.61 |
| 82 | 1.47 | 1.24 | 84.35 | 94.27 | 42.86 | 122 | 1.89 | 1.65 | 87.3 | 95.68 | 42.51 |
| 83 | 1.28 | 1.13 | 88.28 | 94.3 | 43 | 123 | 1.46 | 1.21 | 82.88 | 95.51 | 43.44 |
| 84 | 2.6 | 2.23 | 85.77 | 94.33 | 43.2 | 124 | 5.17 | 4.75 | 91.88 | 94.86 | 42.76 |
| 85 | 1.53 | 1.21 | 79.08 | 94.29 | 42.84 | 125 | 2.4 | 1.67 | 69.58 | 95.21 | 43.69 |
| 86 | 1.84 | 1.59 | 86.41 | 94.24 | 42.92 | 126 | 1.91 | 1.45 | 75.92 | 95.3 | 43.19 |
| 87 | 1.88 | 1.58 | 84.04 | 95.42 | 43.6 | 127 | 2.18 | 2.01 | 92.2 | 94.9 | 42.74 |
| 88 | 2.33 | 2.03 | 87.12 | 94.32 | 42.94 | 128 | 1.64 | 1.5 | 91.46 | 94.57 | 43.58 |
| 89 | 2.55 | 2.21 | 86.67 | 94.29 | 43.35 | 129 | 1.28 | 1.09 | 85.16 | 95.36 | 43.43 |
| 90 | 1.37 | 1.15 | 83.94 | 94.42 | 42.71 | 130 | 2.64 | 2.3 | 87.12 | 94.51 | 43.14 |
| 91 | 1.53 | 1.42 | 92.81 | 94.99 | 42.34 | 131 | 1.75 | 1.33 | 76 | 95.39 | 43.5 |
| 92 | 1.97 | 1.61 | 81.73 | 94.37 | 42.78 | 132 | 1.57 | 1.43 | 91.08 | 95.3 | 42.85 |
| 93 | 1.81 | 1.53 | 84.53 | 94.39 | 42.63 | 133 | 1.49 | 1.3 | 87.25 | 95.23 | 43.32 |
| 94 | 1.57 | 1.14 | 72.61 | 95.48 | 43.51 | 134 | 2.77 | 2.4 | 86.64 | 94.54 | 42.73 |
| 95 | 2.32 | 2.05 | 88.36 | 95.73 | 43.5 | 135 | 1.65 | 1.16 | 70.3 | 95.45 | 42.91 |
| 96 | 1.66 | 1.53 | 92.17 | 94.66 | 43.55 | 136 | 1.8 | 1.43 | 79.44 | 95.57 | 43.41 |
| 97 | 1.22 | 1.1 | 90.16 | 94.72 | 43.31 | 137 | 1.46 | 1.33 | 91.1 | 94.51 | 43.76 |
| 98 | 2.16 | 1.86 | 86.11 | 95.59 | 43.37 | 138 | 1.77 | 1.5 | 84.75 | 95.31 | 43.42 |
| 99 | 3.78 | 3.32 | 87.83 | 94.17 | 43.63 | 139 | 2.14 | 1.66 | 77.57 | 95.27 | 43.7 |
| 100 | 1.38 | 1.12 | 81.16 | 95.62 | 43.37 | 140 | 2.17 | 1.61 | 74.19 | 95.46 | 43.73 |
| 101 | 2.42 | 2.1 | 86.78 | 93.76 | 48.89 | 141 | 2.03 | 1.85 | 91.13 | 94.85 | 42.8 |
| 102 | 5.5 | 5.09 | 92.55 | 94.95 | 42.47 | 142 | 1.45 | 1.25 | 86.21 | 95.24 | 42.92 |
| 103 | 1.43 | 1.24 | 86.71 | 95.74 | 42.37 | 143 | 1.93 | 1.55 | 80.31 | 95.23 | 43.05 |
| 104 | 1.56 | 1.35 | 86.54 | 94.3 | 42.76 | 144 | 1.64 | 1.43 | 87.2 | 95.4 | 42.69 |
| 105 | 1.87 | 1.57 | 83.96 | 95.79 | 42.43 | 145 | 1.34 | 1.23 | 91.79 | 94.9 | 42.56 |
| 106 | 2.82 | 2.44 | 86.52 | 94.56 | 43.36 | 146 | 1.82 | 1.62 | 89.01 | 95.08 | 43.12 |
| 107 | 1.28 | 1.18 | 92.19 | 95.21 | 43.45 | 147 | 2.57 | 2.15 | 83.63 | 95.8 | 42.37 |
| 108 | 1.27 | 1.11 | 87.4 | 95.38 | 42.72 | 148 | 1.69 | 1.47 | 86.98 | 95.49 | 42.96 |
| 109 | 1.61 | 1.49 | 92.55 | 95.06 | 42.43 | 149 | 1.77 | 1.5 | 84.75 | 95.66 | 43.39 |
| 110 | 3.03 | 2.81 | 92.74 | 94.9 | 42.59 | 150 | 1.92 | 1.58 | 82.29 | 95.26 | 43.55 |
| 111 | 1.41 | 1.27 | 90.07 | 95.18 | 42.92 | 151 | 2.04 | 1.41 | 69.12 | 95.25 | 43.54 |
| 112 | 1.49 | 1.29 | 86.58 | 94.56 | 43.01 | 152 | 2.47 | 2.06 | 83.4 | 96.02 | 42.53 |
| 113 | 1.94 | 1.71 | 88.14 | 95.77 | 42.45 | 153 | 2.08 | 1.81 | 87.02 | 95.41 | 42.76 |
| 114 | 2.1 | 1.82 | 86.67 | 95.73 | 42.61 | 154 | 1.18 | 1.05 | 88.98 | 94.54 | 43.72 |
| 115 | 1.48 | 1.36 | 91.89 | 95.03 | 42.55 | 155 | 1.79 | 1.48 | 82.68 | 95.64 | 43.6 |
| 116 | 1.49 | 1.36 | 91.28 | 95.44 | 43.03 | 156 | 2.66 | 2.35 | 88.35 | 94.95 | 43.12 |
| Sample ID | Raw Data (Gbp) | Clean Data (Gbp) | Clean Data Percentage (%) | Q30 Percentage (%) | GC Percentage（%） | Sample ID | Raw Data (Gbp) | Clean Data (Gbp) | Clean Data Percentage (%) | Q30 Percentage (%) | GC Percentage（%） |
| 157 | 1.89 | 1.72 | 91.01 | 94.57 | 43.61 | 182 | 6.12 | 5.49 | 89.71 | 94.8 | 43.09 |
| 158 | 1.18 | 1.08 | 91.53 | 94.94 | 42.44 | 183 | 4.89 | 4.34 | 88.75 | 94.77 | 42.95 |
| 159 | 1.6 | 1.45 | 90.62 | 94.89 | 42.75 | 184 | 2.54 | 2.23 | 87.8 | 94.6 | 43.11 |
| 160 | 1.21 | 1.16 | 95.6 | 95.43 | 41.16 | 185 | 2.92 | 2.51 | 85.96 | 94.65 | 42.86 |
| 161 | 2.68 | 2.37 | 88.43 | 95.68 | 42.48 | 186 | 2.42 | 2.15 | 88.84 | 94.53 | 43.64 |
| 162 | 2.4 | 2.06 | 85.83 | 95.77 | 42.61 | 187 | 2.48 | 2.09 | 84.27 | 94.74 | 43.8 |
| 163 | 3.02 | 2.55 | 84.44 | 95.75 | 42.5 | 188 | 1.97 | 1.74 | 88.32 | 94.65 | 42.9 |
| 164 | 3.02 | 2.71 | 89.74 | 94.81 | 43.11 | 189 | 3.78 | 3.37 | 89.15 | 94.54 | 43.38 |
| 165 | 2.22 | 1.81 | 81.53 | 95.63 | 43.4 | 190 | 1.28 | 1.13 | 88.28 | 94.67 | 42.87 |
| 166 | 3.32 | 2.4 | 72.29 | 95.61 | 42.66 | 191 | 3.65 | 3.23 | 88.49 | 94.61 | 43.15 |
| 167 | 4.15 | 3.81 | 91.81 | 94.88 | 42.67 | 192 | 1.35 | 1.17 | 86.67 | 94.53 | 42.8 |
| 168 | 1.35 | 1.24 | 91.85 | 94.87 | 42.54 | 193 | 2.88 | 2.55 | 88.54 | 94.56 | 43.27 |
| 169 | 1.3 | 1.18 | 90.77 | 94.77 | 42.85 | 194 | 2.11 | 1.81 | 85.78 | 94.61 | 42.96 |
| 170 | 1.69 | 1.52 | 89.94 | 94.83 | 43.14 | 195 | 1.73 | 1.49 | 86.13 | 94.67 | 42.65 |
| 171 | 1.39 | 1.15 | 82.73 | 94.51 | 42.98 | 196 | 1.72 | 1.52 | 88.37 | 94.54 | 42.99 |
| 172 | 2.72 | 2.31 | 84.93 | 95.69 | 42.62 | 197 | 1.19 | 1.02 | 85.71 | 94.53 | 43.02 |
| 173 | 3.15 | 2.78 | 88.25 | 94.97 | 42.82 | 198 | 1.38 | 1.19 | 86.23 | 94.54 | 42.99 |
| 174 | 3.54 | 3.16 | 89.27 | 94.64 | 43.79 | 199 | 4.02 | 3.59 | 89.3 | 94.52 | 43.13 |
| 175 | 2.16 | 1.89 | 87.5 | 94.61 | 42.9 | 200 | 2.26 | 1.94 | 85.84 | 94.72 | 43.02 |
| 176 | 2.52 | 2.22 | 88.1 | 94.64 | 42.71 | 201 | 3.56 | 3.21 | 90.17 | 94.62 | 43.15 |
| 177 | 2.31 | 2.04 | 88.31 | 94.69 | 43.53 | 202 | 5.03 | 4.51 | 89.66 | 94.49 | 43.17 |
| 178 | 1.53 | 1.33 | 86.93 | 94.63 | 42.81 | 203 | 3.33 | 2.83 | 84.98 | 94.54 | 42.98 |
| 179 | 1.76 | 1.55 | 88.07 | 94.67 | 42.8 |  |  |  |  |  |  |
| 180 | 3.5 | 3.01 | 86 | 94.86 | 43.15 |  |  |  |  |  |  |
| 181 | 3.18 | 2.79 | 87.74 | 95.03 | 43.03 |  |  |  |  |  |  |
