# Supplementary material for: GBS Mapping and Analysis of Genes Conserved between Gossypium tomentosum and Gossypium hirsutum Cotton Cultivars that Respond to Drought Stress at the Seedling Stage of the BC2F2 Generation
Source: Int J Mol Sci. 2018 May 30;19(6):1614. doi: 10.3390/ijms19061614 (PMC6032168; doi:10.3390/ijms19061614)
Supplement: Supplementary file 1 [file ijms-19-01614-s001.zip › Supplimentary/Supplementary Table 2 InDel data statistics.docx]

Supplementary Table 2 InDel data statistics

| Sample ID | Insert Number | Delete Number | Heterozygosity Number | Homozygosity Number | Sample ID | Insert Number | Delete Number | Heterozygosity Number | Homozygosity Number |
| --- | --- | --- | --- | --- | --- | --- | --- | --- | --- |
| 1_Gh | 86,467 | 76,206 | 50,170 | 112,503 | 50 | 13,276 | 11,122 | 17,156 | 7,242 |
| 2_Gt | 371,852 | 352,463 | 522,862 | 201,453 | 51 | 13,046 | 11,151 | 16,376 | 7,821 |
| 3_F1 | 18,179 | 15,813 | 20,895 | 13,097 | 52 | 19,064 | 16,659 | 20,780 | 14,943 |
| 4 | 13,691 | 11,574 | 15,844 | 9,421 | 53 | 10,275 | 8,718 | 12,179 | 6,814 |
| 5 | 15,926 | 13,647 | 19,328 | 10,245 | 54 | 13,500 | 11,630 | 15,428 | 9,702 |
| 6 | 16,849 | 14,564 | 19,211 | 12,202 | 55 | 17,232 | 15,003 | 17,630 | 14,605 |
| 7 | 14,495 | 12,189 | 15,637 | 11,047 | 56 | 12,008 | 10,350 | 14,904 | 7,454 |
| 8 | 13,989 | 11,588 | 16,853 | 8,724 | 57 | 15,595 | 13,281 | 16,816 | 12,060 |
| 9 | 15,684 | 13,599 | 16,309 | 12,974 | 58 | 17,832 | 15,440 | 18,839 | 14,433 |
| 10 | 12,004 | 10,038 | 15,465 | 6,577 | 59 | 16,177 | 13,786 | 16,671 | 13,292 |
| 11 | 13,143 | 11,136 | 15,054 | 9,225 | 60 | 17,866 | 15,322 | 21,853 | 11,335 |
| 12 | 12,447 | 10,573 | 13,774 | 9,246 | 61 | 17,524 | 15,122 | 20,273 | 12,373 |
| 13 | 12,729 | 10,492 | 14,765 | 8,456 | 62 | 13,812 | 11,608 | 16,880 | 8,540 |
| 14 | 11,515 | 9,766 | 14,429 | 6,852 | 63 | 15,804 | 13,206 | 19,303 | 9,707 |
| 15 | 13,571 | 11,450 | 17,517 | 7,504 | 64 | 13,746 | 11,316 | 14,993 | 10,069 |
| 16 | 14,632 | 12,394 | 17,269 | 9,757 | 65 | 11,636 | 9,741 | 12,656 | 8,721 |
| 17 | 11,634 | 9,669 | 14,309 | 6,994 | 66 | 11,431 | 9,625 | 14,621 | 6,435 |
| 18 | 11,720 | 9,708 | 14,026 | 7,402 | 67 | 14,379 | 12,086 | 16,824 | 9,641 |
| 19 | 10,249 | 8,616 | 13,821 | 5,044 | 68 | 18,168 | 15,517 | 22,463 | 11,222 |
| 20 | 13,717 | 11,656 | 14,084 | 11,289 | 69 | 19,257 | 16,907 | 20,278 | 15,886 |
| 21 | 12,065 | 10,107 | 14,343 | 7,829 | 70 | 13,553 | 11,559 | 15,564 | 9,548 |
| 22 | 14,460 | 12,170 | 15,168 | 11,462 | 71 | 12,659 | 10,670 | 14,121 | 9,208 |
| 23 | 11,001 | 9,108 | 12,570 | 7,539 | 72 | 13,623 | 11,436 | 15,276 | 9,783 |
| 24 | 14,118 | 11,740 | 16,839 | 9,019 | 73 | 9,367 | 7,791 | 10,952 | 6,206 |
| 25 | 12,939 | 11,100 | 14,991 | 9,048 | 74 | 12,992 | 10,989 | 15,178 | 8,803 |
| 26 | 11,535 | 9,814 | 14,897 | 6,452 | 75 | 16,308 | 13,927 | 19,170 | 11,065 |
| 27 | 11,661 | 9,896 | 15,266 | 6,291 | 76 | 11,434 | 9,636 | 14,682 | 6,388 |
| 28 | 18,454 | 15,792 | 19,616 | 14,630 | 77 | 18,745 | 16,206 | 21,551 | 13,400 |
| 29 | 16,229 | 13,888 | 17,931 | 12,186 | 78 | 13,015 | 11,237 | 15,432 | 8,820 |
| 30 | 10,820 | 8,927 | 12,942 | 6,805 | 79 | 10,055 | 8,360 | 12,753 | 5,662 |
| 31 | 13,470 | 11,422 | 15,296 | 9,596 | 80 | 13,284 | 11,391 | 15,952 | 8,723 |
| 32 | 13,421 | 10,991 | 14,727 | 9,685 | 81 | 15,419 | 13,256 | 18,003 | 10,672 |
| 33 | 12,019 | 10,340 | 13,895 | 8,464 | 82 | 15,281 | 12,921 | 15,790 | 12,412 |
| 34 | 13,211 | 10,718 | 14,401 | 9,528 | 83 | 13,174 | 11,087 | 14,738 | 9,523 |
| 35 | 13,560 | 11,285 | 15,495 | 9,350 | 84 | 16,605 | 14,169 | 18,846 | 11,928 |
| 36 | 15,184 | 12,845 | 17,388 | 10,641 | 85 | 12,902 | 10,873 | 15,340 | 8,435 |
| 37 | 13,141 | 11,004 | 14,335 | 9,810 | 86 | 16,139 | 13,901 | 17,407 | 12,633 |
| 38 | 17,115 | 14,624 | 17,364 | 14,375 | 87 | 12,132 | 10,448 | 13,434 | 9,146 |
| 39 | 12,212 | 10,037 | 16,273 | 5,976 | 88 | 12,118 | 10,204 | 17,425 | 4,897 |
| 40 | 12,303 | 10,547 | 14,163 | 8,687 | 89 | 15,276 | 12,760 | 17,005 | 11,031 |
| 41 | 12,896 | 10,876 | 15,091 | 8,681 | 90 | 12,934 | 10,702 | 14,549 | 9,087 |
| 42 | 13,407 | 11,302 | 16,990 | 7,719 | 91 | 14,365 | 12,194 | 15,911 | 10,648 |
| 43 | 11,877 | 10,015 | 14,145 | 7,747 | 92 | 13,642 | 11,398 | 16,789 | 8,251 |
| 44 | 12,506 | 10,415 | 13,566 | 9,355 | 93 | 15,004 | 12,422 | 16,415 | 11,011 |
| 45 | 15,168 | 12,722 | 17,904 | 9,986 | 94 | 9,085 | 7,587 | 10,989 | 5,683 |
| 46 | 13,161 | 11,146 | 15,490 | 8,817 | 95 | 13,722 | 11,425 | 14,548 | 10,599 |
| 47 | 13,114 | 10,949 | 16,790 | 7,273 | 96 | 12,963 | 10,965 | 14,232 | 9,696 |
| 48 | 16,245 | 13,611 | 17,491 | 12,365 | 97 | 12,498 | 10,557 | 12,940 | 10,115 |
| 49 | 13,512 | 11,176 | 16,119 | 8,569 | 98 | 13,821 | 11,977 | 15,565 | 10,233 |
| Sample ID | Insert Number | Delete Number | Heterozygosity Number | Homozygosity Number | Sample ID | Insert Number | Delete Number | Heterozygosity Number | Homozygosity Number |
| 99 | 16,914 | 14,291 | 19,746 | 11,459 | 150 | 11,364 | 9,621 | 13,365 | 7,620 |
| 100 | 9,690 | 8,275 | 11,481 | 6,484 | 151 | 11,615 | 9,888 | 13,181 | 8,322 |
| 101 | 735 | 573 | 759 | 549 | 152 | 15,778 | 13,640 | 18,948 | 10,470 |
| 102 | 16,440 | 14,057 | 21,297 | 9,200 | 153 | 15,620 | 13,245 | 17,783 | 11,082 |
| 103 | 13,117 | 11,310 | 14,851 | 9,576 | 154 | 9,905 | 8,263 | 11,321 | 6,847 |
| 104 | 13,332 | 11,308 | 15,993 | 8,647 | 155 | 12,080 | 10,169 | 12,853 | 9,396 |
| 105 | 14,690 | 12,441 | 16,623 | 10,508 | 156 | 15,198 | 12,574 | 18,097 | 9,675 |
| 106 | 1,428 | 1,139 | 1,535 | 1,032 | 157 | 13,583 | 11,397 | 15,067 | 9,913 |
| 107 | 995 | 796 | 1,087 | 704 | 158 | 12,157 | 10,112 | 14,190 | 8,079 |
| 108 | 14,364 | 12,341 | 15,132 | 11,573 | 159 | 11,039 | 8,919 | 14,530 | 5,428 |
| 109 | 13,868 | 11,786 | 15,892 | 9,762 | 160 | 12,989 | 11,121 | 12,760 | 11,350 |
| 110 | 15,624 | 13,600 | 19,118 | 10,106 | 161 | 14,979 | 12,668 | 18,926 | 8,721 |
| 111 | 11,742 | 9,856 | 14,980 | 6,618 | 162 | 15,709 | 13,438 | 18,925 | 10,222 |
| 112 | 12,523 | 10,418 | 15,047 | 7,894 | 163 | 16,504 | 14,192 | 19,528 | 11,168 |
| 113 | 13,986 | 12,001 | 16,877 | 9,110 | 164 | 17,256 | 14,845 | 19,452 | 12,649 |
| 114 | 14,061 | 11,989 | 16,697 | 9,353 | 165 | 12,324 | 10,543 | 14,462 | 8,405 |
| 115 | 11,969 | 9,988 | 15,017 | 6,940 | 166 | 15,881 | 13,735 | 19,365 | 10,251 |
| 116 | 11,372 | 9,411 | 15,069 | 5,714 | 167 | 18,460 | 15,873 | 20,955 | 13,378 |
| 117 | 13,277 | 11,234 | 17,498 | 7,013 | 168 | 12,901 | 10,748 | 15,013 | 8,636 |
| 118 | 16,442 | 13,925 | 17,592 | 12,775 | 169 | 12,133 | 10,417 | 14,113 | 8,437 |
| 119 | 14,300 | 12,098 | 17,588 | 8,810 | 170 | 13,862 | 11,748 | 14,985 | 10,625 |
| 120 | 13,731 | 11,677 | 17,620 | 7,788 | 171 | 13,133 | 10,968 | 14,645 | 9,456 |
| 121 | 10,551 | 9,014 | 11,734 | 7,831 | 172 | 16,138 | 13,894 | 19,585 | 10,447 |
| 122 | 14,274 | 12,247 | 16,365 | 10,156 | 173 | 16,322 | 13,986 | 19,635 | 10,673 |
| 123 | 10,228 | 8,449 | 11,647 | 7,030 | 174 | 17,619 | 15,110 | 19,891 | 12,838 |
| 124 | 18,493 | 16,197 | 21,117 | 13,573 | 175 | 15,739 | 13,379 | 17,569 | 11,549 |
| 125 | 12,321 | 10,590 | 13,613 | 9,298 | 176 | 17,846 | 15,654 | 19,818 | 13,682 |
| 126 | 11,403 | 9,742 | 13,439 | 7,706 | 177 | 15,938 | 13,908 | 17,662 | 12,184 |
| 127 | 11,754 | 9,926 | 16,548 | 5,132 | 178 | 12,817 | 10,359 | 15,063 | 8,113 |
| 128 | 12,120 | 10,425 | 13,764 | 8,781 | 179 | 14,033 | 11,838 | 16,415 | 9,456 |
| 129 | 9,083 | 7,494 | 10,950 | 5,627 | 180 | 17,820 | 15,364 | 20,402 | 12,782 |
| 130 | 17,684 | 15,259 | 19,273 | 13,670 | 181 | 17,822 | 15,149 | 20,118 | 12,853 |
| 131 | 9,552 | 7,835 | 12,084 | 5,303 | 182 | 20,129 | 17,529 | 23,074 | 14,584 |
| 132 | 13,139 | 10,917 | 15,664 | 8,392 | 183 | 16,958 | 14,568 | 21,387 | 10,139 |
| 133 | 14,122 | 11,974 | 15,167 | 10,929 | 184 | 13,858 | 11,636 | 17,395 | 8,099 |
| 134 | 16,297 | 13,876 | 19,498 | 10,675 | 185 | 14,988 | 12,465 | 18,384 | 9,069 |
| 135 | 12,291 | 10,135 | 14,269 | 8,157 | 186 | 14,323 | 11,867 | 17,121 | 9,069 |
| 136 | 10,497 | 8,667 | 12,332 | 6,832 | 187 | 12,508 | 10,289 | 16,541 | 6,256 |
| 137 | 10,597 | 9,099 | 12,554 | 7,142 | 188 | 14,303 | 12,053 | 17,151 | 9,205 |
| 138 | 12,239 | 10,305 | 13,607 | 8,937 | 189 | 14,467 | 11,942 | 18,015 | 8,394 |
| 139 | 11,926 | 10,253 | 13,575 | 8,604 | 190 | 11,291 | 9,318 | 13,605 | 7,004 |
| 140 | 10,649 | 8,769 | 12,729 | 6,689 | 191 | 17,001 | 14,699 | 20,274 | 11,426 |
| 141 | 15,775 | 13,562 | 16,962 | 12,375 | 192 | 12,617 | 9,959 | 13,482 | 9,094 |
| 142 | 14,520 | 12,164 | 15,670 | 11,014 | 193 | 15,512 | 13,404 | 17,968 | 10,948 |
| 143 | 13,159 | 11,024 | 15,962 | 8,221 | 194 | 15,307 | 13,106 | 17,610 | 10,803 |
| 144 | 14,634 | 12,202 | 15,759 | 11,077 | 195 | 12,635 | 10,597 | 16,024 | 7,208 |
| 145 | 12,868 | 11,211 | 14,970 | 9,109 | 196 | 15,069 | 12,744 | 16,178 | 11,635 |
| 146 | 13,934 | 11,914 | 16,287 | 9,561 | 197 | 10,108 | 8,273 | 12,948 | 5,433 |
| 147 | 16,744 | 14,674 | 19,242 | 12,176 | 198 | 12,609 | 10,867 | 14,601 | 8,875 |
| 148 | 10,362 | 8,646 | 14,125 | 4,883 | 199 | 17,090 | 14,867 | 19,710 | 12,247 |
| 149 | 11,796 | 10,039 | 13,325 | 8,510 | 200 | 14,902 | 12,179 | 16,820 | 10,261 |
| Sample ID | Insert Number | Delete Number | Heterozygosity Number | Homozygosity Number | Sample ID | Insert Number | Delete Number | Heterozygosity Number | Homozygosity Number |
| 201 | 16,152 | 13,906 | 18,804 | 11,254 | 203 | 19,105 | 16,345 | 19,546 | 15,904 |
| 202 | 14,033 | 11,715 | 19,432 | 6,316 |  |  |  |  |  |
