# Supplementary material for: GBS Mapping and Analysis of Genes Conserved between Gossypium tomentosum and Gossypium hirsutum Cotton Cultivars that Respond to Drought Stress at the Seedling Stage of the BC2F2 Generation
Source: Int J Mol Sci. 2018 May 30;19(6):1614. doi: 10.3390/ijms19061614 (PMC6032168; doi:10.3390/ijms19061614)
Supplement: Supplementary file 1 [file ijms-19-01614-s001.zip › Supplimentary/Supplementary Table 3 The GBS markers numbers distribution per the linkage groups, percentage coverage and density.docx]

Supplementary Table 3: The GBS markers numbers distribution per the linkage groups, percentage coverage and density

| Chromosomes/ linkage groups | Number of markers | Cover length (Mb) | Chromosome Length (Mb) | % coverage | Density (marker/Mb) |
| --- | --- | --- | --- | --- | --- |
| At_chr01 | 2306 | 99.85 | 99.88 | 100 | 23.10 |
| At_chr02 | 2368 | 83.28 | 83.45 | 99.8 | 28.40 |
| At_chr03 | 221 | 100.21 | 100.26 | 99.9 | 2.21 |
| At_chr04 | 2057 | 62.76 | 62.91 | 99.8 | 32.80 |
| At_chr05 | 778 | 91.98 | 92.05 | 99.9 | 8.46 |
| At_chr06 | 193 | 102.95 | 103.17 | 99.8 | 1.87 |
| At_chr07 | 1572 | 78.16 | 78.25 | 99.9 | 20.10 |
| At_chr08 | 1664 | 103.61 | 103.63 | 100 | 16.10 |
| At_chr09 | 1054 | 74.86 | 75.00 | 99.8 | 14.10 |
| At_chr10 | 1139 | 100.69 | 100.87 | 99.8 | 11.30 |
| At_chr11 | 1650 | 93.31 | 93.32 | 100 | 17.70 |
| At_chr12 | 353 | 87.40 | 87.48 | 99.9 | 4.04 |
| At_chr13 | 1285 | 78.02 | 79.96 | 97.6 | 16.50 |
| At_sub genome | **16,640** | **1157.08** | **1160.23** | **99.7** | **14.40** |
| Dt_chr01 | 198 | 60.92 | 61.46 | 99.1 | 3.25 |
| Dt_chr02 | 237 | 67.22 | 67.28 | 99.9 | 3.53 |
| Dt_chr03 | 161 | 46.67 | 46.69 | 100 | 3.45 |
| Dt_chr04 | 365 | 51.27 | 51.45 | 99.6 | 7.12 |
| Dt_chr05 | 109 | 60.24 | 61.93 | 97.3 | 1.81 |
| Dt_chr06 | 2419 | 64.09 | 64.29 | 99.7 | 37.70 |
| Dt_chr07 | 393 | 55.29 | 55.31 | 100 | 7.11 |
| Dt_chr08 | 1918 | 65.83 | 65.89 | 99.9 | 29.10 |
| Dt_chr09 | 852 | 50.91 | 51.00 | 99.8 | 16.70 |
| Dt_chr10 | 1854 | 62.79 | 63.37 | 99.1 | 29.50 |
| Dt_chr11 | 691 | 65.54 | 66.09 | 99.2 | 10.50 |
| Dt_chr12 | 1593 | 58.94 | 59.11 | 99.7 | 27.00 |
| Dt_chr13 | 1230 | 59.88 | 60.53 | 98.9 | 20.50 |
| Dt_sub genome | **12,020** | **769.60** | **774.42** | **99.4** | **15.60** |
| Totals | **28,660** | **1926.68** | **1934.65** | **99.6** | **14.90** |
