# Supplementary material for: GBS Mapping and Analysis of Genes Conserved between Gossypium tomentosum and Gossypium hirsutum Cotton Cultivars that Respond to Drought Stress at the Seedling Stage of the BC2F2 Generation
Source: Int J Mol Sci. 2018 May 30;19(6):1614. doi: 10.3390/ijms19061614 (PMC6032168; doi:10.3390/ijms19061614)
Supplement: Supplementary file 1 [file ijms-19-01614-s001.zip › Supplimentary/Supplementary Table 4 Analysis of the dominant gene domain, of the Pkinase (PF00069).docx]

Supplementary Table 4 : Analysis of the dominant gene domain, of the Pkinase (PF00069)

| Gene ID | Chro. | Gene Name | Description | Start | End | Strand | Domain | mapping position (bp) |
| --- | --- | --- | --- | --- | --- | --- | --- | --- |
| Gh_A05G2494 | chr05 | PDPK1 | 3-phosphoinositide-dependent protein kinase 1 | 33,159,116 | 33,169,797 | - | PF00069 | 33164457 |
| Gh_D01G1146 | chr15 | PDPK1 | 3-phosphoinositide-dependent protein kinase 1 | 25,209,957 | 25,215,862 | + | PF00069 | 25212910 |
| Gh_D05G2771 | chr19 | PDPK1 | 3-phosphoinositide-dependent protein kinase 1 | 30,239,850 | 30,245,733 | - | PF00069 | 30242792 |
| Gh_A05G3412 | chr05 | PDPK2 | 3-phosphoinositide-dependent protein kinase 2 | 88,999,039 | 89,007,310 | - | PF00069 | 89003175 |
| Gh_A05G2681 | chr05 | BAK1 | BRASSINOSTEROID INSENSITIVE 1-associated receptor kinase 1 | 44,698,345 | 44,700,378 | - | PF00069 | 44699362 |
| Gh_D01G1374 | chr15 | BAK1 | BRASSINOSTEROID INSENSITIVE 1-associated receptor kinase 1 | 40,497,327 | 40,499,360 | - | PF00069 | 40498344 |
| Gh_D01G1794 | chr15 | BAK1 | BRASSINOSTEROID INSENSITIVE 1-associated receptor kinase 1 | 55,065,788 | 55,067,821 | - | PF00069 | 55066805 |
| Gh_A06G1503 | chr06 | CCAMK | Calcium and calcium/calmodulin-dependent serine/threonine-protein kinase | 98,583,400 | 98,586,964 | - | PF00069 | 98585182 |
| Gh_D06G1869 | chr25 | CCAMK | Calcium and calcium/calmodulin-dependent serine/threonine-protein kinase | 59,177,952 | 59,184,725 | - | PF00069 | 59181339 |
| Gh_A10G1756 | chr10 | CPK1 | Calcium-dependent protein kinase 1 | 91,965,125 | 91,970,397 | - | PF00069 | 91967761 |
| Gh_A10G1756 | chr10 | CPK1 | Calcium-dependent protein kinase 1 | 91,965,125 | 91,970,397 | - | PF00069 | 91967761 |
| Gh_A13G0017 | chr13 | CPK1 | Calcium-dependent protein kinase 1 | 149,337 | 152,705 | - | PF00069 | 151021 |
| Gh_D10G2029 | chr20 | CPK1 | Calcium-dependent protein kinase 1 | 56,010,875 | 56,015,207 | - | PF00069 | 56013041 |
| Gh_D13G0033 | chr18 | CPK1 | Calcium-dependent protein kinase 1 | 246,690 | 250,046 | - | PF00069 | 248368 |
| Gh_D11G0314 | chr21 | CPK10 | Calcium-dependent protein kinase 10 | 2,722,348 | 2,725,395 | + | PF00069 | 2723872 |
| Gh_A02G0609 | Chr02 | CPK11 | Calcium-dependent protein kinase 11 | 9,500,456 | 9,503,183 | + | PF00069 | 9501820 |
| Gh_A05G2355 | chr05 | CPK11 | Calcium-dependent protein kinase 11 | 28,825,850 | 28,830,758 | + | PF00069 | 28828304 |
| Gh_D02G0663 | chr14 | CPK11 | Calcium-dependent protein kinase 11 | 9,261,983 | 9,264,700 | + | PF00069 | 9263342 |
| Gh_D05G2622 | chr19 | CPK11 | Calcium-dependent protein kinase 11 | 27,103,112 | 27,107,994 | + | PF00069 | 27105553 |
| Gh_A11G2941 | chr11 | CPK13 | Calcium-dependent protein kinase 13 | 93,261,063 | 93,265,206 | + | PF00069 | 93263135 |
| Gh_D04G1271 | chr22 | CPK13 | Calcium-dependent protein kinase 13 | 41,779,274 | 41,784,032 | - | PF00069 | 41781653 |
| Gh_A01G1094 | chr01 | CPK14 | Calcium-dependent protein kinase 14 | 41,868,709 | 41,875,773 | - | PF00069 | 41872241 |
| Gh_A07G1099 | chr07 | CPK17 | Calcium-dependent protein kinase 17 | 22,889,554 | 22,891,905 | - | PF00069 | 22890730 |
| Gh_A09G1033 | chr09 | CPK17 | Calcium-dependent protein kinase 17 | 60,455,043 | 60,457,397 | - | PF00069 | 60456220 |
| Gh_D05G0748 | chr19 | CPK17 | Calcium-dependent protein kinase 17 | 6,137,190 | 6,139,492 | + | PF00069 | 6138341 |
| Gh_D07G1198 | chr16 | CPK17 | Calcium-dependent protein kinase 17 | 18,231,735 | 18,234,096 | - | PF00069 | 18232916 |
| Gh_D08G0142 | chr24 | CPK17 | Calcium-dependent protein kinase 17 | 1,197,380 | 1,200,506 | - | PF00069 | 1198943 |
| Gh_D09G1054 | chr23 | CPK17 | Calcium-dependent protein kinase 17 | 36,618,092 | 36,620,409 | - | PF00069 | 36619251 |
| Gh_D09G1835 | chr23 | CPK17 | Calcium-dependent protein kinase 17 | 45,937,215 | 45,939,002 | - | PF00069 | 45938109 |
| Gh_D13G2278 | chr18 | CPK17 | Calcium-dependent protein kinase 17 | 58,814,168 | 58,818,344 | + | PF00069 | 58816256 |
| Gh_A01G1119 | chr01 | CPK2 | Calcium-dependent protein kinase 2 | 45,532,122 | 45,534,741 | + | PF00069 | 45533432 |
| Gh_A09G1248 | chr09 | CPK2 | Calcium-dependent protein kinase 2 | 64,587,018 | 64,590,334 | - | PF00069 | 64588676 |
| Gh_D01G1194 | chr15 | CPK2 | Calcium-dependent protein kinase 2 | 27,904,260 | 27,906,874 | + | PF00069 | 27905567 |
| Gh_D04G0895 | chr22 | CPK2 | Calcium-dependent protein kinase 2 | 25,443,458 | 25,447,176 | + | PF00069 | 25445317 |
| Gh_D09G1249 | chr23 | CPK2 | Calcium-dependent protein kinase 2 | 39,552,322 | 39,555,623 | - | PF00069 | 39553973 |
| Gh_D04G0900 | chr22 | CPK20 | Calcium-dependent protein kinase 20 | 25,701,269 | 25,707,494 | - | PF00069 | 25704382 |
| Gh_A06G1772 | chr06 | CPK21 | Calcium-dependent protein kinase 21 | 102,737,441 | 102,739,595 | - | PF00069 | 1.03E+08 |
| Gh_A10G0124 | chr10 | CPK21 | Calcium-dependent protein kinase 21 | 1,022,813 | 1,026,173 | - | PF00069 | 1024493 |
| Gh_A10G0124 | chr10 | CPK21 | Calcium-dependent protein kinase 21 | 1,022,813 | 1,026,173 | - | PF00069 | 1024493 |
| Gh_D06G2206 | chr25 | CPK21 | Calcium-dependent protein kinase 21 | 63,778,915 | 63,781,070 | + | PF00069 | 63779993 |
| Gh_D10G0130 | chr20 | CPK21 | Calcium-dependent protein kinase 21 | 1,030,951 | 1,034,394 | - | PF00069 | 1032673 |
| Gh_A02G0144 | Chr02 | CPK24 | Calcium-dependent protein kinase 24 | 1,508,323 | 1,512,491 | - | PF00069 | 1510407 |
| Gh_A05G2859 | chr05 | CPK24 | Calcium-dependent protein kinase 24 | 65,056,518 | 65,058,712 | - | PF00069 | 65057615 |
| Gh_A10G1195 | chr10 | CPK24 | Calcium-dependent protein kinase 24 | 62,477,215 | 62,482,365 | + | PF00069 | 62479790 |
| Gh_A10G1195 | chr10 | CPK24 | Calcium-dependent protein kinase 24 | 62,477,215 | 62,482,365 | + | PF00069 | 62479790 |
| Gh_D02G0183 | chr14 | CPK24 | Calcium-dependent protein kinase 24 | 2,005,202 | 2,011,106 | - | PF00069 | 2008154 |
| Gh_D05G3156 | chr19 | CPK24 | Calcium-dependent protein kinase 24 | 47,939,538 | 47,941,747 | - | PF00069 | 47940643 |
| Gh_D10G1303 | chr20 | CPK24 | Calcium-dependent protein kinase 24 | 24,177,574 | 24,181,997 | - | PF00069 | 24179786 |
| Gh_A02G1635 | Chr02 | CPK28 | Calcium-dependent protein kinase 28 | 82,802,954 | 82,807,746 | + | PF00069 | 82805350 |
| Gh_A10G0886 | chr10 | CPK28 | Calcium-dependent protein kinase 28 | 19,193,297 | 19,197,076 | + | PF00069 | 19195187 |
| Gh_A10G0886 | chr10 | CPK28 | Calcium-dependent protein kinase 28 | 19,193,297 | 19,197,076 | + | PF00069 | 19195187 |
| Gh_A11G1615 | chr11 | CPK28 | Calcium-dependent protein kinase 28 | 23,975,683 | 23,979,976 | - | PF00069 | 23977830 |
| Gh_D03G0087 | chr17 | CPK28 | Calcium-dependent protein kinase 28 | 613,069 | 617,844 | - | PF00069 | 615456.5 |
| Gh_D10G0863 | chr20 | CPK28 | Calcium-dependent protein kinase 28 | 10,909,734 | 10,913,532 | - | PF00069 | 10911633 |
| Gh_D11G1774 | chr21 | CPK28 | Calcium-dependent protein kinase 28 | 19,767,240 | 19,771,544 | - | PF00069 | 19769392 |
| Gh_A05G1571 | chr05 | CPK29 | Calcium-dependent protein kinase 29 | 16,092,204 | 16,102,107 | - | PF00069 | 16097156 |
| Gh_D05G1749 | chr19 | CPK29 | Calcium-dependent protein kinase 29 | 15,794,186 | 15,800,643 | - | PF00069 | 15797415 |
| Gh_A02G1029 | Chr02 | CPK3 | Calcium-dependent protein kinase 3 | 45,630,623 | 45,633,866 | + | PF00069 | 45632245 |
| Gh_A11G0213 | chr11 | CPK3 | Calcium-dependent protein kinase 3 | 2,006,974 | 2,009,900 | - | PF00069 | 2008437 |
| Gh_D05G2284 | chr19 | CPK3 | Calcium-dependent protein kinase 3 | 22,132,208 | 22,132,733 | - | PF00069 | 22132471 |
| Gh_D11G0227 | chr21 | CPK3 | Calcium-dependent protein kinase 3 | 1,905,499 | 1,908,419 | - | PF00069 | 1906959 |
| Gh_A09G1157 | chr09 | CPK32 | Calcium-dependent protein kinase 32 | 62,989,834 | 62,997,485 | - | PF00069 | 62993660 |
| Gh_D03G0609 | chr17 | CPK32 | Calcium-dependent protein kinase 32 | 15,802,257 | 15,805,491 | + | PF00069 | 15803874 |
| Gh_D09G1163 | chr23 | CPK32 | Calcium-dependent protein kinase 32 | 38,278,169 | 38,281,037 | - | PF00069 | 38279603 |
| Gh_A09G1722 | chr09 | CPK34 | Calcium-dependent protein kinase 34 | 70,654,787 | 70,656,580 | - | PF00069 | 70655684 |
| Gh_A12G2360 | chr12 | CPK34 | Calcium-dependent protein kinase 34 | 86,133,164 | 86,135,364 | - | PF00069 | 86134264 |
| Gh_D12G2497 | chr26 | CPK34 | Calcium-dependent protein kinase 34 | 57,868,861 | 57,871,071 | - | PF00069 | 57869966 |
| Gh_A03G1505 | chr03 | CPK4 | Calcium-dependent protein kinase 4 | 95,507,304 | 95,510,335 | - | PF00069 | 95508820 |
| Gh_A12G0109 | chr12 | CPK4 | Calcium-dependent protein kinase 4 | 1,542,851 | 1,545,587 | - | PF00069 | 1544219 |
| Gh_A13G0563 | chr13 | CPK4 | Calcium-dependent protein kinase 4 | 13,153,568 | 13,156,341 | - | PF00069 | 13154955 |
| Gh_A13G0566 | chr13 | CPK4 | Calcium-dependent protein kinase 4 | 13,367,845 | 13,370,645 | - | PF00069 | 13369245 |
| Gh_D02G1973 | chr14 | CPK4 | Calcium-dependent protein kinase 4 | 63,684,341 | 63,687,392 | - | PF00069 | 63685867 |
| Gh_D04G1486 | chr22 | CPK4 | Calcium-dependent protein kinase 4 | 46,703,911 | 46,706,311 | - | PF00069 | 46705111 |
| Gh_D12G0121 | chr26 | CPK4 | Calcium-dependent protein kinase 4 | 1,591,928 | 1,594,742 | - | PF00069 | 1593335 |
| Gh_D13G0560 | chr18 | CPK4 | Calcium-dependent protein kinase 4 | 7,586,741 | 7,589,365 | - | PF00069 | 7588053 |
| Gh_D13G0561 | chr18 | CPK4 | Calcium-dependent protein kinase 4 | 7,614,829 | 7,617,628 | - | PF00069 | 7616229 |
| Gh_A09G1067 | chr09 | CPK7 | Calcium-dependent protein kinase 7 | 61,271,009 | 61,274,418 | - | PF00069 | 61272714 |
| Gh_D09G1074 | chr23 | CPK7 | Calcium-dependent protein kinase 7 | 36,952,932 | 36,956,372 | - | PF00069 | 36954652 |
| Gh_A07G1123 | chr07 | CPK8 | Calcium-dependent protein kinase 8 | 23,758,317 | 23,762,270 | - | PF00069 | 23760294 |
| Gh_A13G1041 | chr13 | CPK8 | Calcium-dependent protein kinase 8 | 58,751,797 | 58,754,612 | - | PF00069 | 58753205 |
| Gh_D07G1228 | chr16 | CPK8 | Calcium-dependent protein kinase 8 | 18,817,674 | 18,820,435 | - | PF00069 | 18819055 |
| Gh_A01G0621 | chr01 | CPK9 | Calcium-dependent protein kinase 9 | 11,154,745 | 11,157,809 | + | PF00069 | 11156277 |
| Gh_A04G0148 | chr04 | CPK9 | Calcium-dependent protein kinase 9 | 2,396,366 | 2,399,478 | - | PF00069 | 2397922 |
| Gh_A13G1164 | chr13 | CPK9 | Calcium-dependent protein kinase 9 | 64,133,647 | 64,137,746 | - | PF00069 | 64135697 |
| Gh_D05G3567 | chr19 | CPK9 | Calcium-dependent protein kinase 9 | 59,026,817 | 59,029,919 | + | PF00069 | 59028368 |
| Gh_D13G1455 | chr18 | CPK9 | Calcium-dependent protein kinase 9 | 45,574,159 | 45,578,015 | + | PF00069 | 45576087 |
| Gh_A05G3246 | chr05 | NA | Calcium-dependent protein kinase SK5 | 84,817,227 | 84,822,268 | - | PF00069 | 84819748 |
| Gh_D04G0366 | chr22 | NA | Calcium-dependent protein kinase SK5 | 5,618,562 | 5,627,848 | + | PF00069 | 5623205 |
| Gh_A07G0932 | chr07 | CRCK1 | Calmodulin-binding receptor-like cytoplasmic kinase 1 | 16,932,611 | 16,939,535 | - | PF00069 | 16936073 |
| Gh_D07G1010 | chr16 | CRCK1 | Calmodulin-binding receptor-like cytoplasmic kinase 1 | 13,830,083 | 13,833,558 | - | PF00069 | 13831821 |
| Gh_A07G1304 | chr07 | CRCK2 | Calmodulin-binding receptor-like cytoplasmic kinase 2 | 31,164,819 | 31,167,032 | - | PF00069 | 31165926 |
| Gh_D07G1415 | chr16 | CRCK2 | Calmodulin-binding receptor-like cytoplasmic kinase 2 | 23,513,497 | 23,519,094 | + | PF00069 | 23516296 |
| Gh_D11G0955 | chr21 | CRCK2 | Calmodulin-binding receptor-like cytoplasmic kinase 2 | 8,296,358 | 8,298,530 | + | PF00069 | 8297444 |
| Gh_A08G0110 | chr08 | NA | Casein kinase I | 925,654 | 939,192 | - | PF00069 | 932423 |
| Gh_A13G1646 | chr13 | NA | Casein kinase I | 75,385,706 | 75,389,484 | + | PF00069 | 75387595 |
| Gh_D05G0476 | chr19 | NA | Casein kinase I | 3,809,340 | 3,813,650 | - | PF00069 | 3811495 |
| Gh_D08G0154 | chr24 | NA | Casein kinase I | 1,390,931 | 1,400,680 | - | PF00069 | 1395806 |
| Gh_D10G0542 | chr20 | NA | Casein kinase I | 5,281,295 | 5,291,152 | - | PF00069 | 5286224 |
| Gh_A10G0586 | chr10 | cki2 | Casein kinase I homolog 2 | 7,708,440 | 7,711,795 | - | PF00069 | 7710118 |
| Gh_A10G0586 | chr10 | cki2 | Casein kinase I homolog 2 | 7,708,440 | 7,711,795 | - | PF00069 | 7710118 |
| Gh_D08G0155 | chr24 | cki2 | Casein kinase I homolog 2 | 1,401,978 | 1,407,461 | - | PF00069 | 1404720 |
| Gh_D08G2507 | chr24 | cki2 | Casein kinase I homolog 2 | 64,854,282 | 64,859,703 | - | PF00069 | 64856993 |
| Gh_D13G2009 | chr18 | cki2 | Casein kinase I homolog 2 | 55,539,595 | 55,543,286 | + | PF00069 | 55541441 |
| Gh_A11G2352 | chr11 | kin-19 | Casein kinase I isoform alpha | 80,488,087 | 80,494,526 | + | PF00069 | 80491307 |
| Gh_A13G0876 | chr13 | kin-19 | Casein kinase I isoform alpha | 45,566,320 | 45,571,154 | + | PF00069 | 45568737 |
| Gh_D05G0222 | chr19 | csnk1a1 | Casein kinase I isoform alpha | 2,053,381 | 2,057,671 | - | PF00069 | 2055526 |
| Gh_D10G0631 | chr20 | kin-19 | Casein kinase I isoform alpha | 6,772,888 | 6,778,652 | - | PF00069 | 6775770 |
| Gh_D10G1159 | chr20 | CSNK1A1 | Casein kinase I isoform alpha | 19,323,611 | 19,323,946 | + | PF00069 | 19323779 |
| Gh_D11G2665 | chr21 | kin-19 | Casein kinase I isoform alpha | 55,383,992 | 55,389,055 | + | PF00069 | 55386524 |
| Gh_D07G1214 | chr16 | CSNK1B | Casein kinase I isoform beta | 18,534,993 | 18,535,442 | + | PF00069 | 18535218 |
| Gh_A08G2140 | chr08 | CSNK1D | Casein kinase I isoform delta | 102,536,592 | 102,541,891 | - | PF00069 | 1.03E+08 |
| Gh_A13G1511 | chr13 | CSNK1D | Casein kinase I isoform delta | 73,629,090 | 73,633,903 | + | PF00069 | 73631497 |
| Gh_D13G1118 | chr18 | CSNK1D | Casein kinase I isoform delta | 32,988,800 | 33,000,291 | + | PF00069 | 32994546 |
| Gh_D13G1841 | chr18 | CSNK1D | Casein kinase I isoform delta | 53,517,870 | 53,522,646 | + | PF00069 | 53520258 |
| Gh_A02G0391 | Chr02 | At4g26100 | Casein kinase I isoform delta-like | 4,980,237 | 4,984,157 | - | PF00069 | 4982197 |
| Gh_A05G1088 | chr05 | At4g26100 | Casein kinase I isoform delta-like | 11,029,978 | 11,036,866 | + | PF00069 | 11033422 |
| Gh_A05G1130 | chr05 | At4g26100 | Casein kinase I isoform delta-like | 11,473,782 | 11,478,069 | + | PF00069 | 11475926 |
| Gh_A06G0560 | chr06 | At4g26100 | Casein kinase I isoform delta-like | 14,420,838 | 14,424,612 | + | PF00069 | 14422725 |
| Gh_A07G0121 | chr07 | At4g26100 | Casein kinase I isoform delta-like | 1,434,048 | 1,438,559 | - | PF00069 | 1436304 |
| Gh_A07G1408 | chr07 | At4g26100 | Casein kinase I isoform delta-like | 38,992,841 | 38,997,693 | + | PF00069 | 38995267 |
| Gh_A07G1952 | chr07 | At4g26100 | Casein kinase I isoform delta-like | 75,361,944 | 75,366,132 | + | PF00069 | 75364038 |
| Gh_A08G1695 | chr08 | At4g26100 | Casein kinase I isoform delta-like | 96,678,364 | 96,683,226 | + | PF00069 | 96680795 |
| Gh_A08G1934 | chr08 | At4g26100 | Casein kinase I isoform delta-like | 100,182,242 | 100,187,749 | + | PF00069 | 1E+08 |
| Gh_A08G2241 | chr08 | At4g26100 | Casein kinase I isoform delta-like | 103,287,700 | 103,291,262 | - | PF00069 | 1.03E+08 |
| Gh_A09G2005 | chr09 | At4g26100 | Casein kinase I isoform delta-like | 73,243,804 | 73,248,023 | + | PF00069 | 73245914 |
| Gh_A10G0993 | chr10 | At4g26100 | Casein kinase I isoform delta-like | 26,473,800 | 26,479,205 | - | PF00069 | 26476503 |
| Gh_A10G0993 | chr10 | At4g26100 | Casein kinase I isoform delta-like | 26,473,800 | 26,479,205 | - | PF00069 | 26476503 |
| Gh_A11G1211 | chr11 | At4g26100 | Casein kinase I isoform delta-like | 15,040,319 | 15,045,649 | + | PF00069 | 15042984 |
| Gh_A11G2235 | chr11 | At4g26100 | Casein kinase I isoform delta-like | 76,875,437 | 76,880,562 | - | PF00069 | 76878000 |
| Gh_A11G2652 | chr11 | At4g26100 | Casein kinase I isoform delta-like | 88,153,652 | 88,158,624 | - | PF00069 | 88156138 |
| Gh_A13G1711 | chr13 | At4g26100 | Casein kinase I isoform delta-like | 76,035,747 | 76,040,698 | - | PF00069 | 76038223 |
| Gh_D02G0444 | chr14 | At4g26100 | Casein kinase I isoform delta-like | 5,827,804 | 5,831,718 | - | PF00069 | 5829761 |
| Gh_D05G0310 | chr19 | At4g26100 | Casein kinase I isoform delta-like | 2,677,715 | 2,682,089 | + | PF00069 | 2679902 |
| Gh_D05G1267 | chr19 | At4g26100 | Casein kinase I isoform delta-like | 10,889,875 | 10,897,358 | + | PF00069 | 10893617 |
| Gh_D05G1301 | chr19 | At4g26100 | Casein kinase I isoform delta-like | 11,497,894 | 11,502,176 | + | PF00069 | 11500035 |
| Gh_D06G0632 | chr25 | At4g26100 | Casein kinase I isoform delta-like | 10,786,736 | 10,790,513 | + | PF00069 | 10788625 |
| Gh_D07G0215 | chr16 | At4g26100 | Casein kinase I isoform delta-like | 2,248,895 | 2,255,632 | - | PF00069 | 2252264 |
| Gh_D07G1506 | chr16 | At4g26100 | Casein kinase I isoform delta-like | 27,106,253 | 27,111,202 | + | PF00069 | 27108728 |
| Gh_D07G2169 | chr16 | At4g26100 | Casein kinase I isoform delta-like | 52,220,468 | 52,224,642 | + | PF00069 | 52222555 |
| Gh_D07G2179 | chr16 | At4g26100 | Casein kinase I isoform delta-like | 52,373,757 | 52,375,665 | - | PF00069 | 52374711 |
| Gh_D08G2053 | chr24 | At4g26100 | Casein kinase I isoform delta-like | 59,215,675 | 59,220,493 | + | PF00069 | 59218084 |
| Gh_D08G2607 | chr24 | At4g26100 | Casein kinase I isoform delta-like | 65,664,372 | 65,667,948 | - | PF00069 | 65666160 |
| Gh_D09G2218 | chr23 | At4g26100 | Casein kinase I isoform delta-like | 49,380,577 | 49,384,739 | + | PF00069 | 49382658 |
| Gh_D11G2540 | chr21 | At4g26100 | Casein kinase I isoform delta-like | 52,376,694 | 52,381,850 | - | PF00069 | 52379272 |
| Gh_D13G2059 | chr18 | At4g26100 | Casein kinase I isoform delta-like | 56,111,391 | 56,116,348 | - | PF00069 | 56113870 |
| Gh_A02G1463 | Chr02 | At2g23070 | Casein kinase II subunit alpha, chloroplastic | 80,725,709 | 80,738,929 | - | PF00069 | 80732319 |
| Gh_A11G1960 | chr11 | At2g23070 | Casein kinase II subunit alpha, chloroplastic | 54,532,917 | 54,537,894 | - | PF00069 | 54535406 |
| Gh_D03G0248 | chr17 | At2g23070 | Casein kinase II subunit alpha, chloroplastic | 2,626,244 | 2,635,366 | + | PF00069 | 2630805 |
| Gh_D03G0249 | chr17 | At2g23070 | Casein kinase II subunit alpha, chloroplastic | 2,638,001 | 2,642,195 | + | PF00069 | 2640098 |
| Gh_D11G2015 | chr21 | At2g23070 | Casein kinase II subunit alpha, chloroplastic | 27,020,639 | 27,025,682 | + | PF00069 | 27023161 |
| Gh_A10G0812 | chr10 | CKA1 | Casein kinase II subunit alpha-1 | 16,669,531 | 16,673,227 | + | PF00069 | 16671379 |
| Gh_A10G0812 | chr10 | CKA1 | Casein kinase II subunit alpha-1 | 16,669,531 | 16,673,227 | + | PF00069 | 16671379 |
| Gh_A11G0445 | chr11 | CKA1 | Casein kinase II subunit alpha-1 | 4,198,944 | 4,202,197 | - | PF00069 | 4200571 |
| Gh_A12G0692 | chr12 | CKA1 | Casein kinase II subunit alpha-1 | 26,738,526 | 26,741,885 | - | PF00069 | 26740206 |
| Gh_D11G0516 | chr21 | CKA1 | Casein kinase II subunit alpha-1 | 4,485,549 | 4,488,807 | - | PF00069 | 4487178 |
| Gh_D12G0696 | chr26 | CKA1 | Casein kinase II subunit alpha-1 | 15,184,386 | 15,187,641 | - | PF00069 | 15186014 |
| Gh_D10G0949 | chr20 | CKA2 | Casein kinase II subunit alpha-2 | 12,695,088 | 12,698,795 | - | PF00069 | 12696942 |
| Gh_A05G2394 | chr05 | CIPK18 | CBL-interacting protein kinase 18 | 29,707,386 | 29,708,711 | - | PF00069 | 29708049 |
| Gh_A10G1618 | chr10 | CIPK18 | CBL-interacting protein kinase 18 | 87,175,695 | 87,176,243 | - | PF00069 | 87175969 |
| Gh_A10G1618 | chr10 | CIPK18 | CBL-interacting protein kinase 18 | 87,175,695 | 87,176,243 | - | PF00069 | 87175969 |
| Gh_D06G1928 | chr25 | CIPK18 | CBL-interacting protein kinase 18 | 60,407,459 | 60,408,769 | + | PF00069 | 60408114 |
| Gh_D10G1874 | chr20 | CIPK18 | CBL-interacting protein kinase 18 | 52,509,685 | 52,511,004 | - | PF00069 | 52510345 |
| Gh_A02G0047 | Chr02 | CIPK2 | CBL-interacting protein kinase 2 | 312,633 | 313,985 | - | PF00069 | 313309 |
| Gh_A06G1572 | chr06 | CIPK2 | CBL-interacting protein kinase 2 | 100,273,728 | 100,275,035 | + | PF00069 | 1E+08 |
| Gh_D02G0060 | chr14 | CIPK2 | CBL-interacting protein kinase 2 | 353,473 | 354,825 | - | PF00069 | 354149 |
| Gh_D05G2659 | chr19 | CIPK2 | CBL-interacting protein kinase 2 | 27,762,148 | 27,763,473 | - | PF00069 | 27762811 |
| Gh_A11G1462 | chr11 | CIPK23 | CBL-interacting protein kinase 23 | 20,161,023 | 20,161,502 | + | PF00069 | 20161263 |
| Gh_A06G1159 | chr06 | CIPK5 | CBL-interacting protein kinase 5 | 79,405,748 | 79,407,070 | + | PF00069 | 79406409 |
| Gh_D06G1440 | chr25 | CIPK5 | CBL-interacting protein kinase 5 | 46,716,902 | 46,718,224 | + | PF00069 | 46717563 |
| Gh_A06G1594 | chr06 | CIPK1 | CBL-interacting serine/threonine-protein kinase 1 | 100,603,107 | 100,608,690 | + | PF00069 | 1.01E+08 |
| Gh_A09G0653 | chr09 | CIPK1 | CBL-interacting serine/threonine-protein kinase 1 | 49,820,229 | 49,825,799 | - | PF00069 | 49823014 |
| Gh_D06G1948 | chr25 | CIPK1 | CBL-interacting serine/threonine-protein kinase 1 | 60,658,830 | 60,664,748 | + | PF00069 | 60661789 |
| Gh_D09G0655 | chr23 | CIPK1 | CBL-interacting serine/threonine-protein kinase 1 | 29,791,626 | 29,797,615 | - | PF00069 | 29794621 |
| Gh_A03G0196 | chr03 | CIPK10 | CBL-interacting serine/threonine-protein kinase 10 | 2,928,617 | 2,930,047 | - | PF00069 | 2929332 |
| Gh_D03G1385 | chr17 | CIPK10 | CBL-interacting serine/threonine-protein kinase 10 | 42,505,673 | 42,507,103 | + | PF00069 | 42506388 |
| Gh_A03G0193 | chr03 | CIPK11 | CBL-interacting serine/threonine-protein kinase 11 | 2,913,594 | 2,914,817 | + | PF00069 | 2914206 |
| Gh_A10G1323 | chr10 | CIPK11 | CBL-interacting serine/threonine-protein kinase 11 | 70,179,037 | 70,180,320 | - | PF00069 | 70179679 |
| Gh_A10G1323 | chr10 | CIPK11 | CBL-interacting serine/threonine-protein kinase 11 | 70,179,037 | 70,180,320 | - | PF00069 | 70179679 |
| Gh_D03G1388 | chr17 | CIPK11 | CBL-interacting serine/threonine-protein kinase 11 | 42,540,460 | 42,541,683 | - | PF00069 | 42541072 |
| Gh_D10G1152 | chr20 | CIPK11 | CBL-interacting serine/threonine-protein kinase 11 | 19,115,407 | 19,116,690 | + | PF00069 | 19116049 |
| Gh_A05G1293 | chr05 | CIPK12 | CBL-interacting serine/threonine-protein kinase 12 | 13,277,307 | 13,278,761 | + | PF00069 | 13278034 |
| Gh_A06G1160 | chr06 | CIPK12 | CBL-interacting serine/threonine-protein kinase 12 | 79,738,843 | 79,740,282 | - | PF00069 | 79739563 |
| Gh_A10G0336 | chr10 | CIPK12 | CBL-interacting serine/threonine-protein kinase 12 | 3,063,903 | 3,065,339 | + | PF00069 | 3064621 |
| Gh_A10G0336 | chr10 | CIPK12 | CBL-interacting serine/threonine-protein kinase 12 | 3,063,903 | 3,065,339 | + | PF00069 | 3064621 |
| Gh_D05G1464 | chr19 | CIPK12 | CBL-interacting serine/threonine-protein kinase 12 | 13,036,106 | 13,037,542 | + | PF00069 | 13036824 |
| Gh_D06G1441 | chr25 | CIPK12 | CBL-interacting serine/threonine-protein kinase 12 | 46,855,259 | 46,856,698 | - | PF00069 | 46855979 |
| Gh_D10G0342 | chr20 | CIPK12 | CBL-interacting serine/threonine-protein kinase 12 | 2,942,040 | 2,943,476 | + | PF00069 | 2942758 |
| Gh_A02G0045 | Chr02 | CIPK14 | CBL-interacting serine/threonine-protein kinase 14 | 301,702 | 302,994 | + | PF00069 | 302348 |
| Gh_D02G0058 | chr14 | CIPK14 | CBL-interacting serine/threonine-protein kinase 14 | 341,646 | 342,938 | + | PF00069 | 342292 |
| Gh_A09G0711 | chr09 | CIPK20 | CBL-interacting serine/threonine-protein kinase 20 | 52,830,264 | 52,833,420 | + | PF00069 | 52831842 |
| Gh_D06G2143 | chr25 | CIPK20 | CBL-interacting serine/threonine-protein kinase 20 | 63,267,674 | 63,269,062 | - | PF00069 | 63268368 |
| Gh_A08G0773 | chr08 | CIPK21 | CBL-interacting serine/threonine-protein kinase 21 | 28,968,573 | 28,971,101 | - | PF00069 | 28969837 |
| Gh_D08G0921 | chr24 | CIPK21 | CBL-interacting serine/threonine-protein kinase 21 | 19,312,291 | 19,314,817 | - | PF00069 | 19313554 |
| Gh_A06G1219 | chr06 | CIPK23 | CBL-interacting serine/threonine-protein kinase 23 | 88,102,737 | 88,108,568 | + | PF00069 | 88105653 |
| Gh_A09G0738 | chr09 | CIPK23 | CBL-interacting serine/threonine-protein kinase 23 | 53,291,797 | 53,298,835 | - | PF00069 | 53295316 |
| Gh_A10G0406 | chr10 | CIPK23 | CBL-interacting serine/threonine-protein kinase 23 | 4,036,396 | 4,041,446 | + | PF00069 | 4038921 |
| Gh_A10G0406 | chr10 | CIPK23 | CBL-interacting serine/threonine-protein kinase 23 | 4,036,396 | 4,041,446 | + | PF00069 | 4038921 |
| Gh_D06G1517 | chr25 | CIPK23 | CBL-interacting serine/threonine-protein kinase 23 | 50,429,582 | 50,444,707 | + | PF00069 | 50437145 |
| Gh_D09G0739 | chr23 | CIPK23 | CBL-interacting serine/threonine-protein kinase 23 | 31,547,152 | 31,553,826 | - | PF00069 | 31550489 |
| Gh_D10G0421 | chr20 | CIPK23 | CBL-interacting serine/threonine-protein kinase 23 | 3,871,538 | 3,876,571 | + | PF00069 | 3874055 |
| Gh_A06G0475 | chr06 | CIPK24 | CBL-interacting serine/threonine-protein kinase 24 | 9,107,511 | 9,116,036 | + | PF00069 | 9111774 |
| Gh_D06G0521 | chr25 | CIPK24 | CBL-interacting serine/threonine-protein kinase 24 | 7,915,286 | 7,922,101 | + | PF00069 | 7918694 |
| Gh_A05G1020 | chr05 | CIPK25 | CBL-interacting serine/threonine-protein kinase 25 | 10,256,855 | 10,258,201 | + | PF00069 | 10257528 |
| Gh_A06G0919 | chr06 | CIPK25 | CBL-interacting serine/threonine-protein kinase 25 | 36,770,365 | 36,771,714 | - | PF00069 | 36771040 |
| Gh_D05G1138 | chr19 | CIPK25 | CBL-interacting serine/threonine-protein kinase 25 | 9,767,303 | 9,768,649 | + | PF00069 | 9767976 |
| Gh_D06G1080 | chr25 | CIPK25 | CBL-interacting serine/threonine-protein kinase 25 | 24,075,242 | 24,076,591 | - | PF00069 | 24075917 |
| Gh_A02G0872 | Chr02 | CIPK3 | CBL-interacting serine/threonine-protein kinase 3 | 26,580,897 | 26,582,742 | - | PF00069 | 26581820 |
| Gh_A07G1075 | chr07 | CIPK3 | CBL-interacting serine/threonine-protein kinase 3 | 21,797,067 | 21,803,253 | - | PF00069 | 21800160 |
| Gh_A09G1004 | chr09 | CIPK3 | CBL-interacting serine/threonine-protein kinase 3 | 59,966,369 | 59,969,961 | + | PF00069 | 59968165 |
| Gh_D05G0732 | chr19 | CIPK3 | CBL-interacting serine/threonine-protein kinase 3 | 6,018,959 | 6,023,612 | + | PF00069 | 6021286 |
| Gh_D07G1173 | chr16 | CIPK3 | CBL-interacting serine/threonine-protein kinase 3 | 17,555,863 | 17,562,067 | - | PF00069 | 17558965 |
| Gh_D09G1024 | chr23 | CIPK3 | CBL-interacting serine/threonine-protein kinase 3 | 36,223,845 | 36,227,746 | + | PF00069 | 36225796 |
| Gh_A07G0260 | chr07 | CIPK5 | CBL-interacting serine/threonine-protein kinase 5 | 3,230,637 | 3,231,977 | + | PF00069 | 3231307 |
| Gh_D05G0599 | chr19 | CIPK5 | CBL-interacting serine/threonine-protein kinase 5 | 4,865,742 | 4,867,094 | + | PF00069 | 4866418 |
| Gh_D07G0316 | chr16 | CIPK5 | CBL-interacting serine/threonine-protein kinase 5 | 3,341,270 | 3,342,616 | + | PF00069 | 3341943 |
| Gh_A06G0873 | chr06 | CIPK6 | CBL-interacting serine/threonine-protein kinase 6 | 33,360,018 | 33,361,313 | - | PF00069 | 33360666 |
| Gh_A07G0210 | chr07 | CIPK6 | CBL-interacting serine/threonine-protein kinase 6 | 2,565,789 | 2,567,287 | - | PF00069 | 2566538 |
| Gh_A13G1623 | chr13 | CIPK6 | CBL-interacting serine/threonine-protein kinase 6 | 75,062,402 | 75,063,914 | + | PF00069 | 75063158 |
| Gh_D05G0536 | chr19 | CIPK6 | CBL-interacting serine/threonine-protein kinase 6 | 4,357,435 | 4,358,998 | - | PF00069 | 4358217 |
| Gh_D06G1020 | chr25 | CIPK6 | CBL-interacting serine/threonine-protein kinase 6 | 21,731,900 | 21,733,431 | - | PF00069 | 21732666 |
| Gh_D07G0265 | chr16 | CIPK6 | CBL-interacting serine/threonine-protein kinase 6 | 2,774,586 | 2,776,080 | - | PF00069 | 2775333 |
| Gh_D13G1983 | chr18 | CIPK6 | CBL-interacting serine/threonine-protein kinase 6 | 55,190,864 | 55,192,315 | + | PF00069 | 55191590 |
| Gh_A02G0339 | Chr02 | CIPK7 | CBL-interacting serine/threonine-protein kinase 7 | 4,015,866 | 4,017,254 | + | PF00069 | 4016560 |
| Gh_A09G1937 | chr09 | CIPK7 | CBL-interacting serine/threonine-protein kinase 7 | 72,542,163 | 72,543,539 | + | PF00069 | 72542851 |
| Gh_A10G1057 | chr10 | CIPK7 | CBL-interacting serine/threonine-protein kinase 7 | 44,474,286 | 44,475,635 | - | PF00069 | 44474961 |
| Gh_A10G1057 | chr10 | CIPK7 | CBL-interacting serine/threonine-protein kinase 7 | 44,474,286 | 44,475,635 | - | PF00069 | 44474961 |
| Gh_D02G0404 | chr14 | CIPK7 | CBL-interacting serine/threonine-protein kinase 7 | 5,207,702 | 5,209,090 | + | PF00069 | 5208396 |
| Gh_D09G2147 | chr23 | CIPK7 | CBL-interacting serine/threonine-protein kinase 7 | 48,631,845 | 48,633,221 | + | PF00069 | 48632533 |
| Gh_D10G1464 | chr20 | CIPK7 | CBL-interacting serine/threonine-protein kinase 7 | 33,008,242 | 33,009,564 | - | PF00069 | 33008903 |
| Gh_D05G2084 | chr19 | CIPK8 | CBL-interacting serine/threonine-protein kinase 8 | 19,377,503 | 19,381,107 | + | PF00069 | 19379305 |
| Gh_D06G0240 | chr25 | CIPK8 | CBL-interacting serine/threonine-protein kinase 8 | 2,550,394 | 2,553,958 | + | PF00069 | 2552176 |
| Gh_A01G1132 | chr01 | CIPK9 | CBL-interacting serine/threonine-protein kinase 9 | 46,963,639 | 46,969,960 | - | PF00069 | 46966800 |
| Gh_A02G0800 | Chr02 | CIPK9 | CBL-interacting serine/threonine-protein kinase 9 | 17,090,598 | 17,091,086 | + | PF00069 | 17090842 |
| Gh_A07G1228 | chr07 | CIPK9 | CBL-interacting serine/threonine-protein kinase 9 | 27,938,535 | 27,941,951 | - | PF00069 | 27940243 |
| Gh_A11G0911 | chr11 | CIPK9 | CBL-interacting serine/threonine-protein kinase 9 | 9,648,603 | 9,652,958 | + | PF00069 | 9650781 |
| Gh_A12G1043 | chr12 | CIPK9 | CBL-interacting serine/threonine-protein kinase 9 | 62,327,203 | 62,333,606 | + | PF00069 | 62330405 |
| Gh_D07G1332 | chr16 | CIPK9 | CBL-interacting serine/threonine-protein kinase 9 | 21,380,346 | 21,383,783 | - | PF00069 | 21382065 |
| Gh_D11G1058 | chr21 | CIPK9 | CBL-interacting serine/threonine-protein kinase 9 | 9,509,532 | 9,514,267 | + | PF00069 | 9511900 |
| Gh_A09G1081 | chr09 | CRK1 | CDPK-related kinase 1 | 61,548,414 | 61,553,893 | + | PF00069 | 61551154 |
| Gh_D09G1090 | chr23 | CRK1 | CDPK-related kinase 1 | 37,180,762 | 37,186,220 | + | PF00069 | 37183491 |
| Gh_A07G1236 | chr07 | CRK3 | CDPK-related kinase 3 | 28,306,900 | 28,325,724 | + | PF00069 | 28316312 |
| Gh_A11G0907 | chr11 | CRK3 | CDPK-related kinase 3 | 9,520,652 | 9,526,136 | - | PF00069 | 9523394 |
| Gh_D07G1343 | chr16 | CRK3 | CDPK-related kinase 3 | 21,612,640 | 21,619,006 | + | PF00069 | 21615823 |
| Gh_D11G1053 | chr21 | CRK3 | CDPK-related kinase 3 | 9,401,602 | 9,406,971 | - | PF00069 | 9404287 |
| Gh_A08G0912 | chr08 | CRK4 | CDPK-related kinase 4 | 56,874,763 | 56,878,815 | - | PF00069 | 56876789 |
| Gh_D08G1115 | chr24 | CRK4 | CDPK-related kinase 4 | 34,484,380 | 34,488,445 | - | PF00069 | 34486413 |
| Gh_A11G1762 | chr11 | CRK5 | CDPK-related kinase 5 | 29,388,815 | 29,393,076 | + | PF00069 | 29390946 |
| Gh_A12G0646 | chr12 | CRK5 | CDPK-related kinase 5 | 20,101,786 | 20,106,151 | + | PF00069 | 20103969 |
| Gh_A13G0338 | chr13 | CRK5 | CDPK-related kinase 5 | 4,378,253 | 4,383,193 | + | PF00069 | 4380723 |
| Gh_D11G1919 | chr21 | CRK5 | CDPK-related kinase 5 | 23,564,721 | 23,568,985 | + | PF00069 | 23566853 |
| Gh_D12G0848 | chr26 | CRK5 | CDPK-related kinase 5 | 27,440,935 | 27,445,272 | - | PF00069 | 27443104 |
| Gh_D13G0379 | chr18 | CRK5 | CDPK-related kinase 5 | 4,083,249 | 4,088,161 | + | PF00069 | 4085705 |
| Gh_A03G1690 | chr03 | CRK | CDPK-related protein kinase | 98,184,439 | 98,188,806 | + | PF00069 | 98186623 |
| Gh_D02G2110 | chr14 | CRK | CDPK-related protein kinase | 65,259,594 | 65,263,947 | + | PF00069 | 65261771 |
| Gh_A06G0979 | chr06 | CDC2A | Cell division control protein 2 homolog A | 45,744,003 | 45,748,850 | - | PF00069 | 45746427 |
| Gh_A13G0911 | chr13 | CDC2A | Cell division control protein 2 homolog A | 47,919,289 | 47,919,585 | - | PF00069 | 47919437 |
| Gh_D06G1153 | chr25 | CDC2A | Cell division control protein 2 homolog A | 26,981,507 | 26,985,215 | - | PF00069 | 26983361 |
| Gh_A05G3228 | chr05 | CDC2C | Cell division control protein 2 homolog C | 84,481,671 | 84,482,135 | - | PF00069 | 84481903 |
| Gh_A01G0813 | chr01 | CDC2D | Cell division control protein 2 homolog D | 18,461,866 | 18,463,408 | + | PF00069 | 18462637 |
| Gh_D01G0840 | chr15 | CDC2D | Cell division control protein 2 homolog D | 13,703,472 | 13,705,020 | + | PF00069 | 13704246 |
| Gh_D07G0829 | chr16 | cdc7 | Cell division control protein 7 | 10,550,225 | 10,551,928 | - | PF00069 | 10551077 |
| Gh_A09G0392 | chr09 | CDKB1-2 | Cyclin-dependent kinase B1-2 | 25,358,935 | 25,360,412 | + | PF00069 | 25359674 |
| Gh_D04G0378 | chr22 | CDKB1-2 | Cyclin-dependent kinase B1-2 | 5,886,575 | 5,888,627 | + | PF00069 | 5887601 |
| Gh_A08G1333 | chr08 | CDKC-1 | Cyclin-dependent kinase C-1 | 86,315,556 | 86,319,915 | - | PF00069 | 86317736 |
| Gh_D08G1628 | chr24 | CDKC-2 | Cyclin-dependent kinase C-2 | 51,109,124 | 51,113,530 | - | PF00069 | 51111327 |
| Gh_A09G0498 | chr09 | CDKD-1 | Cyclin-dependent kinase D-1 | 39,565,295 | 39,569,539 | - | PF00069 | 39567417 |
| Gh_A09G1581 | chr09 | CDKD-1 | Cyclin-dependent kinase D-1 | 69,169,679 | 69,177,142 | + | PF00069 | 69173411 |
| Gh_D09G0505 | chr23 | CDKD-1 | Cyclin-dependent kinase D-1 | 24,483,522 | 24,487,751 | - | PF00069 | 24485637 |
| Gh_D09G1668 | chr23 | CDKD-1 | Cyclin-dependent kinase D-1 | 44,329,951 | 44,337,731 | + | PF00069 | 44333841 |
| Gh_A09G1688 | chr09 | CDKE-1 | Cyclin-dependent kinase E-1 | 70,391,997 | 70,393,439 | + | PF00069 | 70392718 |
| Gh_A13G0098 | chr13 | CDKE-1 | Cyclin-dependent kinase E-1 | 1,179,217 | 1,180,641 | + | PF00069 | 1179929 |
| Gh_D09G1794 | chr23 | CDKE-1 | Cyclin-dependent kinase E-1 | 45,602,368 | 45,603,810 | + | PF00069 | 45603089 |
| Gh_D13G0113 | chr18 | CDKE-1 | Cyclin-dependent kinase E-1 | 1,148,889 | 1,150,328 | + | PF00069 | 1149609 |
| Gh_D05G0242 | chr19 | CDKF-1 | Cyclin-dependent kinase F-1 | 2,194,647 | 2,196,286 | - | PF00069 | 2195467 |
| Gh_A08G1357 | chr08 | CDKF-4 | Cyclin-dependent kinase F-4 | 87,774,325 | 87,778,918 | + | PF00069 | 87776622 |
| Gh_A12G1847 | chr12 | CDKF-4 | Cyclin-dependent kinase F-4 | 81,080,441 | 81,083,558 | + | PF00069 | 81082000 |
| Gh_D08G1653 | chr24 | CDKF-4 | Cyclin-dependent kinase F-4 | 52,031,672 | 52,034,687 | + | PF00069 | 52033180 |
| Gh_D12G2017 | chr26 | CDKF-4 | Cyclin-dependent kinase F-4 | 53,176,398 | 53,179,530 | + | PF00069 | 53177964 |
| Gh_A03G1115 | chr03 | CDKG-2 | Cyclin-dependent kinase G-2 | 81,001,450 | 81,004,726 | - | PF00069 | 81003088 |
| Gh_A07G0469 | chr07 | CDKG-2 | Cyclin-dependent kinase G-2 | 6,070,719 | 6,072,909 | - | PF00069 | 6071814 |
| Gh_A12G1705 | chr12 | CDKG-2 | Cyclin-dependent kinase G-2 | 78,774,164 | 78,776,378 | + | PF00069 | 78775271 |
| Gh_D02G1543 | chr14 | CDKG-2 | Cyclin-dependent kinase G-2 | 53,453,957 | 53,457,227 | - | PF00069 | 53455592 |
| Gh_D04G1812 | chr22 | CDKG-2 | Cyclin-dependent kinase G-2 | 50,433,225 | 50,436,586 | + | PF00069 | 50434906 |
| Gh_D07G0534 | chr16 | CDKG-2 | Cyclin-dependent kinase G-2 | 6,034,826 | 6,036,921 | - | PF00069 | 6035874 |
| Gh_D12G1867 | chr26 | CDKG-2 | Cyclin-dependent kinase G-2 | 51,280,460 | 51,282,677 | + | PF00069 | 51281569 |
| Gh_A06G1072 | chr06 | CRK10 | Cysteine-rich receptor-like protein kinase 10 | 64,176,972 | 64,182,934 | + | PF00069 | 64179953 |
| Gh_D10G1548 | chr20 | CRK29 | Cysteine-rich receptor-like protein kinase 29 | 40,612,373 | 40,613,598 | - | PF00069 | 40612986 |
| Gh_A07G1433 | chr07 | YAK1 | Dual specificity protein kinase YAK1 | 39,798,977 | 39,803,081 | - | PF00069 | 39801029 |
| Gh_A12G0025 | chr12 | bud32 | EKC/KEOPS complex subunit bud32 | 439,659 | 441,016 | + | PF00069 | 440337.5 |
| Gh_A09G0488 | chr09 | GDPDL2 | Glycerophosphodiester phosphodiesterase protein kinase domain-containing GDPDL2 | 38,843,052 | 38,845,481 | - | PF00069 | 38844267 |
| Gh_D05G2756 | chr19 | GDPDL2 | Glycerophosphodiester phosphodiesterase protein kinase domain-containing GDPDL2 | 29,877,321 | 29,879,925 | + | PF00069 | 29878623 |
| Gh_A12G1106 | chr12 | MSK-3 | Glycogen synthase kinase-3 homolog MsK-3 | 64,114,146 | 64,116,909 | + | PF00069 | 64115528 |
| Gh_D12G1230 | chr26 | MSK-3 | Glycogen synthase kinase-3 homolog MsK-3 | 40,170,076 | 40,172,846 | + | PF00069 | 40171461 |
| Gh_D09G1099 | chr23 | At1g34300 | G-type lectin S-receptor-like serine/threonine-protein kinase At1g34300 | 37,365,768 | 37,367,274 | + | PF00069 | 37366521 |
| Gh_A05G3348 | chr05 | RLK1 | G-type lectin S-receptor-like serine/threonine-protein kinase RLK1 | 87,482,056 | 87,482,463 | - | PF00069 | 87482260 |
| Gh_A05G3351 | chr05 | RLK1 | G-type lectin S-receptor-like serine/threonine-protein kinase RLK1 | 87,550,574 | 87,550,981 | - | PF00069 | 87550778 |
| Gh_A05G3354 | chr05 | RLK1 | G-type lectin S-receptor-like serine/threonine-protein kinase RLK1 | 87,747,100 | 87,747,507 | - | PF00069 | 87747304 |
| Gh_D04G0237 | chr22 | RLK1 | G-type lectin S-receptor-like serine/threonine-protein kinase RLK1 | 3,377,049 | 3,378,059 | + | PF00069 | 3377554 |
| Gh_D04G0244 | chr22 | RLK1 | G-type lectin S-receptor-like serine/threonine-protein kinase RLK1 | 3,640,379 | 3,640,786 | - | PF00069 | 3640583 |
| Gh_A10G1564 | chr10 | SD25 | G-type lectin S-receptor-like serine/threonine-protein kinase SD2-5 | 85,387,435 | 85,388,610 | + | PF00069 | 85388023 |
| Gh_A10G1564 | chr10 | SD25 | G-type lectin S-receptor-like serine/threonine-protein kinase SD2-5 | 85,387,435 | 85,388,610 | + | PF00069 | 85388023 |
| Gh_D10G0387 | chr20 | SD25 | G-type lectin S-receptor-like serine/threonine-protein kinase SD2-5 | 3,418,496 | 3,419,479 | - | PF00069 | 3418988 |
| Gh_D10G1819 | chr20 | SD25 | G-type lectin S-receptor-like serine/threonine-protein kinase SD2-5 | 51,156,988 | 51,158,473 | + | PF00069 | 51157731 |
| Gh_D10G1820 | chr20 | SD25 | G-type lectin S-receptor-like serine/threonine-protein kinase SD2-5 | 51,204,385 | 51,205,560 | + | PF00069 | 51204973 |
| Gh_D10G1848 | chr20 | SD25 | G-type lectin S-receptor-like serine/threonine-protein kinase SD2-5 | 51,647,671 | 51,648,852 | - | PF00069 | 51648262 |
| Gh_D04G1362 | chr22 | CRN | Inactive leucine-rich repeat receptor-like protein kinase CORYNE | 44,304,395 | 44,306,016 | + | PF00069 | 44305206 |
| Gh_D03G0055 | chr17 | At2g14510 | Leucine-rich repeat receptor-like serine/threonine-protein kinase At2g14510 | 351,234 | 353,411 | + | PF00069 | 352322.5 |
| Gh_D07G0658 | chr16 | FEI1 | LRR receptor-like serine/threonine-protein kinase FEI 1 | 7,672,698 | 7,674,939 | - | PF00069 | 7673819 |
| Gh_A13G0735 | chr13 | RPK2 | LRR receptor-like serine/threonine-protein kinase RPK2 | 25,336,017 | 25,337,133 | + | PF00069 | 25336575 |
| Gh_A09G1258 | chr09 | LECRK91 | L-type lectin-domain containing receptor kinase IX.1 | 64,672,290 | 64,672,952 | - | PF00069 | 64672621 |
| Gh_A10G0794 | chr10 | LECRK91 | L-type lectin-domain containing receptor kinase IX.1 | 16,256,010 | 16,256,684 | + | PF00069 | 16256347 |
| Gh_A10G0794 | chr10 | LECRK91 | L-type lectin-domain containing receptor kinase IX.1 | 16,256,010 | 16,256,684 | + | PF00069 | 16256347 |
| Gh_A11G1876 | chr11 | LECRK91 | L-type lectin-domain containing receptor kinase IX.1 | 48,110,507 | 48,111,579 | - | PF00069 | 48111043 |
| Gh_D04G1541 | chr22 | LECRK91 | L-type lectin-domain containing receptor kinase IX.1 | 47,508,157 | 47,512,162 | + | PF00069 | 47510160 |
| Gh_D04G1544 | chr22 | LECRK91 | L-type lectin-domain containing receptor kinase IX.1 | 47,545,867 | 47,547,399 | - | PF00069 | 47546633 |
| Gh_D09G1259 | chr23 | LECRK91 | L-type lectin-domain containing receptor kinase IX.1 | 39,667,272 | 39,667,934 | - | PF00069 | 39667603 |
| Gh_D11G0478 | chr21 | LYK4 | LysM domain receptor-like kinase 4 | 4,112,431 | 4,114,323 | + | PF00069 | 4113377 |
| Gh_A03G1448 | chr03 | M3KE1 | MAP3K epsilon protein kinase 1 | 94,380,604 | 94,409,827 | - | PF00069 | 94395216 |
| Gh_A12G1226 | chr12 | M3KE1 | MAP3K epsilon protein kinase 1 | 66,590,972 | 66,604,396 | + | PF00069 | 66597684 |
| Gh_D01G0148 | chr15 | M3KE1 | MAP3K epsilon protein kinase 1 | 1,074,731 | 1,086,830 | + | PF00069 | 1080781 |
| Gh_D02G1908 | chr14 | M3KE1 | MAP3K epsilon protein kinase 1 | 62,704,220 | 62,720,782 | - | PF00069 | 62712501 |
| Gh_D04G0447 | chr22 | M3KE1 | MAP3K epsilon protein kinase 1 | 7,253,714 | 7,255,866 | + | PF00069 | 7254790 |
| Gh_D09G0500 | chr23 | M3KE1 | MAP3K epsilon protein kinase 1 | 24,014,909 | 24,024,117 | - | PF00069 | 24019513 |
| Gh_D10G0722 | chr20 | M3KE1 | MAP3K epsilon protein kinase 1 | 8,350,980 | 8,352,536 | + | PF00069 | 8351758 |
| Gh_D12G1351 | chr26 | M3KE1 | MAP3K epsilon protein kinase 1 | 42,123,640 | 42,137,184 | + | PF00069 | 42130412 |
| Gh_A08G0488 | chr08 | TOR1 | Microtubule-associated protein TORTIFOLIA1 | 6,856,888 | 6,867,134 | - | PF00069 | 6862011 |
| Gh_A02G0240 | Chr02 | MPK16 | Mitogen-activated protein kinase 16 | 2,994,068 | 2,999,263 | + | PF00069 | 2996666 |
| Gh_D02G0309 | chr14 | MPK16 | Mitogen-activated protein kinase 16 | 4,215,787 | 4,220,996 | + | PF00069 | 4218392 |
| Gh_D01G1468 | chr15 | MPK17 | Mitogen-activated protein kinase 17 | 43,953,091 | 43,956,375 | + | PF00069 | 43954733 |
| Gh_D02G1456 | chr14 | MPK17 | Mitogen-activated protein kinase 17 | 50,153,272 | 50,156,248 | + | PF00069 | 50154760 |
| Gh_A01G0304 | chr01 | MPK19 | Mitogen-activated protein kinase 19 | 3,985,939 | 3,990,924 | + | PF00069 | 3988432 |
| Gh_D01G0338 | chr15 | MPK19 | Mitogen-activated protein kinase 19 | 3,681,534 | 3,686,591 | + | PF00069 | 3684063 |
| Gh_D05G3251 | chr19 | MPK19 | Mitogen-activated protein kinase 19 | 50,944,955 | 50,949,902 | + | PF00069 | 50947429 |
| Gh_D11G2977 | chr21 | MPK19 | Mitogen-activated protein kinase 19 | 60,595,513 | 60,599,309 | - | PF00069 | 60597411 |
| Gh_A05G3276 | chr05 | MPK20 | Mitogen-activated protein kinase 20 | 85,815,088 | 85,820,370 | - | PF00069 | 85817729 |
| Gh_A09G0065 | chr09 | MPK20 | Mitogen-activated protein kinase 20 | 1,410,983 | 1,425,240 | - | PF00069 | 1418112 |
| Gh_D09G0061 | chr23 | MPK20 | Mitogen-activated protein kinase 20 | 1,623,703 | 1,630,559 | - | PF00069 | 1627131 |
| Gh_A03G0291 | chr03 | MPK3 | Mitogen-activated protein kinase 3 | 4,812,676 | 4,815,197 | - | PF00069 | 4813937 |
| Gh_A05G0864 | chr05 | MPK3 | Mitogen-activated protein kinase 3 | 8,610,707 | 8,612,800 | + | PF00069 | 8611754 |
| Gh_D03G1283 | chr17 | MPK3 | Mitogen-activated protein kinase 3 | 40,842,880 | 40,845,406 | + | PF00069 | 40844143 |
| Gh_A03G0163 | chr03 | MPK4 | Mitogen-activated protein kinase 4 | 2,477,461 | 2,480,961 | - | PF00069 | 2479211 |
| Gh_A07G1297 | chr07 | MPK4 | Mitogen-activated protein kinase 4 | 30,788,888 | 30,792,208 | + | PF00069 | 30790548 |
| Gh_A07G1299 | chr07 | MPK4 | Mitogen-activated protein kinase 4 | 30,830,393 | 30,833,735 | + | PF00069 | 30832064 |
| Gh_A08G1183 | chr08 | MPK4 | Mitogen-activated protein kinase 4 | 81,815,347 | 81,818,130 | - | PF00069 | 81816739 |
| Gh_A12G0964 | chr12 | MPK4 | Mitogen-activated protein kinase 4 | 60,529,329 | 60,532,246 | + | PF00069 | 60530788 |
| Gh_D03G1422 | chr17 | MPK4 | Mitogen-activated protein kinase 4 | 42,988,477 | 42,991,949 | + | PF00069 | 42990213 |
| Gh_D07G1410 | chr16 | MPK4 | Mitogen-activated protein kinase 4 | 23,229,322 | 23,267,869 | + | PF00069 | 23248596 |
| Gh_D08G1467 | chr24 | MPK4 | Mitogen-activated protein kinase 4 | 47,809,956 | 47,812,698 | - | PF00069 | 47811327 |
| Gh_D12G1076 | chr26 | MPK4 | Mitogen-activated protein kinase 4 | 36,955,089 | 36,959,280 | + | PF00069 | 36957185 |
| Gh_A02G1605 | Chr02 | MPK7 | Mitogen-activated protein kinase 7 | 82,556,491 | 82,559,182 | + | PF00069 | 82557837 |
| Gh_A10G0867 | chr10 | MPK7 | Mitogen-activated protein kinase 7 | 18,812,916 | 18,815,114 | + | PF00069 | 18814015 |
| Gh_A10G0867 | chr10 | MPK7 | Mitogen-activated protein kinase 7 | 18,812,916 | 18,815,114 | + | PF00069 | 18814015 |
| Gh_A11G0401 | chr11 | MPK7 | Mitogen-activated protein kinase 7 | 3,724,025 | 3,726,118 | - | PF00069 | 3725072 |
| Gh_A12G0575 | chr12 | MPK7 | Mitogen-activated protein kinase 7 | 14,325,447 | 14,326,347 | - | PF00069 | 14325897 |
| Gh_A12G0576 | chr12 | MPK7 | Mitogen-activated protein kinase 7 | 14,326,376 | 14,327,853 | - | PF00069 | 14327115 |
| Gh_D03G0118 | chr17 | MPK7 | Mitogen-activated protein kinase 7 | 882,446 | 884,959 | - | PF00069 | 883702.5 |
| Gh_D10G0887 | chr20 | MPK7 | Mitogen-activated protein kinase 7 | 11,299,251 | 11,301,447 | - | PF00069 | 11300349 |
| Gh_D11G0464 | chr21 | MPK7 | Mitogen-activated protein kinase 7 | 3,969,416 | 3,971,510 | - | PF00069 | 3970463 |
| Gh_D12G0590 | chr26 | MPK7 | Mitogen-activated protein kinase 7 | 10,988,058 | 10,989,252 | - | PF00069 | 10988655 |
| Gh_A11G0035 | chr11 | MPK9 | Mitogen-activated protein kinase 9 | 370,389 | 376,385 | + | PF00069 | 373387 |
| Gh_D11G0033 | chr21 | MPK9 | Mitogen-activated protein kinase 9 | 309,838 | 315,813 | + | PF00069 | 312825.5 |
| Gh_D12G2548 | chr26 | MPK9 | Mitogen-activated protein kinase 9 | 58,340,597 | 58,345,504 | - | PF00069 | 58343051 |
| Gh_A02G0080 | Chr02 | MMK1 | Mitogen-activated protein kinase homolog MMK1 | 697,341 | 704,493 | + | PF00069 | 700917 |
| Gh_A10G1302 | chr10 | MMK1 | Mitogen-activated protein kinase homolog MMK1 | 67,409,834 | 67,413,136 | + | PF00069 | 67411485 |
| Gh_A10G1302 | chr10 | MMK1 | Mitogen-activated protein kinase homolog MMK1 | 67,409,834 | 67,413,136 | + | PF00069 | 67411485 |
| Gh_D02G0105 | chr14 | MMK1 | Mitogen-activated protein kinase homolog MMK1 | 796,564 | 800,472 | + | PF00069 | 798518 |
| Gh_D10G1179 | chr20 | MMK1 | Mitogen-activated protein kinase homolog MMK1 | 20,453,993 | 20,457,305 | - | PF00069 | 20455649 |
| Gh_A03G0709 | chr03 | NTF3 | Mitogen-activated protein kinase homolog NTF3 | 27,245,758 | 27,247,790 | + | PF00069 | 27246774 |
| Gh_D02G0958 | chr14 | NTF3 | Mitogen-activated protein kinase homolog NTF3 | 21,858,974 | 21,861,015 | + | PF00069 | 21859995 |
| Gh_D05G1825 | chr19 | NTF3 | Mitogen-activated protein kinase homolog NTF3 | 16,590,893 | 16,592,876 | - | PF00069 | 16591885 |
| Gh_A03G0759 | chr03 | NTF4 | Mitogen-activated protein kinase homolog NTF4 | 33,473,098 | 33,476,818 | - | PF00069 | 33474958 |
| Gh_D02G1080 | chr14 | NTF4 | Mitogen-activated protein kinase homolog NTF4 | 29,663,583 | 29,667,311 | - | PF00069 | 29665447 |
| Gh_A05G0857 | chr05 | NTF6 | Mitogen-activated protein kinase homolog NTF6 | 8,545,041 | 8,547,219 | - | PF00069 | 8546130 |
| Gh_A12G1883 | chr12 | MKK10 | Mitogen-activated protein kinase kinase 10 | 81,427,192 | 81,428,151 | - | PF00069 | 81427672 |
| Gh_D03G1645 | chr17 | MKK10 | Mitogen-activated protein kinase kinase 10 | 46,519,165 | 46,520,127 | + | PF00069 | 46519646 |
| Gh_D12G2062 | chr26 | MKK10 | Mitogen-activated protein kinase kinase 10 | 53,607,635 | 53,608,593 | - | PF00069 | 53608114 |
| Gh_A06G0660 | chr06 | MKK2 | Mitogen-activated protein kinase kinase 2 | 17,714,906 | 17,717,410 | - | PF00069 | 17716158 |
| Gh_A07G0124 | chr07 | MKK2 | Mitogen-activated protein kinase kinase 2 | 1,472,093 | 1,475,590 | - | PF00069 | 1473842 |
| Gh_D05G0323 | chr19 | MKK2 | Mitogen-activated protein kinase kinase 2 | 2,757,752 | 2,760,469 | - | PF00069 | 2759111 |
| Gh_D06G0748 | chr25 | MKK2 | Mitogen-activated protein kinase kinase 2 | 13,226,758 | 13,229,260 | - | PF00069 | 13228009 |
| Gh_A11G0616 | chr11 | MKK3 | Mitogen-activated protein kinase kinase 3 | 5,909,547 | 5,912,821 | - | PF00069 | 5911184 |
| Gh_D11G0703 | chr21 | MKK3 | Mitogen-activated protein kinase kinase 3 | 6,056,104 | 6,059,397 | - | PF00069 | 6057751 |
| Gh_A05G0965 | chr05 | MKK4 | Mitogen-activated protein kinase kinase 4 | 9,627,393 | 9,628,445 | + | PF00069 | 9627919 |
| Gh_D05G1074 | chr19 | MKK4 | Mitogen-activated protein kinase kinase 4 | 9,157,923 | 9,158,975 | + | PF00069 | 9158449 |
| Gh_D06G1960 | chr25 | MKK4 | Mitogen-activated protein kinase kinase 4 | 60,991,537 | 60,992,544 | - | PF00069 | 60992041 |
| Gh_A07G0085 | chr07 | MKK6 | Mitogen-activated protein kinase kinase 6 | 1,063,469 | 1,065,566 | - | PF00069 | 1064518 |
| Gh_A13G1734 | chr13 | MKK6 | Mitogen-activated protein kinase kinase 6 | 76,228,787 | 76,231,754 | + | PF00069 | 76230271 |
| Gh_D13G2082 | chr18 | MKK6 | Mitogen-activated protein kinase kinase 6 | 56,321,439 | 56,324,593 | + | PF00069 | 56323016 |
| Gh_A01G1308 | chr01 | MKK7 | Mitogen-activated protein kinase kinase 7 | 79,115,809 | 79,116,714 | + | PF00069 | 79116262 |
| Gh_D01G1562 | chr15 | MKK7 | Mitogen-activated protein kinase kinase 7 | 48,712,340 | 48,713,245 | - | PF00069 | 48712793 |
| Gh_D12G2574 | chr26 | MKK7 | Mitogen-activated protein kinase kinase 7 | 58,626,038 | 58,627,009 | - | PF00069 | 58626524 |
| Gh_D12G2575 | chr26 | MKK7 | Mitogen-activated protein kinase kinase 7 | 58,632,546 | 58,633,508 | - | PF00069 | 58633027 |
| Gh_A12G2448 | chr12 | MKK9 | Mitogen-activated protein kinase kinase 9 | 86,974,847 | 86,975,818 | - | PF00069 | 86975333 |
| Gh_A02G0770 | Chr02 | MEKK1 | Mitogen-activated protein kinase kinase kinase 1 | 15,000,122 | 15,005,535 | - | PF00069 | 15002829 |
| Gh_A05G1997 | chr05 | MEKK1 | Mitogen-activated protein kinase kinase kinase 1 | 21,364,365 | 21,369,049 | + | PF00069 | 21366707 |
| Gh_D02G0817 | chr14 | MEKK1 | Mitogen-activated protein kinase kinase kinase 1 | 13,560,408 | 13,565,850 | - | PF00069 | 13563129 |
| Gh_A07G0760 | chr07 | Map3k15 | Mitogen-activated protein kinase kinase kinase 15 | 11,873,188 | 11,874,704 | - | PF00069 | 11873946 |
| Gh_A05G1471 | chr05 | ANP2 | Mitogen-activated protein kinase kinase kinase 2 | 15,124,207 | 15,126,868 | - | PF00069 | 15125538 |
| Gh_A10G0115 | chr10 | ANP2 | Mitogen-activated protein kinase kinase kinase 2 | 944,190 | 945,530 | + | PF00069 | 944860 |
| Gh_A10G0115 | chr10 | ANP2 | Mitogen-activated protein kinase kinase kinase 2 | 944,190 | 945,530 | + | PF00069 | 944860 |
| Gh_D01G0655 | chr15 | ANP2 | Mitogen-activated protein kinase kinase kinase 2 | 9,148,745 | 9,150,134 | - | PF00069 | 9149440 |
| Gh_D05G1644 | chr19 | ANP2 | Mitogen-activated protein kinase kinase kinase 2 | 14,748,688 | 14,750,904 | - | PF00069 | 14749796 |
| Gh_D10G0119 | chr20 | ANP2 | Mitogen-activated protein kinase kinase kinase 2 | 948,355 | 949,695 | + | PF00069 | 949025 |
| Gh_A08G1792 | chr08 | ANP3 | Mitogen-activated protein kinase kinase kinase 3 | 98,252,941 | 98,254,428 | + | PF00069 | 98253685 |
| Gh_D08G2141 | chr24 | ANP3 | Mitogen-activated protein kinase kinase kinase 3 | 60,753,749 | 60,755,236 | + | PF00069 | 60754493 |
| Gh_A02G1500 | Chr02 | mkkA | Mitogen-activated protein kinase kinase kinase A | 81,194,530 | 81,195,546 | - | PF00069 | 81195038 |
| Gh_D03G0213 | chr17 | mkkA | Mitogen-activated protein kinase kinase kinase A | 2,218,541 | 2,219,557 | + | PF00069 | 2219049 |
| Gh_A03G0235 | chr03 | ANP1 | Mitogen-activated protein kinase kinase kinase ANP1 | 3,595,340 | 3,597,023 | - | PF00069 | 3596182 |
| Gh_A11G2401 | chr11 | ANP1 | Mitogen-activated protein kinase kinase kinase ANP1 | 81,868,714 | 81,873,063 | + | PF00069 | 81870889 |
| Gh_D11G2718 | chr21 | ANP1 | Mitogen-activated protein kinase kinase kinase ANP1 | 56,559,467 | 56,563,819 | + | PF00069 | 56561643 |
| Gh_A01G0637 | chr01 | NPK1 | Mitogen-activated protein kinase kinase kinase NPK1 | 11,471,680 | 11,472,993 | - | PF00069 | 11472337 |
| Gh_A09G0532 | chr09 | NPK1 | Mitogen-activated protein kinase kinase kinase NPK1 | 43,343,254 | 43,347,262 | - | PF00069 | 43345258 |
| Gh_D03G1335 | chr17 | NPK1 | Mitogen-activated protein kinase kinase kinase NPK1 | 41,819,952 | 41,821,828 | + | PF00069 | 41820890 |
| Gh_D05G1016 | chr19 | NPK1 | Mitogen-activated protein kinase kinase kinase NPK1 | 8,578,142 | 8,579,701 | + | PF00069 | 8578922 |
| Gh_D09G0528 | chr23 | NPK1 | Mitogen-activated protein kinase kinase kinase NPK1 | 25,805,826 | 25,809,889 | - | PF00069 | 25807858 |
| Gh_A02G0700 | Chr02 | YDA | Mitogen-activated protein kinase kinase kinase YODA | 11,881,841 | 11,884,664 | + | PF00069 | 11883253 |
| Gh_A05G1720 | chr05 | YDA | Mitogen-activated protein kinase kinase kinase YODA | 18,142,680 | 18,147,312 | + | PF00069 | 18144996 |
| Gh_A05G3333 | chr05 | YDA | Mitogen-activated protein kinase kinase kinase YODA | 87,261,951 | 87,266,545 | - | PF00069 | 87264248 |
| Gh_A08G1641 | chr08 | YDA | Mitogen-activated protein kinase kinase kinase YODA | 95,870,723 | 95,875,694 | - | PF00069 | 95873209 |
| Gh_A10G0824 | chr10 | YDA | Mitogen-activated protein kinase kinase kinase YODA | 17,018,029 | 17,019,039 | - | PF00069 | 17018534 |
| Gh_A10G0824 | chr10 | YDA | Mitogen-activated protein kinase kinase kinase YODA | 17,018,029 | 17,019,039 | - | PF00069 | 17018534 |
| Gh_A10G0833 | chr10 | YDA | Mitogen-activated protein kinase kinase kinase YODA | 17,384,356 | 17,388,012 | - | PF00069 | 17386184 |
| Gh_A10G0833 | chr10 | YDA | Mitogen-activated protein kinase kinase kinase YODA | 17,384,356 | 17,388,012 | - | PF00069 | 17386184 |
| Gh_A11G0423 | chr11 | YDA | Mitogen-activated protein kinase kinase kinase YODA | 3,928,895 | 3,932,397 | + | PF00069 | 3930646 |
| Gh_A11G2777 | chr11 | YDA | Mitogen-activated protein kinase kinase kinase YODA | 90,766,442 | 90,769,581 | - | PF00069 | 90768012 |
| Gh_A12G0645 | chr12 | YDA | Mitogen-activated protein kinase kinase kinase YODA | 19,761,403 | 19,765,492 | + | PF00069 | 19763448 |
| Gh_A12G0663 | chr12 | YDA | Mitogen-activated protein kinase kinase kinase YODA | 22,808,825 | 22,809,877 | + | PF00069 | 22809351 |
| Gh_D02G0745 | chr14 | YDA | Mitogen-activated protein kinase kinase kinase YODA | 11,184,498 | 11,187,375 | + | PF00069 | 11185937 |
| Gh_D04G0218 | chr22 | YDA | Mitogen-activated protein kinase kinase kinase YODA | 3,187,334 | 3,191,954 | - | PF00069 | 3189644 |
| Gh_D05G1912 | chr19 | YDA | Mitogen-activated protein kinase kinase kinase YODA | 17,563,909 | 17,569,163 | + | PF00069 | 17566536 |
| Gh_D09G0424 | chr23 | YDA | Mitogen-activated protein kinase kinase kinase YODA | 17,798,854 | 17,803,559 | - | PF00069 | 17801207 |
| Gh_D10G0923 | chr20 | YDA | Mitogen-activated protein kinase kinase kinase YODA | 12,239,057 | 12,242,193 | + | PF00069 | 12240625 |
| Gh_D10G0936 | chr20 | YDA | Mitogen-activated protein kinase kinase kinase YODA | 12,475,554 | 12,476,564 | + | PF00069 | 12476059 |
| Gh_D11G0492 | chr21 | YDA | Mitogen-activated protein kinase kinase kinase YODA | 4,189,883 | 4,193,370 | + | PF00069 | 4191627 |
| Gh_D11G1937 | chr21 | YDA | Mitogen-activated protein kinase kinase kinase YODA | 24,193,830 | 24,194,909 | + | PF00069 | 24194370 |
| Gh_D12G0667 | chr26 | YDA | Mitogen-activated protein kinase kinase kinase YODA | 13,979,159 | 13,980,211 | + | PF00069 | 13979685 |
| Gh_D12G0854 | chr26 | YDA | Mitogen-activated protein kinase kinase kinase YODA | 27,661,490 | 27,665,583 | - | PF00069 | 27663537 |
| Gh_A11G2705 | chr11 | NA | NA | 89,607,979 | 89,610,927 | - | PF00069 | 89609453 |
| Gh_A05G2000 | chr05 | ORP1C | Oxysterol-binding protein-related protein 1C | 21,424,202 | 21,446,535 | - | PF00069 | 21435369 |
| Gh_A10G1004 | chr10 | PPCK1 | Phosphoenolpyruvate carboxylase kinase 1 | 28,471,144 | 28,471,971 | + | PF00069 | 28471558 |
| Gh_A10G1004 | chr10 | PPCK1 | Phosphoenolpyruvate carboxylase kinase 1 | 28,471,144 | 28,471,971 | + | PF00069 | 28471558 |
| Gh_A13G1485 | chr13 | PPCK1 | Phosphoenolpyruvate carboxylase kinase 1 | 73,251,841 | 73,252,754 | - | PF00069 | 73252298 |
| Gh_D10G1543 | chr20 | PPCK1 | Phosphoenolpyruvate carboxylase kinase 1 | 40,324,851 | 40,325,747 | - | PF00069 | 40325299 |
| Gh_D11G2549 | chr21 | PPCK1 | Phosphoenolpyruvate carboxylase kinase 1 | 52,618,032 | 52,618,844 | + | PF00069 | 52618438 |
| Gh_D05G0261 | chr19 | Pik3r4 | Phosphoinositide 3-kinase regulatory subunit 4 | 2,328,565 | 2,336,214 | - | PF00069 | 2332390 |
| Gh_D12G0111 | chr26 | PHOT2 | Phototropin-2 | 1,402,840 | 1,419,186 | + | PF00069 | 1411013 |
| Gh_A08G0157 | chr08 | PRK2 | Pollen receptor-like kinase 2 | 1,491,172 | 1,492,005 | + | PF00069 | 1491589 |
| Gh_D03G0701 | chr17 | POT3 | Potassium transporter 3 | 24,399,473 | 24,411,190 | - | PF00069 | 24405332 |
| Gh_A11G0252 | chr11 | CDC7-1 | Probable cell division control protein 7 homolog 1 | 2,361,521 | 2,365,723 | + | PF00069 | 2363622 |
| Gh_D11G0271 | chr21 | CDC7-1 | Probable cell division control protein 7 homolog 1 | 2,329,739 | 2,333,942 | + | PF00069 | 2331841 |
| Gh_D02G2101 | chr14 | RKL1 | Probable inactive receptor kinase At1g48480 | 65,159,509 | 65,161,223 | - | PF00069 | 65160366 |
| Gh_A07G0552 | chr07 | At2g26730 | Probable inactive receptor kinase At2g26730 | 7,628,230 | 7,631,002 | - | PF00069 | 7629616 |
| Gh_A07G2006 | chr07 | At2g26730 | Probable inactive receptor kinase At2g26730 | 76,323,182 | 76,324,738 | + | PF00069 | 76323960 |
| Gh_D08G1229 | chr24 | At5g58300 | Probable inactive receptor kinase At5g58300 | 39,479,180 | 39,480,763 | + | PF00069 | 39479972 |
| Gh_A02G1491 | Chr02 | At5g67200 | Probable inactive receptor kinase At5g67200 | 81,050,018 | 81,052,920 | + | PF00069 | 81051469 |
| Gh_A11G1799 | chr11 | At5g67200 | Probable inactive receptor kinase At5g67200 | 34,418,395 | 34,420,828 | - | PF00069 | 34419612 |
| Gh_D03G0223 | chr17 | At5g67200 | Probable inactive receptor kinase At5g67200 | 2,348,736 | 2,351,761 | - | PF00069 | 2350249 |
| Gh_A03G1681 | chr03 | RLK902 | Probable inactive receptor kinase RLK902 | 98,107,942 | 98,109,654 | - | PF00069 | 98108798 |
| Gh_D07G1778 | chr16 | RLK902 | Probable inactive receptor kinase RLK902 | 42,410,446 | 42,411,503 | - | PF00069 | 42410975 |
| Gh_D05G2643 | chr19 | scy1 | Probable inactive serine/threonine-protein kinase scy1 | 27,443,247 | 27,452,455 | + | PF00069 | 27447851 |
| Gh_D06G1938 | chr25 | scy1 | Probable inactive serine/threonine-protein kinase scy1 | 60,548,721 | 60,558,574 | - | PF00069 | 60553648 |
| Gh_A11G0378 | chr11 | At1g68400 | Probable leucine-rich repeat receptor-like protein kinase At1g68400 | 3,467,545 | 3,470,260 | - | PF00069 | 3468903 |
| Gh_A11G2943 | chr11 | LRR-RLK | Probable leucine-rich repeat receptor-like serine/threonine-protein kinase At3g14840 | 93,270,229 | 93,272,350 | + | PF00069 | 93271290 |
| Gh_D11G2958 | chr21 | LRR-RLK | Probable leucine-rich repeat receptor-like serine/threonine-protein kinase At3g14840 | 60,419,906 | 60,422,107 | - | PF00069 | 60421007 |
| Gh_A10G1353 | chr10 | At1g06840 | Probable LRR receptor-like serine/threonine-protein kinase At1g06840 | 72,114,077 | 72,116,204 | - | PF00069 | 72115141 |
| Gh_A10G1353 | chr10 | At1g06840 | Probable LRR receptor-like serine/threonine-protein kinase At1g06840 | 72,114,077 | 72,116,204 | - | PF00069 | 72115141 |
| Gh_A03G0858 | chr03 | At1g14390 | Probable LRR receptor-like serine/threonine-protein kinase At1g14390 | 48,683,376 | 48,686,069 | - | PF00069 | 48684723 |
| Gh_D02G1140 | chr14 | At1g14390 | Probable LRR receptor-like serine/threonine-protein kinase At1g14390 | 34,053,503 | 34,056,193 | + | PF00069 | 34054848 |
| Gh_A01G1974 | chr01 | At1g53430 | Probable LRR receptor-like serine/threonine-protein kinase At1g53430 | 99,712,747 | 99,719,218 | + | PF00069 | 99715983 |
| Gh_A11G2593 | chr11 | At1g53430 | Probable LRR receptor-like serine/threonine-protein kinase At1g53430 | 86,491,729 | 86,493,940 | - | PF00069 | 86492835 |
| Gh_A12G0089 | chr12 | At1g53430 | Probable LRR receptor-like serine/threonine-protein kinase At1g53430 | 1,290,941 | 1,293,363 | - | PF00069 | 1292152 |
| Gh_D01G1376 | chr15 | At1g53430 | Probable LRR receptor-like serine/threonine-protein kinase At1g53430 | 40,554,981 | 40,558,684 | - | PF00069 | 40556833 |
| Gh_D05G3312 | chr19 | At1g53430 | Probable LRR receptor-like serine/threonine-protein kinase At1g53430 | 53,115,708 | 53,117,802 | + | PF00069 | 53116755 |
| Gh_D05G1458 | chr19 | At1g53440 | Probable LRR receptor-like serine/threonine-protein kinase At1g53440 | 12,968,861 | 12,972,186 | - | PF00069 | 12970524 |
| Gh_A01G0248 | chr01 | At1g56140 | Probable LRR receptor-like serine/threonine-protein kinase At1g56140 | 2,393,495 | 2,395,851 | - | PF00069 | 2394673 |
| Gh_A10G1849 | chr10 | At1g56140 | Probable LRR receptor-like serine/threonine-protein kinase At1g56140 | 94,593,019 | 94,597,612 | + | PF00069 | 94595316 |
| Gh_A10G1849 | chr10 | At1g56140 | Probable LRR receptor-like serine/threonine-protein kinase At1g56140 | 94,593,019 | 94,597,612 | + | PF00069 | 94595316 |
| Gh_D01G0246 | chr15 | At1g56140 | Probable LRR receptor-like serine/threonine-protein kinase At1g56140 | 2,142,272 | 2,144,447 | - | PF00069 | 2143360 |
| Gh_D10G2109 | chr20 | At1g56140 | Probable LRR receptor-like serine/threonine-protein kinase At1g56140 | 57,899,108 | 57,904,630 | + | PF00069 | 57901869 |
| Gh_D10G0063 | chr20 | At2g23950 | Probable LRR receptor-like serine/threonine-protein kinase At2g23950 | 514,085 | 515,682 | - | PF00069 | 514883.5 |
| Gh_A04G0157 | chr04 | At3g47570 | Probable LRR receptor-like serine/threonine-protein kinase At3g47570 | 2,639,927 | 2,640,544 | + | PF00069 | 2640236 |
| Gh_D01G1761 | chr15 | At3g47570 | Probable LRR receptor-like serine/threonine-protein kinase At3g47570 | 54,563,851 | 54,564,263 | + | PF00069 | 54564057 |
| Gh_D05G3546 | chr19 | At3g47570 | Probable LRR receptor-like serine/threonine-protein kinase At3g47570 | 58,587,389 | 58,590,300 | + | PF00069 | 58588845 |
| Gh_D09G0465 | chr23 | At3g47570 | Probable LRR receptor-like serine/threonine-protein kinase At3g47570 | 22,487,235 | 22,488,709 | + | PF00069 | 22487972 |
| Gh_D10G2369 | chr20 | At3g47570 | Probable LRR receptor-like serine/threonine-protein kinase At3g47570 | 62,594,435 | 62,595,745 | + | PF00069 | 62595090 |
| Gh_D10G2372 | chr20 | At3g47570 | Probable LRR receptor-like serine/threonine-protein kinase At3g47570 | 62,614,004 | 62,616,061 | - | PF00069 | 62615033 |
| Gh_D10G2374 | chr20 | At3g47570 | Probable LRR receptor-like serine/threonine-protein kinase At3g47570 | 62,751,967 | 62,752,779 | - | PF00069 | 62752373 |
| Gh_D10G2377 | chr20 | At3g47570 | Probable LRR receptor-like serine/threonine-protein kinase At3g47570 | 62,765,707 | 62,768,189 | - | PF00069 | 62766948 |
| Gh_A09G0182 | chr09 | At4g08850 | Probable LRR receptor-like serine/threonine-protein kinase At4g08850 | 5,098,549 | 5,123,728 | + | PF00069 | 5111139 |
| Gh_A09G0187 | chr09 | At4g08850 | Probable LRR receptor-like serine/threonine-protein kinase At4g08850 | 5,247,078 | 5,285,611 | - | PF00069 | 5266345 |
| Gh_D09G0187 | chr23 | At4g08850 | Probable LRR receptor-like serine/threonine-protein kinase At4g08850 | 5,997,156 | 5,998,496 | - | PF00069 | 5997826 |
| Gh_D02G1187 | chr14 | At4g26540 | Probable LRR receptor-like serine/threonine-protein kinase At4g26540 | 36,365,979 | 36,373,068 | - | PF00069 | 36369524 |
| Gh_D08G2307 | chr24 | At5g48740 | Probable LRR receptor-like serine/threonine-protein kinase At5g48740 | 62,746,209 | 62,748,688 | - | PF00069 | 62747449 |
| Gh_A08G1652 | chr08 | RKF3 | Probable LRR receptor-like serine/threonine-protein kinase RKF3 | 96,134,473 | 96,136,359 | + | PF00069 | 96135416 |
| Gh_A08G1653 | chr08 | RKF3 | Probable LRR receptor-like serine/threonine-protein kinase RKF3 | 96,137,835 | 96,139,676 | + | PF00069 | 96138756 |
| Gh_A11G1095 | chr11 | RKF3 | Probable LRR receptor-like serine/threonine-protein kinase RKF3 | 12,771,908 | 12,773,815 | + | PF00069 | 12772862 |
| Gh_A11G2367 | chr11 | RKF3 | Probable LRR receptor-like serine/threonine-protein kinase RKF3 | 81,008,648 | 81,010,498 | + | PF00069 | 81009573 |
| Gh_A11G2368 | chr11 | RKF3 | Probable LRR receptor-like serine/threonine-protein kinase RKF3 | 81,100,425 | 81,102,224 | + | PF00069 | 81101325 |
| Gh_A12G0046 | chr12 | RKF3 | Probable LRR receptor-like serine/threonine-protein kinase RKF3 | 632,759 | 634,645 | - | PF00069 | 633702 |
| Gh_D08G1986 | chr24 | RKF3 | Probable LRR receptor-like serine/threonine-protein kinase RKF3 | 58,321,621 | 58,323,381 | + | PF00069 | 58322501 |
| Gh_D11G1245 | chr21 | RKF3 | Probable LRR receptor-like serine/threonine-protein kinase RKF3 | 11,748,492 | 11,750,399 | + | PF00069 | 11749446 |
| Gh_D11G2683 | chr21 | RKF3 | Probable LRR receptor-like serine/threonine-protein kinase RKF3 | 55,852,090 | 55,853,892 | + | PF00069 | 55852991 |
| Gh_A01G1619 | chr01 | LECRKS5 | Probable L-type lectin-domain containing receptor kinase S.5 | 94,128,420 | 94,130,078 | + | PF00069 | 94129249 |
| Gh_D01G1842 | chr15 | LECRKS5 | Probable L-type lectin-domain containing receptor kinase S.5 | 56,128,750 | 56,129,496 | - | PF00069 | 56129123 |
| Gh_A01G0718 | chr01 | LECRKS7 | Probable L-type lectin-domain containing receptor kinase S.7 | 13,733,244 | 13,734,284 | + | PF00069 | 13733764 |
| Gh_A12G2271 | chr12 | PAP14 | Probable plastid-lipid-associated protein 14, chloroplastic | 85,371,201 | 85,375,397 | + | PF00069 | 85373299 |
| Gh_D12G2421 | chr26 | PAP14 | Probable plastid-lipid-associated protein 14, chloroplastic | 57,229,354 | 57,233,541 | + | PF00069 | 57231448 |
| Gh_A02G0743 | Chr02 | At1g11050 | Probable receptor-like protein kinase At1g11050 | 13,686,893 | 13,688,740 | - | PF00069 | 13687817 |
| Gh_A03G1710 | chr03 | At1g11050 | Probable receptor-like protein kinase At1g11050 | 98,337,490 | 98,339,436 | - | PF00069 | 98338463 |
| Gh_D02G0785 | chr14 | At1g11050 | Probable receptor-like protein kinase At1g11050 | 12,780,246 | 12,782,105 | - | PF00069 | 12781176 |
| Gh_D02G2131 | chr14 | At1g11050 | Probable receptor-like protein kinase At1g11050 | 65,443,761 | 65,445,707 | - | PF00069 | 65444734 |
| Gh_D12G0249 | chr26 | At1g11050 | Probable receptor-like protein kinase At1g11050 | 3,329,467 | 3,331,419 | - | PF00069 | 3330443 |
| Gh_A11G0150 | chr11 | At1g33260 | Probable receptor-like protein kinase At1g33260 | 1,448,956 | 1,450,937 | - | PF00069 | 1449947 |
| Gh_A12G2208 | chr12 | At1g33260 | Probable receptor-like protein kinase At1g33260 | 84,816,411 | 84,817,878 | + | PF00069 | 84817145 |
| Gh_D11G0164 | chr21 | At1g33260 | Probable receptor-like protein kinase At1g33260 | 1,433,543 | 1,435,496 | - | PF00069 | 1434520 |
| Gh_D12G2387 | chr26 | At1g33260 | Probable receptor-like protein kinase At1g33260 | 56,916,490 | 56,917,963 | + | PF00069 | 56917227 |
| Gh_A12G0207 | chr12 | At1g49730 | Probable receptor-like protein kinase At1g49730 | 3,122,831 | 3,127,177 | + | PF00069 | 3125004 |
| Gh_A12G0324 | chr12 | At1g49730 | Probable receptor-like protein kinase At1g49730 | 5,836,988 | 5,839,342 | - | PF00069 | 5838165 |
| Gh_A13G0358 | chr13 | At1g49730 | Probable receptor-like protein kinase At1g49730 | 4,771,864 | 4,775,437 | - | PF00069 | 4773651 |
| Gh_D12G0209 | chr26 | At1g49730 | Probable receptor-like protein kinase At1g49730 | 2,783,019 | 2,787,383 | + | PF00069 | 2785201 |
| Gh_D13G0403 | chr18 | At1g49730 | Probable receptor-like protein kinase At1g49730 | 4,452,713 | 4,456,331 | - | PF00069 | 4454522 |
| Gh_A07G0651 | chr07 | At1g67000 | Probable receptor-like protein kinase At1g67000 | 9,504,440 | 9,506,953 | - | PF00069 | 9505697 |
| Gh_A09G1584 | chr09 | At1g67000 | Probable receptor-like protein kinase At1g67000 | 69,256,419 | 69,257,300 | - | PF00069 | 69256860 |
| Gh_D01G0306 | chr15 | At1g67000 | Probable receptor-like protein kinase At1g67000 | 3,143,624 | 3,144,307 | + | PF00069 | 3143966 |
| Gh_D07G0728 | chr16 | At1g67000 | Probable receptor-like protein kinase At1g67000 | 8,886,032 | 8,888,547 | - | PF00069 | 8887290 |
| Gh_D09G0495 | chr23 | At1g67000 | Probable receptor-like protein kinase At1g67000 | 23,883,733 | 23,886,287 | - | PF00069 | 23885010 |
| Gh_D09G1675 | chr23 | At1g67000 | Probable receptor-like protein kinase At1g67000 | 44,387,217 | 44,398,821 | - | PF00069 | 44393019 |
| Gh_A07G0250 | chr07 | At1g80640 | Probable receptor-like protein kinase At1g80640 | 3,036,959 | 3,039,766 | + | PF00069 | 3038363 |
| Gh_D05G0580 | chr19 | At1g80640 | Probable receptor-like protein kinase At1g80640 | 4,715,111 | 4,718,356 | + | PF00069 | 4716734 |
| Gh_D05G1151 | chr19 | At1g80640 | Probable receptor-like protein kinase At1g80640 | 9,905,973 | 9,908,689 | - | PF00069 | 9907331 |
| Gh_D06G1972 | chr25 | At1g80640 | Probable receptor-like protein kinase At1g80640 | 61,218,730 | 61,220,618 | - | PF00069 | 61219674 |
| Gh_D07G0307 | chr16 | At1g80640 | Probable receptor-like protein kinase At1g80640 | 3,185,750 | 3,188,561 | + | PF00069 | 3187156 |
| Gh_A05G3285 | chr05 | At2g42960 | Probable receptor-like protein kinase At2g42960 | 86,188,093 | 86,191,046 | - | PF00069 | 86189570 |
| Gh_D04G0321 | chr22 | At2g42960 | Probable receptor-like protein kinase At2g42960 | 4,822,557 | 4,825,505 | + | PF00069 | 4824031 |
| Gh_D11G2835 | chr21 | At2g42960 | Probable receptor-like protein kinase At2g42960 | 58,266,133 | 58,268,099 | - | PF00069 | 58267116 |
| Gh_A09G0250 | chr09 | At3g17420 | Probable receptor-like protein kinase At3g17420 | 7,795,614 | 7,799,237 | - | PF00069 | 7797426 |
| Gh_D09G0248 | chr23 | At3g17420 | Probable receptor-like protein kinase At3g17420 | 7,634,709 | 7,637,773 | - | PF00069 | 7636241 |
| Gh_D08G1150 | chr24 | At4g10390 | Probable receptor-like protein kinase At4g10390 | 35,911,652 | 35,912,809 | - | PF00069 | 35912231 |
| Gh_A09G0212 | chr09 | At5g15080 | Probable receptor-like protein kinase At5g15080 | 6,302,756 | 6,305,783 | + | PF00069 | 6304270 |
| Gh_A13G0455 | chr13 | At5g15080 | Probable receptor-like protein kinase At5g15080 | 8,693,716 | 8,697,694 | + | PF00069 | 8695705 |
| Gh_D04G0458 | chr22 | At5g15080 | Probable receptor-like protein kinase At5g15080 | 7,423,541 | 7,426,965 | + | PF00069 | 7425253 |
| Gh_D05G0928 | chr19 | At5g15080 | Probable receptor-like protein kinase At5g15080 | 7,814,930 | 7,816,325 | + | PF00069 | 7815628 |
| Gh_D11G0732 | chr21 | At5g15080 | Probable receptor-like protein kinase At5g15080 | 6,313,719 | 6,316,814 | - | PF00069 | 6315267 |
| Gh_D13G0697 | chr18 | At5g15080 | Probable receptor-like protein kinase At5g15080 | 10,391,791 | 10,395,769 | - | PF00069 | 10393780 |
| Gh_A01G0239 | chr01 | At5g18500 | Probable receptor-like protein kinase At5g18500 | 2,307,203 | 2,309,122 | - | PF00069 | 2308163 |
| Gh_A02G0581 | Chr02 | At5g18500 | Probable receptor-like protein kinase At5g18500 | 8,909,406 | 8,911,670 | - | PF00069 | 8910538 |
| Gh_A03G1227 | chr03 | At5g18500 | Probable receptor-like protein kinase At5g18500 | 86,755,721 | 86,758,306 | + | PF00069 | 86757014 |
| Gh_A09G2139 | chr09 | At5g18500 | Probable receptor-like protein kinase At5g18500 | 74,474,486 | 74,477,350 | - | PF00069 | 74475918 |
| Gh_D02G0639 | chr14 | At5g18500 | Probable receptor-like protein kinase At5g18500 | 8,739,848 | 8,742,110 | - | PF00069 | 8740979 |
| Gh_A06G0491 | chr06 | At5g20050 | Probable receptor-like protein kinase At5g20050 | 9,575,031 | 9,576,380 | - | PF00069 | 9575706 |
| Gh_D06G0546 | chr25 | At5g20050 | Probable receptor-like protein kinase At5g20050 | 8,306,594 | 8,312,720 | - | PF00069 | 8309657 |
| Gh_A05G3591 | chr05 | At5g39020 | Probable receptor-like protein kinase At5g39020 | 91,781,885 | 91,784,026 | + | PF00069 | 91782956 |
| Gh_A11G1521 | chr11 | At5g39020 | Probable receptor-like protein kinase At5g39020 | 21,628,410 | 21,634,031 | - | PF00069 | 21631221 |
| Gh_D01G0308 | chr15 | At5g39020 | Probable receptor-like protein kinase At5g39020 | 3,150,462 | 3,151,205 | + | PF00069 | 3150834 |
| Gh_D01G0325 | chr15 | At5g39020 | Probable receptor-like protein kinase At5g39020 | 3,465,014 | 3,466,066 | - | PF00069 | 3465540 |
| Gh_D09G0492 | chr23 | At5g39020 | Probable receptor-like protein kinase At5g39020 | 23,752,022 | 23,753,011 | + | PF00069 | 23752517 |
| Gh_D11G1682 | chr21 | At5g39020 | Probable receptor-like protein kinase At5g39020 | 18,204,191 | 18,205,039 | - | PF00069 | 18204615 |
| Gh_A09G1391 | chr09 | At5g47070 | Probable receptor-like protein kinase At5g47070 | 67,025,147 | 67,027,572 | - | PF00069 | 67026360 |
| Gh_A12G1932 | chr12 | At5g47070 | Probable receptor-like protein kinase At5g47070 | 82,073,487 | 82,075,745 | - | PF00069 | 82074616 |
| Gh_D04G1073 | chr22 | At5g47070 | Probable receptor-like protein kinase At5g47070 | 35,295,621 | 35,298,770 | - | PF00069 | 35297196 |
| Gh_D08G0334 | chr24 | At5g47070 | Probable receptor-like protein kinase At5g47070 | 3,336,028 | 3,338,576 | - | PF00069 | 3337302 |
| Gh_D09G1396 | chr23 | At5g47070 | Probable receptor-like protein kinase At5g47070 | 41,451,193 | 41,453,574 | - | PF00069 | 41452384 |
| Gh_D12G2113 | chr26 | At5g47070 | Probable receptor-like protein kinase At5g47070 | 54,158,850 | 54,160,708 | - | PF00069 | 54159779 |
| Gh_A12G0251 | chr12 | At4g34500 | Probable receptor-like serine/threonine-protein kinase At4g34500 | 3,902,453 | 3,905,263 | - | PF00069 | 3903858 |
| Gh_D02G2141 | chr14 | At4g34500 | Probable receptor-like serine/threonine-protein kinase At4g34500 | 65,529,138 | 65,532,316 | - | PF00069 | 65530727 |
| Gh_D12G0253 | chr26 | At4g34500 | Probable receptor-like serine/threonine-protein kinase At4g34500 | 3,366,309 | 3,369,157 | - | PF00069 | 3367733 |
| Gh_A03G0183 | chr03 | At5g57670 | Probable receptor-like serine/threonine-protein kinase At5g57670 | 2,734,620 | 2,736,944 | + | PF00069 | 2735782 |
| Gh_A09G0440 | chr09 | IRE | Probable serine/threonine protein kinase IRE | 33,208,718 | 33,216,282 | + | PF00069 | 33212500 |
| Gh_D09G0451 | chr23 | IRE | Probable serine/threonine protein kinase IRE | 21,634,380 | 21,644,759 | + | PF00069 | 21639570 |
| Gh_A05G1676 | chr05 | IRE4 | Probable serine/threonine protein kinase IRE4 | 17,464,619 | 17,473,000 | + | PF00069 | 17468810 |
| Gh_D05G1860 | chr19 | IRE4 | Probable serine/threonine protein kinase IRE4 | 16,953,870 | 16,962,164 | + | PF00069 | 16958017 |
| Gh_A11G0025 | chr11 | IREH1 | Probable serine/threonine protein kinase IREH1 | 276,499 | 286,119 | - | PF00069 | 281309 |
| Gh_D09G1647 | chr23 | IREH1 | Probable serine/threonine protein kinase IREH1 | 44,092,214 | 44,104,519 | - | PF00069 | 44098367 |
| Gh_D11G0023 | chr21 | IREH1 | Probable serine/threonine protein kinase IREH1 | 229,729 | 239,349 | - | PF00069 | 234539 |
| Gh_D08G2300 | chr24 | B''GAMMA | Probable serine/threonine protein phosphatase 2A regulatory subunit B''gamma | 62,627,671 | 62,642,424 | + | PF00069 | 62635048 |
| Gh_A05G2236 | chr05 | At1g01540 | Probable serine/threonine-protein kinase At1g01540 | 25,966,553 | 25,969,117 | - | PF00069 | 25967835 |
| Gh_A07G1295 | chr07 | At1g01540 | Probable serine/threonine-protein kinase At1g01540 | 30,742,897 | 30,743,792 | + | PF00069 | 30743345 |
| Gh_A07G1296 | chr07 | At1g01540 | Probable serine/threonine-protein kinase At1g01540 | 30,743,869 | 30,747,209 | + | PF00069 | 30745539 |
| Gh_A11G0824 | chr11 | At1g01540 | Probable serine/threonine-protein kinase At1g01540 | 8,242,714 | 8,244,817 | - | PF00069 | 8243766 |
| Gh_A11G1778 | chr11 | At1g01540 | Probable serine/threonine-protein kinase At1g01540 | 31,225,226 | 31,227,702 | - | PF00069 | 31226464 |
| Gh_D05G1957 | chr19 | At1g01540 | Probable serine/threonine-protein kinase At1g01540 | 17,977,748 | 17,980,546 | - | PF00069 | 17979147 |
| Gh_D05G2498 | chr19 | At1g01540 | Probable serine/threonine-protein kinase At1g01540 | 25,121,424 | 25,123,999 | - | PF00069 | 25122712 |
| Gh_D07G1409 | chr16 | At1g01540 | Probable serine/threonine-protein kinase At1g01540 | 23,172,560 | 23,175,439 | + | PF00069 | 23174000 |
| Gh_D11G0965 | chr21 | At1g01540 | Probable serine/threonine-protein kinase At1g01540 | 8,386,457 | 8,388,563 | - | PF00069 | 8387510 |
| Gh_D11G1942 | chr21 | At1g01540 | Probable serine/threonine-protein kinase At1g01540 | 24,235,844 | 24,238,320 | - | PF00069 | 24237082 |
| Gh_A01G1162 | chr01 | At1g09600 | Probable serine/threonine-protein kinase At1g09600 | 55,445,754 | 55,450,959 | + | PF00069 | 55448357 |
| Gh_A03G0599 | chr03 | At1g09600 | Probable serine/threonine-protein kinase At1g09600 | 16,492,575 | 16,496,351 | - | PF00069 | 16494463 |
| Gh_A08G0182 | chr08 | At1g09600 | Probable serine/threonine-protein kinase At1g09600 | 1,819,020 | 1,827,222 | + | PF00069 | 1823121 |
| Gh_A08G2035 | chr08 | At1g09600 | Probable serine/threonine-protein kinase At1g09600 | 101,507,203 | 101,510,987 | - | PF00069 | 1.02E+08 |
| Gh_A09G0117 | chr09 | At1g09600 | Probable serine/threonine-protein kinase At1g09600 | 2,753,570 | 2,756,407 | - | PF00069 | 2754989 |
| Gh_A09G2146 | chr09 | At1g09600 | Probable serine/threonine-protein kinase At1g09600 | 74,520,703 | 74,526,073 | - | PF00069 | 74523388 |
| Gh_A11G2569 | chr11 | At1g09600 | Probable serine/threonine-protein kinase At1g09600 | 85,686,166 | 85,690,808 | + | PF00069 | 85688487 |
| Gh_A12G0417 | chr12 | At1g09600 | Probable serine/threonine-protein kinase At1g09600 | 8,377,697 | 8,388,378 | - | PF00069 | 8383038 |
| Gh_A13G0972 | chr13 | At1g09600 | Probable serine/threonine-protein kinase At1g09600 | 52,681,158 | 52,687,428 | + | PF00069 | 52684293 |
| Gh_A13G1241 | chr13 | At1g09600 | Probable serine/threonine-protein kinase At1g09600 | 66,272,022 | 66,276,580 | + | PF00069 | 66274301 |
| Gh_D01G1290 | chr15 | At1g09600 | Probable serine/threonine-protein kinase At1g09600 | 35,573,234 | 35,578,409 | + | PF00069 | 35575822 |
| Gh_D03G0888 | chr17 | At1g09600 | Probable serine/threonine-protein kinase At1g09600 | 30,519,083 | 30,522,569 | - | PF00069 | 30520826 |
| Gh_D04G1391 | chr22 | At1g09600 | Probable serine/threonine-protein kinase At1g09600 | 45,178,055 | 45,181,768 | - | PF00069 | 45179912 |
| Gh_D04G1763 | chr22 | At1g09600 | Probable serine/threonine-protein kinase At1g09600 | 49,917,786 | 49,922,328 | - | PF00069 | 49920057 |
| Gh_D08G2426 | chr24 | At1g09600 | Probable serine/threonine-protein kinase At1g09600 | 64,189,835 | 64,193,139 | - | PF00069 | 64191487 |
| Gh_D09G0112 | chr23 | At1g09600 | Probable serine/threonine-protein kinase At1g09600 | 2,776,480 | 2,779,437 | - | PF00069 | 2777959 |
| Gh_D09G2351 | chr23 | At1g09600 | Probable serine/threonine-protein kinase At1g09600 | 50,496,425 | 50,501,885 | - | PF00069 | 50499155 |
| Gh_D12G0413 | chr26 | At1g09600 | Probable serine/threonine-protein kinase At1g09600 | 6,636,798 | 6,641,544 | - | PF00069 | 6639171 |
| Gh_D13G1221 | chr18 | At1g09600 | Probable serine/threonine-protein kinase At1g09600 | 36,835,438 | 36,837,842 | + | PF00069 | 36836640 |
| Gh_D13G1543 | chr18 | At1g09600 | Probable serine/threonine-protein kinase At1g09600 | 47,589,890 | 47,594,530 | + | PF00069 | 47592210 |
| Gh_A03G1280 | chr03 | At1g18390 | Probable serine/threonine-protein kinase At1g18390 | 89,196,127 | 89,198,034 | - | PF00069 | 89197081 |
| Gh_D02G1720 | chr14 | At1g18390 | Probable serine/threonine-protein kinase At1g18390 | 58,720,973 | 58,722,880 | - | PF00069 | 58721927 |
| Gh_A03G0471 | chr03 | At1g54610 | Probable serine/threonine-protein kinase At1g54610 | 10,328,235 | 10,332,480 | - | PF00069 | 10330358 |
| Gh_A03G1383 | chr03 | At1g54610 | Probable serine/threonine-protein kinase At1g54610 | 92,978,110 | 92,982,769 | - | PF00069 | 92980440 |
| Gh_A05G2145 | chr05 | At1g54610 | Probable serine/threonine-protein kinase At1g54610 | 24,446,951 | 24,449,705 | - | PF00069 | 24448328 |
| Gh_A07G0419 | chr07 | At1g54610 | Probable serine/threonine-protein kinase At1g54610 | 5,374,470 | 5,378,192 | + | PF00069 | 5376331 |
| Gh_A08G1896 | chr08 | At1g54610 | Probable serine/threonine-protein kinase At1g54610 | 99,680,536 | 99,684,055 | - | PF00069 | 99682296 |
| Gh_A11G2330 | chr11 | At1g54610 | Probable serine/threonine-protein kinase At1g54610 | 80,289,757 | 80,293,494 | + | PF00069 | 80291626 |
| Gh_A12G0829 | chr12 | At1g54610 | Probable serine/threonine-protein kinase At1g54610 | 52,587,021 | 52,592,439 | + | PF00069 | 52589730 |
| Gh_A13G1507 | chr13 | At1g54610 | Probable serine/threonine-protein kinase At1g54610 | 73,570,119 | 73,573,757 | + | PF00069 | 73571938 |
| Gh_D02G1822 | chr14 | At1g54610 | Probable serine/threonine-protein kinase At1g54610 | 61,164,677 | 61,169,328 | - | PF00069 | 61167003 |
| Gh_D03G1069 | chr17 | At1g54610 | Probable serine/threonine-protein kinase At1g54610 | 35,851,626 | 35,855,883 | + | PF00069 | 35853755 |
| Gh_D05G2399 | chr19 | At1g54610 | Probable serine/threonine-protein kinase At1g54610 | 23,899,408 | 23,902,178 | - | PF00069 | 23900793 |
| Gh_D06G1201 | chr25 | At1g54610 | Probable serine/threonine-protein kinase At1g54610 | 30,349,185 | 30,353,172 | - | PF00069 | 30351179 |
| Gh_D06G1410 | chr25 | At1g54610 | Probable serine/threonine-protein kinase At1g54610 | 45,094,690 | 45,096,244 | - | PF00069 | 45095467 |
| Gh_D07G0484 | chr16 | At1g54610 | Probable serine/threonine-protein kinase At1g54610 | 5,375,384 | 5,379,095 | + | PF00069 | 5377240 |
| Gh_D08G2261 | chr24 | At1g54610 | Probable serine/threonine-protein kinase At1g54610 | 62,169,184 | 62,172,693 | - | PF00069 | 62170939 |
| Gh_D09G2395 | chr23 | At1g54610 | Probable serine/threonine-protein kinase At1g54610 | 50,863,626 | 50,867,506 | - | PF00069 | 50865566 |
| Gh_D10G1657 | chr20 | At1g54610 | Probable serine/threonine-protein kinase At1g54610 | 45,841,538 | 45,845,291 | - | PF00069 | 45843415 |
| Gh_D11G2644 | chr21 | At1g54610 | Probable serine/threonine-protein kinase At1g54610 | 55,188,782 | 55,192,511 | + | PF00069 | 55190647 |
| Gh_D12G0893 | chr26 | At1g54610 | Probable serine/threonine-protein kinase At1g54610 | 31,108,802 | 31,113,139 | + | PF00069 | 31110971 |
| Gh_D13G1836 | chr18 | At1g54610 | Probable serine/threonine-protein kinase At1g54610 | 53,458,196 | 53,461,902 | + | PF00069 | 53460049 |
| Gh_D07G1006 | chr16 | At5g41260 | Probable serine/threonine-protein kinase At5g41260 | 13,767,600 | 13,799,786 | + | PF00069 | 13783693 |
| Gh_A08G1487 | chr08 | cdc7 | Probable serine/threonine-protein kinase cdc7 | 91,316,445 | 91,321,036 | - | PF00069 | 91318741 |
| Gh_D08G1786 | chr24 | cdc7 | Probable serine/threonine-protein kinase cdc7 | 54,371,232 | 54,375,827 | - | PF00069 | 54373530 |
| Gh_D08G0574 | chr24 | DDB_G0276461 | Probable serine/threonine-protein kinase DDB_G0276461 | 6,788,288 | 6,791,204 | - | PF00069 | 6789746 |
| Gh_A03G0778 | chr03 | dyrk2 | Probable serine/threonine-protein kinase dyrk2 | 37,164,574 | 37,170,809 | - | PF00069 | 37167692 |
| Gh_A07G0545 | chr07 | dyrk2 | Probable serine/threonine-protein kinase dyrk2 | 7,438,152 | 7,445,547 | + | PF00069 | 7441850 |
| Gh_A11G0017 | chr11 | dyrk2 | Probable serine/threonine-protein kinase dyrk2 | 240,588 | 247,311 | - | PF00069 | 243949.5 |
| Gh_D02G1193 | chr14 | dyrk2 | Probable serine/threonine-protein kinase dyrk2 | 36,860,330 | 36,866,572 | - | PF00069 | 36863451 |
| Gh_D07G0612 | chr16 | dyrk2 | Probable serine/threonine-protein kinase dyrk2 | 7,078,706 | 7,086,540 | + | PF00069 | 7082623 |
| Gh_D11G0015 | chr21 | dyrk2 | Probable serine/threonine-protein kinase dyrk2 | 192,373 | 199,719 | - | PF00069 | 196046 |
| Gh_A13G0890 | chr13 | NAK | Probable serine/threonine-protein kinase NAK | 46,189,074 | 46,189,682 | - | PF00069 | 46189378 |
| Gh_D08G0016 | chr24 | At1g07870 | Probable serine/threonine-protein kinase RLCKVII | 199,494 | 201,347 | + | PF00069 | 200420.5 |
| Gh_A01G0680 | chr01 | WNK11 | Probable serine/threonine-protein kinase WNK11 | 12,211,045 | 12,213,721 | - | PF00069 | 12212383 |
| Gh_A11G1393 | chr11 | WNK11 | Probable serine/threonine-protein kinase WNK11 | 18,190,081 | 18,191,591 | + | PF00069 | 18190836 |
| Gh_D01G0701 | chr15 | WNK11 | Probable serine/threonine-protein kinase WNK11 | 9,828,245 | 9,829,254 | - | PF00069 | 9828750 |
| Gh_D11G1538 | chr21 | WNK11 | Probable serine/threonine-protein kinase WNK11 | 15,515,567 | 15,517,077 | + | PF00069 | 15516322 |
| Gh_D13G1422 | chr18 | WNK11 | Probable serine/threonine-protein kinase WNK11 | 45,063,005 | 45,063,982 | + | PF00069 | 45063494 |
| Gh_A07G0497 | chr07 | WNK2 | Probable serine/threonine-protein kinase WNK2 | 6,531,158 | 6,534,155 | + | PF00069 | 6532657 |
| Gh_A09G2164 | chr09 | WNK2 | Probable serine/threonine-protein kinase WNK2 | 74,680,999 | 74,684,571 | - | PF00069 | 74682785 |
| Gh_D07G0567 | chr16 | WNK2 | Probable serine/threonine-protein kinase WNK2 | 6,436,946 | 6,439,991 | + | PF00069 | 6438469 |
| Gh_D09G2371 | chr23 | WNK2 | Probable serine/threonine-protein kinase WNK2 | 50,669,814 | 50,673,321 | - | PF00069 | 50671568 |
| Gh_A08G1102 | chr08 | WNK4 | Probable serine/threonine-protein kinase WNK4 | 77,748,233 | 77,750,877 | - | PF00069 | 77749555 |
| Gh_D08G1382 | chr24 | WNK4 | Probable serine/threonine-protein kinase WNK4 | 45,555,930 | 45,558,586 | - | PF00069 | 45557258 |
| Gh_A02G0042 | Chr02 | WNK5 | Probable serine/threonine-protein kinase WNK5 | 276,247 | 277,915 | + | PF00069 | 277081 |
| Gh_A10G1328 | chr10 | WNK5 | Probable serine/threonine-protein kinase WNK5 | 70,439,883 | 70,442,678 | - | PF00069 | 70441281 |
| Gh_A10G1328 | chr10 | WNK5 | Probable serine/threonine-protein kinase WNK5 | 70,439,883 | 70,442,678 | - | PF00069 | 70441281 |
| Gh_D02G0055 | chr14 | WNK5 | Probable serine/threonine-protein kinase WNK5 | 316,662 | 318,141 | + | PF00069 | 317401.5 |
| Gh_D10G1149 | chr20 | WNK5 | Probable serine/threonine-protein kinase WNK5 | 18,892,742 | 18,895,539 | + | PF00069 | 18894141 |
| Gh_D04G0229 | chr22 | WNK6 | Probable serine/threonine-protein kinase WNK6 | 3,280,689 | 3,283,401 | - | PF00069 | 3282045 |
| Gh_A08G2146 | chr08 | PERK1 | Proline-rich receptor-like protein kinase PERK1 | 102,580,042 | 102,584,495 | + | PF00069 | 1.03E+08 |
| Gh_D08G2513 | chr24 | PERK1 | Proline-rich receptor-like protein kinase PERK1 | 64,889,571 | 64,893,877 | + | PF00069 | 64891724 |
| Gh_A10G0535 | chr10 | PERK12 | Proline-rich receptor-like protein kinase PERK12 | 6,295,854 | 6,299,881 | - | PF00069 | 6297868 |
| Gh_A10G0535 | chr10 | PERK12 | Proline-rich receptor-like protein kinase PERK12 | 6,295,854 | 6,299,881 | - | PF00069 | 6297868 |
| Gh_A02G0715 | Chr02 | PERK13 | Proline-rich receptor-like protein kinase PERK13 | 12,171,673 | 12,175,398 | - | PF00069 | 12173536 |
| Gh_A05G2039 | chr05 | PERK13 | Proline-rich receptor-like protein kinase PERK13 | 22,184,371 | 22,188,079 | + | PF00069 | 22186225 |
| Gh_D02G0758 | chr14 | PERK13 | Proline-rich receptor-like protein kinase PERK13 | 11,364,418 | 11,368,113 | - | PF00069 | 11366266 |
| Gh_D05G2282 | chr19 | PERK13 | Proline-rich receptor-like protein kinase PERK13 | 22,116,416 | 22,121,400 | + | PF00069 | 22118908 |
| Gh_A02G1573 | Chr02 | PERK4 | Proline-rich receptor-like protein kinase PERK4 | 82,283,866 | 82,293,853 | + | PF00069 | 82288860 |
| Gh_D03G0151 | chr17 | PERK4 | Proline-rich receptor-like protein kinase PERK4 | 1,118,333 | 1,123,672 | - | PF00069 | 1121003 |
| Gh_A12G1175 | chr12 | PERK8 | Proline-rich receptor-like protein kinase PERK8 | 65,391,071 | 65,393,291 | - | PF00069 | 65392181 |
| Gh_A09G0650 | chr09 | GFS12 | Protein GFS12 | 49,795,930 | 49,803,359 | + | PF00069 | 49799645 |
| Gh_D09G0654 | chr23 | GFS12 | Protein GFS12 | 29,765,015 | 29,788,546 | + | PF00069 | 29776781 |
| Gh_A09G1001 | chr09 | APK2B | Protein kinase 2B, chloroplastic | 59,914,642 | 59,918,526 | - | PF00069 | 59916584 |
| Gh_D09G1021 | chr23 | APK2B | Protein kinase 2B, chloroplastic | 36,189,413 | 36,193,214 | - | PF00069 | 36191314 |
| Gh_A09G1456 | chr09 | APK1B | Protein kinase APK1B, chloroplastic | 67,824,309 | 67,826,095 | - | PF00069 | 67825202 |
| Gh_A01G1804 | chr01 | dsk1 | Protein kinase dsk1 | 97,838,326 | 97,842,314 | + | PF00069 | 97840320 |
| Gh_A09G0833 | chr09 | dsk1 | Protein kinase dsk1 | 55,261,038 | 55,264,776 | - | PF00069 | 55262907 |
| Gh_D01G2044 | chr15 | dsk1 | Protein kinase dsk1 | 59,475,111 | 59,481,626 | + | PF00069 | 59478369 |
| Gh_D04G1021 | chr22 | dsk1 | Protein kinase dsk1 | 32,787,072 | 32,795,009 | + | PF00069 | 32791041 |
| Gh_D09G0838 | chr23 | dsk1 | Protein kinase dsk1 | 32,996,401 | 33,000,136 | - | PF00069 | 32998269 |
| Gh_A03G1930 | chr03 | PID | Protein kinase PINOID | 100,127,613 | 100,129,469 | + | PF00069 | 1E+08 |
| Gh_A12G0426 | chr12 | PID | Protein kinase PINOID | 8,807,798 | 8,809,801 | - | PF00069 | 8808800 |
| Gh_D02G2369 | chr14 | PID | Protein kinase PINOID | 67,146,713 | 67,148,567 | + | PF00069 | 67147640 |
| Gh_D12G0423 | chr26 | PID | Protein kinase PINOID | 6,940,024 | 6,942,008 | - | PF00069 | 6941016 |
| Gh_D05G0175 | chr19 | PID2 | Protein kinase PINOID 2 | 1,673,473 | 1,675,040 | - | PF00069 | 1674257 |
| Gh_D05G1196 | chr19 | PID2 | Protein kinase PINOID 2 | 10,311,022 | 10,311,497 | + | PF00069 | 10311260 |
| Gh_A05G3099 | chr05 | NA | Protein kinase PVPK-1 | 79,860,553 | 79,862,882 | - | PF00069 | 79861718 |
| Gh_A09G0290 | chr09 | NA | Protein kinase PVPK-1 | 10,023,251 | 10,025,621 | + | PF00069 | 10024436 |
| Gh_A10G0916 | chr10 | NA | Protein kinase PVPK-1 | 20,399,778 | 20,402,324 | - | PF00069 | 20401051 |
| Gh_A10G0916 | chr10 | NA | Protein kinase PVPK-1 | 20,399,778 | 20,402,324 | - | PF00069 | 20401051 |
| Gh_A11G0614 | chr11 | NA | Protein kinase PVPK-1 | 5,893,861 | 5,897,134 | - | PF00069 | 5895498 |
| Gh_D04G0541 | chr22 | NA | Protein kinase PVPK-1 | 9,328,783 | 9,331,112 | + | PF00069 | 9329948 |
| Gh_D09G0294 | chr23 | NA | Protein kinase PVPK-1 | 9,539,625 | 9,541,974 | + | PF00069 | 9540800 |
| Gh_D10G0834 | chr20 | NA | Protein kinase PVPK-1 | 10,384,554 | 10,387,098 | - | PF00069 | 10385826 |
| Gh_D11G0701 | chr21 | NA | Protein kinase PVPK-1 | 6,033,481 | 6,039,530 | - | PF00069 | 6036506 |
| Gh_A10G1411 | chr10 | LYK2 | Protein LYK2 | 76,686,025 | 76,687,272 | + | PF00069 | 76686649 |
| Gh_A10G1411 | chr10 | LYK2 | Protein LYK2 | 76,686,025 | 76,687,272 | + | PF00069 | 76686649 |
| Gh_D10G0813 | chr20 | LYK2 | Protein LYK2 | 10,121,353 | 10,122,600 | + | PF00069 | 10121977 |
| Gh_A05G0913 | chr05 | LYK5 | Protein LYK5 | 9,035,313 | 9,036,620 | + | PF00069 | 9035967 |
| Gh_D05G0997 | chr19 | LYK5 | Protein LYK5 | 8,426,890 | 8,428,197 | + | PF00069 | 8427544 |
| Gh_A12G2114 | chr12 | SPA2 | Protein SPA1-RELATED 2 | 84,035,768 | 84,044,594 | - | PF00069 | 84040181 |
| Gh_D12G2294 | chr26 | SPA2 | Protein SPA1-RELATED 2 | 56,154,372 | 56,163,080 | - | PF00069 | 56158726 |
| Gh_A11G2570 | chr11 | SPA3 | Protein SPA1-RELATED 3 | 85,694,689 | 85,698,838 | + | PF00069 | 85696764 |
| Gh_A11G0861 | chr11 | SPA1 | Protein SUPPRESSOR OF PHYA-105 1 | 8,669,914 | 8,674,874 | - | PF00069 | 8672394 |
| Gh_D11G1003 | chr21 | SPA1 | Protein SUPPRESSOR OF PHYA-105 1 | 8,767,995 | 8,772,961 | - | PF00069 | 8770478 |
| Gh_A06G1618 | chr06 | At3g15890 | PTI1-like tyrosine-protein kinase At3g15890 | 101,121,190 | 101,123,933 | - | PF00069 | 1.01E+08 |
| Gh_A09G0417 | chr09 | At3g15890 | PTI1-like tyrosine-protein kinase At3g15890 | 29,399,384 | 29,401,098 | + | PF00069 | 29400241 |
| Gh_D04G0816 | chr22 | At3g15890 | PTI1-like tyrosine-protein kinase At3g15890 | 18,235,524 | 18,237,351 | - | PF00069 | 18236438 |
| Gh_D05G1173 | chr19 | At3g15890 | PTI1-like tyrosine-protein kinase At3g15890 | 10,122,076 | 10,124,789 | - | PF00069 | 10123433 |
| Gh_D06G1988 | chr25 | At3g15890 | PTI1-like tyrosine-protein kinase At3g15890 | 61,493,193 | 61,495,931 | - | PF00069 | 61494562 |
| Gh_D09G0435 | chr23 | At3g15890 | PTI1-like tyrosine-protein kinase At3g15890 | 19,985,921 | 19,987,512 | - | PF00069 | 19986717 |
| Gh_A09G1943 | chr09 | At5g43745 | Putative ion channel POLLUX-like 2 | 72,562,023 | 72,571,101 | + | PF00069 | 72566562 |
| Gh_A12G1790 | chr12 | TMKL1 | Putative kinase-like protein TMKL1 | 80,485,454 | 80,487,322 | + | PF00069 | 80486388 |
| Gh_D12G1956 | chr26 | TMKL1 | Putative kinase-like protein TMKL1 | 52,599,711 | 52,601,584 | - | PF00069 | 52600648 |
| Gh_A06G1026 | chr06 | At2g24130 | Putative leucine-rich repeat receptor-like serine/threonine-protein kinase At2g24130 | 54,578,770 | 54,580,967 | - | PF00069 | 54579869 |
| Gh_D06G1239 | chr25 | At2g24130 | Putative leucine-rich repeat receptor-like serine/threonine-protein kinase At2g24130 | 33,083,718 | 33,085,043 | - | PF00069 | 33084381 |
| Gh_D10G0004 | chr20 | At2g24130 | Putative leucine-rich repeat receptor-like serine/threonine-protein kinase At2g24130 | 31,512 | 34,306 | - | PF00069 | 32909 |
| Gh_D11G3042 | chr21 | LECRK52 | Putative L-type lectin-domain containing receptor kinase V.2 | 62,146,924 | 62,147,781 | - | PF00069 | 62147353 |
| Gh_A01G0891 | chr01 | PERK11 | Putative proline-rich receptor-like protein kinase PERK11 | 21,142,087 | 21,145,440 | - | PF00069 | 21143764 |
| Gh_A02G0848 | Chr02 | PK1 | Putative receptor protein kinase ZmPK1 | 21,153,783 | 21,154,481 | + | PF00069 | 21154132 |
| Gh_A05G1787 | chr05 | PK1 | Putative receptor protein kinase ZmPK1 | 18,820,303 | 18,823,188 | - | PF00069 | 18821746 |
| Gh_D05G1982 | chr19 | PK1 | Putative receptor protein kinase ZmPK1 | 18,252,722 | 18,253,414 | - | PF00069 | 18253068 |
| Gh_D05G1984 | chr19 | PK1 | Putative receptor protein kinase ZmPK1 | 18,255,660 | 18,256,187 | - | PF00069 | 18255924 |
| Gh_D05G1341 | chr19 | At1g72540 | Putative receptor-like protein kinase At1g72540 | 11,824,821 | 11,826,548 | - | PF00069 | 11825685 |
| Gh_A05G1010 | chr05 | At1g80870 | Putative receptor-like protein kinase At1g80870 | 10,115,842 | 10,117,998 | - | PF00069 | 10116920 |
| Gh_A05G2677 | chr05 | At4g00960 | Putative receptor-like protein kinase At4g00960 | 44,285,076 | 44,288,846 | - | PF00069 | 44286961 |
| Gh_A07G1630 | chr07 | At5g39000 | Putative receptor-like protein kinase At5g39000 | 64,358,822 | 64,360,075 | + | PF00069 | 64359449 |
| Gh_D07G1943 | chr16 | NA | Putative serine/threonine-protein kinase (Fragment) | 48,197,819 | 48,199,195 | + | PF00069 | 48198507 |
| Gh_A06G1438 | chr06 | CCR3 | Putative serine/threonine-protein kinase-like protein CCR3 | 97,135,622 | 97,138,063 | + | PF00069 | 97136843 |
| Gh_A13G0142 | chr13 | CCR3 | Putative serine/threonine-protein kinase-like protein CCR3 | 1,607,720 | 1,608,916 | + | PF00069 | 1608318 |
| Gh_D06G1252 | chr25 | CCR3 | Putative serine/threonine-protein kinase-like protein CCR3 | 33,912,965 | 33,913,704 | - | PF00069 | 33913335 |
| Gh_D06G1786 | chr25 | CCR3 | Putative serine/threonine-protein kinase-like protein CCR3 | 57,699,323 | 57,701,764 | + | PF00069 | 57700544 |
| Gh_D08G0065 | chr24 | CCR3 | Putative serine/threonine-protein kinase-like protein CCR3 | 482,973 | 485,282 | - | PF00069 | 484127.5 |
| Gh_A10G1103 | chr10 | LECRKS2 | Receptor like protein kinase S.2 | 54,325,901 | 54,328,399 | - | PF00069 | 54327150 |
| Gh_A10G1103 | chr10 | LECRKS2 | Receptor like protein kinase S.2 | 54,325,901 | 54,328,399 | - | PF00069 | 54327150 |
| Gh_D10G1404 | chr20 | LECRKS2 | Receptor like protein kinase S.2 | 28,664,412 | 28,666,910 | + | PF00069 | 28665661 |
| Gh_A02G1393 | Chr02 | RBK1 | Receptor-like cytosolic serine/threonine-protein kinase RBK1 | 79,159,046 | 79,160,937 | - | PF00069 | 79159992 |
| Gh_A12G0341 | chr12 | RBK1 | Receptor-like cytosolic serine/threonine-protein kinase RBK1 | 6,300,217 | 6,301,979 | - | PF00069 | 6301098 |
| Gh_A12G0790 | chr12 | RBK1 | Receptor-like cytosolic serine/threonine-protein kinase RBK1 | 45,237,278 | 45,242,138 | - | PF00069 | 45239708 |
| Gh_D03G0329 | chr17 | RBK1 | Receptor-like cytosolic serine/threonine-protein kinase RBK1 | 3,887,719 | 3,889,609 | + | PF00069 | 3888664 |
| Gh_D11G0535 | chr21 | RBK1 | Receptor-like cytosolic serine/threonine-protein kinase RBK1 | 4,727,725 | 4,730,115 | + | PF00069 | 4728920 |
| Gh_D12G0321 | chr26 | RBK1 | Receptor-like cytosolic serine/threonine-protein kinase RBK1 | 4,545,267 | 4,547,020 | + | PF00069 | 4546144 |
| Gh_A11G1094 | chr11 | RBK2 | Receptor-like cytosolic serine/threonine-protein kinase RBK2 | 12,756,985 | 12,758,775 | - | PF00069 | 12757880 |
| Gh_A11G2648 | chr11 | RBK2 | Receptor-like cytosolic serine/threonine-protein kinase RBK2 | 88,128,432 | 88,132,771 | + | PF00069 | 88130602 |
| Gh_A12G0047 | chr12 | RBK2 | Receptor-like cytosolic serine/threonine-protein kinase RBK2 | 654,106 | 655,879 | + | PF00069 | 654992.5 |
| Gh_D01G0929 | chr15 | RBK2 | Receptor-like cytosolic serine/threonine-protein kinase RBK2 | 15,607,056 | 15,610,419 | - | PF00069 | 15608738 |
| Gh_D11G1244 | chr21 | RBK2 | Receptor-like cytosolic serine/threonine-protein kinase RBK2 | 11,739,570 | 11,741,358 | - | PF00069 | 11740464 |
| Gh_D12G0061 | chr26 | RBK2 | Receptor-like cytosolic serine/threonine-protein kinase RBK2 | 748,538 | 752,731 | + | PF00069 | 750634.5 |
| Gh_A08G0917 | chr08 | INRPK1 | Receptor-like protein kinase | 57,947,649 | 57,948,968 | - | PF00069 | 57948309 |
| Gh_D07G1826 | chr16 | FER | Receptor-like protein kinase FERONIA | 43,969,576 | 43,970,802 | + | PF00069 | 43970189 |
| Gh_D07G1827 | chr16 | FER | Receptor-like protein kinase FERONIA | 43,997,978 | 43,999,228 | + | PF00069 | 43998603 |
| Gh_A05G2260 | chr05 | IKU2 | Receptor-like protein kinase HAIKU2 | 26,468,385 | 26,468,965 | - | PF00069 | 26468675 |
| Gh_D05G2070 | chr19 | HSL1 | Receptor-like protein kinase HSL1 | 19,175,125 | 19,178,083 | - | PF00069 | 19176604 |
| Gh_A07G1176 | chr07 | ALE2 | Receptor-like serine/threonine-protein kinase ALE2 | 25,867,646 | 25,871,624 | - | PF00069 | 25869635 |
| Gh_A12G1121 | chr12 | ALE2 | Receptor-like serine/threonine-protein kinase ALE2 | 64,579,735 | 64,583,549 | + | PF00069 | 64581642 |
| Gh_D07G1276 | chr16 | ALE2 | Receptor-like serine/threonine-protein kinase ALE2 | 20,011,843 | 20,015,809 | - | PF00069 | 20013826 |
| Gh_D12G1249 | chr26 | ALE2 | Receptor-like serine/threonine-protein kinase ALE2 | 40,543,791 | 40,547,593 | + | PF00069 | 40545692 |
| Gh_A01G0300 | chr01 | At1g78530 | Receptor-like serine/threonine-protein kinase At1g78530 | 3,768,969 | 3,770,550 | - | PF00069 | 3769760 |
| Gh_D11G0876 | chr21 | At2g45590 | Receptor-like serine/threonine-protein kinase At2g45590 | 7,588,872 | 7,590,830 | + | PF00069 | 7589851 |
| Gh_A03G0265 | chr03 | NCRK | Receptor-like serine/threonine-protein kinase NCRK | 4,233,917 | 4,237,382 | + | PF00069 | 4235650 |
| Gh_D03G1302 | chr17 | NCRK | Receptor-like serine/threonine-protein kinase NCRK | 41,190,692 | 41,194,138 | - | PF00069 | 41192415 |
| Gh_D05G0988 | chr19 | NCRK | Receptor-like serine/threonine-protein kinase NCRK | 8,317,001 | 8,321,467 | - | PF00069 | 8319234 |
| Gh_D12G0342 | chr26 | NCRK | Receptor-like serine/threonine-protein kinase NCRK | 4,949,699 | 4,952,053 | + | PF00069 | 4950876 |
| Gh_A05G2118 | chr05 | Scyl2 | SCY1-like protein 2 | 24,114,443 | 24,127,488 | - | PF00069 | 24120966 |
| Gh_A05G2358 | chr05 | Scyl2 | SCY1-like protein 2 | 28,926,384 | 28,939,133 | - | PF00069 | 28932759 |
| Gh_D05G2370 | chr19 | Scyl2 | SCY1-like protein 2 | 23,550,805 | 23,563,880 | - | PF00069 | 23557343 |
| Gh_D05G2625 | chr19 | Scyl2 | SCY1-like protein 2 | 27,162,848 | 27,175,666 | - | PF00069 | 27169257 |
| Gh_D08G2439 | chr24 | Scyl2 | SCY1-like protein 2 | 64,289,549 | 64,300,678 | - | PF00069 | 64295114 |
| Gh_A03G0646 | chr03 | Stk16 | Serine/threonine-protein kinase 16 | 19,351,403 | 19,357,582 | - | PF00069 | 19354493 |
| Gh_A07G0512 | chr07 | STK16 | Serine/threonine-protein kinase 16 | 6,701,344 | 6,704,048 | + | PF00069 | 6702696 |
| Gh_A12G1672 | chr12 | Stk16 | Serine/threonine-protein kinase 16 | 78,363,362 | 78,366,290 | - | PF00069 | 78364826 |
| Gh_D03G0939 | chr17 | Stk16 | Serine/threonine-protein kinase 16 | 32,282,588 | 32,286,423 | - | PF00069 | 32284506 |
| Gh_D07G0582 | chr16 | Stk16 | Serine/threonine-protein kinase 16 | 6,640,767 | 6,643,489 | + | PF00069 | 6642128 |
| Gh_D12G1829 | chr26 | Stk16 | Serine/threonine-protein kinase 16 | 50,905,401 | 50,908,285 | - | PF00069 | 50906843 |
| Gh_A09G2007 | chr09 | STK38 | Serine/threonine-protein kinase 38 | 73,256,103 | 73,259,826 | - | PF00069 | 73257965 |
| Gh_D04G1145 | chr22 | AFC1 | Serine/threonine-protein kinase AFC1 | 37,359,418 | 37,362,893 | - | PF00069 | 37361156 |
| Gh_A03G0665 | chr03 | AFC2 | Serine/threonine-protein kinase AFC2 | 21,444,833 | 21,448,377 | - | PF00069 | 21446605 |
| Gh_A07G0492 | chr07 | AFC2 | Serine/threonine-protein kinase AFC2 | 6,443,040 | 6,446,145 | + | PF00069 | 6444593 |
| Gh_D03G0962 | chr17 | AFC2 | Serine/threonine-protein kinase AFC2 | 32,983,697 | 32,987,238 | - | PF00069 | 32985468 |
| Gh_D07G0560 | chr16 | AFC2 | Serine/threonine-protein kinase AFC2 | 6,344,453 | 6,347,576 | + | PF00069 | 6346015 |
| Gh_D05G0513 | chr19 | AFC3 | Serine/threonine-protein kinase AFC3 | 4,119,024 | 4,121,420 | + | PF00069 | 4120222 |
| Gh_D06G0995 | chr25 | AFC3 | Serine/threonine-protein kinase AFC3 | 20,631,430 | 20,635,494 | - | PF00069 | 20633462 |
| Gh_A13G1068 | chr13 | AGC1-7 | Serine/threonine-protein kinase AGC1-7 | 60,479,218 | 60,481,035 | + | PF00069 | 60480127 |
| Gh_D13G1334 | chr18 | AGC1-7 | Serine/threonine-protein kinase AGC1-7 | 42,038,967 | 42,040,777 | + | PF00069 | 42039872 |
| Gh_A05G3371 | chr05 | At3g07070 | Serine/threonine-protein kinase At3g07070 | 88,301,055 | 88,303,667 | + | PF00069 | 88302361 |
| Gh_A11G1264 | chr11 | At3g07070 | Serine/threonine-protein kinase At3g07070 | 15,702,659 | 15,704,324 | + | PF00069 | 15703492 |
| Gh_A13G0755 | chr13 | At3g07070 | Serine/threonine-protein kinase At3g07070 | 29,926,085 | 29,928,785 | - | PF00069 | 29927435 |
| Gh_D04G0266 | chr22 | At3g07070 | Serine/threonine-protein kinase At3g07070 | 3,931,573 | 3,932,793 | + | PF00069 | 3932183 |
| Gh_D11G1413 | chr21 | At3g07070 | Serine/threonine-protein kinase At3g07070 | 13,891,688 | 13,893,354 | + | PF00069 | 13892521 |
| Gh_D13G0942 | chr18 | At3g07070 | Serine/threonine-protein kinase At3g07070 | 21,028,743 | 21,031,485 | - | PF00069 | 21030114 |
| Gh_A11G0927 | chr11 | ATG1A | Serine/threonine-protein kinase ATG1a | 9,897,302 | 9,901,692 | - | PF00069 | 9899497 |
| Gh_D11G1069 | chr21 | ATG1A | Serine/threonine-protein kinase ATG1a | 9,705,891 | 9,710,288 | - | PF00069 | 9708090 |
| Gh_D04G1206 | chr22 | ATG1C | Serine/threonine-protein kinase ATG1c | 39,501,006 | 39,506,578 | + | PF00069 | 39503792 |
| Gh_D13G0973 | chr18 | ATG1C | Serine/threonine-protein kinase ATG1c | 22,358,743 | 22,365,356 | + | PF00069 | 22362050 |
| Gh_D11G0594 | chr21 | At1g49180 | Serine/threonine-protein kinase ATG1t | 5,050,671 | 5,056,480 | + | PF00069 | 5053576 |
| Gh_A03G0672 | chr03 | ATPK2 | Serine/threonine-protein kinase AtPK2/AtPK19 | 21,951,128 | 21,953,654 | + | PF00069 | 21952391 |
| Gh_A05G2375 | chr05 | ATPK2 | Serine/threonine-protein kinase AtPK2/AtPK19 | 29,300,727 | 29,303,217 | - | PF00069 | 29301972 |
| Gh_A06G1588 | chr06 | ATPK2 | Serine/threonine-protein kinase AtPK2/AtPK19 | 100,499,912 | 100,502,187 | + | PF00069 | 1.01E+08 |
| Gh_A10G1361 | chr10 | ATPK2 | Serine/threonine-protein kinase AtPK2/AtPK19 | 72,360,700 | 72,363,365 | - | PF00069 | 72362033 |
| Gh_A10G1361 | chr10 | ATPK2 | Serine/threonine-protein kinase AtPK2/AtPK19 | 72,360,700 | 72,363,365 | - | PF00069 | 72362033 |
| Gh_D03G0837 | chr17 | ATPK2 | Serine/threonine-protein kinase AtPK2/AtPK19 | 29,032,390 | 29,034,991 | - | PF00069 | 29033691 |
| Gh_D05G2640 | chr19 | ATPK2 | Serine/threonine-protein kinase AtPK2/AtPK19 | 27,374,235 | 27,376,727 | - | PF00069 | 27375481 |
| Gh_D06G1942 | chr25 | ATPK2 | Serine/threonine-protein kinase AtPK2/AtPK19 | 60,592,589 | 60,594,845 | + | PF00069 | 60593717 |
| Gh_D10G1109 | chr20 | ATPK2 | Serine/threonine-protein kinase AtPK2/AtPK19 | 17,756,731 | 17,759,390 | + | PF00069 | 17758061 |
| Gh_D05G0466 | chr19 | AUR1 | Serine/threonine-protein kinase Aurora-1 | 3,739,748 | 3,741,818 | + | PF00069 | 3740783 |
| Gh_D07G0202 | chr16 | AUR1 | Serine/threonine-protein kinase Aurora-1 | 2,145,586 | 2,147,874 | + | PF00069 | 2146730 |
| Gh_A08G1143 | chr08 | AUR3 | Serine/threonine-protein kinase Aurora-3 | 80,334,431 | 80,336,432 | - | PF00069 | 80335432 |
| Gh_D08G1426 | chr24 | AUR3 | Serine/threonine-protein kinase Aurora-3 | 46,956,577 | 46,958,566 | - | PF00069 | 46957572 |
| Gh_A02G0649 | Chr02 | BLUS1 | Serine/threonine-protein kinase BLUS1 | 10,365,470 | 10,366,112 | - | PF00069 | 10365791 |
| Gh_A09G1965 | chr09 | BLUS1 | Serine/threonine-protein kinase BLUS1 | 72,754,449 | 72,756,014 | + | PF00069 | 72755232 |
| Gh_A10G1126 | chr10 | BLUS1 | Serine/threonine-protein kinase BLUS1 | 57,194,822 | 57,196,222 | - | PF00069 | 57195522 |
| Gh_A10G1126 | chr10 | BLUS1 | Serine/threonine-protein kinase BLUS1 | 57,194,822 | 57,196,222 | - | PF00069 | 57195522 |
| Gh_A10G1127 | chr10 | BLUS1 | Serine/threonine-protein kinase BLUS1 | 57,205,771 | 57,207,386 | - | PF00069 | 57206579 |
| Gh_A10G1127 | chr10 | BLUS1 | Serine/threonine-protein kinase BLUS1 | 57,205,771 | 57,207,386 | - | PF00069 | 57206579 |
| Gh_A10G1128 | chr10 | BLUS1 | Serine/threonine-protein kinase BLUS1 | 57,237,749 | 57,239,146 | - | PF00069 | 57238448 |
| Gh_A10G1128 | chr10 | BLUS1 | Serine/threonine-protein kinase BLUS1 | 57,237,749 | 57,239,146 | - | PF00069 | 57238448 |
| Gh_D09G2168 | chr23 | BLUS1 | Serine/threonine-protein kinase BLUS1 | 48,846,363 | 48,847,928 | + | PF00069 | 48847146 |
| Gh_D10G1370 | chr20 | BLUS1 | Serine/threonine-protein kinase BLUS1 | 27,102,872 | 27,104,296 | + | PF00069 | 27103584 |
| Gh_D10G1378 | chr20 | BLUS1 | Serine/threonine-protein kinase BLUS1 | 27,324,637 | 27,326,037 | - | PF00069 | 27325337 |
| Gh_D10G1380 | chr20 | BLUS1 | Serine/threonine-protein kinase BLUS1 | 27,366,551 | 27,367,954 | - | PF00069 | 27367253 |
| Gh_D10G1382 | chr20 | BLUS1 | Serine/threonine-protein kinase BLUS1 | 27,545,204 | 27,545,771 | - | PF00069 | 27545488 |
| Gh_A08G1693 | chr08 | CBK1 | Serine/threonine-protein kinase CBK1 | 96,659,354 | 96,665,549 | + | PF00069 | 96662452 |
| Gh_A11G1210 | chr11 | CBK1 | Serine/threonine-protein kinase CBK1 | 15,032,215 | 15,036,793 | + | PF00069 | 15034504 |
| Gh_D02G1046 | chr14 | CBK1 | Serine/threonine-protein kinase CBK1 | 27,082,267 | 27,089,840 | + | PF00069 | 27086054 |
| Gh_D05G0320 | chr19 | CBK1 | Serine/threonine-protein kinase CBK1 | 2,724,980 | 2,731,098 | + | PF00069 | 2728039 |
| Gh_D05G0411 | chr19 | CBK1 | Serine/threonine-protein kinase CBK1 | 3,353,511 | 3,360,410 | - | PF00069 | 3356961 |
| Gh_D08G2051 | chr24 | CBK1 | Serine/threonine-protein kinase CBK1 | 59,200,899 | 59,206,963 | + | PF00069 | 59203931 |
| Gh_A01G0842 | chr01 | CDL1 | Serine/threonine-protein kinase CDL1 | 19,326,732 | 19,329,911 | + | PF00069 | 19328322 |
| Gh_A03G1241 | chr03 | CDL1 | Serine/threonine-protein kinase CDL1 | 87,893,180 | 87,900,170 | + | PF00069 | 87896675 |
| Gh_A11G2389 | chr11 | CDL1 | Serine/threonine-protein kinase CDL1 | 81,711,818 | 81,713,830 | - | PF00069 | 81712824 |
| Gh_A12G0517 | chr12 | CDL1 | Serine/threonine-protein kinase CDL1 | 12,622,688 | 12,625,188 | + | PF00069 | 12623938 |
| Gh_A13G1528 | chr13 | CDL1 | Serine/threonine-protein kinase CDL1 | 73,872,476 | 73,874,354 | - | PF00069 | 73873415 |
| Gh_D01G0869 | chr15 | CDL1 | Serine/threonine-protein kinase CDL1 | 14,257,745 | 14,261,002 | + | PF00069 | 14259374 |
| Gh_D02G1680 | chr14 | CDL1 | Serine/threonine-protein kinase CDL1 | 57,936,029 | 57,938,164 | + | PF00069 | 57937097 |
| Gh_D04G1772 | chr22 | CDL1 | Serine/threonine-protein kinase CDL1 | 49,974,643 | 49,977,993 | - | PF00069 | 49976318 |
| Gh_D11G2704 | chr21 | CDL1 | Serine/threonine-protein kinase CDL1 | 56,373,217 | 56,375,219 | - | PF00069 | 56374218 |
| Gh_D13G1859 | chr18 | CDL1 | Serine/threonine-protein kinase CDL1 | 53,727,747 | 53,729,620 | - | PF00069 | 53728684 |
| Gh_A01G0730 | chr01 | D6PKL2 | Serine/threonine-protein kinase D6PKL2 | 14,103,942 | 14,105,839 | - | PF00069 | 14104891 |
| Gh_A05G3152 | chr05 | D6PKL2 | Serine/threonine-protein kinase D6PKL2 | 81,851,585 | 81,853,597 | - | PF00069 | 81852591 |
| Gh_A05G3420 | chr05 | D6PKL2 | Serine/threonine-protein kinase D6PKL2 | 89,180,213 | 89,184,146 | + | PF00069 | 89182180 |
| Gh_A06G1729 | chr06 | D6PKL2 | Serine/threonine-protein kinase D6PKL2 | 102,491,116 | 102,493,036 | + | PF00069 | 1.02E+08 |
| Gh_A07G0985 | chr07 | D6PKL2 | Serine/threonine-protein kinase D6PKL2 | 18,734,164 | 18,737,741 | + | PF00069 | 18735953 |
| Gh_A08G1247 | chr08 | D6PKL2 | Serine/threonine-protein kinase D6PKL2 | 83,755,763 | 83,758,975 | - | PF00069 | 83757369 |
| Gh_A09G0893 | chr09 | D6PKL2 | Serine/threonine-protein kinase D6PKL2 | 57,349,149 | 57,352,284 | + | PF00069 | 57350717 |
| Gh_A13G1106 | chr13 | D6PKL2 | Serine/threonine-protein kinase D6PKL2 | 61,653,551 | 61,655,990 | + | PF00069 | 61654771 |
| Gh_D01G0750 | chr15 | D6PKL2 | Serine/threonine-protein kinase D6PKL2 | 10,831,070 | 10,832,964 | - | PF00069 | 10832017 |
| Gh_D04G0170 | chr22 | D6PKL2 | Serine/threonine-protein kinase D6PKL2 | 2,449,510 | 2,453,494 | - | PF00069 | 2451502 |
| Gh_D04G0480 | chr22 | D6PKL2 | Serine/threonine-protein kinase D6PKL2 | 7,924,247 | 7,926,264 | + | PF00069 | 7925256 |
| Gh_D06G2249 | chr25 | D6PKL2 | Serine/threonine-protein kinase D6PKL2 | 64,001,102 | 64,003,021 | - | PF00069 | 64002062 |
| Gh_D07G1063 | chr16 | D6PKL2 | Serine/threonine-protein kinase D6PKL2 | 15,100,883 | 15,104,560 | + | PF00069 | 15102722 |
| Gh_D08G1537 | chr24 | D6PKL2 | Serine/threonine-protein kinase D6PKL2 | 49,265,263 | 49,268,362 | - | PF00069 | 49266813 |
| Gh_D09G0918 | chr23 | D6PKL2 | Serine/threonine-protein kinase D6PKL2 | 34,555,537 | 34,558,556 | + | PF00069 | 34557047 |
| Gh_D13G1374 | chr18 | D6PKL2 | Serine/threonine-protein kinase D6PKL2 | 43,028,787 | 43,031,227 | + | PF00069 | 43030007 |
| Gh_A03G0920 | chr03 | dst1 | Serine/threonine-protein kinase dst1 | 58,029,297 | 58,068,872 | + | PF00069 | 58049085 |
| Gh_A12G1469 | chr12 | dst1 | Serine/threonine-protein kinase dst1 | 73,989,650 | 73,998,202 | - | PF00069 | 73993926 |
| Gh_D02G1301 | chr14 | dst1 | Serine/threonine-protein kinase dst1 | 42,567,875 | 42,599,261 | + | PF00069 | 42583568 |
| Gh_D12G1597 | chr26 | dst1 | Serine/threonine-protein kinase dst1 | 47,186,333 | 47,195,143 | - | PF00069 | 47190738 |
| Gh_A05G1198 | chr05 | fray2 | Serine/threonine-protein kinase fray2 | 12,052,475 | 12,059,518 | - | PF00069 | 12055997 |
| Gh_A09G0260 | chr09 | fray2 | Serine/threonine-protein kinase fray2 | 8,066,244 | 8,075,963 | + | PF00069 | 8071104 |
| Gh_A10G0452 | chr10 | fray2 | Serine/threonine-protein kinase fray2 | 4,624,153 | 4,635,381 | + | PF00069 | 4629767 |
| Gh_A10G0452 | chr10 | fray2 | Serine/threonine-protein kinase fray2 | 4,624,153 | 4,635,381 | + | PF00069 | 4629767 |
| Gh_A10G0929 | chr10 | fray2 | Serine/threonine-protein kinase fray2 | 20,595,736 | 20,601,766 | - | PF00069 | 20598751 |
| Gh_A10G0929 | chr10 | fray2 | Serine/threonine-protein kinase fray2 | 20,595,736 | 20,601,766 | - | PF00069 | 20598751 |
| Gh_A11G0169 | chr11 | fray2 | Serine/threonine-protein kinase fray2 | 1,594,066 | 1,600,916 | + | PF00069 | 1597491 |
| Gh_A11G0621 | chr11 | fray2 | Serine/threonine-protein kinase fray2 | 5,958,585 | 5,981,010 | - | PF00069 | 5969798 |
| Gh_A12G2165 | chr12 | fray2 | Serine/threonine-protein kinase fray2 | 84,423,803 | 84,430,737 | - | PF00069 | 84427270 |
| Gh_A13G1292 | chr13 | fray2 | Serine/threonine-protein kinase fray2 | 67,470,869 | 67,472,056 | - | PF00069 | 67471463 |
| Gh_D05G1375 | chr19 | fray2 | Serine/threonine-protein kinase fray2 | 12,073,222 | 12,080,262 | - | PF00069 | 12076742 |
| Gh_D08G1180 | chr24 | fray2 | Serine/threonine-protein kinase fray2 | 37,768,759 | 37,775,539 | + | PF00069 | 37772149 |
| Gh_D09G0260 | chr23 | fray2 | Serine/threonine-protein kinase fray2 | 8,002,711 | 8,009,075 | + | PF00069 | 8005893 |
| Gh_D10G0469 | chr20 | fray2 | Serine/threonine-protein kinase fray2 | 4,453,648 | 4,459,660 | + | PF00069 | 4456654 |
| Gh_D10G0845 | chr20 | fray2 | Serine/threonine-protein kinase fray2 | 10,525,352 | 10,532,985 | - | PF00069 | 10529169 |
| Gh_D11G0179 | chr21 | fray2 | Serine/threonine-protein kinase fray2 | 1,602,618 | 1,609,464 | + | PF00069 | 1606041 |
| Gh_D11G0709 | chr21 | fray2 | Serine/threonine-protein kinase fray2 | 6,123,097 | 6,130,839 | - | PF00069 | 6126968 |
| Gh_D12G2342 | chr26 | fray2 | Serine/threonine-protein kinase fray2 | 56,491,515 | 56,498,401 | - | PF00069 | 56494958 |
| Gh_D13G1592 | chr18 | fray2 | Serine/threonine-protein kinase fray2 | 48,589,420 | 48,590,586 | - | PF00069 | 48590003 |
| Gh_A08G0085 | chr08 | GRIK2 | Serine/threonine-protein kinase GRIK2 | 756,097 | 760,011 | + | PF00069 | 758054 |
| Gh_D05G0750 | chr19 | GRIK2 | Serine/threonine-protein kinase GRIK2 | 6,143,073 | 6,145,995 | - | PF00069 | 6144534 |
| Gh_D07G1171 | chr16 | GRIK2 | Serine/threonine-protein kinase GRIK2 | 17,525,427 | 17,529,418 | + | PF00069 | 17527423 |
| Gh_D08G0126 | chr24 | GRIK2 | Serine/threonine-protein kinase GRIK2 | 1,067,848 | 1,071,689 | + | PF00069 | 1069769 |
| Gh_A03G0494 | chr03 | HT1 | Serine/threonine-protein kinase HT1 | 11,520,005 | 11,522,787 | + | PF00069 | 11521396 |
| Gh_A09G1287 | chr09 | KIPK | Serine/threonine-protein kinase KIPK | 65,282,432 | 65,285,551 | - | PF00069 | 65283992 |
| Gh_A11G2867 | chr11 | KIPK | Serine/threonine-protein kinase KIPK | 92,601,631 | 92,604,346 | + | PF00069 | 92602989 |
| Gh_D04G0991 | chr22 | KIPK | Serine/threonine-protein kinase KIPK | 30,645,985 | 30,649,131 | + | PF00069 | 30647558 |
| Gh_D05G0835 | chr19 | KIPK | Serine/threonine-protein kinase KIPK | 6,998,869 | 7,004,243 | + | PF00069 | 7001556 |
| Gh_D09G1330 | chr23 | KIPK | Serine/threonine-protein kinase KIPK | 40,490,915 | 40,494,036 | + | PF00069 | 40492476 |
| Gh_D11G3249 | chr21 | KIPK | Serine/threonine-protein kinase KIPK | 65,521,804 | 65,524,512 | + | PF00069 | 65523158 |
| Gh_A07G1806 | chr07 | MHK | Serine/threonine-protein kinase MHK | 72,968,276 | 72,974,657 | + | PF00069 | 72971467 |
| Gh_A11G1297 | chr11 | MHK | Serine/threonine-protein kinase MHK | 16,666,275 | 16,676,526 | - | PF00069 | 16671401 |
| Gh_D07G2011 | chr16 | MHK | Serine/threonine-protein kinase MHK | 49,800,247 | 49,812,068 | + | PF00069 | 49806158 |
| Gh_D11G1445 | chr21 | MHK | Serine/threonine-protein kinase MHK | 14,403,950 | 14,410,456 | - | PF00069 | 14407203 |
| Gh_A06G0347 | chr06 | mph1 | Serine/threonine-protein kinase mph1 | 5,374,944 | 5,378,215 | - | PF00069 | 5376580 |
| Gh_D05G2207 | chr19 | mph1 | Serine/threonine-protein kinase mph1 | 20,995,694 | 21,001,140 | - | PF00069 | 20998417 |
| Gh_D06G0379 | chr25 | mph1 | Serine/threonine-protein kinase mph1 | 5,202,318 | 5,206,875 | - | PF00069 | 5204597 |
| Gh_A08G0593 | chr08 | NEK2 | Serine/threonine-protein kinase Nek2 | 10,699,081 | 10,704,271 | - | PF00069 | 10701676 |
| Gh_A11G2297 | chr11 | NEK2 | Serine/threonine-protein kinase Nek2 | 79,088,452 | 79,092,801 | + | PF00069 | 79090627 |
| Gh_A12G0021 | chr12 | NEK2 | Serine/threonine-protein kinase Nek2 | 368,170 | 378,917 | - | PF00069 | 373543.5 |
| Gh_A13G1495 | chr13 | NEK2 | Serine/threonine-protein kinase Nek2 | 73,381,116 | 73,387,669 | + | PF00069 | 73384393 |
| Gh_D08G0686 | chr24 | NEK2 | Serine/threonine-protein kinase Nek2 | 9,432,516 | 9,437,709 | - | PF00069 | 9435113 |
| Gh_D11G2608 | chr21 | NEK2 | Serine/threonine-protein kinase Nek2 | 54,233,353 | 54,237,706 | + | PF00069 | 54235530 |
| Gh_D13G1824 | chr18 | NEK2 | Serine/threonine-protein kinase Nek2 | 53,316,988 | 53,320,721 | + | PF00069 | 53318855 |
| Gh_A07G1028 | chr07 | NEK5 | Serine/threonine-protein kinase Nek5 | 19,864,250 | 19,870,015 | - | PF00069 | 19867133 |
| Gh_A09G0932 | chr09 | NEK5 | Serine/threonine-protein kinase Nek5 | 58,241,730 | 58,249,141 | - | PF00069 | 58245436 |
| Gh_D05G0790 | chr19 | NEK5 | Serine/threonine-protein kinase Nek5 | 6,581,939 | 6,587,945 | + | PF00069 | 6584942 |
| Gh_D07G1105 | chr16 | NEK5 | Serine/threonine-protein kinase Nek5 | 15,970,600 | 15,976,377 | - | PF00069 | 15973489 |
| Gh_D09G0960 | chr23 | NEK5 | Serine/threonine-protein kinase Nek5 | 35,132,881 | 35,140,321 | - | PF00069 | 35136601 |
| Gh_D13G2315 | chr18 | NEK5 | Serine/threonine-protein kinase Nek5 | 59,126,926 | 59,132,412 | + | PF00069 | 59129669 |
| Gh_D13G2319 | chr18 | NEK5 | Serine/threonine-protein kinase Nek5 | 59,309,847 | 59,313,119 | + | PF00069 | 59311483 |
| Gh_A08G0117 | chr08 | NEK6 | Serine/threonine-protein kinase Nek6 | 1,031,008 | 1,047,041 | + | PF00069 | 1039025 |
| Gh_A09G1167 | chr09 | NEK6 | Serine/threonine-protein kinase Nek6 | 63,164,387 | 63,168,974 | - | PF00069 | 63166681 |
| Gh_D08G0162 | chr24 | NEK6 | Serine/threonine-protein kinase Nek6 | 1,486,621 | 1,492,671 | + | PF00069 | 1489646 |
| Gh_D09G1173 | chr23 | NEK6 | Serine/threonine-protein kinase Nek6 | 38,513,077 | 38,520,819 | - | PF00069 | 38516948 |
| Gh_A01G1666 | chr01 | NEK7 | Serine/threonine-protein kinase Nek7 | 95,207,256 | 95,218,115 | + | PF00069 | 95212686 |
| Gh_D01G1916 | chr15 | NEK7 | Serine/threonine-protein kinase Nek7 | 57,421,994 | 57,428,557 | + | PF00069 | 57425276 |
| Gh_D06G1026 | chr25 | OXSR1 | Serine/threonine-protein kinase OSR1 | 21,877,378 | 21,878,200 | - | PF00069 | 21877789 |
| Gh_A07G1798 | chr07 | OXI1 | Serine/threonine-protein kinase OXI1 | 72,824,405 | 72,825,837 | - | PF00069 | 72825121 |
| Gh_D07G1608 | chr16 | OXI1 | Serine/threonine-protein kinase OXI1 | 31,378,990 | 31,380,350 | - | PF00069 | 31379670 |
| Gh_D07G2002 | chr16 | OXI1 | Serine/threonine-protein kinase OXI1 | 49,665,743 | 49,667,148 | - | PF00069 | 49666446 |
| Gh_A01G0195 | chr01 | PBS1 | Serine/threonine-protein kinase PBS1 | 1,977,528 | 1,980,567 | - | PF00069 | 1979048 |
| Gh_A01G0607 | chr01 | PBS1 | Serine/threonine-protein kinase PBS1 | 10,949,030 | 10,950,941 | - | PF00069 | 10949986 |
| Gh_A09G0371 | chr09 | PBS1 | Serine/threonine-protein kinase PBS1 | 20,643,450 | 20,646,670 | + | PF00069 | 20645060 |
| Gh_A09G0599 | chr09 | PBS1 | Serine/threonine-protein kinase PBS1 | 48,180,544 | 48,185,529 | - | PF00069 | 48183037 |
| Gh_A09G2123 | chr09 | PBS1 | Serine/threonine-protein kinase PBS1 | 74,214,065 | 74,216,591 | + | PF00069 | 74215328 |
| Gh_A10G0136 | chr10 | PBS1 | Serine/threonine-protein kinase PBS1 | 1,140,092 | 1,143,498 | + | PF00069 | 1141795 |
| Gh_A10G0136 | chr10 | PBS1 | Serine/threonine-protein kinase PBS1 | 1,140,092 | 1,143,498 | + | PF00069 | 1141795 |
| Gh_A10G1372 | chr10 | PBS1 | Serine/threonine-protein kinase PBS1 | 72,808,957 | 72,811,853 | + | PF00069 | 72810405 |
| Gh_A10G1372 | chr10 | PBS1 | Serine/threonine-protein kinase PBS1 | 72,808,957 | 72,811,853 | + | PF00069 | 72810405 |
| Gh_A10G1428 | chr10 | PBS1 | Serine/threonine-protein kinase PBS1 | 76,992,334 | 76,996,340 | - | PF00069 | 76994337 |
| Gh_A10G1428 | chr10 | PBS1 | Serine/threonine-protein kinase PBS1 | 76,992,334 | 76,996,340 | - | PF00069 | 76994337 |
| Gh_A11G0581 | chr11 | PBS1 | Serine/threonine-protein kinase PBS1 | 5,526,202 | 5,528,583 | + | PF00069 | 5527393 |
| Gh_A11G2528 | chr11 | PBS1 | Serine/threonine-protein kinase PBS1 | 84,174,473 | 84,179,684 | + | PF00069 | 84177079 |
| Gh_D01G0203 | chr15 | PBS1 | Serine/threonine-protein kinase PBS1 | 1,722,442 | 1,726,729 | - | PF00069 | 1724586 |
| Gh_D01G0204 | chr15 | PBS1 | Serine/threonine-protein kinase PBS1 | 1,728,998 | 1,733,542 | - | PF00069 | 1731270 |
| Gh_D01G0620 | chr15 | PBS1 | Serine/threonine-protein kinase PBS1 | 8,739,921 | 8,741,797 | - | PF00069 | 8740859 |
| Gh_D01G1270 | chr15 | PBS1 | Serine/threonine-protein kinase PBS1 | 33,149,935 | 33,151,816 | + | PF00069 | 33150876 |
| Gh_D09G0393 | chr23 | PBS1 | Serine/threonine-protein kinase PBS1 | 14,685,202 | 14,688,491 | - | PF00069 | 14686847 |
| Gh_D09G0598 | chr23 | PBS1 | Serine/threonine-protein kinase PBS1 | 28,597,778 | 28,602,783 | - | PF00069 | 28600281 |
| Gh_D09G2328 | chr23 | PBS1 | Serine/threonine-protein kinase PBS1 | 50,307,778 | 50,310,311 | + | PF00069 | 50309045 |
| Gh_D10G0142 | chr20 | PBS1 | Serine/threonine-protein kinase PBS1 | 1,136,664 | 1,140,481 | + | PF00069 | 1138573 |
| Gh_D10G1093 | chr20 | PBS1 | Serine/threonine-protein kinase PBS1 | 17,444,019 | 17,446,887 | - | PF00069 | 17445453 |
| Gh_D10G1669 | chr20 | PBS1 | Serine/threonine-protein kinase PBS1 | 46,120,320 | 46,124,338 | - | PF00069 | 46122329 |
| Gh_D11G0666 | chr21 | PBS1 | Serine/threonine-protein kinase PBS1 | 5,785,103 | 5,787,771 | + | PF00069 | 5786437 |
| Gh_D11G2878 | chr21 | PBS1 | Serine/threonine-protein kinase PBS1 | 58,817,997 | 58,819,812 | + | PF00069 | 58818905 |
| Gh_D12G0531 | chr26 | PBS1 | Serine/threonine-protein kinase PBS1 | 9,721,146 | 9,723,631 | + | PF00069 | 9722389 |
| Gh_A12G0138 | chr12 | PEPKR2 | Serine/threonine-protein kinase PEPKR2 | 2,042,354 | 2,044,782 | + | PF00069 | 2043568 |
| Gh_D05G2131 | chr19 | PEPKR2 | Serine/threonine-protein kinase PEPKR2 | 19,949,019 | 19,951,531 | - | PF00069 | 19950275 |
| Gh_D06G0278 | chr25 | PEPKR2 | Serine/threonine-protein kinase PEPKR2 | 3,166,167 | 3,168,472 | - | PF00069 | 3167320 |
| Gh_D12G0152 | chr26 | PEPKR2 | Serine/threonine-protein kinase PEPKR2 | 1,941,763 | 1,944,196 | + | PF00069 | 1942980 |
| Gh_A10G2128 | chr10 | ppk15 | Serine/threonine-protein kinase ppk15 | 100,086,421 | 100,089,305 | - | PF00069 | 1E+08 |
| Gh_A10G2128 | chr10 | ppk15 | Serine/threonine-protein kinase ppk15 | 100,086,421 | 100,089,305 | - | PF00069 | 1E+08 |
| Gh_A11G2021 | chr11 | ppk15 | Serine/threonine-protein kinase ppk15 | 58,841,476 | 58,852,585 | - | PF00069 | 58847031 |
| Gh_A13G1373 | chr13 | ppk15 | Serine/threonine-protein kinase ppk15 | 69,884,107 | 69,895,526 | - | PF00069 | 69889817 |
| Gh_D13G1683 | chr18 | ppk15 | Serine/threonine-protein kinase ppk15 | 50,452,902 | 50,464,400 | - | PF00069 | 50458651 |
| Gh_D13G0738 | chr18 | Prpf4b | Serine/threonine-protein kinase PRP4 homolog | 11,599,231 | 11,604,269 | - | PF00069 | 11601750 |
| Gh_A02G0451 | Chr02 | RUK | Serine/threonine-protein kinase RUNKEL | 6,132,920 | 6,138,808 | + | PF00069 | 6135864 |
| Gh_D02G0503 | chr14 | RUK | Serine/threonine-protein kinase RUNKEL | 6,585,300 | 6,591,185 | + | PF00069 | 6588243 |
| Gh_D10G1651 | chr20 | RUK | Serine/threonine-protein kinase RUNKEL | 45,597,222 | 45,603,302 | + | PF00069 | 45600262 |
| Gh_A11G1858 | chr11 | SAPK1 | Serine/threonine-protein kinase SAPK1 | 45,306,099 | 45,308,154 | + | PF00069 | 45307127 |
| Gh_A02G0789 | Chr02 | SAPK2 | Serine/threonine-protein kinase SAPK2 | 16,299,920 | 16,301,838 | - | PF00069 | 16300879 |
| Gh_A05G1922 | chr05 | SAPK2 | Serine/threonine-protein kinase SAPK2 | 20,148,675 | 20,150,866 | + | PF00069 | 20149771 |
| Gh_A11G0474 | chr11 | SAPK2 | Serine/threonine-protein kinase SAPK2 | 4,550,580 | 4,552,694 | + | PF00069 | 4551637 |
| Gh_D02G0839 | chr14 | SAPK2 | Serine/threonine-protein kinase SAPK2 | 14,597,151 | 14,599,078 | - | PF00069 | 14598115 |
| Gh_D05G2155 | chr19 | SAPK2 | Serine/threonine-protein kinase SAPK2 | 20,185,702 | 20,187,904 | + | PF00069 | 20186803 |
| Gh_D11G0552 | chr21 | SAPK2 | Serine/threonine-protein kinase SAPK2 | 4,806,770 | 4,808,880 | + | PF00069 | 4807825 |
| Gh_D11G2149 | chr21 | SAPK2 | Serine/threonine-protein kinase SAPK2 | 32,580,911 | 32,582,968 | + | PF00069 | 32581940 |
| Gh_A08G1469 | chr08 | SAPK3 | Serine/threonine-protein kinase SAPK3 | 90,892,068 | 90,893,718 | + | PF00069 | 90892893 |
| Gh_D08G1765 | chr24 | SAPK3 | Serine/threonine-protein kinase SAPK3 | 54,052,159 | 54,054,485 | + | PF00069 | 54053322 |
| Gh_A10G1380 | chr10 | SAPK7 | Serine/threonine-protein kinase SAPK7 | 73,521,474 | 73,525,386 | + | PF00069 | 73523430 |
| Gh_A10G1380 | chr10 | SAPK7 | Serine/threonine-protein kinase SAPK7 | 73,521,474 | 73,525,386 | + | PF00069 | 73523430 |
| Gh_D10G1083 | chr20 | SAPK7 | Serine/threonine-protein kinase SAPK7 | 17,134,725 | 17,138,661 | - | PF00069 | 17136693 |
| Gh_D03G0174 | chr17 | SAPK8 | Serine/threonine-protein kinase SAPK8 | 1,297,257 | 1,297,878 | - | PF00069 | 1297568 |
| Gh_D05G0851 | chr19 | spk-1 | Serine/threonine-protein kinase spk-1 | 7,098,750 | 7,101,686 | - | PF00069 | 7100218 |
| Gh_A12G0247 | chr12 | SRK2B | Serine/threonine-protein kinase SRK2B | 3,776,901 | 3,779,651 | + | PF00069 | 3778276 |
| Gh_A13G0314 | chr13 | SRK2B | Serine/threonine-protein kinase SRK2B | 3,920,394 | 3,923,558 | - | PF00069 | 3921976 |
| Gh_D12G0247 | chr26 | SRK2B | Serine/threonine-protein kinase SRK2B | 3,272,445 | 3,275,139 | + | PF00069 | 3273792 |
| Gh_D13G0352 | chr18 | SRK2B | Serine/threonine-protein kinase SRK2B | 3,566,616 | 3,569,517 | - | PF00069 | 3568067 |
| Gh_A01G0057 | chr01 | SRK2E | Serine/threonine-protein kinase SRK2E | 447,830 | 450,835 | - | PF00069 | 449332.5 |
| Gh_A03G1684 | chr03 | SRK2E | Serine/threonine-protein kinase SRK2E | 98,135,397 | 98,138,978 | - | PF00069 | 98137188 |
| Gh_A11G1757 | chr11 | SRK2E | Serine/threonine-protein kinase SRK2E | 29,069,141 | 29,071,943 | - | PF00069 | 29070542 |
| Gh_D02G2104 | chr14 | SRK2E | Serine/threonine-protein kinase SRK2E | 65,195,858 | 65,199,438 | - | PF00069 | 65197648 |
| Gh_D02G1835 | chr14 | SRK2G | Serine/threonine-protein kinase SRK2G | 61,371,452 | 61,372,160 | - | PF00069 | 61371806 |
| Gh_D05G1382 | chr19 | SRK2G | Serine/threonine-protein kinase SRK2G | 12,127,766 | 12,129,761 | + | PF00069 | 12128764 |
| Gh_D07G0405 | chr16 | SRK2G | Serine/threonine-protein kinase SRK2G | 4,449,878 | 4,452,750 | - | PF00069 | 4451314 |
| Gh_D08G1246 | chr24 | SRK2H | Serine/threonine-protein kinase SRK2H | 40,595,319 | 40,595,681 | - | PF00069 | 40595500 |
| Gh_A12G0641 | chr12 | SRK2I | Serine/threonine-protein kinase SRK2I | 18,241,166 | 18,244,267 | - | PF00069 | 18242717 |
| Gh_D11G0489 | chr21 | SRK2I | Serine/threonine-protein kinase SRK2I | 4,160,991 | 4,163,929 | - | PF00069 | 4162460 |
| Gh_D12G0859 | chr26 | SRK2I | Serine/threonine-protein kinase SRK2I | 28,126,914 | 28,129,959 | + | PF00069 | 28128437 |
| Gh_A02G1710 | Chr02 | NA | Serine/threonine-protein kinase SRPK | 83,259,873 | 83,262,376 | - | PF00069 | 83261125 |
| Gh_A10G0892 | chr10 | NA | Serine/threonine-protein kinase SRPK | 19,305,188 | 19,306,760 | - | PF00069 | 19305974 |
| Gh_A10G0892 | chr10 | NA | Serine/threonine-protein kinase SRPK | 19,305,188 | 19,306,760 | - | PF00069 | 19305974 |
| Gh_D10G0857 | chr20 | NA | Serine/threonine-protein kinase SRPK | 10,814,404 | 10,815,976 | + | PF00069 | 10815190 |
| Gh_A12G1556 | chr12 | STN7 | Serine/threonine-protein kinase STN7, chloroplastic | 75,626,925 | 75,631,401 | + | PF00069 | 75629163 |
| Gh_D12G1659 | chr26 | STN7 | Serine/threonine-protein kinase STN7, chloroplastic | 48,147,834 | 48,159,145 | - | PF00069 | 48153490 |
| Gh_A02G0024 | Chr02 | STN8 | Serine/threonine-protein kinase STN8, chloroplastic | 147,617 | 150,195 | - | PF00069 | 148906 |
| Gh_D02G0038 | chr14 | STN8 | Serine/threonine-protein kinase STN8, chloroplastic | 193,649 | 196,213 | - | PF00069 | 194931 |
| Gh_A01G0443 | chr01 | svkA | Serine/threonine-protein kinase svkA | 7,081,664 | 7,089,822 | - | PF00069 | 7085743 |
| Gh_A13G1253 | chr13 | svkA | Serine/threonine-protein kinase svkA | 66,602,025 | 66,608,948 | + | PF00069 | 66605487 |
| Gh_D01G0453 | chr15 | svkA | Serine/threonine-protein kinase svkA | 5,401,264 | 5,409,445 | - | PF00069 | 5405355 |
| Gh_D05G3352 | chr19 | svkA | Serine/threonine-protein kinase svkA | 54,297,272 | 54,307,354 | - | PF00069 | 54302313 |
| Gh_A05G3551 | chr05 | TIO | Serine/threonine-protein kinase TIO | 91,208,249 | 91,218,049 | - | PF00069 | 91213149 |
| Gh_D05G0400 | chr19 | Tnni3k | Serine/threonine-protein kinase TNNI3K | 3,285,304 | 3,286,755 | - | PF00069 | 3286030 |
| Gh_A07G1048 | chr07 | TOUSLED | Serine/threonine-protein kinase TOUSLED | 20,753,470 | 20,762,204 | + | PF00069 | 20757837 |
| Gh_D07G1128 | chr16 | TOUSLED | Serine/threonine-protein kinase TOUSLED | 16,588,329 | 16,597,063 | + | PF00069 | 16592696 |
| Gh_D13G2312 | chr18 | TOUSLED | Serine/threonine-protein kinase TOUSLED | 59,089,512 | 59,098,021 | + | PF00069 | 59093767 |
| Gh_A02G0390 | Chr02 | trc | Serine/threonine-protein kinase tricorner | 4,974,014 | 4,977,919 | + | PF00069 | 4975967 |
| Gh_A05G1749 | chr05 | trc | Serine/threonine-protein kinase tricorner | 18,466,458 | 18,470,808 | - | PF00069 | 18468633 |
| Gh_D02G0443 | chr14 | trc | Serine/threonine-protein kinase tricorner | 5,821,536 | 5,825,453 | + | PF00069 | 5823495 |
| Gh_D05G1944 | chr19 | trc | Serine/threonine-protein kinase tricorner | 17,870,935 | 17,875,297 | - | PF00069 | 17873116 |
| Gh_D06G0148 | chr25 | trc | Serine/threonine-protein kinase tricorner | 1,463,047 | 1,467,231 | - | PF00069 | 1465139 |
| Gh_D09G2220 | chr23 | trc | Serine/threonine-protein kinase tricorner | 49,393,505 | 49,397,211 | - | PF00069 | 49395358 |
| Gh_A07G1140 | chr07 | UCNL | Serine/threonine-protein kinase UCNL | 24,703,720 | 24,704,952 | + | PF00069 | 24704336 |
| Gh_A08G0116 | chr08 | UCNL | Serine/threonine-protein kinase UCNL | 1,013,789 | 1,015,027 | - | PF00069 | 1014408 |
| Gh_D05G0690 | chr19 | UCNL | Serine/threonine-protein kinase UCNL | 5,611,428 | 5,612,672 | - | PF00069 | 5612050 |
| Gh_D07G1236 | chr16 | UCNL | Serine/threonine-protein kinase UCNL | 19,196,059 | 19,197,291 | + | PF00069 | 19196675 |
| Gh_D08G0161 | chr24 | UCNL | Serine/threonine-protein kinase UCNL | 1,471,521 | 1,472,759 | - | PF00069 | 1472140 |
| Gh_A05G2870 | chr05 | WAG1 | Serine/threonine-protein kinase WAG1 | 66,850,589 | 66,852,430 | + | PF00069 | 66851510 |
| Gh_D05G3179 | chr19 | WAG1 | Serine/threonine-protein kinase WAG1 | 48,877,410 | 48,878,882 | + | PF00069 | 48878146 |
| Gh_A01G0246 | chr01 | WAG2 | Serine/threonine-protein kinase WAG2 | 2,375,340 | 2,376,647 | - | PF00069 | 2375994 |
| Gh_D01G0244 | chr15 | WAG2 | Serine/threonine-protein kinase WAG2 | 2,124,303 | 2,125,610 | - | PF00069 | 2124957 |
| Gh_A02G0300 | Chr02 | WNK1 | Serine/threonine-protein kinase WNK1 | 3,580,344 | 3,583,100 | + | PF00069 | 3581722 |
| Gh_A11G2311 | chr11 | WNK1 | Serine/threonine-protein kinase WNK1 | 79,445,838 | 79,448,802 | + | PF00069 | 79447320 |
| Gh_D02G0364 | chr14 | WNK1 | Serine/threonine-protein kinase WNK1 | 4,843,486 | 4,846,222 | + | PF00069 | 4844854 |
| Gh_D11G2621 | chr21 | WNK1 | Serine/threonine-protein kinase WNK1 | 54,650,203 | 54,653,173 | + | PF00069 | 54651688 |
| Gh_A02G0963 | Chr02 | WNK8 | Serine/threonine-protein kinase WNK8 | 40,734,538 | 40,736,580 | + | PF00069 | 40735559 |
| Gh_A12G2159 | chr12 | WNK8 | Serine/threonine-protein kinase WNK8 | 84,396,273 | 84,399,203 | + | PF00069 | 84397738 |
| Gh_D03G0796 | chr17 | WNK8 | Serine/threonine-protein kinase WNK8 | 27,359,386 | 27,362,243 | + | PF00069 | 27360815 |
| Gh_D12G2336 | chr26 | WNK8 | Serine/threonine-protein kinase WNK8 | 56,462,404 | 56,465,331 | + | PF00069 | 56463868 |
| Gh_A01G0827 | chr01 | IRE1B | Serine/threonine-protein kinase/endoribonuclease IRE1b | 18,922,173 | 18,925,648 | + | PF00069 | 18923911 |
| Gh_D01G0855 | chr15 | IRE1B | Serine/threonine-protein kinase/endoribonuclease IRE1b | 14,005,193 | 14,008,660 | + | PF00069 | 14006927 |
| Gh_D05G2533 | chr19 | At1g28390 | Serine/threonine-protein kinase-like protein At1g28390 | 25,614,257 | 25,615,714 | + | PF00069 | 25614986 |
| Gh_A11G0665 | chr11 | At3g51990 | Serine/threonine-protein kinase-like protein At3g51990 | 6,535,421 | 6,536,755 | - | PF00069 | 6536088 |
| Gh_A01G1533 | chr01 | At5g23170 | Serine/threonine-protein kinase-like protein At5g23170 | 91,449,874 | 91,450,950 | - | PF00069 | 91450412 |
| Gh_D01G1782 | chr15 | At5g23170 | Serine/threonine-protein kinase-like protein At5g23170 | 54,841,997 | 54,843,076 | - | PF00069 | 54842537 |
| Gh_D09G1411 | chr23 | CCR1 | Serine/threonine-protein kinase-like protein CCR1 | 41,665,916 | 41,667,895 | + | PF00069 | 41666906 |
| Gh_A05G2557 | chr05 | CCR2 | Serine/threonine-protein kinase-like protein CCR2 | 36,151,160 | 36,153,469 | - | PF00069 | 36152315 |
| Gh_D05G2835 | chr19 | CCR2 | Serine/threonine-protein kinase-like protein CCR2 | 31,877,753 | 31,880,062 | - | PF00069 | 31878908 |
| Gh_A05G2138 | chr05 | CCR4 | Serine/threonine-protein kinase-like protein CCR4 | 24,392,723 | 24,394,747 | + | PF00069 | 24393735 |
| Gh_D11G2830 | chr21 | ASK1 | Shaggy-related protein kinase alpha | 58,071,261 | 58,074,075 | - | PF00069 | 58072668 |
| Gh_A09G0712 | chr09 | ASK7 | Shaggy-related protein kinase eta | 52,841,598 | 52,844,894 | + | PF00069 | 52843246 |
| Gh_A09G0713 | chr09 | ASK7 | Shaggy-related protein kinase eta | 52,861,382 | 52,864,569 | + | PF00069 | 52862976 |
| Gh_D06G2142 | chr25 | ASK7 | Shaggy-related protein kinase eta | 63,261,021 | 63,263,915 | - | PF00069 | 63262468 |
| Gh_A01G1558 | chr01 | ASK10 | Shaggy-related protein kinase kappa | 92,410,497 | 92,413,873 | + | PF00069 | 92412185 |
| Gh_A12G0411 | chr12 | ASK10 | Shaggy-related protein kinase kappa | 8,244,999 | 8,248,430 | - | PF00069 | 8246715 |
| Gh_D01G1809 | chr15 | ASK10 | Shaggy-related protein kinase kappa | 55,472,543 | 55,475,936 | + | PF00069 | 55474240 |
| Gh_D12G0407 | chr26 | ASK10 | Shaggy-related protein kinase kappa | 6,533,937 | 6,537,332 | - | PF00069 | 6535635 |
| Gh_A08G0285 | chr08 | ASK8 | Shaggy-related protein kinase theta | 3,329,052 | 3,334,479 | - | PF00069 | 3331766 |
| Gh_A08G1158 | chr08 | ASK8 | Shaggy-related protein kinase theta | 81,021,333 | 81,025,764 | + | PF00069 | 81023549 |
| Gh_A11G0778 | chr11 | ASK8 | Shaggy-related protein kinase theta | 7,649,123 | 7,654,545 | + | PF00069 | 7651834 |
| Gh_D08G0378 | chr24 | ASK8 | Shaggy-related protein kinase theta | 3,871,236 | 3,880,133 | - | PF00069 | 3875685 |
| Gh_D08G1440 | chr24 | ASK8 | Shaggy-related protein kinase theta | 47,361,120 | 47,366,590 | + | PF00069 | 47363855 |
| Gh_D11G0907 | chr21 | ASK8 | Shaggy-related protein kinase theta | 7,839,230 | 7,844,650 | + | PF00069 | 7841940 |
| Gh_A07G1890 | chr07 | KIN10 | SNF1-related protein kinase catalytic subunit alpha KIN10 | 74,319,961 | 74,324,190 | - | PF00069 | 74322076 |
| Gh_A09G0121 | chr09 | KIN10 | SNF1-related protein kinase catalytic subunit alpha KIN10 | 2,820,437 | 2,824,427 | + | PF00069 | 2822432 |
| Gh_A11G0099 | chr11 | KIN10 | SNF1-related protein kinase catalytic subunit alpha KIN10 | 1,017,591 | 1,019,698 | - | PF00069 | 1018645 |
| Gh_A11G0635 | chr11 | KIN10 | SNF1-related protein kinase catalytic subunit alpha KIN10 | 6,187,260 | 6,191,524 | - | PF00069 | 6189392 |
| Gh_D07G2105 | chr16 | KIN10 | SNF1-related protein kinase catalytic subunit alpha KIN10 | 51,203,302 | 51,207,560 | - | PF00069 | 51205431 |
| Gh_D09G0115 | chr23 | KIN10 | SNF1-related protein kinase catalytic subunit alpha KIN10 | 2,887,561 | 2,891,530 | + | PF00069 | 2889546 |
| Gh_D11G0103 | chr21 | KIN10 | SNF1-related protein kinase catalytic subunit alpha KIN10 | 969,282 | 971,360 | - | PF00069 | 970321 |
| Gh_D11G0746 | chr21 | KIN10 | SNF1-related protein kinase catalytic subunit alpha KIN10 | 6,438,510 | 6,442,742 | - | PF00069 | 6440626 |
| Gh_A07G1313 | chr07 | PUB33 | U-box domain-containing protein 33 | 32,067,793 | 32,073,385 | - | PF00069 | 32070589 |
| Gh_D07G1426 | chr16 | PUB33 | U-box domain-containing protein 33 | 23,982,296 | 23,987,682 | - | PF00069 | 23984989 |
| Gh_A05G3526 | chr05 | PUB34 | U-box domain-containing protein 34 | 90,923,175 | 90,929,967 | + | PF00069 | 90926571 |
| Gh_D05G0413 | chr19 | PUB34 | U-box domain-containing protein 34 | 3,375,791 | 3,378,930 | + | PF00069 | 3377361 |
| Gh_A07G1074 | chr07 | VPS26B | Vacuolar protein sorting-associated protein 26B | 21,768,248 | 21,795,680 | + | PF00069 | 21781964 |
| Gh_A02G1099 | Chr02 | WAK2 | Wall-associated receptor kinase 2 | 56,279,712 | 56,280,827 | - | PF00069 | 56280270 |
| Gh_D05G2276 | chr19 | WAK3 | Wall-associated receptor kinase 3 | 22,008,129 | 22,010,496 | + | PF00069 | 22009313 |
| Gh_A06G0113 | chr06 | WAKL14 | Wall-associated receptor kinase-like 14 | 1,021,213 | 1,025,194 | - | PF00069 | 1023204 |
| Gh_D11G1900 | chr21 | WAKL14 | Wall-associated receptor kinase-like 14 | 22,573,821 | 22,576,791 | - | PF00069 | 22575306 |
| Gh_A09G0176 | chr09 | WAKL9 | Wall-associated receptor kinase-like 9 | 4,692,719 | 4,694,242 | - | PF00069 | 4693481 |
| Gh_D10G1949 | chr20 | WAKL9 | Wall-associated receptor kinase-like 9 | 54,525,208 | 54,528,884 | - | PF00069 | 54527046 |
| Gh_A11G1058 | chr11 | WEE1 | Wee1-like protein kinase | 12,177,041 | 12,200,356 | + | PF00069 | 12188699 |
| Gh_D11G1213 | chr21 | WEE1 | Wee1-like protein kinase | 11,351,065 | 11,354,836 | + | PF00069 | 11352951 |
