# Supplementary material for: GBS Mapping and Analysis of Genes Conserved between Gossypium tomentosum and Gossypium hirsutum Cotton Cultivars that Respond to Drought Stress at the Seedling Stage of the BC2F2 Generation
Source: Int J Mol Sci. 2018 May 30;19(6):1614. doi: 10.3390/ijms19061614 (PMC6032168; doi:10.3390/ijms19061614)
Supplement: Supplementary file 1 [file ijms-19-01614-s001.zip › Supplimentary/Supplementary Table 5 The dominant sub domain of the Pkinase, Serine threonine-protein kinase proteins family.docx]

Supplementary Table 5: the dominant sub domain of the Pkinase, Serine/threonine-protein kinase proteins family

| Gene ID | Chro. | Total numbers | Gene Name | Description | Start | End | Strand | Domain | mapping position (bp) |
| --- | --- | --- | --- | --- | --- | --- | --- | --- | --- |
| Gh_A03G0646 | chr03 | 7 | Stk16 | Serine/threonine-protein kinase 16 | 19,351,403 | 19,357,582 | - | PF00069 | 19354492.5 |
| Gh_A07G0512 | chr07 |  | STK16 | Serine/threonine-protein kinase 16 | 6,701,344 | 6,704,048 | + | PF00069 | 6702696 |
| Gh_A12G1672 | chr12 |  | Stk16 | Serine/threonine-protein kinase 16 | 78,363,362 | 78,366,290 | - | PF00069 | 78364826 |
| Gh_D03G0939 | chr17 |  | Stk16 | Serine/threonine-protein kinase 16 | 32,282,588 | 32,286,423 | - | PF00069 | 32284505.5 |
| Gh_D07G0582 | chr16 |  | Stk16 | Serine/threonine-protein kinase 16 | 6,640,767 | 6,643,489 | + | PF00069 | 6642128 |
| Gh_D12G1829 | chr26 |  | Stk16 | Serine/threonine-protein kinase 16 | 50,905,401 | 50,908,285 | - | PF00069 | 50906843 |
| Gh_A09G2007 | chr09 |  | Stk38 | Serine/threonine-protein kinase 38 | 73,256,103 | 73,259,826 | - | PF00069 | 73257964.5 |
| Gh_D04G1145 | chr22 | 7 | AFC1 | Serine/threonine-protein kinase AFC1 | 37,359,418 | 37,362,893 | - | PF00069 | 37361155.5 |
| Gh_A03G0665 | chr03 |  | AFC2 | Serine/threonine-protein kinase AFC2 | 21,444,833 | 21,448,377 | - | PF00069 | 21446605 |
| Gh_A07G0492 | chr07 |  | AFC2 | Serine/threonine-protein kinase AFC2 | 6,443,040 | 6,446,145 | + | PF00069 | 6444592.5 |
| Gh_D03G0962 | chr17 |  | AFC2 | Serine/threonine-protein kinase AFC2 | 32,983,697 | 32,987,238 | - | PF00069 | 32985467.5 |
| Gh_D07G0560 | chr16 |  | AFC2 | Serine/threonine-protein kinase AFC2 | 6,344,453 | 6,347,576 | + | PF00069 | 6346014.5 |
| Gh_D05G0513 | chr19 |  | AFC3 | Serine/threonine-protein kinase AFC3 | 4,119,024 | 4,121,420 | + | PF00069 | 4120222 |
| Gh_D06G0995 | chr25 |  | AFC3 | Serine/threonine-protein kinase AFC3 | 20,631,430 | 20,635,494 | - | PF00069 | 20633462 |
| Gh_A13G1068 | chr13 | 2 | AGC1-7 | Serine/threonine-protein kinase AGC1-7 | 60,479,218 | 60,481,035 | + | PF00069 | 60480126.5 |
| Gh_D13G1334 | chr18 |  | AGC1-7 | Serine/threonine-protein kinase AGC1-7 | 42,038,967 | 42,040,777 | + | PF00069 | 42039872 |
| Gh_A05G3371 | chr05 | 6 | At3g07070 | Serine/threonine-protein kinase At3g07070 | 88,301,055 | 88,303,667 | + | PF00069 | 88302361 |
| Gh_A11G1264 | chr11 |  | At3g07070 | Serine/threonine-protein kinase At3g07070 | 15,702,659 | 15,704,324 | + | PF00069 | 15703491.5 |
| Gh_A13G0755 | chr13 |  | At3g07070 | Serine/threonine-protein kinase At3g07070 | 29,926,085 | 29,928,785 | - | PF00069 | 29927435 |
| Gh_D04G0266 | chr22 |  | At3g07070 | Serine/threonine-protein kinase At3g07070 | 3,931,573 | 3,932,793 | + | PF00069 | 3932183 |
| Gh_D11G1413 | chr21 |  | At3g07070 | Serine/threonine-protein kinase At3g07070 | 13,891,688 | 13,893,354 | + | PF00069 | 13892521 |
| Gh_D13G0942 | chr18 |  | At3g07070 | Serine/threonine-protein kinase At3g07070 | 21,028,743 | 21,031,485 | - | PF00069 | 21030114 |
| Gh_A11G0927 | chr11 | 4 | ATG1A | Serine/threonine-protein kinase ATG1a | 9,897,302 | 9,901,692 | - | PF00069 | 9899497 |
| Gh_D11G1069 | chr21 |  | ATG1A | Serine/threonine-protein kinase ATG1a | 9,705,891 | 9,710,288 | - | PF00069 | 9708089.5 |
| Gh_D04G1206 | chr22 |  | ATG1C | Serine/threonine-protein kinase ATG1c | 39,501,006 | 39,506,578 | + | PF00069 | 39503792 |
| Gh_D13G0973 | chr18 |  | ATG1C | Serine/threonine-protein kinase ATG1c | 22,358,743 | 22,365,356 | + | PF00069 | 22362049.5 |
| Gh_D11G0594 | chr21 | 1 | At1g49180 | Serine/threonine-protein kinase ATG1t | 5,050,671 | 5,056,480 | + | PF00069 | 5053575.5 |
| Gh_A03G0672 | chr03 | 9 | ATPK2 | Serine/threonine-protein kinase AtPK2/AtPK19 | 21,951,128 | 21,953,654 | + | PF00069 | 21952391 |
| Gh_A05G2375 | chr05 |  | ATPK2 | Serine/threonine-protein kinase AtPK2/AtPK19 | 29,300,727 | 29,303,217 | - | PF00069 | 29301972 |
| Gh_A06G1588 | chr06 |  | ATPK2 | Serine/threonine-protein kinase AtPK2/AtPK19 | 100,499,912 | 100,502,187 | + | PF00069 | 100501049.5 |
| Gh_A10G1361 | chr10 |  | ATPK2 | Serine/threonine-protein kinase AtPK2/AtPK19 | 72,360,700 | 72,363,365 | - | PF00069 | 72362032.5 |
| Gh_A10G1361 | chr10 |  | ATPK2 | Serine/threonine-protein kinase AtPK2/AtPK19 | 72,360,700 | 72,363,365 | - | PF00069 | 72362032.5 |
| Gh_D03G0837 | chr17 |  | ATPK2 | Serine/threonine-protein kinase AtPK2/AtPK19 | 29,032,390 | 29,034,991 | - | PF00069 | 29033690.5 |
| Gh_D05G2640 | chr19 |  | ATPK2 | Serine/threonine-protein kinase AtPK2/AtPK19 | 27,374,235 | 27,376,727 | - | PF00069 | 27375481 |
| Gh_D06G1942 | chr25 |  | ATPK2 | Serine/threonine-protein kinase AtPK2/AtPK19 | 60,592,589 | 60,594,845 | + | PF00069 | 60593717 |
| Gh_D10G1109 | chr20 |  | ATPK2 | Serine/threonine-protein kinase AtPK2/AtPK19 | 17,756,731 | 17,759,390 | + | PF00069 | 17758060.5 |
| Gh_D05G0466 | chr19 | 4 | AUR1 | Serine/threonine-protein kinase Aurora-1 | 3,739,748 | 3,741,818 | + | PF00069 | 3740783 |
| Gh_D07G0202 | chr16 |  | AUR1 | Serine/threonine-protein kinase Aurora-1 | 2,145,586 | 2,147,874 | + | PF00069 | 2146730 |
| Gh_A08G1143 | chr08 |  | AUR3 | Serine/threonine-protein kinase Aurora-3 | 80,334,431 | 80,336,432 | - | PF00069 | 80335431.5 |
| Gh_D08G1426 | chr24 |  | AUR3 | Serine/threonine-protein kinase Aurora-3 | 46,956,577 | 46,958,566 | - | PF00069 | 46957571.5 |
| Gh_A02G0649 | Chr02 | 13 | BLUS1 | Serine/threonine-protein kinase BLUS1 | 10,365,470 | 10,366,112 | - | PF00069 | 10365791 |
| Gh_A09G1965 | chr09 |  | BLUS1 | Serine/threonine-protein kinase BLUS1 | 72,754,449 | 72,756,014 | + | PF00069 | 72755231.5 |
| Gh_A10G1126 | chr10 |  | BLUS1 | Serine/threonine-protein kinase BLUS1 | 57,194,822 | 57,196,222 | - | PF00069 | 57195522 |
| Gh_A10G1126 | chr10 |  | BLUS1 | Serine/threonine-protein kinase BLUS1 | 57,194,822 | 57,196,222 | - | PF00069 | 57195522 |
| Gh_A10G1127 | chr10 |  | BLUS1 | Serine/threonine-protein kinase BLUS1 | 57,205,771 | 57,207,386 | - | PF00069 | 57206578.5 |
| Gh_A10G1127 | chr10 |  | BLUS1 | Serine/threonine-protein kinase BLUS1 | 57,205,771 | 57,207,386 | - | PF00069 | 57206578.5 |
| Gh_A10G1128 | chr10 |  | BLUS1 | Serine/threonine-protein kinase BLUS1 | 57,237,749 | 57,239,146 | - | PF00069 | 57238447.5 |
| Gh_A10G1128 | chr10 |  | BLUS1 | Serine/threonine-protein kinase BLUS1 | 57,237,749 | 57,239,146 | - | PF00069 | 57238447.5 |
| Gh_D09G2168 | chr23 |  | BLUS1 | Serine/threonine-protein kinase BLUS1 | 48,846,363 | 48,847,928 | + | PF00069 | 48847145.5 |
| Gh_D10G1370 | chr20 |  | BLUS1 | Serine/threonine-protein kinase BLUS1 | 27,102,872 | 27,104,296 | + | PF00069 | 27103584 |
| Gh_D10G1378 | chr20 |  | BLUS1 | Serine/threonine-protein kinase BLUS1 | 27,324,637 | 27,326,037 | - | PF00069 | 27325337 |
| Gh_D10G1380 | chr20 |  | BLUS1 | Serine/threonine-protein kinase BLUS1 | 27,366,551 | 27,367,954 | - | PF00069 | 27367252.5 |
| Gh_D10G1382 | chr20 |  | BLUS1 | Serine/threonine-protein kinase BLUS1 | 27,545,204 | 27,545,771 | - | PF00069 | 27545487.5 |
| Gh_A08G1693 | chr08 | 6 | CBK1 | Serine/threonine-protein kinase CBK1 | 96,659,354 | 96,665,549 | + | PF00069 | 96662451.5 |
| Gh_A11G1210 | chr11 |  | CBK1 | Serine/threonine-protein kinase CBK1 | 15,032,215 | 15,036,793 | + | PF00069 | 15034504 |
| Gh_D02G1046 | chr14 |  | CBK1 | Serine/threonine-protein kinase CBK1 | 27,082,267 | 27,089,840 | + | PF00069 | 27086053.5 |
| Gh_D05G0320 | chr19 |  | CBK1 | Serine/threonine-protein kinase CBK1 | 2,724,980 | 2,731,098 | + | PF00069 | 2728039 |
| Gh_D05G0411 | chr19 |  | CBK1 | Serine/threonine-protein kinase CBK1 | 3,353,511 | 3,360,410 | - | PF00069 | 3356960.5 |
| Gh_D08G2051 | chr24 |  | CBK1 | Serine/threonine-protein kinase CBK1 | 59,200,899 | 59,206,963 | + | PF00069 | 59203931 |
| Gh_A01G0842 | chr01 | 10 | CDL1 | Serine/threonine-protein kinase CDL1 | 19,326,732 | 19,329,911 | + | PF00069 | 19328321.5 |
| Gh_A03G1241 | chr03 |  | CDL1 | Serine/threonine-protein kinase CDL1 | 87,893,180 | 87,900,170 | + | PF00069 | 87896675 |
| Gh_A11G2389 | chr11 |  | CDL1 | Serine/threonine-protein kinase CDL1 | 81,711,818 | 81,713,830 | - | PF00069 | 81712824 |
| Gh_A12G0517 | chr12 |  | CDL1 | Serine/threonine-protein kinase CDL1 | 12,622,688 | 12,625,188 | + | PF00069 | 12623938 |
| Gh_A13G1528 | chr13 |  | CDL1 | Serine/threonine-protein kinase CDL1 | 73,872,476 | 73,874,354 | - | PF00069 | 73873415 |
| Gh_D01G0869 | chr15 |  | CDL1 | Serine/threonine-protein kinase CDL1 | 14,257,745 | 14,261,002 | + | PF00069 | 14259373.5 |
| Gh_D02G1680 | chr14 |  | CDL1 | Serine/threonine-protein kinase CDL1 | 57,936,029 | 57,938,164 | + | PF00069 | 57937096.5 |
| Gh_D04G1772 | chr22 |  | CDL1 | Serine/threonine-protein kinase CDL1 | 49,974,643 | 49,977,993 | - | PF00069 | 49976318 |
| Gh_D11G2704 | chr21 |  | CDL1 | Serine/threonine-protein kinase CDL1 | 56,373,217 | 56,375,219 | - | PF00069 | 56374218 |
| Gh_D13G1859 | chr18 |  | CDL1 | Serine/threonine-protein kinase CDL1 | 53,727,747 | 53,729,620 | - | PF00069 | 53728683.5 |
| Gh_A01G0730 | chr01 | 16 | D6PKL2 | Serine/threonine-protein kinase D6PKL2 | 14,103,942 | 14,105,839 | - | PF00069 | 14104890.5 |
| Gh_A05G3152 | chr05 |  | D6PKL2 | Serine/threonine-protein kinase D6PKL2 | 81,851,585 | 81,853,597 | - | PF00069 | 81852591 |
| Gh_A05G3420 | chr05 |  | D6PKL2 | Serine/threonine-protein kinase D6PKL2 | 89,180,213 | 89,184,146 | + | PF00069 | 89182179.5 |
| Gh_A06G1729 | chr06 |  | D6PKL2 | Serine/threonine-protein kinase D6PKL2 | 102,491,116 | 102,493,036 | + | PF00069 | 102492076 |
| Gh_A07G0985 | chr07 |  | D6PKL2 | Serine/threonine-protein kinase D6PKL2 | 18,734,164 | 18,737,741 | + | PF00069 | 18735952.5 |
| Gh_A08G1247 | chr08 |  | D6PKL2 | Serine/threonine-protein kinase D6PKL2 | 83,755,763 | 83,758,975 | - | PF00069 | 83757369 |
| Gh_A09G0893 | chr09 |  | D6PKL2 | Serine/threonine-protein kinase D6PKL2 | 57,349,149 | 57,352,284 | + | PF00069 | 57350716.5 |
| Gh_A13G1106 | chr13 |  | D6PKL2 | Serine/threonine-protein kinase D6PKL2 | 61,653,551 | 61,655,990 | + | PF00069 | 61654770.5 |
| Gh_D01G0750 | chr15 |  | D6PKL2 | Serine/threonine-protein kinase D6PKL2 | 10,831,070 | 10,832,964 | - | PF00069 | 10832017 |
| Gh_D04G0170 | chr22 |  | D6PKL2 | Serine/threonine-protein kinase D6PKL2 | 2,449,510 | 2,453,494 | - | PF00069 | 2451502 |
| Gh_D04G0480 | chr22 |  | D6PKL2 | Serine/threonine-protein kinase D6PKL2 | 7,924,247 | 7,926,264 | + | PF00069 | 7925255.5 |
| Gh_D06G2249 | chr25 |  | D6PKL2 | Serine/threonine-protein kinase D6PKL2 | 64,001,102 | 64,003,021 | - | PF00069 | 64002061.5 |
| Gh_D07G1063 | chr16 |  | D6PKL2 | Serine/threonine-protein kinase D6PKL2 | 15,100,883 | 15,104,560 | + | PF00069 | 15102721.5 |
| Gh_D08G1537 | chr24 |  | D6PKL2 | Serine/threonine-protein kinase D6PKL2 | 49,265,263 | 49,268,362 | - | PF00069 | 49266812.5 |
| Gh_D09G0918 | chr23 |  | D6PKL2 | Serine/threonine-protein kinase D6PKL2 | 34,555,537 | 34,558,556 | + | PF00069 | 34557046.5 |
| Gh_D13G1374 | chr18 |  | D6PKL2 | Serine/threonine-protein kinase D6PKL2 | 43,028,787 | 43,031,227 | + | PF00069 | 43030007 |
| Gh_A03G0920 | chr03 | 4 | dst1 | Serine/threonine-protein kinase dst1 | 58,029,297 | 58,068,872 | + | PF00069 | 58049084.5 |
| Gh_A12G1469 | chr12 |  | dst1 | Serine/threonine-protein kinase dst1 | 73,989,650 | 73,998,202 | - | PF00069 | 73993926 |
| Gh_D02G1301 | chr14 |  | dst1 | Serine/threonine-protein kinase dst1 | 42,567,875 | 42,599,261 | + | PF00069 | 42583568 |
| Gh_D12G1597 | chr26 |  | dst1 | Serine/threonine-protein kinase dst1 | 47,186,333 | 47,195,143 | - | PF00069 | 47190738 |
| Gh_A05G1198 | chr05 | 19 | fray2 | Serine/threonine-protein kinase fray2 | 12,052,475 | 12,059,518 | - | PF00069 | 12055996.5 |
| Gh_A09G0260 | chr09 |  | fray2 | Serine/threonine-protein kinase fray2 | 8,066,244 | 8,075,963 | + | PF00069 | 8071103.5 |
| Gh_A10G0452 | chr10 |  | fray2 | Serine/threonine-protein kinase fray2 | 4,624,153 | 4,635,381 | + | PF00069 | 4629767 |
| Gh_A10G0452 | chr10 |  | fray2 | Serine/threonine-protein kinase fray2 | 4,624,153 | 4,635,381 | + | PF00069 | 4629767 |
| Gh_A10G0929 | chr10 |  | fray2 | Serine/threonine-protein kinase fray2 | 20,595,736 | 20,601,766 | - | PF00069 | 20598751 |
| Gh_A10G0929 | chr10 |  | fray2 | Serine/threonine-protein kinase fray2 | 20,595,736 | 20,601,766 | - | PF00069 | 20598751 |
| Gh_A11G0169 | chr11 |  | fray2 | Serine/threonine-protein kinase fray2 | 1,594,066 | 1,600,916 | + | PF00069 | 1597491 |
| Gh_A11G0621 | chr11 |  | fray2 | Serine/threonine-protein kinase fray2 | 5,958,585 | 5,981,010 | - | PF00069 | 5969797.5 |
| Gh_A12G2165 | chr12 |  | fray2 | Serine/threonine-protein kinase fray2 | 84,423,803 | 84,430,737 | - | PF00069 | 84427270 |
| Gh_A13G1292 | chr13 |  | fray2 | Serine/threonine-protein kinase fray2 | 67,470,869 | 67,472,056 | - | PF00069 | 67471462.5 |
| Gh_D05G1375 | chr19 |  | fray2 | Serine/threonine-protein kinase fray2 | 12,073,222 | 12,080,262 | - | PF00069 | 12076742 |
| Gh_D08G1180 | chr24 |  | fray2 | Serine/threonine-protein kinase fray2 | 37,768,759 | 37,775,539 | + | PF00069 | 37772149 |
| Gh_D09G0260 | chr23 |  | fray2 | Serine/threonine-protein kinase fray2 | 8,002,711 | 8,009,075 | + | PF00069 | 8005893 |
| Gh_D10G0469 | chr20 |  | fray2 | Serine/threonine-protein kinase fray2 | 4,453,648 | 4,459,660 | + | PF00069 | 4456654 |
| Gh_D10G0845 | chr20 |  | fray2 | Serine/threonine-protein kinase fray2 | 10,525,352 | 10,532,985 | - | PF00069 | 10529168.5 |
| Gh_D11G0179 | chr21 |  | fray2 | Serine/threonine-protein kinase fray2 | 1,602,618 | 1,609,464 | + | PF00069 | 1606041 |
| Gh_D11G0709 | chr21 |  | fray2 | Serine/threonine-protein kinase fray2 | 6,123,097 | 6,130,839 | - | PF00069 | 6126968 |
| Gh_D12G2342 | chr26 |  | fray2 | Serine/threonine-protein kinase fray2 | 56,491,515 | 56,498,401 | - | PF00069 | 56494958 |
| Gh_D13G1592 | chr18 |  | fray2 | Serine/threonine-protein kinase fray2 | 48,589,420 | 48,590,586 | - | PF00069 | 48590003 |
| Gh_A08G0085 | chr08 | 4 | GRIK2 | Serine/threonine-protein kinase GRIK2 | 756,097 | 760,011 | + | PF00069 | 758054 |
| Gh_D05G0750 | chr19 |  | GRIK2 | Serine/threonine-protein kinase GRIK2 | 6,143,073 | 6,145,995 | - | PF00069 | 6144534 |
| Gh_D07G1171 | chr16 |  | GRIK2 | Serine/threonine-protein kinase GRIK2 | 17,525,427 | 17,529,418 | + | PF00069 | 17527422.5 |
| Gh_D08G0126 | chr24 |  | GRIK2 | Serine/threonine-protein kinase GRIK2 | 1,067,848 | 1,071,689 | + | PF00069 | 1069768.5 |
| Gh_A03G0494 | chr03 | 1 | HT1 | Serine/threonine-protein kinase HT1 | 11,520,005 | 11,522,787 | + | PF00069 | 11521396 |
| Gh_A09G1287 | chr09 | 6 | KIPK | Serine/threonine-protein kinase KIPK | 65,282,432 | 65,285,551 | - | PF00069 | 65283991.5 |
| Gh_A11G2867 | chr11 |  | KIPK | Serine/threonine-protein kinase KIPK | 92,601,631 | 92,604,346 | + | PF00069 | 92602988.5 |
| Gh_D04G0991 | chr22 |  | KIPK | Serine/threonine-protein kinase KIPK | 30,645,985 | 30,649,131 | + | PF00069 | 30647558 |
| Gh_D05G0835 | chr19 |  | KIPK | Serine/threonine-protein kinase KIPK | 6,998,869 | 7,004,243 | + | PF00069 | 7001556 |
| Gh_D09G1330 | chr23 |  | KIPK | Serine/threonine-protein kinase KIPK | 40,490,915 | 40,494,036 | + | PF00069 | 40492475.5 |
| Gh_D11G3249 | chr21 |  | KIPK | Serine/threonine-protein kinase KIPK | 65,521,804 | 65,524,512 | + | PF00069 | 65523158 |
| Gh_A07G1806 | chr07 | 4 | MHK | Serine/threonine-protein kinase MHK | 72,968,276 | 72,974,657 | + | PF00069 | 72971466.5 |
| Gh_A11G1297 | chr11 |  | MHK | Serine/threonine-protein kinase MHK | 16,666,275 | 16,676,526 | - | PF00069 | 16671400.5 |
| Gh_D07G2011 | chr16 |  | MHK | Serine/threonine-protein kinase MHK | 49,800,247 | 49,812,068 | + | PF00069 | 49806157.5 |
| Gh_D11G1445 | chr21 |  | MHK | Serine/threonine-protein kinase MHK | 14,403,950 | 14,410,456 | - | PF00069 | 14407203 |
| Gh_A06G0347 | chr06 | 3 | mph1 | Serine/threonine-protein kinase mph1 | 5,374,944 | 5,378,215 | - | PF00069 | 5376579.5 |
| Gh_D05G2207 | chr19 |  | mph1 | Serine/threonine-protein kinase mph1 | 20,995,694 | 21,001,140 | - | PF00069 | 20998417 |
| Gh_D06G0379 | chr25 |  | mph1 | Serine/threonine-protein kinase mph1 | 5,202,318 | 5,206,875 | - | PF00069 | 5204596.5 |
| Gh_A08G0593 | chr08 | 20 | NEK2 | Serine/threonine-protein kinase Nek2 | 10,699,081 | 10,704,271 | - | PF00069 | 10701676 |
| Gh_A11G2297 | chr11 |  | NEK2 | Serine/threonine-protein kinase Nek2 | 79,088,452 | 79,092,801 | + | PF00069 | 79090626.5 |
| Gh_A12G0021 | chr12 |  | NEK2 | Serine/threonine-protein kinase Nek2 | 368,170 | 378,917 | - | PF00069 | 373543.5 |
| Gh_A13G1495 | chr13 |  | NEK2 | Serine/threonine-protein kinase Nek2 | 73,381,116 | 73,387,669 | + | PF00069 | 73384392.5 |
| Gh_D08G0686 | chr24 |  | NEK2 | Serine/threonine-protein kinase Nek2 | 9,432,516 | 9,437,709 | - | PF00069 | 9435112.5 |
| Gh_D11G2608 | chr21 |  | NEK2 | Serine/threonine-protein kinase Nek2 | 54,233,353 | 54,237,706 | + | PF00069 | 54235529.5 |
| Gh_D13G1824 | chr18 |  | NEK2 | Serine/threonine-protein kinase Nek2 | 53,316,988 | 53,320,721 | + | PF00069 | 53318854.5 |
| Gh_A07G1028 | chr07 |  | NEK5 | Serine/threonine-protein kinase Nek5 | 19,864,250 | 19,870,015 | - | PF00069 | 19867132.5 |
| Gh_A09G0932 | chr09 |  | NEK5 | Serine/threonine-protein kinase Nek5 | 58,241,730 | 58,249,141 | - | PF00069 | 58245435.5 |
| Gh_D05G0790 | chr19 |  | NEK5 | Serine/threonine-protein kinase Nek5 | 6,581,939 | 6,587,945 | + | PF00069 | 6584942 |
| Gh_D07G1105 | chr16 |  | NEK5 | Serine/threonine-protein kinase Nek5 | 15,970,600 | 15,976,377 | - | PF00069 | 15973488.5 |
| Gh_D09G0960 | chr23 |  | NEK5 | Serine/threonine-protein kinase Nek5 | 35,132,881 | 35,140,321 | - | PF00069 | 35136601 |
| Gh_D13G2315 | chr18 |  | NEK5 | Serine/threonine-protein kinase Nek5 | 59,126,926 | 59,132,412 | + | PF00069 | 59129669 |
| Gh_D13G2319 | chr18 |  | NEK5 | Serine/threonine-protein kinase Nek5 | 59,309,847 | 59,313,119 | + | PF00069 | 59311483 |
| Gh_A08G0117 | chr08 |  | NEK6 | Serine/threonine-protein kinase Nek6 | 1,031,008 | 1,047,041 | + | PF00069 | 1039024.5 |
| Gh_A09G1167 | chr09 |  | NEK6 | Serine/threonine-protein kinase Nek6 | 63,164,387 | 63,168,974 | - | PF00069 | 63166680.5 |
| Gh_D08G0162 | chr24 |  | NEK6 | Serine/threonine-protein kinase Nek6 | 1,486,621 | 1,492,671 | + | PF00069 | 1489646 |
| Gh_D09G1173 | chr23 |  | NEK6 | Serine/threonine-protein kinase Nek6 | 38,513,077 | 38,520,819 | - | PF00069 | 38516948 |
| Gh_A01G1666 | chr01 |  | NEK7 | Serine/threonine-protein kinase Nek7 | 95,207,256 | 95,218,115 | + | PF00069 | 95212685.5 |
| Gh_D01G1916 | chr15 |  | NEK7 | Serine/threonine-protein kinase Nek7 | 57,421,994 | 57,428,557 | + | PF00069 | 57425275.5 |
| Gh_D06G1026 | chr25 | 1 | OXSR1 | Serine/threonine-protein kinase OSR1 | 21,877,378 | 21,878,200 | - | PF00069 | 21877789 |
| Gh_A07G1798 | chr07 | 3 | OXI1 | Serine/threonine-protein kinase OXI1 | 72,824,405 | 72,825,837 | - | PF00069 | 72825121 |
| Gh_D07G1608 | chr16 |  | OXI1 | Serine/threonine-protein kinase OXI1 | 31,378,990 | 31,380,350 | - | PF00069 | 31379670 |
| Gh_D07G2002 | chr16 |  | OXI1 | Serine/threonine-protein kinase OXI1 | 49,665,743 | 49,667,148 | - | PF00069 | 49666445.5 |
| Gh_A01G0195 | chr01 | 26 | PBS1 | Serine/threonine-protein kinase PBS1 | 1,977,528 | 1,980,567 | - | PF00069 | 1979047.5 |
| Gh_A01G0607 | chr01 |  | PBS1 | Serine/threonine-protein kinase PBS1 | 10,949,030 | 10,950,941 | - | PF00069 | 10949985.5 |
| Gh_A09G0371 | chr09 |  | PBS1 | Serine/threonine-protein kinase PBS1 | 20,643,450 | 20,646,670 | + | PF00069 | 20645060 |
| Gh_A09G0599 | chr09 |  | PBS1 | Serine/threonine-protein kinase PBS1 | 48,180,544 | 48,185,529 | - | PF00069 | 48183036.5 |
| Gh_A09G2123 | chr09 |  | PBS1 | Serine/threonine-protein kinase PBS1 | 74,214,065 | 74,216,591 | + | PF00069 | 74215328 |
| Gh_A10G0136 | chr10 |  | PBS1 | Serine/threonine-protein kinase PBS1 | 1,140,092 | 1,143,498 | + | PF00069 | 1141795 |
| Gh_A10G0136 | chr10 |  | PBS1 | Serine/threonine-protein kinase PBS1 | 1,140,092 | 1,143,498 | + | PF00069 | 1141795 |
| Gh_A10G1372 | chr10 |  | PBS1 | Serine/threonine-protein kinase PBS1 | 72,808,957 | 72,811,853 | + | PF00069 | 72810405 |
| Gh_A10G1372 | chr10 |  | PBS1 | Serine/threonine-protein kinase PBS1 | 72,808,957 | 72,811,853 | + | PF00069 | 72810405 |
| Gh_A10G1428 | chr10 |  | PBS1 | Serine/threonine-protein kinase PBS1 | 76,992,334 | 76,996,340 | - | PF00069 | 76994337 |
| Gh_A10G1428 | chr10 |  | PBS1 | Serine/threonine-protein kinase PBS1 | 76,992,334 | 76,996,340 | - | PF00069 | 76994337 |
| Gh_A11G0581 | chr11 |  | PBS1 | Serine/threonine-protein kinase PBS1 | 5,526,202 | 5,528,583 | + | PF00069 | 5527392.5 |
| Gh_A11G2528 | chr11 |  | PBS1 | Serine/threonine-protein kinase PBS1 | 84,174,473 | 84,179,684 | + | PF00069 | 84177078.5 |
| Gh_D01G0203 | chr15 |  | PBS1 | Serine/threonine-protein kinase PBS1 | 1,722,442 | 1,726,729 | - | PF00069 | 1724585.5 |
| Gh_D01G0204 | chr15 |  | PBS1 | Serine/threonine-protein kinase PBS1 | 1,728,998 | 1,733,542 | - | PF00069 | 1731270 |
| Gh_D01G0620 | chr15 |  | PBS1 | Serine/threonine-protein kinase PBS1 | 8,739,921 | 8,741,797 | - | PF00069 | 8740859 |
| Gh_D01G1270 | chr15 |  | PBS1 | Serine/threonine-protein kinase PBS1 | 33,149,935 | 33,151,816 | + | PF00069 | 33150875.5 |
| Gh_D09G0393 | chr23 |  | PBS1 | Serine/threonine-protein kinase PBS1 | 14,685,202 | 14,688,491 | - | PF00069 | 14686846.5 |
| Gh_D09G0598 | chr23 |  | PBS1 | Serine/threonine-protein kinase PBS1 | 28,597,778 | 28,602,783 | - | PF00069 | 28600280.5 |
| Gh_D09G2328 | chr23 |  | PBS1 | Serine/threonine-protein kinase PBS1 | 50,307,778 | 50,310,311 | + | PF00069 | 50309044.5 |
| Gh_D10G0142 | chr20 |  | PBS1 | Serine/threonine-protein kinase PBS1 | 1,136,664 | 1,140,481 | + | PF00069 | 1138572.5 |
| Gh_D10G1093 | chr20 |  | PBS1 | Serine/threonine-protein kinase PBS1 | 17,444,019 | 17,446,887 | - | PF00069 | 17445453 |
| Gh_D10G1669 | chr20 |  | PBS1 | Serine/threonine-protein kinase PBS1 | 46,120,320 | 46,124,338 | - | PF00069 | 46122329 |
| Gh_D11G0666 | chr21 |  | PBS1 | Serine/threonine-protein kinase PBS1 | 5,785,103 | 5,787,771 | + | PF00069 | 5786437 |
| Gh_D11G2878 | chr21 |  | PBS1 | Serine/threonine-protein kinase PBS1 | 58,817,997 | 58,819,812 | + | PF00069 | 58818904.5 |
| Gh_D12G0531 | chr26 |  | PBS1 | Serine/threonine-protein kinase PBS1 | 9,721,146 | 9,723,631 | + | PF00069 | 9722388.5 |
| Gh_A12G0138 | chr12 | 4 | PEPKR2 | Serine/threonine-protein kinase PEPKR2 | 2,042,354 | 2,044,782 | + | PF00069 | 2043568 |
| Gh_D05G2131 | chr19 |  | PEPKR2 | Serine/threonine-protein kinase PEPKR2 | 19,949,019 | 19,951,531 | - | PF00069 | 19950275 |
| Gh_D06G0278 | chr25 |  | PEPKR2 | Serine/threonine-protein kinase PEPKR2 | 3,166,167 | 3,168,472 | - | PF00069 | 3167319.5 |
| Gh_D12G0152 | chr26 |  | PEPKR2 | Serine/threonine-protein kinase PEPKR2 | 1,941,763 | 1,944,196 | + | PF00069 | 1942979.5 |
| Gh_A10G2128 | chr10 | 5 | ppk15 | Serine/threonine-protein kinase ppk15 | 100,086,421 | 100,089,305 | - | PF00069 | 100087863 |
| Gh_A10G2128 | chr10 |  | ppk15 | Serine/threonine-protein kinase ppk15 | 100,086,421 | 100,089,305 | - | PF00069 | 100087863 |
| Gh_A11G2021 | chr11 |  | ppk15 | Serine/threonine-protein kinase ppk15 | 58,841,476 | 58,852,585 | - | PF00069 | 58847030.5 |
| Gh_A13G1373 | chr13 |  | ppk15 | Serine/threonine-protein kinase ppk15 | 69,884,107 | 69,895,526 | - | PF00069 | 69889816.5 |
| Gh_D13G1683 | chr18 |  | ppk15 | Serine/threonine-protein kinase ppk15 | 50,452,902 | 50,464,400 | - | PF00069 | 50458651 |
| Gh_D13G0738 | chr18 | 1 | Prpf4b | Serine/threonine-protein kinase PRP4 homolog | 11,599,231 | 11,604,269 | - | PF00069 | 11601750 |
| Gh_A02G0451 | Chr02 | 3 | RUK | Serine/threonine-protein kinase RUNKEL | 6,132,920 | 6,138,808 | + | PF00069 | 6135864 |
| Gh_D02G0503 | chr14 |  | RUK | Serine/threonine-protein kinase RUNKEL | 6,585,300 | 6,591,185 | + | PF00069 | 6588242.5 |
| Gh_D10G1651 | chr20 |  | RUK | Serine/threonine-protein kinase RUNKEL | 45,597,222 | 45,603,302 | + | PF00069 | 45600262 |
| Gh_A11G1858 | chr11 | 14 | SAPK1 | Serine/threonine-protein kinase SAPK1 | 45,306,099 | 45,308,154 | + | PF00069 | 45307126.5 |
| Gh_A02G0789 | Chr02 |  | SAPK2 | Serine/threonine-protein kinase SAPK2 | 16,299,920 | 16,301,838 | - | PF00069 | 16300879 |
| Gh_A05G1922 | chr05 |  | SAPK2 | Serine/threonine-protein kinase SAPK2 | 20,148,675 | 20,150,866 | + | PF00069 | 20149770.5 |
| Gh_A11G0474 | chr11 |  | SAPK2 | Serine/threonine-protein kinase SAPK2 | 4,550,580 | 4,552,694 | + | PF00069 | 4551637 |
| Gh_D02G0839 | chr14 |  | SAPK2 | Serine/threonine-protein kinase SAPK2 | 14,597,151 | 14,599,078 | - | PF00069 | 14598114.5 |
| Gh_D05G2155 | chr19 |  | SAPK2 | Serine/threonine-protein kinase SAPK2 | 20,185,702 | 20,187,904 | + | PF00069 | 20186803 |
| Gh_D11G0552 | chr21 |  | SAPK2 | Serine/threonine-protein kinase SAPK2 | 4,806,770 | 4,808,880 | + | PF00069 | 4807825 |
| Gh_D11G2149 | chr21 |  | SAPK2 | Serine/threonine-protein kinase SAPK2 | 32,580,911 | 32,582,968 | + | PF00069 | 32581939.5 |
| Gh_A08G1469 | chr08 |  | SAPK3 | Serine/threonine-protein kinase SAPK3 | 90,892,068 | 90,893,718 | + | PF00069 | 90892893 |
| Gh_D08G1765 | chr24 |  | SAPK3 | Serine/threonine-protein kinase SAPK3 | 54,052,159 | 54,054,485 | + | PF00069 | 54053322 |
| Gh_A10G1380 | chr10 |  | SAPK7 | Serine/threonine-protein kinase SAPK7 | 73,521,474 | 73,525,386 | + | PF00069 | 73523430 |
| Gh_A10G1380 | chr10 |  | SAPK7 | Serine/threonine-protein kinase SAPK7 | 73,521,474 | 73,525,386 | + | PF00069 | 73523430 |
| Gh_D10G1083 | chr20 |  | SAPK7 | Serine/threonine-protein kinase SAPK7 | 17,134,725 | 17,138,661 | - | PF00069 | 17136693 |
| Gh_D03G0174 | chr17 |  | SAPK8 | Serine/threonine-protein kinase SAPK8 | 1,297,257 | 1,297,878 | - | PF00069 | 1297567.5 |
| Gh_D05G0851 | chr19 | 1 | spk-1 | Serine/threonine-protein kinase spk-1 | 7,098,750 | 7,101,686 | - | PF00069 | 7100218 |
| Gh_A12G0247 | chr12 | 15 | SRK2B | Serine/threonine-protein kinase SRK2B | 3,776,901 | 3,779,651 | + | PF00069 | 3778276 |
| Gh_A13G0314 | chr13 |  | SRK2B | Serine/threonine-protein kinase SRK2B | 3,920,394 | 3,923,558 | - | PF00069 | 3921976 |
| Gh_D12G0247 | chr26 |  | SRK2B | Serine/threonine-protein kinase SRK2B | 3,272,445 | 3,275,139 | + | PF00069 | 3273792 |
| Gh_D13G0352 | chr18 |  | SRK2B | Serine/threonine-protein kinase SRK2B | 3,566,616 | 3,569,517 | - | PF00069 | 3568066.5 |
| Gh_A01G0057 | chr01 |  | SRK2E | Serine/threonine-protein kinase SRK2E | 447,830 | 450,835 | - | PF00069 | 449332.5 |
| Gh_A03G1684 | chr03 |  | SRK2E | Serine/threonine-protein kinase SRK2E | 98,135,397 | 98,138,978 | - | PF00069 | 98137187.5 |
| Gh_A11G1757 | chr11 |  | SRK2E | Serine/threonine-protein kinase SRK2E | 29,069,141 | 29,071,943 | - | PF00069 | 29070542 |
| Gh_D02G2104 | chr14 |  | SRK2E | Serine/threonine-protein kinase SRK2E | 65,195,858 | 65,199,438 | - | PF00069 | 65197648 |
| Gh_D02G1835 | chr14 |  | SRK2G | Serine/threonine-protein kinase SRK2G | 61,371,452 | 61,372,160 | - | PF00069 | 61371806 |
| Gh_D05G1382 | chr19 |  | SRK2G | Serine/threonine-protein kinase SRK2G | 12,127,766 | 12,129,761 | + | PF00069 | 12128763.5 |
| Gh_D07G0405 | chr16 |  | SRK2G | Serine/threonine-protein kinase SRK2G | 4,449,878 | 4,452,750 | - | PF00069 | 4451314 |
| Gh_D08G1246 | chr24 |  | SRK2H | Serine/threonine-protein kinase SRK2H | 40,595,319 | 40,595,681 | - | PF00069 | 40595500 |
| Gh_A12G0641 | chr12 |  | SRK2I | Serine/threonine-protein kinase SRK2I | 18,241,166 | 18,244,267 | - | PF00069 | 18242716.5 |
| Gh_D11G0489 | chr21 |  | SRK2I | Serine/threonine-protein kinase SRK2I | 4,160,991 | 4,163,929 | - | PF00069 | 4162460 |
| Gh_D12G0859 | chr26 |  | SRK2I | Serine/threonine-protein kinase SRK2I | 28,126,914 | 28,129,959 | + | PF00069 | 28128436.5 |
| Gh_A02G1710 | Chr02 | 4 | SRPK | Serine/threonine-protein kinase SRPK | 83,259,873 | 83,262,376 | - | PF00069 | 83261124.5 |
| Gh_A10G0892 | chr10 |  | SRPK | Serine/threonine-protein kinase SRPK | 19,305,188 | 19,306,760 | - | PF00069 | 19305974 |
| Gh_A10G0892 | chr10 |  | SRPK | Serine/threonine-protein kinase SRPK | 19,305,188 | 19,306,760 | - | PF00069 | 19305974 |
| Gh_D10G0857 | chr20 |  | SRPK | Serine/threonine-protein kinase SRPK | 10,814,404 | 10,815,976 | + | PF00069 | 10815190 |
| Gh_A12G1556 | chr12 | 4 | STN7 | Serine/threonine-protein kinase STN7, chloroplastic | 75,626,925 | 75,631,401 | + | PF00069 | 75629163 |
| Gh_D12G1659 | chr26 |  | STN7 | Serine/threonine-protein kinase STN7, chloroplastic | 48,147,834 | 48,159,145 | - | PF00069 | 48153489.5 |
| Gh_A02G0024 | Chr02 |  | STN8 | Serine/threonine-protein kinase STN8, chloroplastic | 147,617 | 150,195 | - | PF00069 | 148906 |
| Gh_D02G0038 | chr14 |  | STN8 | Serine/threonine-protein kinase STN8, chloroplastic | 193,649 | 196,213 | - | PF00069 | 194931 |
| Gh_A01G0443 | chr01 | 4 | svkA | Serine/threonine-protein kinase svkA | 7,081,664 | 7,089,822 | - | PF00069 | 7085743 |
| Gh_A13G1253 | chr13 |  | svkA | Serine/threonine-protein kinase svkA | 66,602,025 | 66,608,948 | + | PF00069 | 66605486.5 |
| Gh_D01G0453 | chr15 |  | svkA | Serine/threonine-protein kinase svkA | 5,401,264 | 5,409,445 | - | PF00069 | 5405354.5 |
| Gh_D05G3352 | chr19 |  | svkA | Serine/threonine-protein kinase svkA | 54,297,272 | 54,307,354 | - | PF00069 | 54302313 |
| Gh_A05G3551 | chr05 | 1 | TIO | Serine/threonine-protein kinase TIO | 91,208,249 | 91,218,049 | - | PF00069 | 91213149 |
| Gh_D05G0400 | chr19 | 1 | Tnni3k | Serine/threonine-protein kinase TNNI3K | 3,285,304 | 3,286,755 | - | PF00069 | 3286029.5 |
| Gh_A07G1048 | chr07 | 3 | TOUSLED | Serine/threonine-protein kinase TOUSLED | 20,753,470 | 20,762,204 | + | PF00069 | 20757837 |
| Gh_D07G1128 | chr16 |  | TOUSLED | Serine/threonine-protein kinase TOUSLED | 16,588,329 | 16,597,063 | + | PF00069 | 16592696 |
| Gh_D13G2312 | chr18 |  | TOUSLED | Serine/threonine-protein kinase TOUSLED | 59,089,512 | 59,098,021 | + | PF00069 | 59093766.5 |
| Gh_A02G0390 | Chr02 | 6 | trc | Serine/threonine-protein kinase tricorner | 4,974,014 | 4,977,919 | + | PF00069 | 4975966.5 |
| Gh_A05G1749 | chr05 |  | trc | Serine/threonine-protein kinase tricorner | 18,466,458 | 18,470,808 | - | PF00069 | 18468633 |
| Gh_D02G0443 | chr14 |  | trc | Serine/threonine-protein kinase tricorner | 5,821,536 | 5,825,453 | + | PF00069 | 5823494.5 |
| Gh_D05G1944 | chr19 |  | trc | Serine/threonine-protein kinase tricorner | 17,870,935 | 17,875,297 | - | PF00069 | 17873116 |
| Gh_D06G0148 | chr25 |  | trc | Serine/threonine-protein kinase tricorner | 1,463,047 | 1,467,231 | - | PF00069 | 1465139 |
| Gh_D09G2220 | chr23 |  | trc | Serine/threonine-protein kinase tricorner | 49,393,505 | 49,397,211 | - | PF00069 | 49395358 |
| Gh_A07G1140 | chr07 | 5 | UCNL | Serine/threonine-protein kinase UCNL | 24,703,720 | 24,704,952 | + | PF00069 | 24704336 |
| Gh_A08G0116 | chr08 |  | UCNL | Serine/threonine-protein kinase UCNL | 1,013,789 | 1,015,027 | - | PF00069 | 1014408 |
| Gh_D05G0690 | chr19 |  | UCNL | Serine/threonine-protein kinase UCNL | 5,611,428 | 5,612,672 | - | PF00069 | 5612050 |
| Gh_D07G1236 | chr16 |  | UCNL | Serine/threonine-protein kinase UCNL | 19,196,059 | 19,197,291 | + | PF00069 | 19196675 |
| Gh_D08G0161 | chr24 |  | UCNL | Serine/threonine-protein kinase UCNL | 1,471,521 | 1,472,759 | - | PF00069 | 1472140 |
| Gh_A05G2870 | chr05 | 4 | WAG1 | Serine/threonine-protein kinase WAG1 | 66,850,589 | 66,852,430 | + | PF00069 | 66851509.5 |
| Gh_D05G3179 | chr19 |  | WAG1 | Serine/threonine-protein kinase WAG1 | 48,877,410 | 48,878,882 | + | PF00069 | 48878146 |
| Gh_A01G0246 | chr01 |  | WAG2 | Serine/threonine-protein kinase WAG2 | 2,375,340 | 2,376,647 | - | PF00069 | 2375993.5 |
| Gh_D01G0244 | chr15 |  | WAG2 | Serine/threonine-protein kinase WAG2 | 2,124,303 | 2,125,610 | - | PF00069 | 2124956.5 |
| Gh_A02G0300 | Chr02 | 8 | WNK1 | Serine/threonine-protein kinase WNK1 | 3,580,344 | 3,583,100 | + | PF00069 | 3581722 |
| Gh_A11G2311 | chr11 |  | WNK1 | Serine/threonine-protein kinase WNK1 | 79,445,838 | 79,448,802 | + | PF00069 | 79447320 |
| Gh_D02G0364 | chr14 |  | WNK1 | Serine/threonine-protein kinase WNK1 | 4,843,486 | 4,846,222 | + | PF00069 | 4844854 |
| Gh_D11G2621 | chr21 |  | WNK1 | Serine/threonine-protein kinase WNK1 | 54,650,203 | 54,653,173 | + | PF00069 | 54651688 |
| Gh_A02G0963 | Chr02 |  | WNK8 | Serine/threonine-protein kinase WNK8 | 40,734,538 | 40,736,580 | + | PF00069 | 40735559 |
| Gh_A12G2159 | chr12 |  | WNK8 | Serine/threonine-protein kinase WNK8 | 84,396,273 | 84,399,203 | + | PF00069 | 84397738 |
| Gh_D03G0796 | chr17 |  | WNK8 | Serine/threonine-protein kinase WNK8 | 27,359,386 | 27,362,243 | + | PF00069 | 27360814.5 |
| Gh_D12G2336 | chr26 |  | WNK8 | Serine/threonine-protein kinase WNK8 | 56,462,404 | 56,465,331 | + | PF00069 | 56463867.5 |
| Gh_A01G0827 | chr01 | 2 | IRE1B | Serine/threonine-protein kinase/endoribonuclease IRE1b | 18,922,173 | 18,925,648 | + | PF00069 | 18923910.5 |
| Gh_D01G0855 | chr15 |  | IRE1B | Serine/threonine-protein kinase/endoribonuclease IRE1b | 14,005,193 | 14,008,660 | + | PF00069 | 14006926.5 |
| Gh_D05G2533 | chr19 | 4 | At1g28390 | Serine/threonine-protein kinase-like protein At1g28390 | 25,614,257 | 25,615,714 | + | PF00069 | 25614985.5 |
| Gh_A11G0665 | chr11 |  | At3g51990 | Serine/threonine-protein kinase-like protein At3g51990 | 6,535,421 | 6,536,755 | - | PF00069 | 6536088 |
| Gh_A01G1533 | chr01 |  | At5g23170 | Serine/threonine-protein kinase-like protein At5g23170 | 91,449,874 | 91,450,950 | - | PF00069 | 91450412 |
| Gh_D01G1782 | chr15 |  | At5g23170 | Serine/threonine-protein kinase-like protein At5g23170 | 54,841,997 | 54,843,076 | - | PF00069 | 54842536.5 |
| Gh_D09G1411 | chr23 | 4 | CCR1 | Serine/threonine-protein kinase-like protein CCR1 | 41,665,916 | 41,667,895 | + | PF00069 | 41666905.5 |
| Gh_A05G2557 | chr05 |  | CCR2 | Serine/threonine-protein kinase-like protein CCR2 | 36,151,160 | 36,153,469 | - | PF00069 | 36152314.5 |
| Gh_D05G2835 | chr19 |  | CCR2 | Serine/threonine-protein kinase-like protein CCR2 | 31,877,753 | 31,880,062 | - | PF00069 | 31878907.5 |
| Gh_A05G2138 | chr05 |  | CCR4 | Serine/threonine-protein kinase-like protein CCR4 | 24,392,723 | 24,394,747 | + | PF00069 | 24393735 |
| Gh_D11G2830 | chr21 | 14 | ASK1 | Shaggy-related protein kinase alpha | 58,071,261 | 58,074,075 | - | PF00069 | 58072668 |
| Gh_A09G0712 | chr09 |  | ASK7 | Shaggy-related protein kinase eta | 52,841,598 | 52,844,894 | + | PF00069 | 52843246 |
| Gh_A09G0713 | chr09 |  | ASK7 | Shaggy-related protein kinase eta | 52,861,382 | 52,864,569 | + | PF00069 | 52862975.5 |
| Gh_D06G2142 | chr25 |  | ASK7 | Shaggy-related protein kinase eta | 63,261,021 | 63,263,915 | - | PF00069 | 63262468 |
| Gh_A01G1558 | chr01 |  | ASK10 | Shaggy-related protein kinase kappa | 92,410,497 | 92,413,873 | + | PF00069 | 92412185 |
| Gh_A12G0411 | chr12 |  | ASK10 | Shaggy-related protein kinase kappa | 8,244,999 | 8,248,430 | - | PF00069 | 8246714.5 |
| Gh_D01G1809 | chr15 |  | ASK10 | Shaggy-related protein kinase kappa | 55,472,543 | 55,475,936 | + | PF00069 | 55474239.5 |
| Gh_D12G0407 | chr26 |  | ASK10 | Shaggy-related protein kinase kappa | 6,533,937 | 6,537,332 | - | PF00069 | 6535634.5 |
| Gh_A08G0285 | chr08 |  | ASK8 | Shaggy-related protein kinase theta | 3,329,052 | 3,334,479 | - | PF00069 | 3331765.5 |
| Gh_A08G1158 | chr08 |  | ASK8 | Shaggy-related protein kinase theta | 81,021,333 | 81,025,764 | + | PF00069 | 81023548.5 |
| Gh_A11G0778 | chr11 |  | ASK8 | Shaggy-related protein kinase theta | 7,649,123 | 7,654,545 | + | PF00069 | 7651834 |
| Gh_D08G0378 | chr24 |  | ASK8 | Shaggy-related protein kinase theta | 3,871,236 | 3,880,133 | - | PF00069 | 3875684.5 |
| Gh_D08G1440 | chr24 |  | ASK8 | Shaggy-related protein kinase theta | 47,361,120 | 47,366,590 | + | PF00069 | 47363855 |
| Gh_D11G0907 | chr21 |  | ASK8 | Shaggy-related protein kinase theta | 7,839,230 | 7,844,650 | + | PF00069 | 7841940 |
