# Supplementary material for: GBS Mapping and Analysis of Genes Conserved between Gossypium tomentosum and Gossypium hirsutum Cotton Cultivars that Respond to Drought Stress at the Seedling Stage of the BC2F2 Generation
Source: Int J Mol Sci. 2018 May 30;19(6):1614. doi: 10.3390/ijms19061614 (PMC6032168; doi:10.3390/ijms19061614)
Supplement: Supplementary file 1 [file ijms-19-01614-s001.zip › Supplimentary/Supplementary Table 6 miRNA targets.docx]

Supplementary Table 6: miRNA targets

| miRNA_Acc. | Target_Acc. | Totals | Expectation | miRNA_start | miRNA_end | Target_start | Target_end | miRNA_aligned_fragment | Target_aligned_fragment | Inhibition |
| --- | --- | --- | --- | --- | --- | --- | --- | --- | --- | --- |
| ghr-miR156a | Gh_D11G0594 | 1 | 4.5 | 1 | 20 | 207 | 226 | UGACAGAAGAGAGUGAGCAC | UAAUUUUCUCUCUUCUGUUA | Cleavage |
| ghr-miR156c | Gh_A02G0451 | 5 | 3.5 | 1 | 20 | 698 | 717 | UGUCAGAAGAGAGUGAGCAC | UUGUUAAUUCUCUUCUGAUU | Cleavage |
| ghr-miR156c | Gh_A03G0672 |  | 4.5 | 1 | 20 | 935 | 954 | UGUCAGAAGAGAGUGAGCAC | AAGCUCACUCUUUACUGAAA | Cleavage |
| ghr-miR156c | Gh_D01G0203 |  | 5 | 1 | 20 | 1070 | 1089 | UGUCAGAAGAGAGUGAGCAC | CUGCUCUCUCGUAUCUGGCA | Translation |
| ghr-miR156c | Gh_D02G0503 |  | 3.5 | 1 | 20 | 698 | 717 | UGUCAGAAGAGAGUGAGCAC | UUGUUAAUUCUCUUCUGAUU | Cleavage |
| ghr-miR156c | Gh_D03G0837 |  | 4.5 | 1 | 20 | 1049 | 1068 | UGUCAGAAGAGAGUGAGCAC | AAGCUCACUCUUUACUGAAA | Cleavage |
| ghr-miR160 | Gh_A11G1297 | 1 | 5 | 1 | 21 | 446 | 466 | UAUGAGGAGCCAUGCAUGUAU | UAUCAUCAAUGCCUCCUUAUA | Translation |
| ghr-miR164 | Gh_A01G1666 | 6 | 4.5 | 1 | 21 | 1860 | 1880 | UGGAGAAGCAGGGCACGUGCA | ACUAUGUGCCCAGCUUCUUAA | Translation |
| ghr-miR164 | Gh_A09G1167 |  | 4.5 | 1 | 21 | 1821 | 1841 | UGGAGAAGCAGGGCACGUGCA | GCUAUGUGCCCGGCUUCUUAA | Translation |
| ghr-miR164 | Gh_D01G1916 |  | 4.5 | 1 | 21 | 1821 | 1841 | UGGAGAAGCAGGGCACGUGCA | GCUAUGUGCCCAGCUUCUUAA | Translation |
| ghr-miR164 | Gh_D08G0686 |  | 4.5 | 1 | 21 | 1438 | 1458 | UGGAGAAGCAGGGCACGUGCA | GCGAAGUGCUCUUCUUCUUCA | Cleavage |
| ghr-miR164 | Gh_D09G1173 |  | 4.5 | 1 | 21 | 1926 | 1946 | UGGAGAAGCAGGGCACGUGCA | GCUAUGUGCCCGGCUUCUUAA | Translation |
| ghr-miR164 | Gh_D10G1380 |  | 5 | 1 | 21 | 888 | 908 | UGGAGAAGCAGGGCACGUGCA | GGUGUCUGGUCUGCUUCUUCA | Cleavage |
| ghr-miR167a | Gh_A05G2375 | 4 | 5 | 1 | 21 | 1114 | 1134 | UGAAGCUGCCAGCAUGAUCUA | AAGAUUAAGUUGCCAGCUUUU | Cleavage |
| ghr-miR167a | Gh_D05G2640 |  | 4.5 | 1 | 21 | 1114 | 1134 | UGAAGCUGCCAGCAUGAUCUA | AAGAUUAAGCUGCCAGCUUUU | Cleavage |
| ghr-miR167a | Gh_D06G0278 |  | 5 | 1 | 21 | 706 | 726 | UGAAGCUGCCAGCAUGAUCUA | AAGAUAAAGCUGGCAGAUUUU | Cleavage |
| ghr-miR167a | Gh_D07G0405 |  | 5 | 1 | 21 | 266 | 286 | UGAAGCUGCCAGCAUGAUCUA | UUUGUAGUGCUGGUAGAUUCA | Cleavage |
| ghr-miR169a | Gh_A05G3152 | 3 | 5 | 1 | 21 | 1484 | 1504 | UAGCCAAGGAUGACUUGCCUG | CAUUUAAGUCAUCCUUGCCUU | Cleavage |
| ghr-miR169a | Gh_A10G2128 |  | 4.5 | 1 | 21 | 1698 | 1718 | UAGCCAAGGAUGACUUGCCUG | CCCGGAAGUCAUCCUUGGCCU | Cleavage |
| ghr-miR169a | Gh_D04G0480 |  | 5 | 1 | 21 | 1484 | 1504 | UAGCCAAGGAUGACUUGCCUG | CAUUUAAGUCAUCCUUGCCUU | Cleavage |
| ghr-miR2948-5p | Gh_A09G1287 | 4 | 4.5 | 1 | 22 | 1922 | 1943 | UGUGGGAGAGUUGGGCAAGAAU | CAGUUAGUCCAACUCUCCUGAA | Cleavage |
| ghr-miR2948-5p | Gh_A10G0929 |  | 4.5 | 1 | 22 | 1733 | 1754 | UGUGGGAGAGUUGGGCAAGAAU | CAGCUAGUGCAAUUCUCCCAUC | Cleavage |
| ghr-miR2948-5p | Gh_D04G0991 |  | 4.5 | 1 | 22 | 1910 | 1931 | UGUGGGAGAGUUGGGCAAGAAU | CAGUUAGUCCAACUCUCCUGAA | Cleavage |
| ghr-miR2948-5p | Gh_D09G1330 |  | 4.5 | 1 | 22 | 1922 | 1943 | UGUGGGAGAGUUGGGCAAGAAU | CAGUUAGUCCAACUCUCCUGAA | Cleavage |
| ghr-miR2949a-3p | Gh_D05G1382 | 3 | 4.5 | 1 | 21 | 229 | 250 | UGCAAAUCCAGUCAAA-AGUUA | GAGCUCUUUGAUCGGAUUUGCA | Translation |
| ghr-miR2949a-5p | Gh_A06G0347 |  | 4.5 | 1 | 22 | 597 | 618 | ACUUUUGAACUGGAUUUGCCGA | UAAGAAAAUCAAGCUCAAAGGU | Cleavage |
| ghr-miR2949a-5p | Gh_D06G0379 |  | 4.5 | 1 | 22 | 1386 | 1407 | ACUUUUGAACUGGAUUUGCCGA | UAAGAAAAUCAAGCUCAAAGGU | Cleavage |
| ghr-miR2949b | Gh_A02G0963 | 7 | 4.5 | 1 | 22 | 1034 | 1055 | UCUUUUGAACUGGAUUUGCCGA | AUAACGAAUUCAGGUUAAAAGG | Cleavage |
| ghr-miR2949b | Gh_A05G3371 |  | 4.5 | 1 | 22 | 1295 | 1316 | UCUUUUGAACUGGAUUUGCCGA | AUGGAAGUUCCCGUUCAAGAGA | Translation |
| ghr-miR2949b | Gh_A08G0117 |  | 5 | 1 | 22 | 1408 | 1429 | UCUUUUGAACUGGAUUUGCCGA | CUACAAGAUCUGGUUCCAAAGA | Cleavage |
| ghr-miR2949b | Gh_A11G1210 |  | 4.5 | 1 | 22 | 1591 | 1612 | UCUUUUGAACUGGAUUUGCCGA | GCUGCACAGCCUGUUCAAGGGA | Translation |
| ghr-miR2949b | Gh_D03G0796 |  | 4.5 | 1 | 22 | 1034 | 1055 | UCUUUUGAACUGGAUUUGCCGA | AUAACGAAUUCAGGUUAAAAGG | Cleavage |
| ghr-miR2949b | Gh_D04G0266 |  | 4.5 | 1 | 22 | 863 | 884 | UCUUUUGAACUGGAUUUGCCGA | AUGGAAGUUCCCGUUCAAGAGA | Translation |
| ghr-miR2949b | Gh_D13G1592 |  | 5 | 1 | 22 | 485 | 506 | UCUUUUGAACUGGAUUUGCCGA | CUUGCAUGUUCGAUUCAGGAGA | Translation |
| ghr-miR2950 | Gh_D02G1046 | 1 | 4.5 | 1 | 21 | 1637 | 1657 | UGGUGUGCAGGGGGUGGAAUA | CUUUCCUUCCUUUGCGUAUUA | Cleavage |
| ghr-miR3476-3p | Gh_A11G0665 | 5 | 4 | 1 | 21 | 686 | 706 | AGCCAACAACAUCAGUUCUAA | CAAAAACUGAUGUUUUUAGCU | Cleavage |
| ghr-miR3476-3p | Gh_D11G0709 |  | 4 | 1 | 21 | 1094 | 1114 | AGCCAACAACAUCAGUUCUAA | GCAGCAAUGGUGUUGUUGGAU | Cleavage |
| ghr-miR3476-5p | Gh_A13G0755 |  | 5 | 1 | 21 | 348 | 368 | UGAACUGGGUUUGUUGGCUGC | ACAGCAUGCAAAUCUAGUUAA | Cleavage |
| ghr-miR3476-5p | Gh_D05G2131 |  | 5 | 1 | 21 | 46 | 66 | UGAACUGGGUUUGUUGGCUGC | GCAACCAACAAAUUUGCUUCG | Cleavage |
| ghr-miR3476-5p | Gh_D13G0942 |  | 5 | 1 | 21 | 348 | 368 | UGAACUGGGUUUGUUGGCUGC | ACAGCAUGCAAAUCUAGUUAA | Cleavage |
| ghr-miR390a | Gh_A05G2870 | 19 | 4 | 1 | 21 | 859 | 879 | AAGCUCAGGAGGGAUAGCGCC | GACGAAGUUCUUCCUGAGUUU | Cleavage |
| ghr-miR390a | Gh_A07G1028 |  | 3.5 | 1 | 21 | 1627 | 1647 | AAGCUCAGGAGGGAUAGCGCC | GAUGCUAUUCCUCCUCAGCUG | Cleavage |
| ghr-miR390a | Gh_A09G2123 |  | 4 | 1 | 21 | 847 | 867 | AAGCUCAGGAGGGAUAGCGCC | GGUGUGGUCUUUCUUGAGCUU | Cleavage |
| ghr-miR390a | Gh_A10G1372 |  | 4.5 | 1 | 21 | 649 | 669 | AAGCUCAGGAGGGAUAGCGCC | CACGUUAGCCCUCCUGUGAUU | Cleavage |
| ghr-miR390a | Gh_A11G0169 |  | 4 | 1 | 21 | 838 | 858 | AAGCUCAGGAGGGAUAGCGCC | CAUGCAAAACCUCCUGAGCUU | Cleavage |
| ghr-miR390a | Gh_A12G1556 |  | 5 | 1 | 21 | 445 | 465 | AAGCUCAGGAGGGAUAGCGCC | CCAGGUGUCGCUCCUGGGUUU | Cleavage |
| ghr-miR390a | Gh_A12G2165 |  | 5 | 1 | 21 | 829 | 849 | AAGCUCAGGAGGGAUAGCGCC | CAUGCAAAGCCUCCUGAGCUC | Cleavage |
| ghr-miR390a | Gh_A13G1292 |  | 4 | 1 | 21 | 820 | 840 | AAGCUCAGGAGGGAUAGCGCC | CAUGCCAAACCUCCUGAGCUU | Cleavage |
| ghr-miR390a | Gh_D02G1680 |  | 5 | 1 | 21 | 826 | 846 | AAGCUCAGGAGGGAUAGCGCC | GGAGUUGUUCUUCUAGAGCUU | Cleavage |
| ghr-miR390a | Gh_D04G1206 |  | 4.5 | 1 | 21 | 598 | 618 | AAGCUCAGGAGGGAUAGCGCC | GGUGCUAUUUUUUUUCAGCUU | Cleavage |
| ghr-miR390a | Gh_D07G1105 |  | 3.5 | 1 | 21 | 1627 | 1647 | AAGCUCAGGAGGGAUAGCGCC | GAUGCUAUUCCUCCUCAGCUG | Cleavage |
| ghr-miR390a | Gh_D08G1180 |  | 4 | 1 | 21 | 838 | 858 | AAGCUCAGGAGGGAUAGCGCC | CAUGCAAAGCCUCCUGAGCUU | Cleavage |
| ghr-miR390a | Gh_D09G2328 |  | 4 | 1 | 21 | 847 | 867 | AAGCUCAGGAGGGAUAGCGCC | GGUGUGGUCUUUCUUGAGCUU | Cleavage |
| ghr-miR390a | Gh_D10G1093 |  | 4.5 | 1 | 21 | 649 | 669 | AAGCUCAGGAGGGAUAGCGCC | CACGUUAGCCCUCCUGUGAUU | Cleavage |
| ghr-miR390a | Gh_D10G1669 |  | 4.5 | 1 | 21 | 841 | 861 | AAGCUCAGGAGGGAUAGCGCC | GGGGUAGUCUUUCUUGAGCUU | Cleavage |
| ghr-miR390a | Gh_D11G0179 |  | 4 | 1 | 21 | 838 | 858 | AAGCUCAGGAGGGAUAGCGCC | CAUGCAAAACCUCCUGAGCUU | Cleavage |
| ghr-miR390a | Gh_D12G0407 |  | 4.5 | 1 | 21 | 1186 | 1206 | AAGCUCAGGAGGGAUAGCGCC | UCUGGUAUCCCUCCUGAAGUU | Cleavage |
| ghr-miR390a | Gh_D12G1659 |  | 5 | 1 | 21 | 397 | 417 | AAGCUCAGGAGGGAUAGCGCC | CCAGGUGUCGCUCCUGGGUUU | Cleavage |
| ghr-miR390a | Gh_D12G2342 |  | 5 | 1 | 21 | 829 | 849 | AAGCUCAGGAGGGAUAGCGCC | CAUGCAAAGCCUCCUGAGCUC | Cleavage |
| ghr-miR393 | Gh_A12G0517 | 4 | 4.5 | 1 | 22 | 1033 | 1054 | UCCAAAGGGAUCGCAUUGAUCU | CCCUCAAUCCCAUUCCUUUGCA | Cleavage |
| ghr-miR393 | Gh_D04G1772 |  | 4 | 1 | 22 | 764 | 785 | UCCAAAGGGAUCGCAUUGAUCU | CAUUCGGAGUGGUUCUUUUGGA | Cleavage |
| ghr-miR393 | Gh_D05G2207 |  | 4 | 1 | 22 | 437 | 458 | UCCAAAGGGAUCGCAUUGAUCU | AGAAUAAUCCGAUCCCUUUGCA | Cleavage |
| ghr-miR393 | Gh_D12G0531 |  | 4.5 | 1 | 22 | 1030 | 1051 | UCCAAAGGGAUCGCAUUGAUCU | CCCUCAAUCCCAUUCCUUUGCA | Cleavage |
| ghr-miR396a | Gh_A07G1048 | 3 | 4.5 | 1 | 21 | 361 | 381 | UUCCACAGCUUUCUUGAACUG | AAGAUUGUGGAAGCUGUGGAU | Cleavage |
| ghr-miR396a | Gh_D05G0400 |  | 5 | 1 | 21 | 940 | 960 | UUCCACAGCUUUCUUGAACUG | AGAUUGAAUAAAGCCGUGGAA | Cleavage |
| ghr-miR396a | Gh_D05G0835 |  | 4.5 | 1 | 21 | 739 | 759 | UUCCACAGCUUUCUUGAACUG | GCUGUCAAGGAAGCUUUGGAG | Cleavage |
| ghr-miR399a | Gh_A11G2021 | 1 | 4 | 1 | 21 | 1142 | 1162 | CGCCAAUGGAGAUUUGUCCGG | AUGGUGAAAUCUCCAGUGGUG | Cleavage |
| ghr-miR479 | Gh_A11G2867 | 1 | 5 | 1 | 22 | 636 | 657 | CGUGAUAUUGGUUCGGCUCAUC | AUCGAGUCGAACUGAUGUUUCU | Cleavage |
| ghr-miR482a | Gh_A10G1428 | 4 | 4.5 | 1 | 22 | 50 | 71 | UCUUUCCUACUCCUCCCAUACC | AUGGUGGGGGCAGUGUGAAAGA | Cleavage |
| ghr-miR482a | Gh_A11G1858 |  | 4.5 | 1 | 22 | 802 | 823 | UCUUUCCUACUCCUCCCAUACC | UUCAUGGGAGGAGGGGGAGGAA | Cleavage |
| ghr-miR482a | Gh_D05G0690 |  | 4.5 | 1 | 22 | 869 | 890 | UCUUUCCUACUCCUCCCAUACC | UCAAAGGGAAGAGCAGGAAGGA | Cleavage |
| ghr-miR482a | Gh_D11G2149 |  | 4 | 1 | 22 | 802 | 822 | UCUUUCCUACUCCUCCCAUACC | UUCAUGGGAGGAG-AGGGAGGA | Cleavage |
| ghr-miR482b | Gh_D05G3179 | 1 | 5 | 1 | 22 | 1276 | 1297 | UCUUGCCUACUCCACCCAUGCC | GUGGUGGGUGGGGUGGCGAGGA | Cleavage |
| ghr-miR7484a | Gh_A05G2557 | 7 | 4 | 1 | 24 | 1558 | 1581 | UUUGUAUAUUAGAUCAAAGAGCAA | GGGAGCUUCGGUUUCGUAUACAAA | Translation |
| ghr-miR7484a | Gh_A13G1373 |  | 4 | 1 | 24 | 2177 | 2200 | UUUGUAUAUUAGAUCAAAGAGCAA | UAGGACUUGGUUCUAAUGUGCAAC | Cleavage |
| ghr-miR7484a | Gh_A13G1528 |  | 5 | 1 | 24 | 448 | 471 | UUUGUAUAUUAGAUCAAAGAGCAA | GGAGGGUUGGGUGUAAUGUAUAAA | Cleavage |
| ghr-miR7484a | Gh_D02G1835 |  | 5 | 1 | 24 | 250 | 273 | UUUGUAUAUUAGAUCAAAGAGCAA | GAGCUCUUUGAUCGAAUUUGCAGU | Translation |
| ghr-miR7484a | Gh_D05G2835 |  | 4 | 1 | 24 | 1558 | 1581 | UUUGUAUAUUAGAUCAAAGAGCAA | GGGAGCUUCGGUUUUGUAUACAAA | Translation |
| ghr-miR7484a | Gh_D13G1683 |  | 5 | 1 | 24 | 2177 | 2200 | UUUGUAUAUUAGAUCAAAGAGCAA | UAGGAGUUGGUUCUAAUGUGCAAC | Cleavage |
| ghr-miR7484a | Gh_D13G1859 |  | 5 | 1 | 24 | 448 | 471 | UUUGUAUAUUAGAUCAAAGAGCAA | GGAGGGUUGGGUGUAAUGUAUAAA | Cleavage |
| ghr-miR7485 | Gh_A11G0621 | 4 | 3.5 | 1 | 24 | 3321 | 3344 | AAAGACAUCUUUGAAUUCUUGGAG | UGUGGAUGAUUUAAAGAUGUCUGU | Cleavage |
| ghr-miR7485 | Gh_A11G2297 |  | 5 | 1 | 24 | 387 | 410 | AAAGACAUCUUUGAAUUCUUGGAG | UGUCAAGUGUUCAAACAUCUUUUU | Cleavage |
| ghr-miR7485 | Gh_A12G0021 |  | 5 | 1 | 24 | 387 | 410 | AAAGACAUCUUUGAAUUCUUGGAG | UGUCAAGUGUUCAAAUAUAUUUUU | Cleavage |
| ghr-miR7485 | Gh_D11G2608 |  | 5 | 1 | 24 | 387 | 410 | AAAGACAUCUUUGAAUUCUUGGAG | UGUCAAGUGUUCAAACAUCUUUUU | Cleavage |
| ghr-miR7486a | Gh_A08G0116 | 2 | 4 | 1 | 24 | 1203 | 1226 | AAGGAAGCGCUUUGUCCACGUGGA | GGAGUAUAGACGGAGCGUUUCUUU | Cleavage |
| ghr-miR7486a | Gh_D08G0161 |  | 4 | 1 | 24 | 1203 | 1226 | AAGGAAGCGCUUUGUCCACGUGGA | GGAGUAUAGACGGAGCGUUUCUUU | Cleavage |
| ghr-miR7489 | Gh_A09G0932 | 3 | 3.5 | 1 | 24 | 2797 | 2820 | AUUGUUGCCAAUACAGGAGAACGU | AGUAGCUUCUGUGUUGGCAGUGAG | Cleavage |
| ghr-miR7489 | Gh_D05G0790 |  | 4.5 | 1 | 24 | 2689 | 2712 | AUUGUUGCCAAUACAGGAGAACGU | AGUAGCUUCCGUGUUGGCAGUGAG | Cleavage |
| ghr-miR7489 | Gh_D09G0960 |  | 3.5 | 1 | 24 | 2827 | 2850 | AUUGUUGCCAAUACAGGAGAACGU | AGUAGCUUCUGUGUUGGCAGUGAG | Cleavage |
| ghr-miR7490 | Gh_D07G1128 | 1 | 4.5 | 1 | 24 | 1249 | 1272 | AGUCUAGAAAACUUCACUGACGGU | GGAUUCAGUGAGGUUUAUAAGGCU | Cleavage |
| ghr-miR7491 | Gh_A01G0057 | 21 | 4 | 1 | 24 | 638 | 661 | UGGGAUCUUCGAGAGGAUUGAGCC | GAGCAUAUCCGUUCGAAGACCCCA | Cleavage |
| ghr-miR7491 | Gh_A01G0443 |  | 5 | 1 | 24 | 1254 | 1277 | UGGGAUCUUCGAGAGGAUUGAGCC | CGUAAGGUCUCCUAGAGGAUCUCA | Translation |
| ghr-miR7491 | Gh_A01G0730 |  | 4.5 | 1 | 24 | 1621 | 1644 | UGGGAUCUUCGAGAGGAUUGAGCC | UGUGCAAGCCCCCCGGAGAUUCCA | Cleavage |
| ghr-miR7491 | Gh_A02G0789 |  | 3.5 | 1 | 24 | 572 | 595 | UGGGAUCUUCGAGAGGAUUGAGCC | GGGCAUAUCCCUUUGAAGAUCCUA | Cleavage |
| ghr-miR7491 | Gh_A03G0920 |  | 5 | 1 | 24 | 1553 | 1576 | UGGGAUCUUCGAGAGGAUUGAGCC | GACUUAAUCCACUGGAAGGUUCAA | Translation |
| ghr-miR7491 | Gh_A03G1684 |  | 4 | 1 | 24 | 644 | 667 | UGGGAUCUUCGAGAGGAUUGAGCC | GAGCAUAUCCUUUUGAAGACCCCG | Cleavage |
| ghr-miR7491 | Gh_A05G1922 |  | 3 | 1 | 24 | 587 | 610 | UGGGAUCUUCGAGAGGAUUGAGCC | GGGCAUAUCCUUUUGAAGAUCCUG | Cleavage |
| ghr-miR7491 | Gh_A08G1469 |  | 4.5 | 1 | 24 | 545 | 568 | UGGGAUCUUCGAGAGGAUUGAGCC | GAGCAUACCCUUUUGAGGAUCCUG | Cleavage |
| ghr-miR7491 | Gh_A11G0474 |  | 4 | 1 | 24 | 588 | 610 | UGGGAUCUUCGAGAGGAUUGAGCC | GGCUUA-UCCUUUUGAAGAUCCUG | Cleavage |
| ghr-miR7491 | Gh_A11G1757 |  | 3.5 | 1 | 24 | 644 | 667 | UGGGAUCUUCGAGAGGAUUGAGCC | GGGCAUAUCCUUUUGAGGAUCCUG | Cleavage |
| ghr-miR7491 | Gh_A13G1253 |  | 4.5 | 1 | 24 | 1254 | 1277 | UGGGAUCUUCGAGAGGAUUGAGCC | CAGAAGAUCUCCUAGAGGAUCUCA | Translation |
| ghr-miR7491 | Gh_D01G0453 |  | 5 | 1 | 24 | 1254 | 1277 | UGGGAUCUUCGAGAGGAUUGAGCC | CAUAAGGUCUCCUAGAGGAUCUCA | Translation |
| ghr-miR7491 | Gh_D01G0750 |  | 4.5 | 1 | 24 | 1618 | 1641 | UGGGAUCUUCGAGAGGAUUGAGCC | UGUGCAAGCCCCCCGGAGAUUCCA | Cleavage |
| ghr-miR7491 | Gh_D02G0839 |  | 3.5 | 1 | 24 | 587 | 610 | UGGGAUCUUCGAGAGGAUUGAGCC | GGGCAUAUCCCUUUGAAGAUCCUA | Cleavage |
| ghr-miR7491 | Gh_D02G2104 |  | 4 | 1 | 24 | 644 | 667 | UGGGAUCUUCGAGAGGAUUGAGCC | GAGCAUAUCCUUUUGAAGACCCCG | Cleavage |
| ghr-miR7491 | Gh_D05G2155 |  | 4.5 | 1 | 24 | 587 | 610 | UGGGAUCUUCGAGAGGAUUGAGCC | GGGCAUAUCCUUUUGAAGAUCCGG | Cleavage |
| ghr-miR7491 | Gh_D05G3352 |  | 4 | 1 | 24 | 1251 | 1274 | UGGGAUCUUCGAGAGGAUUGAGCC | CAUAAAAUCUCCUAGAGGAUCUCA | Translation |
| ghr-miR7491 | Gh_D08G1765 |  | 3.5 | 1 | 24 | 668 | 691 | UGGGAUCUUCGAGAGGAUUGAGCC | GAGCAUACCCUUUUGAGGAUCCCA | Cleavage |
| ghr-miR7491 | Gh_D10G0845 |  | 4.5 | 1 | 24 | 1235 | 1258 | UGGGAUCUUCGAGAGGAUUGAGCC | CUAUUAAACCUCUCCAAGCUCUCA | Translation |
| ghr-miR7491 | Gh_D11G0489 |  | 3 | 1 | 24 | 644 | 667 | UGGGAUCUUCGAGAGGAUUGAGCC | GGGCAUAUCCUUUCGAGGAUCCUG | Cleavage |
| ghr-miR7491 | Gh_D11G0552 |  | 4 | 1 | 24 | 588 | 610 | UGGGAUCUUCGAGAGGAUUGAGCC | GGCUUA-UCCUUUUGAAGAUCCUG | Cleavage |
| ghr-miR7493 | Gh_A02G0390 | 13 | 4 | 1 | 24 | 525 | 548 | AAUAUUUUAAUAAUUCAAUCGUCA | CAGUAAUUGCAUUGUUAAACUAUA | Cleavage |
| ghr-miR7493 | Gh_A05G1198 |  | 4.5 | 1 | 24 | 791 | 814 | AAUAUUUUAAUAAUUCAAUCGUCA | CAGCGAAGAAAUUAUUAAAACAUU | Cleavage |
| ghr-miR7493 | Gh_A05G1749 |  | 5 | 1 | 24 | 910 | 933 | AAUAUUUUAAUAAUUCAAUCGUCA | CGUGCAUUGGCUUAUUCAACUGUU | Cleavage |
| ghr-miR7493 | Gh_A08G0285 |  | 4.5 | 1 | 24 | 1047 | 1070 | AAUAUUUUAAUAAUUCAAUCGUCA | GCUAGUGGAAAUUAUUAAGAUAUU | Cleavage |
| ghr-miR7493 | Gh_A10G0452 |  | 3.5 | 1 | 24 | 791 | 814 | AAUAUUUUAAUAAUUCAAUCGUCA | CAGCCAUGAAAUUAUUAAAACAUU | Cleavage |
| ghr-miR7493 | Gh_A11G2528 |  | 5 | 1 | 24 | 371 | 394 | AAUAUUUUAAUAAUUCAAUCGUCA | ACCUUGUUGAAUUGGUUGGAUAUU | Translation |
| ghr-miR7493 | Gh_D02G0443 |  | 4 | 1 | 24 | 525 | 548 | AAUAUUUUAAUAAUUCAAUCGUCA | CAGUAAUUGCAUUGUUAAACUAUA | Cleavage |
| ghr-miR7493 | Gh_D05G1375 |  | 4.5 | 1 | 24 | 791 | 814 | AAUAUUUUAAUAAUUCAAUCGUCA | CAGCGAAGAAAUUAUUAAAACAUU | Cleavage |
| ghr-miR7493 | Gh_D08G0378 |  | 4.5 | 1 | 24 | 1038 | 1061 | AAUAUUUUAAUAAUUCAAUCGUCA | GCUAGUGGAAAUUAUUAAGAUAUU | Cleavage |
| ghr-miR7493 | Gh_D10G0469 |  | 3.5 | 1 | 24 | 791 | 814 | AAUAUUUUAAUAAUUCAAUCGUCA | CAGCCAUGAAAUUAUUAAAACAUU | Cleavage |
| ghr-miR7493 | Gh_D11G2830 |  | 5 | 1 | 24 | 839 | 863 | AAUAUUUUAAUAAU-UCAAUCGUCA | AGCUUGUUGAGAUUAUUAAGGUCUU | Cleavage |
| ghr-miR7493 | Gh_D11G2878 |  | 5 | 1 | 24 | 392 | 415 | AAUAUUUUAAUAAUUCAAUCGUCA | ACCUUGUUGAAUUGGUUGGAUAUU | Translation |
| ghr-miR7493 | Gh_D13G1374 |  | 4.5 | 1 | 24 | 601 | 624 | AAUAUUUUAAUAAUUCAAUCGUCA | AAGAGAUUGGGUUGUGGGGAUAUU | Cleavage |
| ghr-miR7495a | Gh_A12G0138 | 3 | 4.5 | 1 | 21 | 819 | 839 | UUACUUUAGAUGUCUCCUUCA | AAAAGUUGACAUCUGGAGUGC | Cleavage |
| ghr-miR7495a | Gh_A12G1672 |  | 4.5 | 1 | 21 | 51 | 71 | UUACUUUAGAUGUCUCCUUCA | GGAAGGAGACAUUUGGAUCAA | Cleavage |
| ghr-miR7495a | Gh_D12G0152 |  | 4.5 | 1 | 21 | 819 | 839 | UUACUUUAGAUGUCUCCUUCA | AAAAGUUGACAUCUGGAGUGC | Cleavage |
| ghr-miR7496a | Gh_A09G0712 | 2 | 5 | 1 | 24 | 306 | 329 | AUGACCAAAUUGAUAGAAUGUGUA | UGAGCUUUUUCUUAACUUGGUUAU | Cleavage |
| ghr-miR7496a | Gh_A09G0713 |  | 5 | 1 | 24 | 321 | 344 | AUGACCAAAUUGAUAGAAUGUGUA | UGAGCUUUUUCUUAACUUGGUUAU | Cleavage |
| ghr-miR7499 | Gh_A11G0927 | 2 | 5 | 1 | 24 | 45 | 68 | AUAUAAUUUUCGGUUAAUUCGGUU | GGUCCGACUAGUCGGAGACUAUAU | Cleavage |
| ghr-miR7499 | Gh_D11G1069 |  | 5 | 1 | 24 | 45 | 68 | AUAUAAUUUUCGGUUAAUUCGGUU | GGUCCGACUAGUCGGAGACUAUAU | Cleavage |
| ghr-miR7501 | Gh_A11G2311 | 2 | 3 | 1 | 24 | 1227 | 1250 | AUAUCUGAUUCUGACACGAAAAAA | UGAAAUGGUUUCAGAAUUGGAUAU | Cleavage |
| ghr-miR7501 | Gh_D11G2621 |  | 3 | 1 | 24 | 1227 | 1250 | AUAUCUGAUUCUGACACGAAAAAA | UGAAAUGGUUUCAGAAUUGGAUAU | Cleavage |
| ghr-miR7502 | Gh_A08G0593 | 4 | 5 | 1 | 24 | 1044 | 1067 | UUUUUAACAGUAGAAAUGAAUGAA | UGAAGAGAUUACUACUGUUGACAA | Cleavage |
| ghr-miR7502 | Gh_D01G1270 |  | 5 | 1 | 24 | 534 | 557 | UUUUUAACAGUAGAAAUGAAUGAA | CCACUCUAGUUCUGCUGUUGGAAU | Cleavage |
| ghr-miR7502 | Gh_D05G2533 |  | 5 | 1 | 24 | 225 | 248 | UUUUUAACAGUAGAAAUGAAUGAA | CAAGUUAAUCGCCGCUGUUAAAAA | Cleavage |
| ghr-miR7502 | Gh_D13G0738 |  | 4.5 | 1 | 24 | 2517 | 2540 | UUUUUAACAGUAGAAAUGAAUGAA | UAAUGCCAUGUUUGCUGGUAAAAA | Cleavage |
| ghr-miR7503 | Gh_A10G0892 | 6 | 4.5 | 1 | 24 | 894 | 917 | AGAUCGAUGGCUGAACAAGUUAGA | UUUUGCUUGUACAGCCUUUGAGCU | Cleavage |
| ghr-miR7503 | Gh_A10G1380 |  | 4 | 1 | 24 | 311 | 334 | AGAUCGAUGGCUGAACAAGUUAGA | GAUAUUUUUUUCAGCAGUUGAUCU | Cleavage |
| ghr-miR7503 | Gh_A12G0247 |  | 4 | 1 | 24 | 302 | 325 | AGAUCGAUGGCUGAACAAGUUAGA | GGUAUUUUUUUCAGCAGUUGAUCU | Cleavage |
| ghr-miR7503 | Gh_D10G0857 |  | 4.5 | 1 | 24 | 894 | 917 | AGAUCGAUGGCUGAACAAGUUAGA | UUUUGCUUGUACAGCCUUUGAGCU | Cleavage |
| ghr-miR7503 | Gh_D10G1083 |  | 4 | 1 | 24 | 311 | 334 | AGAUCGAUGGCUGAACAAGUUAGA | GAUAUUUUUUUCAGCAGUUGAUCU | Cleavage |
| ghr-miR7503 | Gh_D12G0247 |  | 4 | 1 | 24 | 380 | 403 | AGAUCGAUGGCUGAACAAGUUAGA | GAUAUUUUUUUCAGCAGUUGAUCU | Cleavage |
| ghr-miR7504a | Gh_A09G1965 | 2 | 5 | 1 | 24 | 1499 | 1522 | UAUGAAACUGUGAUUCCACGUCAU | UAGAGAUGGAAUUGGAGUUUCUUA | Translation |
| ghr-miR7504a | Gh_D09G2168 |  | 5 | 1 | 24 | 1499 | 1522 | UAUGAAACUGUGAUUCCACGUCAU | UAGAGAUGGAAUUGGAGUUUCUUA | Translation |
| ghr-miR7506 | Gh_A07G1806 | 3 | 4.5 | 1 | 24 | 305 | 328 | AUGUCUGGGACAUGGCGUUGGCAC | UACGCAGCUUCAUGUCUCAGAUGC | Cleavage |
| ghr-miR7506 | Gh_D07G2011 |  | 4.5 | 1 | 24 | 305 | 328 | AUGUCUGGGACAUGGCGUUGGCAC | UACGCAGCUUCAUGUCUCAGAUGC | Cleavage |
| ghr-miR7506 | Gh_D11G1445 |  | 4.5 | 1 | 24 | 305 | 328 | AUGUCUGGGACAUGGCGUUGGCAC | UACGCAGCUUCAUGUCUCAGAUGC | Cleavage |
| ghr-miR7507 | Gh_A01G0827 | 1 | 4.5 | 1 | 24 | 1059 | 1082 | AAGGUAGUGAAGUAGGCAAUUGGG | UUUCAUUGCUUUCUUUGCUACCCU | Cleavage |
| ghr-miR7508 | Gh_A06G1729 | 2 | 5 | 1 | 21 | 1125 | 1145 | CAAGAAAAGAAGUCGGGAGAG | UUCUCCCCGUCUCUUUUCUAG | Translation |
| ghr-miR7508 | Gh_D06G2249 |  | 5 | 1 | 21 | 1125 | 1145 | CAAGAAAAGAAGUCGGGAGAG | UUCUCCCCGUCUCUUUUCUAG | Translation |
| ghr-miR7510a | Gh_D07G1171 | 5 | 4 | 1 | 24 | 19 | 42 | AAGGUCAUGAUCUUUAGCGGCGUU | UCAUUUGCUAGAGAUUUUGGCUUU | Cleavage |
| ghr-miR7510a | Gh_D07G1608 |  | 4.5 | 1 | 24 | 757 | 780 | AAGGUCAUGAUCUUUAGCGGCGUU | AUAUCCGGCAAAGGUCAUGAUUUC | Cleavage |
| ghr-miR7510a | Gh_D09G1411 |  | 4.5 | 1 | 24 | 1648 | 1671 | AAGGUCAUGAUCUUUAGCGGCGUU | UCAAGCGAGAAGGAUCUUGAUCUU | Cleavage |
| ghr-miR7510a | Gh_D11G3249 |  | 5 | 1 | 24 | 1452 | 1475 | AAGGUCAUGAUCUUUAGCGGCGUU | CUUUGCGUUAAAGAUCAUGGACAA | Cleavage |
| ghr-miR7510a | Gh_D13G2312 |  | 5 | 1 | 24 | 736 | 759 | AAGGUCAUGAUCUUUAGCGGCGUU | AAAGAUAUUAAAGAUUAUGACCAG | Cleavage |
| ghr-miR7510b | Gh_A10G1128 | 1 | 5 | 1 | 23 | 522 | 544 | AAGAACAUGAUCUUUAGCGGCGU | UUCGGUCUACAGAUUAGGUUCUU | Cleavage |
| ghr-miR7511 | Gh_A03G1241 | 12 | 5 | 1 | 24 | 1008 | 1031 | AGAAGUUUUGCAUGUGUAGCUGAG | UUUCUGUACAAAUGCAAAACGCCU | Cleavage |
| ghr-miR7511 | Gh_A08G1693 |  | 4.5 | 1 | 24 | 1453 | 1476 | AGAAGUUUUGCAUGUGUAGCUGAG | GUGGGAUACACAUACAAAAAUUUU | Translation |
| ghr-miR7511 | Gh_A09G2007 |  | 5 | 1 | 24 | 1313 | 1336 | AGAAGUUUUGCAUGUGUAGCUGAG | ACGAGUUGGACACGCAAAAUUUCG | Cleavage |
| ghr-miR7511 | Gh_A10G1361 |  | 5 | 1 | 24 | 31 | 54 | AGAAGUUUUGCAUGUGUAGCUGAG | AAGGGUCGUACAUGUAAACCUUUU | Cleavage |
| ghr-miR7511 | Gh_A12G1469 |  | 4.5 | 1 | 24 | 1468 | 1491 | AGAAGUUUUGCAUGUGUAGCUGAG | GAGAAAUGCAAAUGUGGAGCUUCU | Cleavage |
| ghr-miR7511 | Gh_D01G0855 |  | 4.5 | 1 | 24 | 1866 | 1889 | AGAAGUUUUGCAUGUGUAGCUGAG | GAAGUCUCUAUGUGCAAAGCUUUC | Cleavage |
| ghr-miR7511 | Gh_D05G0320 |  | 4.5 | 1 | 24 | 1408 | 1431 | AGAAGUUUUGCAUGUGUAGCUGAG | GUGGGCUAUACAUACAAGAAUUUU | Translation |
| ghr-miR7511 | Gh_D05G0513 |  | 4 | 1 | 24 | 168 | 191 | AGAAGUUUUGCAUGUGUAGCUGAG | AUCUUCUGAAUAUGUAAAGCUUCC | Cleavage |
| ghr-miR7511 | Gh_D06G0995 |  | 4 | 1 | 24 | 588 | 611 | AGAAGUUUUGCAUGUGUAGCUGAG | UUCUUCUGAAUAUGUAAAGCUUCC | Cleavage |
| ghr-miR7511 | Gh_D08G2051 |  | 4.5 | 1 | 24 | 1453 | 1476 | AGAAGUUUUGCAUGUGUAGCUGAG | GUGGGAUACACAUACAAAAAUUUU | Translation |
| ghr-miR7511 | Gh_D09G2220 |  | 5 | 1 | 24 | 1313 | 1336 | AGAAGUUUUGCAUGUGUAGCUGAG | ACGAGUUGGACACGCAAAAUUUCG | Cleavage |
| ghr-miR7511 | Gh_D12G1597 |  | 4.5 | 1 | 24 | 1537 | 1560 | AGAAGUUUUGCAUGUGUAGCUGAG | GAGAAAUGCAAAUGUGGAGCUUCU | Cleavage |
| ghr-miR7512 | Gh_A01G0246 | 7 | 4.5 | 1 | 21 | 995 | 1015 | UGCUACUUGUAGUUAUGCAUG | GGAACAUAGCUUCGAGUAGCA | Translation |
| ghr-miR7512 | Gh_A02G0024 |  | 4.5 | 1 | 21 | 1168 | 1188 | UGCUACUUGUAGUUAUGCAUG | AUUGUACUACUACAAAUGGCA | Cleavage |
| ghr-miR7512 | Gh_A09G0260 |  | 4 | 1 | 21 | 1169 | 1189 | UGCUACUUGUAGUUAUGCAUG | AUUCCAUUGCUGCAAGUAGCC | Cleavage |
| ghr-miR7512 | Gh_D01G0244 |  | 4.5 | 1 | 21 | 995 | 1015 | UGCUACUUGUAGUUAUGCAUG | GGAACAUAGCUUCGAGUAGCA | Translation |
| ghr-miR7512 | Gh_D02G0038 |  | 4.5 | 1 | 21 | 1174 | 1194 | UGCUACUUGUAGUUAUGCAUG | AUUGUACUACUACAAAUGGCA | Cleavage |
| ghr-miR7512 | Gh_D06G2142 |  | 5 | 1 | 21 | 68 | 88 | UGCUACUUGUAGUUAUGCAUG | UUUCCACAACUAUUGGUGGCA | Cleavage |
| ghr-miR7512 | Gh_D09G0260 |  | 4 | 1 | 21 | 1169 | 1189 | UGCUACUUGUAGUUAUGCAUG | AUUCCAUUGCUGCAAGUAGCC | Cleavage |
| ghr-miR7513 | Gh_A09G0371 | 4 | 2.5 | 1 | 21 | 878 | 898 | AAUCAGCCAGGAAUCGUUUGA | AAGAACGAUUCCUUGUUGAUU | Cleavage |
| ghr-miR7513 | Gh_A11G0581 |  | 3 | 1 | 21 | 866 | 886 | AAUCAGCCAGGAAUCGUUUGA | AAGAACGGUUCCUUGUUGAUU | Cleavage |
| ghr-miR7513 | Gh_D09G0393 |  | 2.5 | 1 | 21 | 878 | 898 | AAUCAGCCAGGAAUCGUUUGA | AAGAACGAUUCCUUGUUGAUU | Cleavage |
| ghr-miR7513 | Gh_D11G0666 |  | 2.5 | 1 | 21 | 866 | 886 | AAUCAGCCAGGAAUCGUUUGA | AAGAACGAUUCCUUGUUGAUU | Cleavage |
| ghr-miR7514 | Gh_A12G2159 | 2 | 4 | 1 | 24 | 1091 | 1113 | AUAAAGUGAUAAGUGAGAUCGUCU | GGAAUAUC-CACUUUUCAUUUUAU | Translation |
| ghr-miR7514 | Gh_D12G2336 |  | 4.5 | 1 | 24 | 1091 | 1113 | AUAAAGUGAUAAGUGAGAUCGUCU | GGAACAUC-CAUUUUUCAUUUUAU | Translation |
