# Supplementary material for: GBS Mapping and Analysis of Genes Conserved between Gossypium tomentosum and Gossypium hirsutum Cotton Cultivars that Respond to Drought Stress at the Seedling Stage of the BC2F2 Generation
Source: Int J Mol Sci. 2018 May 30;19(6):1614. doi: 10.3390/ijms19061614 (PMC6032168; doi:10.3390/ijms19061614)
Supplement: Supplementary file 1 [file ijms-19-01614-s001.zip › Supplimentary/Supplementary Table 8 List of primers used for qRT-PCR validation.docx]

Supplementary Table 8: List of primers used for qRT-PCR validation

| Gene ID | Primer name | Forward sequence | Reverse sequence |
| --- | --- | --- | --- |
| Gh_A01G0730 | PKN_1 | AGCTTGCATTGAGCCCTC | TCCCATTTTATTCTTCGG |
| Gh_A01G0842 | PKN_2 | GATAACTGGACGGAAGGC | TTTTAAGTACGGACGGGA |
| Gh_A02G0300 | PKN_3 | TACCAGAGTGGAAGAGGG | GATAAGTAATCCGAAAGG |
| Gh_A03G0665 | PKN_4 | TCTAAAGGGAGGAGTGGCT | TATGGGAGATCATGGTCAA |
| Gh_A06G1588 | PKN_5 | GAAGCACACTCAATCCTC | ATCTCCTCACTACCACCT |
| Gh_A06G1729 | PKN_6 | CGCAATGAAAGTTATGGAC | AGAGTAGGAAGGAACGGAT |
| Gh_A07G0492 | PKN_7 | GCGAGAAGATGACAAAGA | AACCTGACCAAAAGTACC |
| Gh_A08G0085 | PKN_8 | GAGTCTATAGGTCGTGCC | CTTATTCCCATTTTCATC |
| Gh_A09G0260 | PKN_9 | TGCTTTTCCTGAAGGTTTT | ATCTCTGTGAATGTGCCCA |
| Gh_A09G0371 | PKN_10 | CAAGGTGGATTTGGGGCTG | CGGGTGACGCAAGAGAGAA |
| Gh_A09G2123 | PKN_11 | TAAAGGCCGGTTGGAGAGT | GTTGACGAGGTTTGGGTGG |
| Gh_A11G0665 | PKN_12 | AAGAAACCAAGAAACCAACC | GCGTCATTACTAAAGCCAAG |
| Gh_A11G1297 | PKN_13 | AATCTTCCTCTTACACTCC | GATCTATTTCACTTTCACC |
| Gh_A11G1858 | PKN_14 | TATCAGGAGTGAGTTATTG | TATTTTTAGTCTGGGTGTA |
| Gh_A12G0021 | PKN_15 | AAATAAGTCTATGGTGGC | GCTGAAGATGTGGGTGAG |
| Gh_A12G0247 | PKN_16 | GTATGGTCTTGTGGGGTGA | GTCTGGGATTTTGTATTGA |
| Gh_A12G1469 | PKN_17 | TGAAATGGCAGAGGGACTT | GGAGCTGGCTCAATAGATA |
| Gh_A12G1556 | PKN_18 | GCAGCCCCTCAATTCCTA | CCTACCCCCACTCCCACA |
| Gh_D01G0869 | PKN_19 | GAGTTGTTTTCCCTGCTT | CGTGCTCCACTACCTTTG |
| Gh_D02G0364 | PKN_20 | TTAATGGCAACCAAGGGG | GTTCCGACACAACGAGCG |
| Gh_D05G0411 | PKN_21 | GGTGGTCATTAGGAGCAA | TTCAGGAAATCTTAGGTG |
| Gh_D05G0750 | PKN_22 | AATATTGTGCATGGCGAT | GCAGTGAAAACAGGAGTC |
| Gh_D06G1942 | PKN_23 | TTTCACTTTCTTACGCCT | ATCTCCTCGCTACCACCT |
| Gh_D06G2142 | PKN_24 | GGAAATGTCTACCCCTGT | GCTCTGCCATATAACTAA |
| Gh_D06G2249 | PKN_25 | GATCATCCGTTCCTTCCTA | CCCTGGTTGCTTCTGCCTA |
| Gh_D07G0202 | PKN_26 | ACACTTGATTATCTCCCT | CTCATAGCACAACACACC |
| Gh_D07G0582 | PKN_27 | GTAAATGGTGGAGGGGAC | CAAATAGACATAAGCGAA |
| Gh_D09G0260 | PKN_28 | TGCTTTTCCTGAAGGTTTT | ATCTCTGTGAATGTGCCCA |
| Gh_D09G1173 | PKN_29 | TCCTCTCCCGATTTTGTAT | AGCATTTGGTCTGTGTTCC |
| Gh_D11G0594 | PKN_30 | AAGTGGCAGTGAAGCAAGT | GGCGAATAATGTTAGGGTG |
| Gh_D11G1445 | PKN_31 | GGGTTCACAATCGCATCA | ACCGTCGGCACACTTACT |
| Gh_D11G2830 | PKN_32 | CTTACATCCACCGCAGCAT | CGCACTTCCAAAATCACAC |
| Gh_D11G3249 | PKN_33 | ATGTCTGGTTCGTGTGGA | TTGGTCTTACCGTCTTTG |
| Gh_D12G1659 | PKN_34 | CCAAAGCCAGCCCCTCAAT | GCAACCCCACCCCTACTCC |
| Gh_D13G0352 | PKN_35 | TTATGTTGGTTGGTGCTT | TCTGGGATTTTGTATTGA |
